# Short-term feeding of defatted bovine colostrum mitigates inflammation in the gut via changes in metabolites and microbiota in a chicken animal model

**DOI:** 10.1186/s42523-023-00225-z

**Published:** 2023-01-26

**Authors:** Ted H. Elsasser, Bing Ma, Jacques Ravel, Stanislaw Kahl, Pawel Gajer, Alan Cross

**Affiliations:** 1 Animal Biosciences and Biotechnology Laboratory, USA Department of Agriculture (USDA), Agricultural Research Service (ARS), Beltsville, MD 20705 USA; 2 Institute for Genome Sciences, University of Maryland School of Medicine, Baltimore, MD 21201 USA; 3 Center for Vaccine Development and Global Health, University of Maryland School of Medicine, Baltimore, MD 21201 USA

**Keywords:** Anti-inflammation, Colostrum, Ileum, Immunometabolism, Metabolome, Gut microbiome, Protein tyrosine nitration

## Abstract

**Background:**

Nondrug supplement strategies to improve gut health have largely focused on the effects of individual compounds to improve one aspect of gut homeostasis. However, there is no comprehensive assessment of the reproducible effects of oral, short-term, low-level colostrum supplementation on gut inflammation status that are specific to the ileum. Herein, a chicken animal model highly responsive to even mild gut inflammatory stimuli was employed to compare the outcomes of feeding a standard diet (CON) to those of CON supplemented with a centrifuge-defatted bovine colostrum (BC) or a nonfat dried milk (NFDM) control on the efficiency of nutrient use, ileal morphology, gut nitro-oxidative inflammation status, metabolites, and the composition of the microbiota.

**Results:**

A repeated design, iterative multiple regression model was developed to analyze how BC affected ileal digesta-associated anti-inflammatory metabolite abundance coincident with observed changes in the ileal microbiome, mitigation of epithelial inflammation, and ileal surface morphology. An improved whole body nutrient use efficiency in the BC group (v CON and NFDM) coincided with the observed increased ileum absorptive surface and reduced epithelial cell content of tyrosine-nitrated protein (NT, biomarker of nitro-oxidative inflammatory stress). Metabolome analysis revealed that anti-inflammatory metabolites were significantly greater in abundance in BC-fed animals. BC also had a beneficial BC impact on microbiota, particularly in promoting the presence of the bacterial types associated with eubiosis and the segmented filamentous bacteria, Candidatus Arthromitus.

**Conclusion:**

The data suggest that an anti-inflammatory environment in the ileum was more evident in BC than in the other feeding groups and associated with an increased content of statistically definable groups of anti-inflammatory metabolites that appear to functionally link the observed interactions between the host’s improved gut health with an observed increase in whole body nutrient use efficiency, beneficial changes in the microbiome and immunometabolism.

**Supplementary Information:**

The online version contains supplementary material available at 10.1186/s42523-023-00225-z.

## Background

The One Health concept [1] recognizes the interdependence between food animal and human health and includes such issues as nutrition, appropriateness of antibiotic use, zoonotic pathogen transfer, the emergence of antimicrobial resistance and alternatives to antimicrobial drug therapies. More specifically, it recognizes the commonality of physiological systems of and interdependence between food animal health and human health [2]. For decades, antibiotic drugs (ABDs) were administered to animals in their food for the purpose of enhancing their growth [3–5]. This practice has possible links to the increased incidence of antimicrobial resistance, a major public health concern. Although the mechanisms by which ABD impact growth have been debated, the diminution of the local inflammatory stress in the gut was attributed to the now recognized non-antimicrobial, anti-inflammatory effects of select ABDs, particularly in the tetracycline/doxycycline/minocycline and macrolide families (4–8). Much of the diminution of local inflammatory stress in the gut was coincident with the improved efficiency with which feed was channeled into tissue deposition in the ABD-fed animals [6, 7]. Through legislative actions, the subtherapeutic level use of ABDs for growth promotion in animals has now been restricted; consequently, other strategies for promoting growth via reduced gut inflammation in food animals were deemed necessary [3, 5] and was highlighted by the National Research Council [5]. Our current study addresses one such alternative with a specific focus on mitigating low-level inflammation.

Over the last 10 years the traditional concept of “nutrition” has evolved from feeding the body to now include feeding the microbes that inhabit microenvironments and niches throughout the digestive tract [8, 9]. The nuances of nutrition now include not only the composition of the foods and supplements ingested and the way the digestive system processes these nutrients, but also must address the interactions of the gut microbiota with both the ingested nutrients and the host’s gastrointestinal cells. The importance of the interaction between the microbes and the host cells, and the nutrient-derived metabolites that function as signaling molecules between the two” compartments” is now encompassed in the term “immunometabolism” [10, 11]. The model developed in our laboratory which was aimed at integrating multiple physiological systems [11] suggests that the regulation of immunometabolism occurs within the interactive relationships between endocrine, immune, metabolic, and microbial inputs to result in the preferential partitioning of nutrients to different tissue beds in times of health or stress.

Mammals “jump-start” the immune system of their offspring at birth by providing the nutrient-rich and physiologically stabilizing first milk, the colostrum. Colostrum not only contains fat, protein, mineral, and vitamin nutrients, but also immunoglobulins, antimicrobial peptides, hormones, oligosaccharides, and a plethora of small molecules derived from the diet and physiological processes of the mother. Bovine colostrum is rich in *Bifidobacterium* and *Lactobacillus* phylotypes and this combination of compound constituents along with the beneficial bacteria promotes gut maturation, mucosal integrity and tissue repair [12] with these beneficial effects attributed to these components being transferred to the young in their initial suckling. The composition and volume of colostrum rapidly changes within days following parturition, as milk production evolves over time [13]. To date, the vast majority of studies that concern the beneficial effects of colostrum have examined either aspects of neonatal organ maturation or the ability to thrive in the extrauterine environment [14]. Still other studies have explored the potential for vaccine-induced antibodies in colostrum (through immunization of cows prior to parturition) to mitigate complications of parasitic and bacterial gut infections [15–18]. Few studies have comprehensively examined the effects of colostrum supplementation on gut health with the simultaneous assessment of changes in a whole-body parameter such as feed use efficiency, impact on the gut microbiome, the patterns of intestinal digesta-associated anti-inflammatory metabolites, and host gut cell responses. In the present study, we hypothesized that the actions of some metabolite constituents of the ileal digesta as modified by bovine colostrum intake can combine in an additive manner or synergize to mitigate inflammation resulting more efficiently in improved nutrient use. We used mass spectroscopic metabolomics and S16 rRNA microbial analysis on the same luminal digesta samples along with quantitative measurement of intestinal morphometrics and a defined epithelial cell nitro-oxidative stress inflammation marker (3′-tyrosine-nitrated proteins, NT) to study the interactions between gut-modifying metabolites of the bovine colostrum diet and the mitigation of low-level inflammation in the ileum. A positive association between a BC-supplemented diet and reduced gut inflammation would be consistent with the outcomes on growth and nutrient use reported with ABDs but devoid of the issues of drug residues and antimicrobial resistance [19]. To explore this further, we examined the effect of short-term feeding of a diet containing semi-purified, centrifuge-defatted bovine colostrum (BC) to a commercial line of meat chickens (broilers). While these animals are characterized as rapidly growing with high nutrient use efficiency under ideal management conditions, they also are highly sensitive to even mild stresses that negatively impact nutrient use efficiency as associated with increased gastrointestinal perturbation. Though less studied than oxidative stress (cell stress responses that mainly generate post-translational carbonyl modifications to proteins), nitro-oxidative stress (i.e., cell responses that result in aberrant intracellular post-translational protein tyrosine nitration) is a significant additional component of such gut disturbances [20, 21]), indicative of sustained gut inflammation [22–24]). We report that the refined colostrum supplement improved feeding efficiency consistent with the observed increase in the absorptive surface area of the ileum and alterations in the gut microbiota and digesta metabolites that reflect an overall increase in the anti-inflammatory capacity of the luminal environment highlighted by a significant decrease in gut epithelial protein NT content.

## Results

Short-term BC supplementation of the standard diet significantly improved feeding efficiency and consistently modified the metabolic and bacteriological milieu of the gut compared to CON and in a manner superior to that of NFDM. To examine these effects in greater detail, we particularly focused on the ileum. We found diet-associated changes in epithelial anatomy, anti-inflammatory metabolites and gut microbiota of the ileum.

### Feeding efficiency was significantly improved in short-term BC diet-fed chickens

**Fig. 1:**
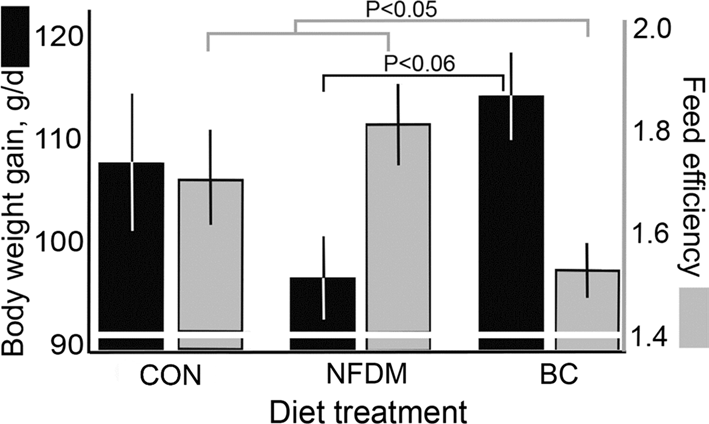
BC-supplemented diet improved feed efficiency. Mean body weight gain ± SEM (grams/day) and feed efficiency (grams feed consumed per gram body weight gained) in chickens fed the base diet (Control, n = 10) or diets supplemented with either nonfat dry milk (NFDM, n = 10) or a processed bovine colostrum preparation (BC, n = 20)

The impact of feeding diets supplemented with BC or the nonfat dry milk-supplement (NFDM, a macronutrient balanced control to BC) compared to CON on weight gain and feeding efficiency is summarized in Fig. 1. For chickens fed the BC diet, the feed efficiency was improved approximately 9 percent (*P* < 0.05) over CON and NFDM diets. Although the lowest and highest mean weight gain responses were observed in NFDM and BC chickens, respectively, the differences between the means of these treatment groups as well as the difference from CON were only trends (*P* > 0.05 < 0.1).

### Intestinal measurements indicate significant biological impacts of the BC diet on gut morphology

**Fig. 2:**
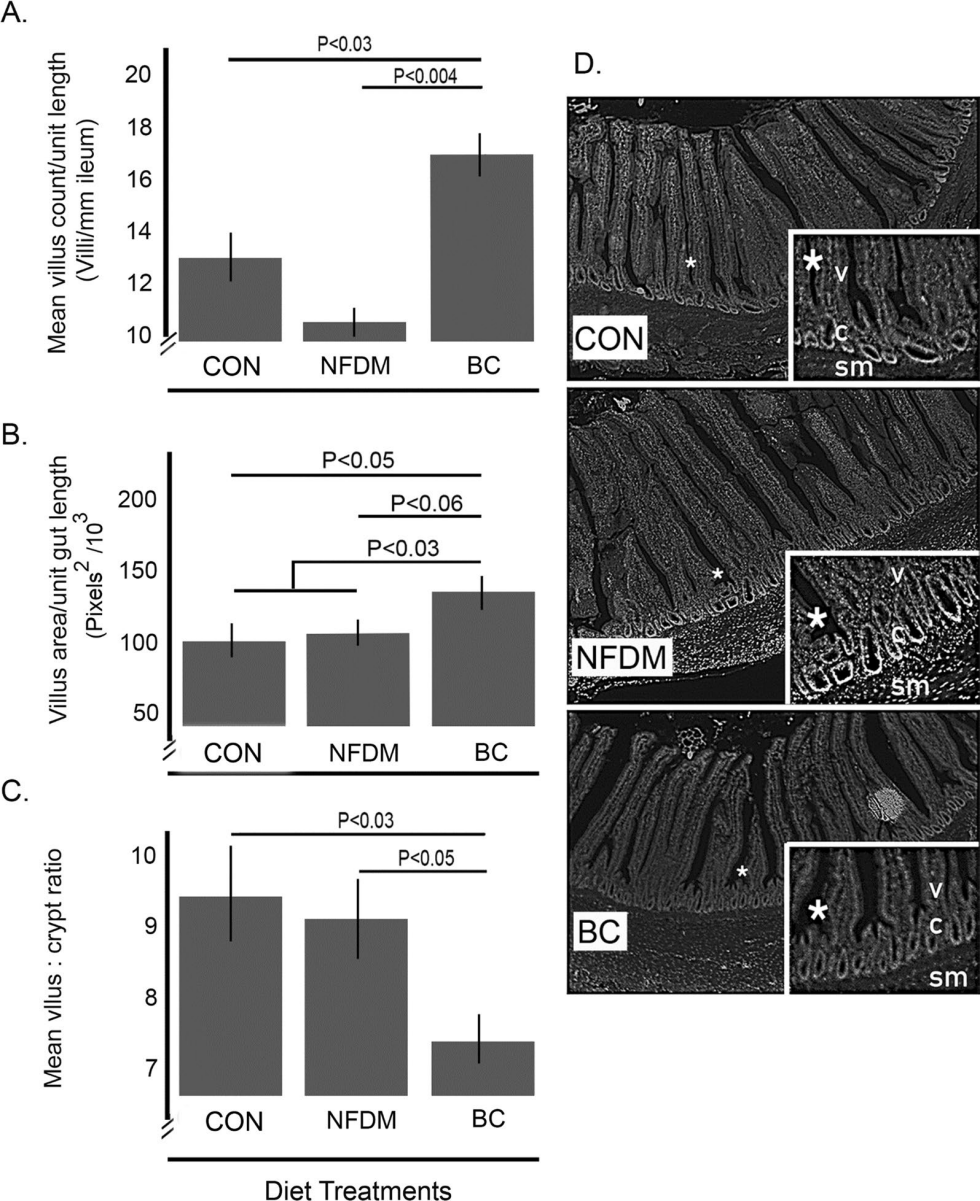
The BC-supplemented diet increases the villus surface abundance. The effects of inclusion of nonfat dry milk (NFDM) or the bovine colostrum fraction (BC) to the standard feed (Control) on the morphology of the ileum. Data represent the means ± SEM of the number of villi counted per standardized length of tissue (Panel** A**) or the calculated area of the villi as associated with the diet treatments fed to the chickens (Panel **B**), or the ratio of the villus height to crypt depth (Panel **C**). Panel **D** depicts representative images of the diet-associated ileal architecture. (Control, basal diet, n = 6. NFDM. n = 6. BC, n = 10). For reference, “V” = villi, “C” = crypts, and “SM” = submucosa

A visual depiction of the ileum structure attributes as associated with the three diets as well as measured and estimated morphological attributes of the ileum are summarized in Fig. 2. Where nuclei are depicted as white objects against the black/grey background, the increased number of nuclei in the lamina propria of villi of animals in the CON and NFDM groups, higher than that in observable in BC, is consistent with a larger number of infiltrating immune cells, a hallmark of inflammation or at the least, a sensed perturbation. Presented as an expression of the density of villi, the number of villi per linear unit of ileum was significantly increased in animals fed the BC diet in contrast to CON (*P* < 0.03) and NFDM (*P* < 0.004, Panel A). When the relative villus area (RVA, mid-villus width x the villus length x number of villi per unit villus length) was calculated for each animal and the mean group RVAs compared, the calculated area for BC was 42 percent (*P* < 0.05) and 24 percent (*P* < 0.06) greater than the mean areas calculated for CON and NFDM, respectively (Panel B). Mean villus lengths were 265, 280 and 250 µm for CON, NFDM and BC, respectively (*P* = NS, data not shown). By comparison, corresponding mean crypt depths, associated with enterocyte cell proliferation and digestion capacity of the small intestine, of 28, 31 and 37 µm were observed for CON, NFDM and BC, respectively, yielding the resulting villus-to-crypt ratios (Fig. 2, Panel C). Though the mean values calculated for villus length and crypt depths alone across the dietary treatments did not attain statistical significance, the mean villus:crypt ratio for chickens in BC was 27 percent lower (*P* < 0.03) and 24 percent lower (*P* < 0.05) than that calculated for CON and NFDM, respectively (Panel B). The value for the calculated estimate of villus area per unit length was significantly increased in BC compared to the other fed diets (Panel C). Collectively, the increase in estimated villus surface area with BC is consistent with an effect of BC to stimulate a more efficient use of nutrients and the observed improved feed efficiency in BC.

### BC- and to a lesser extent nonfat dry milk (NFDM)-supplemented diets are associated with less ileal epithelial cell nitro-oxidative stress

**Fig. 3:**
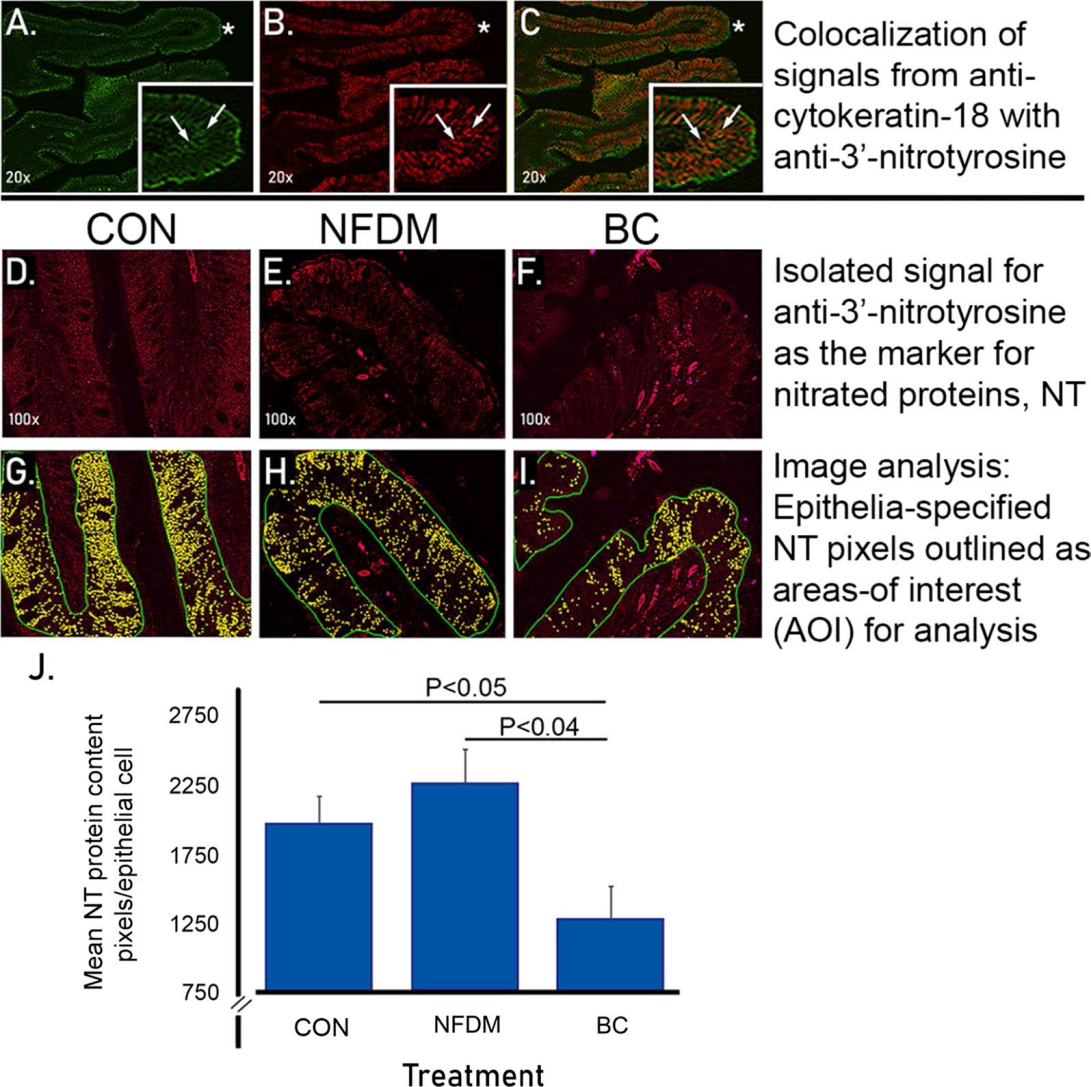
Decreased protein tyrosine nitration (NT) in epithelial cells of villi in samples of ileum from chickens fed a BC-supplemented diet. Immunofluorescence is presented in Panels **A**–**F** and Image analysis depicted in Panels **G**–**I**. Epithelial cell (anti-cytokeratin 18 epithelial cell marker) presence is indicated by green fluorescence pixels (**A**, **C**). NT, as detected using an anti-nitrotyrosine antibody marker, is presented as red (cell presence, **B** and **D**–**F**, for isolated single channel image analysis quantification) or orange (colocalized in green cytokeratin 18-positive cells, **C** Representative patterns of image analysis-selected nitrotyrosine pixels (**G**–**I**, yellow pixels) were summated and processed to yield the statistical analysis summarized in the lower panel bar graph(**J**). Values are group means ± SEM for Control (n = 6), NFDM (n = 6), and BC (n = 10)

We measured the cellular pixel density associated with immunofluorescence localization of NT, on the exterior, epithelial layer of the villi, thereby excluding lamina propria and interior layers of the villi that contain infiltrating immune inflammatory cells. Though nitrated proteins were observed in the gut epithelial cells of all chickens, the highest levels were detected in those chickens fed the CON and NFDM diets (Fig. 3G, H, I). The epithelial cell NT protein content, characterized as pixel density as normalized by nuclear counting as a measure of the number of cells specified in the demarcated area of interest, was significantly decreased by 37% in BC-fed chickens compared to that present in chicken tissues from either the CON (*P* < 0.05) or the NFDM (*P* < 0.04) diets (Fig. 3J). The overall decrease in NT pixel density per epithelial cell was attributable to “clusters” of nitration elements that were both fewer in number and smaller in the contained number of pixels. The lower level of nitrated proteins in the ileal epithelial cells suggests that these cells were less subject to nitro-oxidative stress in the presence of BC-derived diet than were the ileal cells of chickens fed the Con and NFDM diets.

### Short-term feeding of BC- and NFDM-supplemented diets alters the anti-inflammatory metabolite profile of the ileum

We focused on the identified metabolite digesta compounds derived from the animal treatment diets themselves or as further derived from host and/ or microbial enzymatic action on these compounds with a literature-documented capacity to mitigate tissue inflammation. Thus, the goal was to identify those anti-inflammatory compounds derived from NFDM or BC present in amounts over and above those contributed through consumption of the basal CON diet that had a potential to lower the NT proteins and then test them in a statistical model for association with any impact on ileum inflammation status. Across dietary treatments, metabolomic profiling of the ileal digesta revealed 649 named and 300 unnamed compounds (Additional file 8: Data S1)*.* Quantified levels of the identified compounds in the metabolite data base in our third-party analytical contract (Metabolon, Inc., Morrisville, NC) were reported to us as “scaled imputed values (SIV, see “Methods” and Additional file 9: Data S2 for full definition and derivation).

The digesta of BC was significantly more complex in metabolite richness (number of detected compounds) than that of CON or NFDM. Plant-derived compounds are well-known for having anti-inflammatory and anti-nitro-oxidative properties and as such were of significant interest here. Of seventy-seven compounds in the ileal digesta classified as xenobiotics of plant origin identified at or higher than levels stated as the threshold limits of detection (indicated as “DETECTED” in the accompanying table), 40 compounds were in CON, 46 in NFDM and all 77 in BC (Additional file 5: Table S1). Interestingly, though beyond the scope of the present paper, data in Additional file 5: Table S1 are also presented for the sake of relative comparison of the composition of digesta in the ileum compared to that in the duodenum and cecum. These data clearly demonstrate that the availability of metabolites changes with the host-microbiome interactions that process the digesta as it flows down the alimentary canal. This finding is consistent with the concept that anti-inflammatory compounds available in one gut segment might not be available in other gut segments to provide their beneficial effects and that bacterial populations differ between gut segments. Our considerations for inclusion of a metabolite compound in a list of compounds with diet-derived anti-inflammatory character stemmed from a PubMed (https://pubmed.ncbi.nlm.nih.gov/) search for literature citations of studies investigating such biochemical modes of action of qualifying compounds with specific reference to compounds in the digesta identified by the metabolomic separation and analysis (Additional file 10: Online References S1). The output from a Boolean search using the terms “polyphenol, flavonoids, bile acids, amino acids, fatty acids, or oligosaccharides along with small intestine, metabolite, and inflammation or anti-inflammation” yielded 1968 citations and formed the basis of our assessments.

With focus on the ileum, ten xenobiotic compounds with or without anti-inflammatory character were found to be significantly different in abundance between CON and BC and reproducibly across both treatment trials. Apigenin, 2,3-dihydroxyisovalerate, carotenediol, daidzein, daidzein sulfate, genistein, gluconate, naringenin, and 2-piperidinone were higher in BC than CON and panose (a purported prebiotic) lower, respectively. Similarly, digesta from NFDM chickens contained six more compounds than were present in the digesta from CON with 2,8-quinolinediol sulfate, 2-oxindiole-3-acetate, 1-kestose, 1,1-kestotetraose higher and homostachydrine lower.

The effects of the NFDM and BC diets on the changes in these compounds as well as those identified in the other relevant seven pathway metabolite groups are presented in Table 1. We identified numerous compounds with potential anti-inflammatory activity (as cited in the literature) in the ileal digesta. Comparing the levels of the identified metabolites in the ileum digesta between the diet treatments, data revealed, perhaps not unexpectedly, that the mean levels of many individual metabolites varied numerically, sometimes greatly, among the three diet treatments and the two replications of the experiment. Further analysis revealed that fewer individual metabolites consistently differed in the two experimental replications between CON and BC or CON and NFDM. This became apparent with respect to the animal-to-animal variation and replication variation for many of the metabolites. However, when multiple related metabolites for a given common pathway and mechanism of anti-inflammatory action were grouped and concentration levels considered additively as a singular entity (for example, if several anti-inflammatory compounds like flavonoids/isoflavones or active metabolites like glutathione and its major precursors), the abundance of a select set of compounds/groups were significantly greater for BC than those measured in CON or NFDM. The literature-supported anti-inflammatory pathway metabolite categories included tryptophan indole-conjugates, tocopherols (α-, γ-, δ-tocopherols), flavonoids/isoflavones (apigenin, daidzein, genistein, and their conjugates), primary and secondary bile acids (chenodeoxycholic acid, 7-ketolithocholic acid), glutathione (N-acetylglycine, γ-glutamylcysteine, cysteine,), polyunsaturated fatty acid (docosahexaenoic acid, arachidonic and eicosapentaenoic acids), oligosaccharides (3′- and 6′-sialyllactose), and mitochondrial TCA cycle compounds (itaconate). (see Additional file 10: Anti-inflammatory Metabolite References in the online material),

**Table 1:**
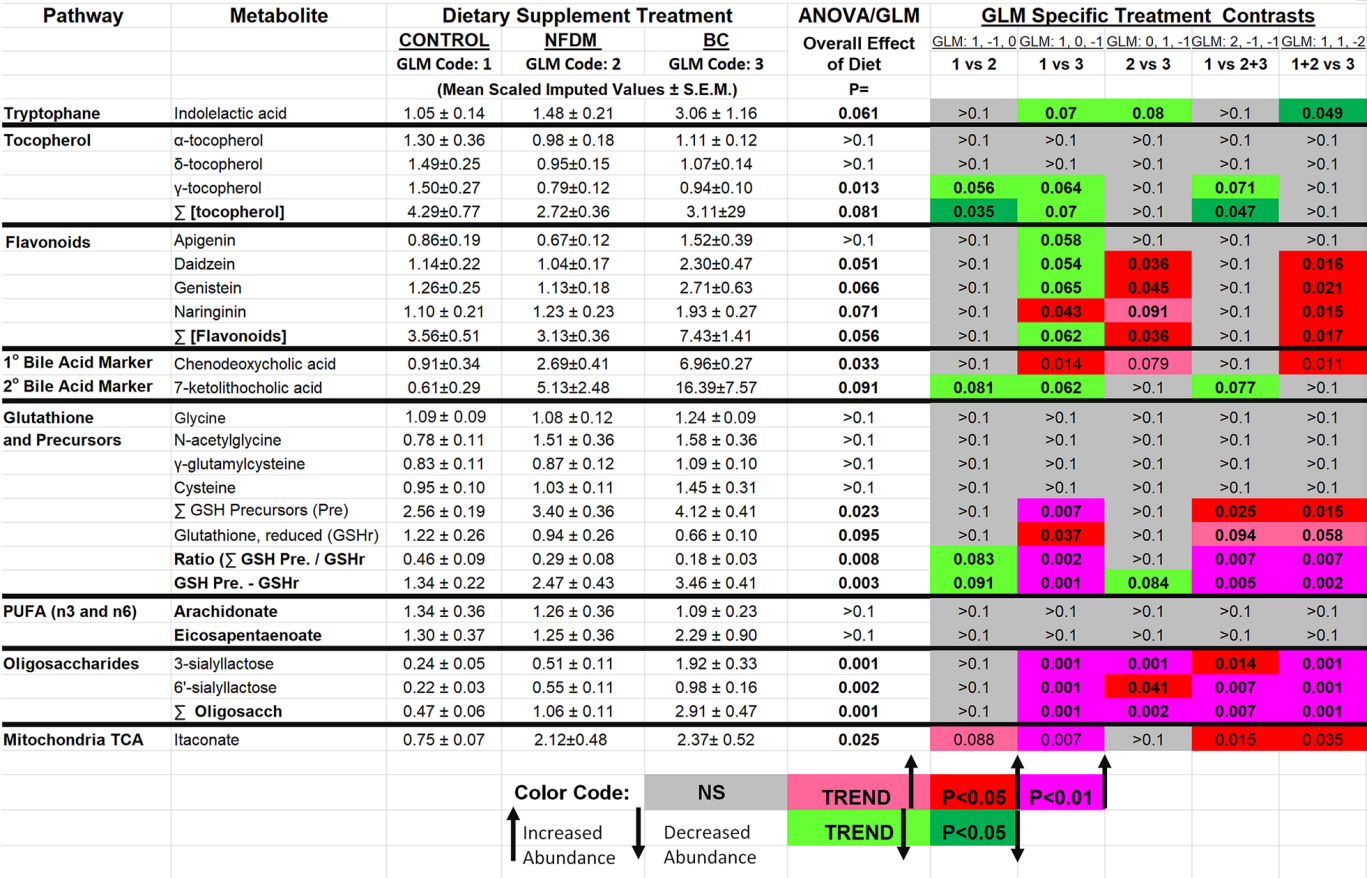
The effects of inclusion of nonfat dry milk (NFDM) or the bovine colostrum fraction (BC) on ileal digesta components with literature-supported anti-inflammatory effects on tissues varied according to the involved biochemical pathway components

Data represent the means ± SEM of the Scaled Imputed Values of metabolites that were increased by either BC or NFDM identified by the metabolomic analysis to be consistently changes across the two experimental replicates and the source(s) of the colostrum. Control, basal diet, n = 11. NFDM. N = 11. BC, n = 20. General Linear Model (GLM) contrast statement coding and weighting indicated in line 2 under GLM Specific Treatment Contrasts. The statistical comparisons of Scaled Imputed Values between control (#1), NFDM (#2) and BC (#3) treatments are shown in the far-right columns with the shades of red colors indicating increases or shades of green colors indicating decreases in significance (or trends). Comparisons highlighted in grey are not significant.

We also observed some significant effects of diet on the potent tripeptide anti-inflammatory compound glutathione and its precursors in the luminal digesta which are critical to the anti-nitro-oxidative capacity of the gut. The metabolomic report contained data on the reduced form of glutathione, GSHr. Overall, the mean level of GSHr in BC was half that present in CON (*P* < 0.04) and 70 percent of that present in the digesta of NFDM (NS). However, the digesta from chickens in BC had a summated glutathione precursor pool of compounds significantly increased over that measured in CON. This is consistent with the need for an abundance of precursors (cystine, glycine, glutamate, and associated dipeptides) being available to the synthesis pathway in the gut epithelial cells to replenish glutathione as it is consumed in countering nitro-oxidative processes. When considered as a precursor group, the summated mean of glycine, cystine, N-acetylglycine, and γ-glutamylcysteine digesta abundances averaged 158 percent greater levels in BC than in CON (3.46 ± 0.46 v 1.34 ± 0.22, *P* < 0.001). Both the difference in summated abundance (*P* < 0.001) as well as the ratio (*P* < 0.002) of GSH_precursors_-to-GSHr may reflected the potential for BC to increase the anti-nitro-oxidative capacity of the gut over that present in CON by contributing to the metabolites the gut needs to make glutathione.

Statistical evaluation of the pathway components in Table 1 showed that polyunsaturated fatty acid (PUFA) compounds, and especially metabolites of eicosapentaenoic and arachidonic acids, were not significantly affected by diet (all grey). This lack of change with BC feeding may relate to the defatting process used to generate the BC matrix and the NFDM supplemented feeds. However, seven other identified pathways were significantly affected by diet either as single compounds or, as summed components of an individual pathway. For some pathways, for example tryptophan or tocopherols, only a single pathway compound with purported anti-inflammatory potential was significantly different between one or among all diets. Only indolelactic acid in the tryptophan pathway or γ-tocopherol in the tocopherol pathway, respectively, were affected by inclusion of nonfat dry milk or the colostrum matrix into the basal diet. Surprisingly, the effect amounted to a reduction in relative mean ileal SIV. The overall impact of this reduction in ileal gamma-tocopherol content was sufficient to account for the majority of the pathway change in total tocopherols.

The inclusion of BC to the basal diet consistently increased the ileal digesta content of four anti-inflammatory flavonoid compounds: apigenin, daidzein, genistein, and naringin. In contrast, none of the compounds was affected by adding NFDM to the basal diet. The effect of adding BC to the diet resulted in a doubling of the amount of flavonoid present in the gut digesta based on summated SIVs. Data from the larger metabolite profiling indicated that the levels of the flavonoids in their conjugated forms (glucoside, glucuronide) did not contribute to the gut levels of the parent compound’s anti-inflammatory status suggesting that the parent compounds alone were to be considered the major components of interest.

The summated values of the primary and secondary bile acids with anti-inflammatory effects, chenodeoxycholic acid and 7-ketolithocholic acid content, respectively, were significantly elevated in digesta from BC and NFDM compared to CON diets. However, digesta from BC-fed chickens tended to have higher levels of chenodeoxycholic acid than that from NFDM (Table 1).

Only two oligosaccharides were identified by the mass spectroscopic analysis, 3′-sialyllactose and 6′-sialyllactose. Levels of total oligosaccharides in BC digesta were 5.7-fold higher (*P* < 0.002) than those measured in CON digesta and 2.8-fold (*P* < 0.003) higher than those in digesta from NFDM.

The cellular energetics generated by subcellular mitochondrial processes is a fundamental requisite of cell stability, immune system function, and the efficiency of nutrient use. Of the eleven mitochondrial compounds associated with energy production identified in the digesta, only one, itaconate, derived from the decarboxylation of cis-aconitate in the mitochondrial matrix, was an anti-inflammatory metabolite and found to be increased in digesta in NFDM and BC relative to CON (*P* < 0.03).

### Short-term colostrum extract supplementation (BC) is associated with a significantly higher Anti-inflammatory index (A-i-i) than found in CON or NFDM diets.

**Fig. 4:**
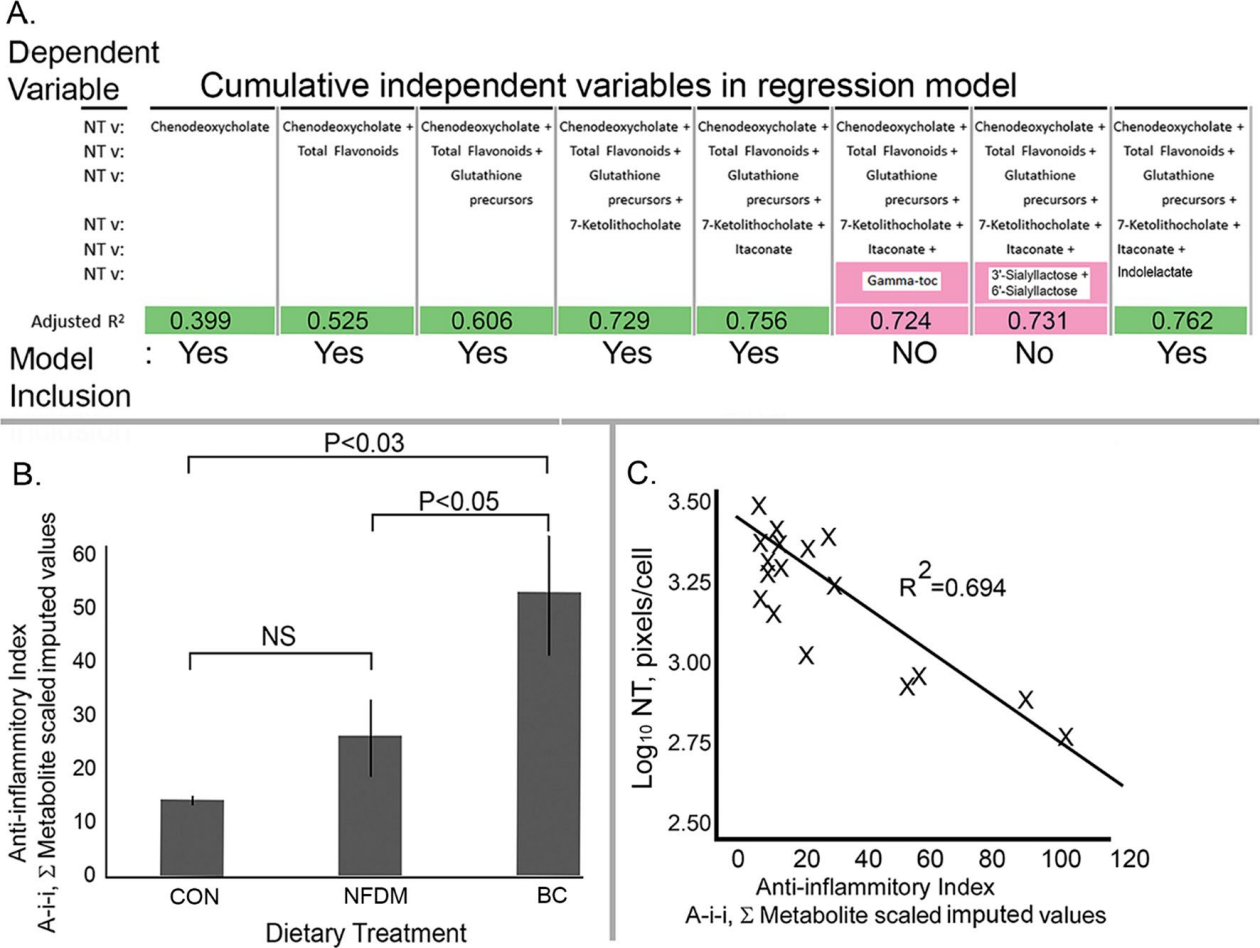
Correlation between anti-inflammatory index (A-i-i) and epithelial cell protein nitration (NT). Multiple regression was used to assess the relationship between the level of NT content (Log10, pixels/cell) present in intestinal epithelial cells and the independent metabolite variables. Inclusion of a metabolite (class) to the model was valid if the resulting adjusted R^2^ was increased over that of the prior iterative assessments (Panel **A**). The effects of dietary treatment on the A-i-i (the sum of the Scaled Imputed Values of the individual metabolites retained in the model, Panel **B**). Linear regression of the NT pixel content on the A-i-i suggested a significant negative correlation where the greater the A-i-i- the lower the nitration stress (Panel **C**)

The relative contribution of each of these metabolites to our model was assessed according to whether the inclusion of a particular metabolite or metabolite group improved the adjusted R^2^ in the multiple regression model, and not all did. To facilitate our analysis of the impact of dietary-derived metabolites on chicken gut inflammation, we devised an anti-inflammatory index and found associations arising from the gut processing of the ingested feed components of the specific diets that correlated with protein nitration (Fig. 4). First, the data used to construct Panel A of Fig. 4 were derived from the SIV values in the metabolomic database. As indicated by the terms “Yes” and “No” in the table line “Model Inclusion”, as each successive variable was added to the regression model, that variable was only retained in the model if it added a positive incremental increase to the adjusted R^2^. While some metabolites present in the digesta of chickens may have had a purported anti-inflammatory character, if they did not have added a benefit in the regression model to increase the adjusted R^2^ (terms shaded in green) they were dropped from the model (terms shaded in pink). Consequently, we excluded gamma-tocopherol and the two sialyloligosaccharides from the cumulative Ai-i, based on the fact that the adjusted R^2^ resolved with these metabolites included in the model was lower than that resolved before either metabolite was added to the model. Therefore, in the final assessment, the combination of chenodeoxycholic acid, total flavonoids, glutathione precursors, 7-ketolithocholate, itaconate and indolelactate accounted for approximately 76 percent of the observed decline of cellular NT protein content. Second, BC feeding yielded mean Ai-i values (derived from data in Panel A) significantly different from and higher than the average Ai-i’s from both NFDM and Con groups (Panel B). Third, the variability in the magnitude of the derived NT protein pixel quantification data among treatment groups could be better analyzed with respect to a linear model after performing a logarithmic transformation of the pixel data. When this was done, the regression analysis showed a significant (*P* < 0.005) negative linear correlation (R^2^ = 0.694) wherein the higher the Ai-i, the lower the NT protein in villus epithelial cells (Panel C).

### BC and NFDM added to a standard diet altered specific populations of bacteria in the ileum rapidly and specifically.

**Fig. 5:**
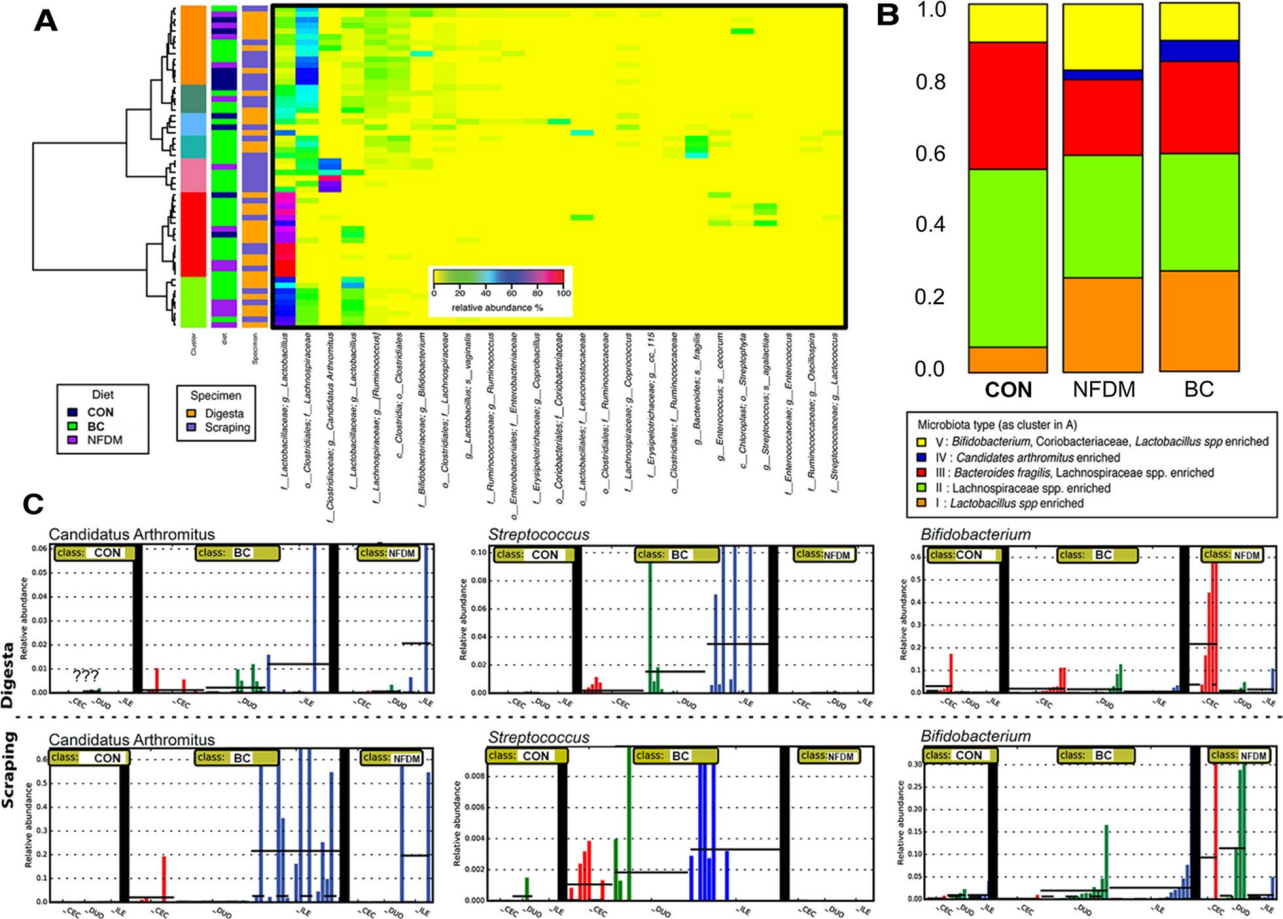
Chicken intestinal microbiota analyses. **A** Heatmap of the 50 most abundant phylotypes of 156 samples collected. Five types of intestinal microbiota were revealed based on clustering patterns. Ward linkage clustering is used to cluster samples based on their Jensen-Shannon distance calculated in vegan package in R [116]. Identified microbiota types are labeled as I–V. **B** Distribution of different community type by cluster as shown in A in diet treatment groups of Control, colostrum and NFDM. Type I is significantly lowered in control than in other two groups while type IV is significantly higher in BC and NFDM. Type II is borderline significantly higher in BC and NFDM than control, based on statistical modeling using Bayesian Poisson model. **C** Phylotype biomarkers for digesta and scrapings generated using program LEfSe [105]. Vertical bars represent the relative abundance of Bacteriodes in each sample. Dotted line represents mean, solid line represents median relative abundance. The alpha value for the non-parametric factorial Kruskal–Wallis (KW) sum-rank test was 0.05 and the threshold for the logarithmic LDA model (43) score for discriminative features was set at 2.0

**Fig. 6:**
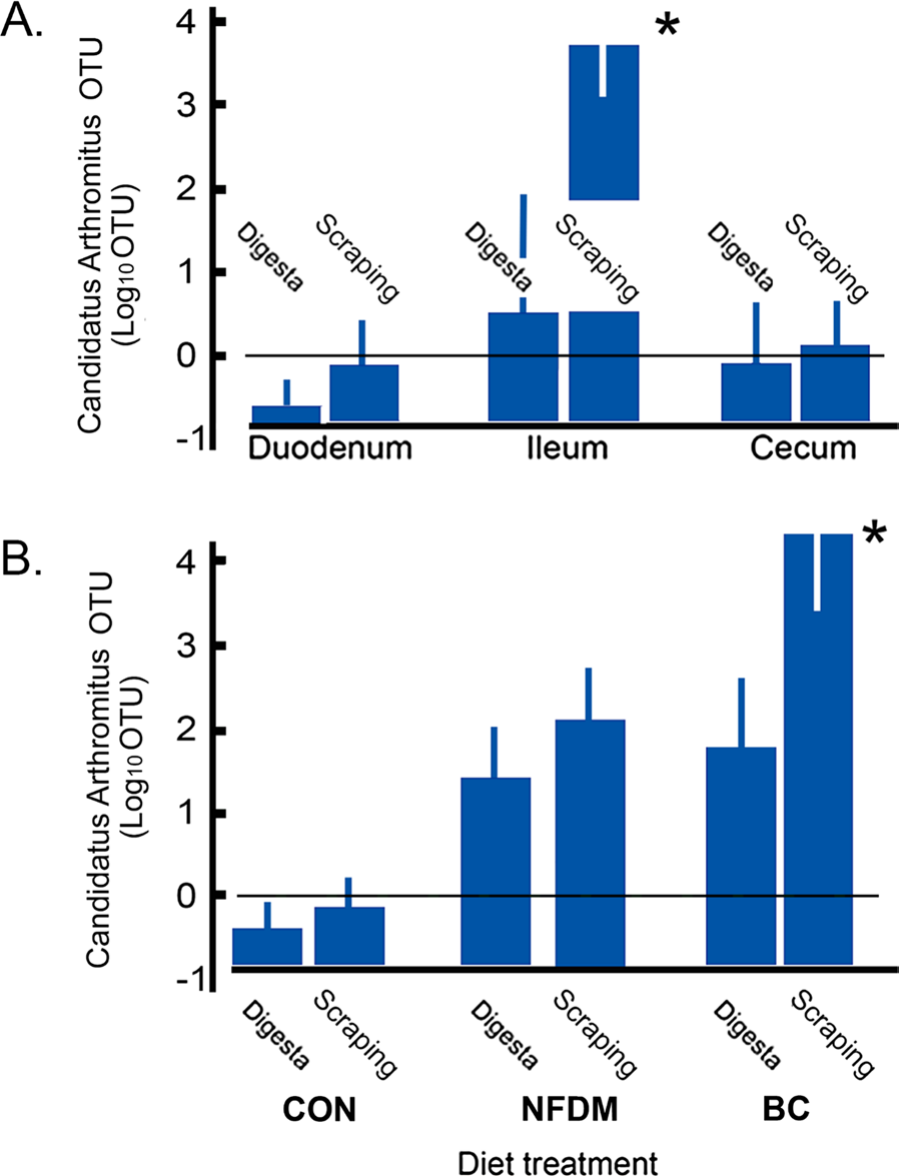
Candidatus Arthromitus is present in the ileal scraping and increased by BC. Mean (± SEM) abundance of Candidatus Arthromitus represented as Log10 [OTU] in the three different segments of the gut and further differentiated by digesta vs. scraping (Panel **A**). Mean (± SEM) abundance of Candidatus Arthromitus represented as Log10 [OTU] in the ileum as measured in digesta or cell scrapings (attached) as affected by diet. *P < 0.04 (Panel **B**)

Using 16S rRNA gene amplicon sequencing, we performed a community survey of the gut microbiota of 41 chickens under 3 different dietary conditions (standard or CON feeding, and CON feeding supplemented by NFDM or BC). We collected intraluminal digesta from the proximal and distal intestine including jejunum, duodenum, and cecum for a total of 156 samples as well as scrapings from ileal epithelial cells washed free of digesta. We obtained a total of 25,838,078 high-quality 16S rRNA V4 amplicon sequences corresponding to 51,165 (± 2,267, μ ± s.e.m.) sequences per sample (Additional file 6: Table S2). Overall, we found distinct differences in the digesta and mucosal-associated microbiota among animals fed the three different diets. Across diets, five distinct types or groupings of intestinal microbiota, Type I–Type V, were observed and summarized in the heat map and bar graph (Fig. 5, Panels A and B); Type I is enriched in *Lactobacillus* spp., Type II is enriched in *Lachnospiraceae* spp., Type III is enriched in both *Lachnospiraceae* spp. and *Bacteroides fragilis*, Type IV is characteristic of Candidatus Arthromitus, and Type V is enriched in *Bifidobacterium*, Coriobacteriaceae spp. and *Lactobacillus* spp**.** Type I and IV microbiota had the lowest community diversity (Additional file 1: Figure S1) and were highly enriched with *Lactobacillus* and Candidatus Arthromitus, respectively (Fig. 5 Panel B). In particular, the different physiological sections of the intestine demonstrated distinct microbiota (Additional file 2: Figure S2), and the Type IV microbiota was enriched in the ileal microbiota. The other types of microbiota were significantly more diverse and had relatively more abundant *Lachnospiraceae* spp*.*, *Bacteroides fragilis* and a wide array of strict and facultative anaerobic bacterial species (Additional file 7: Table S3). Statistical modeling using Bayesian Poisson model (details in Methods and (Additional file 3: Figure S3) showed the intestinal microbiota in the digesta of BC-fed chickens was significantly enriched in Type I and IV microbiota. We further performed LDA effect size (LEfSe) analyses to quantitatively characterize the phylotypes that could explain the differences observed under different conditions (Fig. 5C, (Additional file 1: Figure S4). Candidatus Arthromitus was shown to be the most differentially abundant phylotype in ileal mucosal scrapping samples of BC-fed animals but not in ileal digesta (Fig. 6A). This finding is consistent with the nature of the cell attachment mechanism this organism employs to persevere in its growing microenvironmental niche largely specific to the ileum. Consolidating across all data comprising Fig. 5 and Additional file 2: Figure S2 and Additional file 4: Figure S4, the patterns, distribution, and abundance of microbes is consistent with BC having greater microbial diversity than that in the digesta from animals in the other two diets. Increases in this microorganism in the colon might reflect remaining pass-through organisms originating in the ileum and perhaps simply shed into the digesta going into the large intestine in association with the naturally-occurring programmed epithelial cell losses from the tips of the villi associated with apoptosis. In addition, Candidatus Arthromitus was minimally detected in the cecum or duodenum digesta or scrapings (Fig. 5C, left side).

In order to determine whether the presence of Candidatus Arthromitus might have arisen in the BC chickens as a result of feeding the actual processed colostrum fraction that contained a live Candidatus Arthromitus, we characterized the composition of the microbiota associated with various samples of the bovine colostrum used in the diets, and showed that these samples contained very low abundance of bacteria in general and revealed no *Lactobacillus* nor Candidatus Arthromitus. While fresh colostrum is known to contain many microorganisms beneficial to the nursing infant, the lack of such microbial abundance in our BC diet preparations is consistent with the effects of the 1–h centrifugation of the preparations at 20,000×*g* to sediment bacteria into the bottom of the tube. Further analyses confirmed no detected Candidatus Arthromitus via reads mapping to published Candidatus Arthromitus genomes or using marker gene-based approach (Additional file 7: Table S3). This result indicated that the Candidatus Arthromitus was not likely derived from the bovine colostrum diet.

Other than Candidatus Arthromitus, *Streptococcus* was another phylotype that was shown to be enriched in BC-fed animals, irrespective of the sub-anatomical location (digesta vs. mucosa-associated) (Fig. 5C). This result again emphasizes the importance of sub-anatomical location, anatomic site, and dietary conditions for specific groups of bacteria in stark contrast to data more commonly generated from fecal collection sampling. Other phylotypes that demonstrated varied enrichment under different feeding conditions were also included (Fig. 5C). For example, *Bifidobacterium* were more enriched with the NFDM diet, and *Bacteroides fragilis*, *Bacteroides ovatus*, and *E. coli*, was particularly enriched in cecal samples. Together, we observed that Candidatus Arthromitus was only in the mucosa-associated microbiota of the ileum in these chickens and its presence was amplified significantly when BC was incorporated into the basal diet (Fig. 6A, B). The higher relative abundance of Candidatus Arthromitus is consistent with a localized proliferation of the organism, most likely due to factors in the environment of the organism rather than being sourced from the fed colostrum itself.

**Fig. 7:**
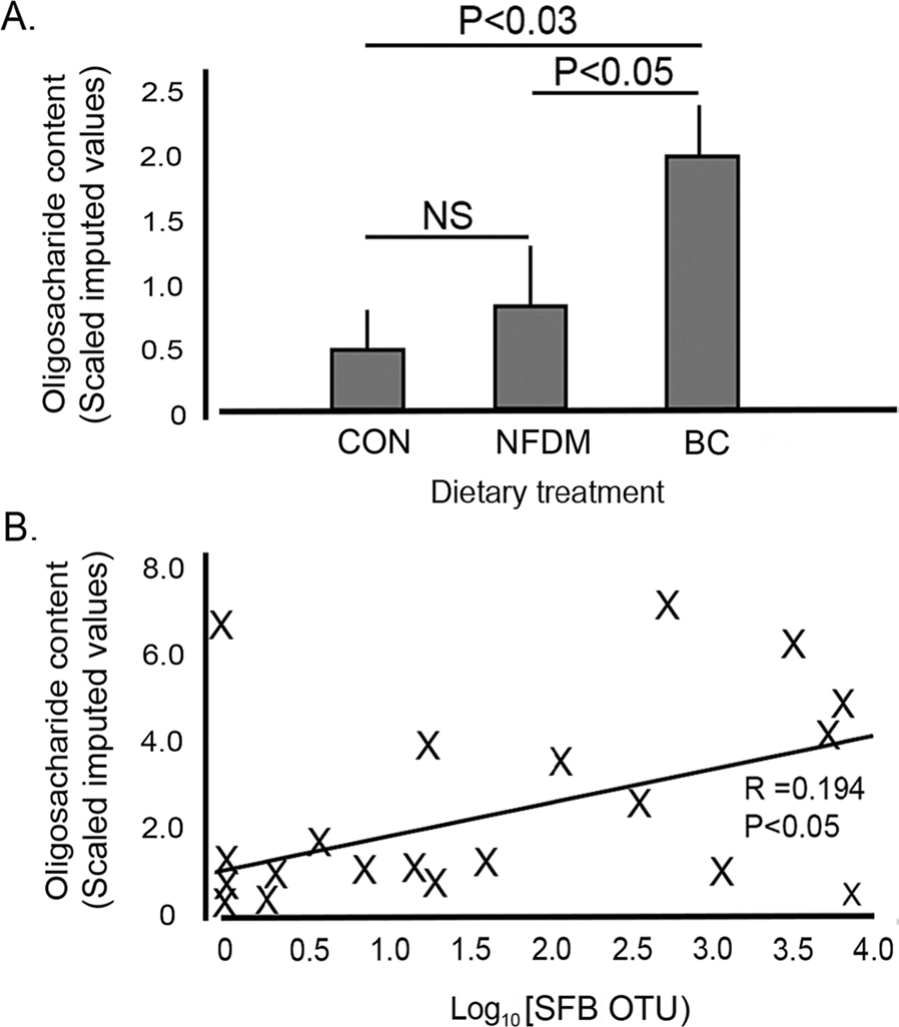
Sialyllactose content of ileal digesta is correlated with presence of SFBs. Ileal digesta content of the combined mean (± SEM) levels of the 3′-sialyllactose and 6'-sialyllactose represented as the metabolomic Scaled Imputed Values as measured in samples from chickens fed the three diets (Panel **A**). Summated 3′-sialyllactose and 6′-sialyllactose values were regressed on the Log10 [OTU of the SFB] yielding a significant positive linear correlation. (Panel **B**)

While the oligosaccharide content of the various diets did not affect the anti-inflammatory index, the 3′- and 6′-sialyl-oligosaccharides did lead to a dramatic site-specific increase in Candidatus Arthromitus. The oligosaccharide content of the ileal digesta was increased approximately 4- and 2.5-fold in BC compared to that measured in digesta from CON and NFDM, respectively (Fig. 7). Further regression analysis between the SIVs for the combined oligosaccharides versus the log_10_ of the abundance of total Candidatus Arthromitus indicate a significant positive correlation wherein higher levels of the oligosaccharides in the digesta were associated with increased numbers of the Candidatus Arthromitus with its potential impact on the immune capacity of the gut.

## Discussion

Significant increases in growth rate of domestic food animals have been linked to some of the nonantibiotic, anti-inflammatory effects of ABDs [5, 6], with more recent trends towards increased popularity of the use of supplements with purported anti-inflammatory effects [25]. Therefore, we addressed whether the anti-inflammatory gut benefits of ABDs could be mimicked by the feeding of defatted colostrum and further, whether a model could be developed to better define what aspects of colostrum consumption fostered these beneficial impacts. Our results were based on the simultaneous assessment of how a diet enriched in a partially refined BC preparation differed from other diets in their impact on the host gut morphology, the gut metabolite milieu, and the gut microbiota with particular emphasis on the ileum. The terminal ileum segment of the gut was focused on because of its role as the transition segment between the small and large intestine, the unique presence of the immune-modulating segmented filamentous bacteria, Candidatus Arthromitus, and further in light of the relative paucity of research compared to that of the cecum and colon on integrated host, metabolome, and microbiome interactions.

Research from our laboratory has demonstrated that nitro-oxidative stress occurs in the intestine as observed with the increased tissue content of nitrated proteins in conjunction with parasitic infection and correlated with the severity of infection [26, 27]. In comparison to extensive research done on oxidative stress in the gut, there is less research on nitration stresses in the gut, even though nitration stress and oxidative stress share a pool of cell response-induced common oxygen- and nitrogen-based reactants (nitric oxide and superoxide anion [20, 24]). With peroxynitrite (ONOO^−^) substantively being the principal effector relating to this form of inflammation, the term nitro-oxidative stress therefore encompasses the result of nitric oxide (the “nitro” component) and superoxide anion (the “oxidative” component) reacting to generate the ONOO^−^ insult [22, 23, 28, 29]. The present study demonstrates that the fractionated preparation of BC developed in this study, when added to a standard feed and consumed by animals for 8 days, is associated with a significant reduction of the amount of nitro-oxidative inflammation in the epithelial cells of the ileum, denoted as a reduction in the content of intracellular NT proteins. In concert with this effect, feeding BC was strongly associated with an increase in the absorptive surface area of the ileum. The rapidity with which this occurred is consistent with an increased capacity to generate epithelial cells [30] as reflected in the calculated crypt-to-villus ratio. These observations on the increased ileal surface area are also consistent with the observed improvement in nutrient absorption as reflected in feed utilization efficiency. The presence of naturally-occurring, low-level gut stressors such as changes in environment and social structure, present in our select, high growth rate animal model, has been identified previously as having the capability of significantly altering the architecture of the villus absorptive area as well as the morphogenesis of the crypts [31–33].

Elevated levels of intracellular tyrosine-nitrated (3’-nitrotyrosine-) NT proteins are a cause of protein dysfunction in non-immune cells, as documented in many tissue inflammation pathologies [23, 24, 29]. Epithelial cells generate highly reactive nitrogen intermediates (i.e., peroxynitrite, ONOO^−^) from nitric oxide and superoxide anion [23, 24, 28], which can react with critical tyrosine(s) in phosphorylation sites of many critical intracellular proteins including signal transduction enzymes such as JAK-2 [34, 35] or mitochondrial proteins thereby causing dysfunction and ATP shortage in cells [28, 36, 37]. Additionally, epithelial cells can have levels of NT proteins with cell function deficits that vary by diet in the absence of detectable infectious disease [26, 27]. Oxidative/nitration protein and lipid damage has been generated in chickens simply by low levels of partially oxidized corn oil or polyunsaturated fatty acids being in the diet [38]; similarly, that oxidized oil-induced inflammation was relieved with the addition of the flavonoid antioxidant quercetin to the chicken diet [39]. Mitigation of gut dysfunction by reducing inflammation is a goal of both human and veterinary medicine. In the present study the short-term feeding of the refined BC matrix was significantly associated with the observed reduction in ileal epithelia-specific NT protein content.

Gut metabolism in health can account for up to 20 percent of maintenance energy expenditure [40–42]. Gut inflammation increases energy demands by as much as 10 percent of the body’s metabolic energy need [43, 44] with a significant portion of the caloric substrates diverted from growth and muscle accretion to support immune function particularly in young animals [22, 24]. Microbial populations in the gut, and more specifically changes in such populations, significantly impact host immune and metabolic gene expression [45] and therefore the energetic needs of the host’s physiological systems. Signals from the gut microbiome can prioritize the flux of nutrients between the nervous, endocrine, and immune systems and shape their interactions [22, 24, 46, 47]. Since oxidative and nitration stress impacts the energy utilization of affected cells, even marginal changes in gut health result in energetic deficiencies [48, 49] that rapidly translate into growth and nutrient use efficiency deficits but improved significantly in BC. These observations are consistent with the regulatory model proposed for the controlling inputs to immunometabolism [50] and further impacts on nutrient use in the young growing animal.

The enhanced genetic lines of commercial production chickens with a high growth rate used in our chicken model required no direct disease or chemical challenge to trigger gut nitrooxidative stress. Further, low levels of nitrated proteins were shown to be generated by enterocytes in response to non-infectious perturbations such as changes in feed composition, allergens, endogenous bacterial endo- and exotoxins, mold toxins, and even metabolic ketosis [51–53]. In the past, this stress sensitivity was managed in part through the extensive use of antibiotics added to the diet [3, 5]. However, herein we report that consumption of components of BC can ameliorate inflammation in this gastrointestinal situation and improve nutrient use.

Dietary factors are potent modulators of the microbiota composition and its interaction with the host [54]. Reciprocally, microbes metabolizing ingested nutrients and generating nutrient-derived metabolites play a critical role in regulating the host immune response and gut cell function, as documented in the newly emerging area of immunometabolism [55]. Active microbial modification of feed-derived nutrients was evident (Additional file 8: Data S1) as observed in the presence of microbe-derived compounds like p-cresol sulfate and hippurate as well as deconjugation of flavonoid compounds differentially affecting their bioavailability [56–59] and levels of the various secondary bile acids consistent with literature reports [57]. From the metabolomic analysis of the ileal digesta of test animals fed the various dietary treatments, we were able to identify and statistically model a defined set of nutrient molecules and microbially-derived metabolites that correlated with the reduction in level of nitrooxidative stress present in the ileal epithelial cells. The positive effects of the colostrum feeding were reflected in the increased portion of bacteria with anti-inflammatory/ immunomodulatory properties in the microbiota profiles, and the generation of anti-inflammatory metabolites bathing the cells of the lumen.

We determined that classes of related microbially-derived metabolites should be considered for their anti-inflammatory effect in addition to the more commonly literature-cited approach of identifying single compounds. For example, the polyphenol flavonoids identified in the mass spectroscopic analysis revealed that as a class they were significantly increased in BC and NFDM feeding in comparison to CON. If, however, only one flavonoid, apigenin, of the identified group was assessed, the larger group effect would have been missed. Similarly, while the effect of adding NFDM or BC to the basal diet on the individual components identified in the anti-inflammatory glutathione pathway may have been insignificant (i.e., *P* > 0.1), collectively the sum of the precursor pool and the glutathione components improved the regression model (Table 1). This is consistent with the need for an abundance of precursors (cystine, glycine, glutamate, and associated dipeptides) being available to the synthesis pathway in the gut epithelial cells to replenish glutathione [60, 61] as it is consumed and to maintain cellular oxidative/nitrosative homeostasis.

Metabolites are an important link in the interactions between the host, its gut microbiota and the lumen environment. For example, metabolites such as metabolically-derived itaconate, generated during the immune response and identified in our screening can regulate both the magnitude and duration of the immune response [62]. In addition, Henrick et al. established the cause-and-effect relationships between gut increases in the tryptophan pathway metabolite, indolelactate, also identified in our metabolomic screening, concurrent with the enhanced presence of bifidobacteria, and their beneficial effects on the balance between inflammatory and anti-inflammatory states [63]. The capacity for microbe-derived indolelactate to function as an anti-inflammatory metabolite [64, 65] is consistent with our findings of the reduction in nitrated proteins, the increased nutrient use efficiency, and favorable microbial populations in BC- compared to CON- or NFDM-fed chickens. These previously published data lend support to the validity of our regression analysis that identified indolelactate among the many metabolites in the digesta. Collectively, the present data demonstrate the potential for diet composition to significantly impact gut health through changes in the microbiota, the gut metabolite milieu and host responses.

Specific metabolites generated by the localized bacterial populations acting on ingested dietary components facilitate processes needed for healthy eubiotic regulation of the gut epithelia. In studies of the murine colonic microbiota, Tiffany and Baumler [66] proposed that during gut homeostasis, bacteria of the phyla *Firmicutes* and *Bacteroidete*s, obligate anaerobic bacteria, fermented dietary fiber and maintained a hypoxic environment that could reduce colonization with *Enterobacteriaceae* (phylum *Proteobacteria*). In the absence of hypoxia, *Proteobacteria* gained a foothold. This deviation from an obligate anaerobic microbiota was defined as gut “dysbiosis”. Dysbiosis in the ileal microbiome stems from lower levels of microbial diversity with imbalances between both beneficial and pathogenic organisms as well as disproportionate imbalances in commensal bacteria that can change the local microenvironments downstream thus facilitating a bacterial overgrowth syndrome or pathogen emergence [67, 68]. Thus, in the present study, the increased diversity and favorable ratios in microbial populations in the BC animals would be consistent with a greater opportunity for eubiosis and sound gut health to be maintained.

The microbiome of the small intestine, the primary site for the absorption of nutrients, differs substantially from that of the colon both in terms of the number of colony-forming units per ml as well as microbial composition [69]. We focused on the ileum in this study, largely because of the large information gaps on the microbiome and its host interactions compared to the large intestine [70]. We observed histological evidence of an ongoing inflammatory process in animals fed the CON and NFDM diets. In contrast, we observed a reduction in epithelial cell nitrooxidative stress in BC-fed animals which may be associated with the greater population diversity that we observed in the ileal microbiome of these animals. The decreased gut inflammation-associated generation of NT proteins in BC-supplemented chickens is consistent with other known anti-inflammatory effects of colostrum such as the observed reduction in NFk-B-mediated proinflammatory cytokine expression in intestinal epithelial cells [71]. Those nitration reactions can play out in the cascade of responses initiated by such mediators as TNF-α cascading through increases in intracellular NO and superoxide anion culminating in the generation of nitrating reactants like ONOO^−^ and the more reactive ONOOCO_2_^−^, as produced when higher pCO_2_ is present in sections of the gut with more anaerobic status with perturbed mesenteric blood flow. The ileal microbiome also differed from that of the duodenal and colonic segments analyzed in that the segmented filamentous bacteria Candidatus Arthromitus was localized to the cell layer scraped from the ileal bowel (rather than the digesta per se). These data suggest that anatomical localization of microbiota composition in the gastrointestinal tract may be important in the regional status of immune activation in different gut segments as well as the inflammation-anti-inflammation status thus suggesting the need for spatially distinct analyses along the gastrointestinal tract.

We hypothesized that BC-initiated changes in the gut microbiota may enhance the metabolic processing of nutrients into anti-inflammatory metabolites, a feature we characterized with the development of the anti-inflammatory index, Ai-i. These observations were reproducible over time (i.e., replicated experiments) and across different sources of colostrum (cow breed and collection time after parturition). The ability of BC to beneficially modify the gut environment was due to the nutrient-derived metabolites and microbial populations in the ileum not matched by the NFDM diet. The group of metabolites identified after statistical regression alignment supported the hypothesis of an increased abundance of metabolites with anti-inflammatory character and aligned with observed effects on gut morphology and an improved efficiency of nutrient use for body weight gain in BC.

Several classes of compounds were altered by the BC supplementation. Compared to CON feed, BC feed increased the microbially-derived secondary bile acids, chenodeoxycholic acid, 7-ketolithocholic acid, in the ileum. Studies in humans and mice have shown bile acids to have an anti-inflammatory effect on immune cells [72] and appear to function in their host-microbe cross-talk signaling capacity through the farnesoid-X receptor (FXR) and the G-protein-coupled bile acid receptor-1 (TGR5) [73].

Oligosaccharides 3’-sialyllactose (3SL) and 6'-sialyllactose (6SL) with prebiotic properties were markedly increased in the ileal contents of chickens fed BC- and NFDM-supplemented feed compared to CON. The levels of these oligosaccharides were significantly higher in BC than marginally-increased levels in NFDM. However, they did not statistically contribute to the Ai-i per se, the positive correlation largely driven by the levels found in BC. Oligosaccharides in BC may facilitate microbiota remodeling in the chicken by reducing the ability of pathogens to gain a niche in the gut microenvironment and through promoting growth of beneficial microbiota as has been seen in humans and mice The increased oligosaccharides in BC associate strongly with the selective increase in ileum mucosa-associated segmented filamentous bacteria (SFB), namely Candidatus Arthromitus, in chickens in the BC-supplemented group. SFB were sparsely detected in the ileal mucosa of both CON or NFDM chickens. Furthermore, Candidatus Arthromitus was not detected in the raw colostrum after incubation on media for 48 h suggesting that some aspect of the BC diet promoted the increase in SFB already present in the ileum. These bacteria, living predominantly attached to the ileal epithelial mucosa, are known to promote gut maturation and gut mucosal and adaptive immune development including the development of germinal centers in Peyer’s patches that generate potent IgA and Th17 responses [74, 75]. By itself, SFB can induce intestinal T cell development similar to that induced by the complete gastrointestinal microbiota [76]. SFB colonization also has been shown to improve barrier protection against enteric viral infections and associated diarrheal diseases [77] as well as serve as a coordination link between metabolism and immune function [78]. Danzeisen et al. [79] demonstrated that in turkeys a fundamental determinate of growth success was the gut microbial milieu, particularly the presence of significantly more SFB.

Tryptophan, an essential amino acid, is metabolized by gut bacteria to generate metabolites specific to indole, kynurenine and serotonin pathways, the latter a key neurotransmitter in both the enteric and central nervous systems [65]. The tryptophan metabolite, indolelactic acid, is elevated in NFDM, but even more so in BC-supplemented diets. Indolelactate acts on gut epithelial cells, Paneth cells and enteroendocrine cells through specific receptors to (1) stimulate the release of antimicrobial peptides, (2) upregulate tight junction proteins to maintain barrier function; (3) suppress inflammatory cytokine production, (4) decrease superoxide anion generation, and (5) decrease apoptosis. Tryptophan metabolites generated by the microbiota can bind to the endogenous tryptophan receptor, the aryl hydrocarbon receptor (AHR) [64]. Indolelactate is generated only by bacteria, particularly *Lactobacillus,* abundant in BC, and bifidobacteria with demonstrated benefits to gut health in colostrum-fed infants [63, 80].

While the methods used by Metabolon Inc. do not allow us to measure short chain fatty acid (SCFAs), they undoubtedly were present in the BC that are high in indigestible carbohydrates (“prebiotics”)” that selectively enhance the growth of *Bifidobacterium* and *Lactobacillus* that have been shown to modulate inflammation and modulate a vast range of physiologic processes that include suppression of inflammatory signals and carcinogenesis [81]. The microbially-derived SCFAs share the use of receptors and signaling pathways that are used by the host gut epithelium to detect molecules that signal between the gut microbiota and the host.

Our findings suggest that BC feeding enhances the generation of several classes of anti-inflammatory metabolites. Collectively, the findings support the need to better understand not only what colostrum components other than traditionally assessed factors like immunoglobulins, antimicrobial factors, growth factors and cytokines contribute to an anti-inflammatory environment in changing the composition of the digesta in the gut lumen. Perhaps more critical though, information gaps exist needing resolution regarding what anti-inflammatory factors and metabolite precursors reach the different sections of the gut and are processed through host-diet-microbe interactions in maintaining gut health and homeostasis.

## Conclusion

The addition of BC to standard feed exerts profound effects on the immunometabolism of chickens, a model for gut health exquisitely sensitive to metabolic perturbations that reflects the impact of gut stress on feeding efficiency and growth [50]. Compared to chickens fed CON or a NFDM-supplemented diet, BC-fed chickens had an increased ileal absorptive surface and developed changes in the composition and structure of the gut microbiota that generated feed- and host-derived metabolites and compounds, resulting in an anti-inflammatory gut microenvironment. This could be attributed to significant increases in several BC-associated digesta metabolites with a defined anti-inflammatory character. Importantly, only the BC-fed chickens had major increases in mucosa-associated SFBs known to promote gut maturation and immune mucosal development. Our study also highlights the importance of sampling the mucosal surface in addition to the digesta to comprehensively assessing gut microbiota. In addition, the results underscore the utility in knowing how the total anti-inflammatory capacity established in the interaction between microbes and metabolites of a given gut region can be changed as a result of feeding a complex food matrix with the active components identified through appropriate statistical analysis. Future studies are required to determine if initiation of the BC supplement at an earlier time point may have an even greater effect, or to define the changes in the immune response provoked by the presence of the SFBs.

## Materials and methods

### Animal model

The rapidly growing commercial meat chicken (*Gallus gallus*, Strain: Ross 708, Amick Farms Hatchery, Hurlock, MD) was used for the nutrition, inflammation, metabolome, and microbiome objectives of the study. Genetic selection for characteristics of allometric growth result in an enhanced accretion of ingested nutrients in lean muscle deposition largely associated with breast and leg tissue. The rapidity of growth (< 49 days from hatch to commercial processing; [> 100 g body weight (BW) gain/day]) and high rate of nutrient utilization for lean tissue accretion (< 1.7 kg feed to attain 1 kg increase in BW) make these animals exquisitely sensitive to perturbations in environmental conditions, including those of diet [82, 83] and require stringent controls on environment and feed [84]. For these chickens, it is recognized that the growth process dynamics and feed efficiency as well as changes in the gut microbiome are highly volatile and change over time in as little as 1 week, essentially as the animals mature [85, 86]. With this awareness, we assessed what was consistently affected by the presented dietary treatments and what metabolite and gut microbiota attributes of the treatment diets might mitigate low level gut inflammation as specifically addressed in terms of nitrooxidative stress, i.e., the abundance of tyrosine nitrated proteins in the gut [26, 27].

### Experimental treatments: diets supplementation with colostrum extract or nonfat dry milk

A total of four colostrum samples (4 L each) were collected from multiparous Jersey (n = 2) or Holstein (n = 2) dairy cows as first and second milkings after parturition, respectively. In accordance with traditional feeding for cows at parturition and early lactation, cows were fed a total mixed ration consisting of corn and grass silages supplemented with roasted soy bean meal, vitamins and minerals. As such, the colostrum from the cows would be naturally enriched with a spectrum of flavones and isoflavones, etc. derived from the plant matrix of the diet [87, 88]. This concept aligns well with the data in Table 1 and Additional file 5: Table S1. Each collected colostrum was frozen at − 80 °C until needed for diet preparation. To prepare the colostrum-containing diet, the following procedures were performed. The thawed, cold colostrum from one cow at a time was thoroughly mixed, aliquoted into eight 50 ml polycarbonate tubes and centrifuged at 2 °C for 1 h at 20,000×*g*, centrifugation conditions sufficient to congeal lipids and sediment bacteria at the top and bottom of the tube, respectively. In agreement with Morrill et al. [89], the fat/lipid content of the different colostrums was not different as a function of cow breed or day obtained. After centrifugation, the solidified lipid (nonaqueous long chain fatty acid component) was removed from the top of the samples and the liquid underneath constituting the middle third of the tube volume poured off and collected. Higher density sedimented material in the bottom third of the centrifuge tubes containing cells, bacteria and cell and membrane debris was discarded. The fluid from each of the four cow’s colostrum was pooled across tubes but maintained as separate pools for the generation of the respective BC-supplemented diets. After the pooling, the material was passed through glass fiber matt filters. Collected material was sprayed onto a base diet previously used for chicken experiments by our laboratory [corn-soybean meal base; 23 crude protein and 13 MJ metabolizable energy [27]] to supply the equivalent of 100 ml colostrum/kg feed, with continuous mixing. The damp diet was put into 4 L glass high-vacuum containers, frozen at − 80 °C, lyophilized to dryness, and fed as such. To assess the effect of the addition of this quantity of colostrum to the overall crude protein and energy content of the base diet, a test batch of each of the BC diets was formulated and sent out for nutritional analysis (Cumberland Valley Analytical Services, Mercersburg, PA). Following analysis, it was observed that BC diets were approximately 2.7% higher in crude protein but otherwise (total fat, gross energy, fiber, etc.) not different from the base diet. To correct for the protein difference, the base diet to be used to prepare the BC was modified by lowering the soybean-derived protein content by 2.7% and that protein difference then made up for with the inclusion of the BC. When the colostrum component was added to this lower protein base diet, reanalysis of the diets showed them to be equivalent in protein. A third diet was developed, analyzed, and formulated to more closely approximate the amino acid composition of the BC diets and for this purpose nonfat dry milk (NFDM, Carnation, Inc.) was added to the lower crude protein base.

### Study design

All research was conducted under an animal management and welfare protocol approved by the USDA Beltsville Institutional Animal Care and Use Committee. One day old chicks were transported from the hatchery to the USDA Poultry Research Facility (Beltsville, MD) and placed in group brooder housing with the environmental temperature set according to the age of the animal. The CON feed and water were provided ad libitum. At 15 days of age, chickens were moved from the group pens to individual pens where the daily feed intake for each animal could be accurately measured. The abrupt change in local environment and housing as well as separation from a group is recognized as a short-term, low-level stress situation impacting the hypothalamic–pituitary–adrenal axis with impact on gastrointestinal function in the chicken [90, 91]. The quantity of fresh diet given to each chicken daily was recorded. The next morning the residual feed not eaten from the previous day was measured and the difference between given and residual used as the measure of feed intake. Live body weight of each chicken was obtained daily at a standardized time of day relative to feed management. Chickens and their environment were observed three times daily as regards the health and welfare of the animals per approved animal care protocol.

The overall experiment was conducted in 2 separate replicates of the following treatments: control diet (CON), nonfat dry milk diet (NFDM), and colostrum diets (BC), the replications conducted 6 months apart. Colostrum was obtained from four different sources; sources consisted of Jersey cow first postpartum milking, Jersey cow second postpartum milking, Holstein cow first postpartum milking, and Holstein cow second postpartum milking. Variability in colostrum composition (day1 and day 2 of lactation postpartum and Jersey and Holstein breeds) was intentionally built into the design so that across the variability a set of metabolites with consistent effects on inflammation and gut parameters could be ascertained. In preparing the diets for the first replicate of the study, one BC diet was prepared using Jersey-derived first milking colostrum and the other BC diet prepared from the Holstein-derived first milking. Similarly, in the second replication the two BC diets were made from the Jersey and Holstein colostrum second milkings.

Transfer of the chickens to the individual pens and the respective switches to the test diets were considered the first day of the start of the experiment. Test diets were fed for 7 days with the animals euthanized (per American Veterinary Medical Association Guidelines for Euthanasia of Animals: 2013 Edition, https://www.avma.org/KB/Policies/Documents/euthanasia.pdf) on day 8 following the recording of the final weight. As pooled across the two experimental replicates, there were 11 CON diet animals, 11 NFDM diet animals and a total of 22 animals fed colostrum-containing diets, the data from which animals was used for the metabolomic and microbiome analysis. Variation in the n across the various diet treatment groups reflects further refinement based on the quality of data primarily obtained from the metabolomic evaluations. When an animal’s metabolite data were identified as an outlier (> 3 S.D.) or compromised as a result of an inefficient extraction, that animal was eliminated from the study. The numbers of animals per treatment group from which the data were amassed exceeded the minimum number for achieving statistical significance based on statistical power analysis (Introduction to Power and Sample Size Analysis., https://support.sas.com/documentation/onlinedoc/stat/131/intropss.pdf). Samples prepared for immunofluorescence were obtained from representative numbers of animals in each replication of the experiment. Following euthanasia, the intestinal tract was rapidly excised and divided into segment samples for duodenum (central loop), distal ileum, and cecum (with the present work focusing on the ileum). These samples were further subdivided into 2 components. Each segment was opened longitudinally. From one the digesta was carefully removed to eliminate intestinal tissue potentially scraped from the wall. The other section was rinsed free of remaining digesta and debris with ice cold PBS and the epithelial surface gently scraped away using a fresh, chilled glass slide. One additional section of rinsed intact gut was immersed overnight in 4% paraformaldehyde and transferred to ethanol for further paraffin embedding and sectioning for microscopy as subsequently described. To evaluate the live growth of the chickens we measured the average daily body weight gain and feed efficiency (the grams of ingested feed corresponding to the grams of weight gained per day).

### Fluorescence microscopy and quantitative imaging analyses

The detailed methods for the immunofluorescent tissue antigen immunostaining as well as the exact process used to quantify the cellular pixel content of the 3’nitrotyrosine antigen used to represent the intensity of nitrooxidative stress can be found in the online Supplementary Information—Detailed Methods. The protocols for tissue fixation, fluorescence immunostaining for 3′-tyrosine-nitrated proteins (NT) as the inflammation marker and morphometric analysis of ileal samples published by our laboratory previously were applied in the present study [27, 34]. In brief, representative tissue sections for immunofluorescence were collected from the first and second replicates of the experiment (CON, n = 6, 3/replicate; NFDM, n = 6, 3/replicate; BC, N = 10, 5/replicate, randomly selected within treatment). Dual color immunofluorescence labeling of the target antigen and nuclei was performed to localize and subsequently quantify epithelial cell inflammation with NT proteins (red pseudocolored immunofluorescence) as the target antigen along with the respective cell nuclei blue pseudocolored immunofluorescence). Following standard deparaffinizing, rehydration, and blocking of nonspecific antibody binding, slides were incubated overnight at 4 °C in a humidified chamber with anti-3′-nitrotyrosine (rabbit polyclonal, Millepore-Sigma, Burlington, MA, Inc., 1:100) with subsequent antigen visualization [goat anti-rabbit IgG Alexa 680 (1:400; 1 h, Thermofisher Scientific, Grand Island, NY)]. For validation purposes, an additional set of slides of the adjacent serial sections were prepared and immunostained to identify villi apical epithelial cells using anti-cytokeratin-18 (Abcam Inc., Cambridge, MA, mouse monoclonal, Clone-C04, 1:200) with antigen visualization (goat anti-mouse IgG Alexa 488, 1:400, Thermofisher Scientific, Grand Island, NY; green pseudocolored immunofluorescence). Nuclei were stained with 4–6-diamino-2-phenylindol-dihydrochloride (DAPI, Thermofisher Scientific). Morphometric measurements to determine the length relationships between the villi and crypts were performed using the DAPI blue channel using a calibrated and validated digital micrometer internal to the ImagePro 9.3^®^ software (Media Cybernetics, Rockville, MD). Quantitative image analysis to ascertain the pixel density of the NT antigen per villus epithelial cell was performed using ImagePro 9.3^®^ software (Media Cybernetics, Rockville, MD) as described in the Supplementary Information—Detailed Methods.

### Metabolome profiling and analyses

Samples of digesta were collected from each of the animals in both replicates of the study (CON, n = 10; NFDM, n = 10; BC, n = 20, with 4 samples failing to be extracted efficiently and the results excluded) from a region of the ileum 3–5 cm cephalad to the ileocecal junction. For the metabolomic profiling of the digesta, a homogenous sample (approximately 100 mg) was accurately weighed to the 0.1 mg into a polypropylene vial, with the vial capped and frozen in liquid nitrogen. Metabolite identification and quantification was performed under contract by Metabolon, Inc., Research Triangle Park, NC, and all steps for this analysis were conducted according to the company’s protocols (Supplementary Information—Detailed Methods). In brief, the basic process was as follows**:** Metabolon’s first preparation step was to lyophilize the weighed samples and then reconstitute the samples for analysis by ultrahigh performance liquid chromatography-tandem mass spectroscopy (UPLC-MS/MS). In the lyophilization step, the low molecular weight volatile short chain fatty acids (SCFA), acetate, propionate, and butyrate, were differentially sublimated off the samples and not available for analysis. Raw data was extracted, peaks identified, and QC processed using Metabolon’s hardware and software. Data were made available as Excel^®^ files containing values for each identified compound representing an area-under the curve which were normalized, per Metabolon, Inc., in terms of raw area counts and presented as the “Original Scale” (Additional file 9: Data S2). For a single day run, this was equivalent to the raw data. For comparing diet treatments, each metabolite’s value in Original Scale was mathematically processed to set the median for that metabolite equal to 1 and each animal’s value for that metabolite proportionately scaled accordingly. These scaled values were referred to “Scaled Imputed Value” (SIV). The preliminary statistical ANOVA analysis of the data was performed by Metabolon, Inc. with comparisons and separated effects established using contrast statements such that an overall understanding of the sources of variation as affected by diet, colostrum source, and replication could be assessed.

### “Anti-inflammatory index” of the metabolome

A major objective of the study was to define a set of mass spectrometry-identified metabolites in the ileal digesta that could facilitate a reduction in epithelial cell inflammation and reflect the metabolite profiles associated with the fed BC or NFDM diets. To accomplish this, we developed the term “Anti-inflammatory-index” (Ai-i) to serve as an integrative parameter intended to reflect the selection and grouping of a set of specific metabolites that contributed significantly to relieving the magnitude of nitration stress present in epithelial cells of the ileum. A detailed description of the A-i-i and rationale for development is further presented in accompanying on-line Supplementary Information—Detailed Methods. Briefly, the A-i-i was derived from summing the Scaled Imputed Values for the metabolites that (a) were defined in the literature (Additional file 10: References 1) as having anti-inflammatory character and not simply antioxidative function, (b) anti-inflammatory metabolites that were significantly greater in abundance in one diet compared (ANOVA; Proc GLM, SAS^®^ ver. 9.4: https://documentation.sas.com/doc/en/pgmsascdc/9.4_3.3/statug/statug_glm_syntax01.htm) to the other diets, and (c) repeatably different between diets across the two experiment replications and the four kinds of colostrum or the NFDM used in the diet replications. To be included in the final multiple regression model set, a given metabolite when added to the regression model needed to promote an improvement (increase) in the adjusted R^2^ of the model as tested for either a linear or exponential fit.

### Microbial community analyses

The assessment of the bacterial milieu of the ileum was made on individual samples collected from the ileum at a point immediately cephalad to the section obtained for metabolite profiling. In addition to digesta samples, one additional sample of the digesta-free epithelial layer of each specimen was carefully scraped from the tissue segments to address what would be considered microbes attached to the epithelial cells. After a bead-beating step on a TissueLyzer II (Qiagen Inc., Germantown, MD), DNA was extracted from all samples (150 mg tissue wet weight) using the MagAttract PowerMicrobiome DNA/RNA kit (Qiagen Inc.) implemented on a Hamilton STAR robotic platform (Hamilton, Reno, NV). Amplification of the 16S rRNA gene V4 hypervariable region was performed using dual-barcoded universal primers 515F and 806R as previously described [92]. High-throughput sequencing of the amplicons was performed on an Illumina MiSeq instrument using the 300 bp paired-end protocol. Raw data was demultiplexed as cited [93, 94]. Barcode, adapter, and primer sequences were trimmed using TagCleaner (2013-10-14 vers; [95]). Quality assessment and sequencing error correction was performed using the software package DADA2 (version 1.14; [96]) and the following parameters: forward reads were truncated at position 220 and the reverse reads at position 160 based on the sequencing quality plot, no ambiguous bases and a maximum of 2 expected errors per-read were allowed [97]. The quality-trimmed reads were used to infer ribosomal sequence variants and their relative abundance in each sample after removing chimera. Sequencing analyses includes denoising, de novo and reference-based chimera detection conducted with UCHIME v5.1 [98]. Taxonomic ranks were assigned to each sequence using the Ribosomal Database Project [[99] Naïve Bayesian Classifier v.2.2 [100] trained on the Greengene database (Aug 2013 version) [101]], using 0.8 confidence values as cutoff. The heatmap and bar plot were generated using statistical package R (v3.2.1) and Phyloseq packages [102]. Clustering of taxonomic composition and abundance in a sample were performed using Ward linkage hierarchical clustering based on Jensen-Shannon divergence metrics. Jensen-Shannon divergence is a measurement of dissimilarity between probability distribution, and Jensen-Shannon metric is the square root of the normalized Jensen-Shannon divergence value [103, 104]. The resulting clusters were used to define microbiota type, which indicates the clustering of similar community compositional profile that is a vector of the percentage of the sequences assigned to a phylotype in each sample. Linear Discriminant Analysis (LDA) effect size (LEfSe) algorithm was adapted to quantitatively characterize the phylotypes that could explain the differences observed between two biological conditions [105]. It identifies phylotypes in their relative abundance profiles through building an LDA model to estimate the “effect size” of each phylotype with respect to biological conditions under inspections. The alpha value for the non-parametric factorial Kruskal–Wallis (KW) sum-rank test [106] is 0.05, the threshold on the logarithmic LDA model [107] score for discriminative features is 2.0. We used all-against-all BLAST search in multi-class analysis that is stricter than the one-against-all search, and we denote both target classes and subject names in the analysis.

In order to ascertain if any contribution to the microbiota in chickens fed BC might have originated in the colostrum itself, especially that of the SFB, samples of the BC matrix as applied to the feed was analyzed. The metagenomic approach was chosen to assess the microbiota of the colostrum. Given the low biomass in colostrum, applying the PCR-based 16S rRNA on such a specimen type may have introduced additional biases. To access the microbiota of the colostrum, the non-processed colostrum was filtered through the sterile 0.22um syringe filter (Sterivex, Germany), and the materials left on the filter was subjected to 45 s beating cycle using a FastPrep instrument (MP Biomedicals) at 5.5 m/s and centrifuged at 14,000 rpm for 1 h at 4 °C. The supernatant after beads beating was used for DNA extraction using the same procedure as above 16S rRNA samples. Metagenomic sequencing libraries were constructed from the same DNA using the Nextera XT Flex kit (Illumina, San Diego, CA) according to the manufacturer’s recommendations. Libraries were then pooled together in equimolar proportions and sequenced on Illumina HiSeq 4000 platform using the 150 bp protocol at the Genomic Resource Center at the University of Maryland School of Medicine. Metagenomic sequence reads were removed using BMTagger v3.101 [108] using a Genome Reference Consortium *Gallus gallus* Build 5.0 (GCA_000002315.3, [109]). Sequence read pairs were removed even if only one of the reads matched to the genome reference. Taxonomic profiling was conducted in MetaPhlAn vers 2 [110]. We specifically performed reads mapping of the metagenomic reads to published genomes of Candidatus Arthromitus isolated from chicken, turkey, mouse, and rat (NZ_CP008713.1, NC_015913.1, NZ_AGAG01000005.1, NZ_AGVP01000010.1, NC_017294.1, NC_016012.1, LXFF01000001.1) using bowtie [v1, parameters: “-l 25-fullref -chunkmbs 512 -best -strata -m 20”, [111]].

### Statistical analyses

The basic statistical analysis for the effects of diet treatment on growth, feed efficiency, morphometric measurements, NT pixel content, and the assessment of specific metabolites on an anti-inflammatory index was analysis of variance performed using the General Linear Models procedure of the Statistical Analysis System^®^ ver. 9.4 [112]. Specific contrast statements [113] were used to test hypotheses regarding the effects of the various diets as well as diet combinations on the dependent variables. “Animal-within-treatment” was used as the error term.

For microbial population inferences, in order to estimate differences in relative abundances of different bacterial clusters within different diet status, a Bayesian Poisson model was employed and the structure is as follows:

$$y_{i} \sim Poisson\left( {\lambda_{i} } \right)$$

$$\log \left( {\lambda_{i} } \right) = a + b_{diet\left( i \right)} + c_{cluster\left( i \right)} + d_{diet\left( i \right),cluster\left( i \right)}$$

where $y_{i}$ is the count in the i-th cell and *diet(i), cluster(i)* are the diet and cluster of the i-th cell, respectively. The model was fitted using JAGS R package [114], and 100,000 iterations with the same number of burn-in iterations was used. The convergence of the model was assessed using Gelman and Rubin’s potential scale reduction factor [115] and visual inspection of each coefficient’s Markov chains.

## Supplementary Information

**Additional file 1**.** Figure S1**: Within-sample diversity by community type. Notched boxplot of diversity index of samples grouped in community type (color indicated in legend). Within-sample diversity was estimated using Shannon diversity index using Phyloseq R package [102]. The top and bottom of the box are the lower and upper quartiles, and the band near the middle of the box represents the median. Box width is proportional to the square root of the size of the category.

**Additional file 2**.** Figure S2**: Statistical analyses of relative frequency of community types. The mean relative frequencies and their 95% credible intervals are shown. A Bayesian Poisson model was employed, and model fitting was performed using JAGS R package [114] and 100,000 iterations with the same number of burn in iterations. Abbr: DUO: duodenum; CEC: cecum; ILE: ileum.

**Additional file 3**.** Figure S3**: Community diversity analyses using weighted UniFrac principal coordinates analysis (PCoA) analyses using QIIME2 (v2019.10) [117]. Each symbol represents a sample colored by its different anatomical sites. The scatterplot is of principal coordinate 1 (PC1) plotting against principal coordinate 2 (PC2), and the percentage of the variation described by the plotted principal coordinates in indicated on the axes.

**Additional file 4**.** Figure S4**: Relative abundance of phylotype biomarkers. Analyses were performed using program LEfSe [105]. Bars represent the relative abundance of a phylotype in each sample. Dotted line represents mean, solid line represents median relative abundance. The alpha value for the non-parametric factorial Kruskal-Wallis (KW) sum-rank test was 0.05 and the threshold for the logarithmic LDA model ([107]) score for discriminative features was set at 2.0. Abbr: DUO: duodenum; CEC: cecum; ILE: ileum.

**Additional file 5**.** Table S1**: Summary of the identified plant-derived xenobiotic compounds in digests of the duodenum, ileum and cecum. Data illustrate the nature of the changes in presence/detention and relative levels of xenobiotic plant-derived compounds in the relevant digesta as affected by diet. Digesta from chickens fed BC was more complex with more compounds present in the ileal digesta.

**Additional file 6**.** Table S2**: Taxonomic profiles of the intraluminal stool specimens collected in this study. Intraluminal stool specimens collected from A) different anatomical sites of ileum, duodenum and cecum for a total of 156 samples; B) only ileum. The taxonomic composition of the microbiomes was established using 16S rRNA gene V4 hypervariable region and dual-barcoded universal primers 515F and 806R as previously described [92].

**Additional file 7**.** Table S3**: Taxonomic profiles of the colostrum specimens. Two non-processed colostrum was included in this study. The taxonomic composition of the microbiomes was established using MetaPhlAn version 2 [110].

**Additional file 8**.** Supplementary Data 1**. Metabolomics.

**Additional file 9**.** Supplementary Data 2**. Metabolon Analytic Platform.

**Additional file 10**. Reference database obtained using a Boolean construct of the terms: polyphenol, flavonoids, bile acids, amino acids, fatty acids, or oligosaccharides along with small intestine, metabolite, and inflammation. Other relevant references have already been incorporated into the main reference information.

## Data Availability

The sequences of 16S rRNA and metagenomes were submitted to GenBank under BioProject PRJNA821505 (https://www.ncbi.nlm.nih.gov/bioproject/PRJNA821505). Supplemental tables are shared on figshare, download link is https://figshare.com/articles/dataset/_/19469372 and https://figshare.com/articles/dataset//19469378
