# Supplementary figures and images for: Short-term feeding of defatted bovine colostrum mitigates inflammation in the gut via changes in metabolites and microbiota in a chicken animal model
Source: Anim Microbiome. 2023 Jan 26;5:6. doi: 10.1186/s42523-023-00225-z (PMC9878500; doi:10.1186/s42523-023-00225-z)

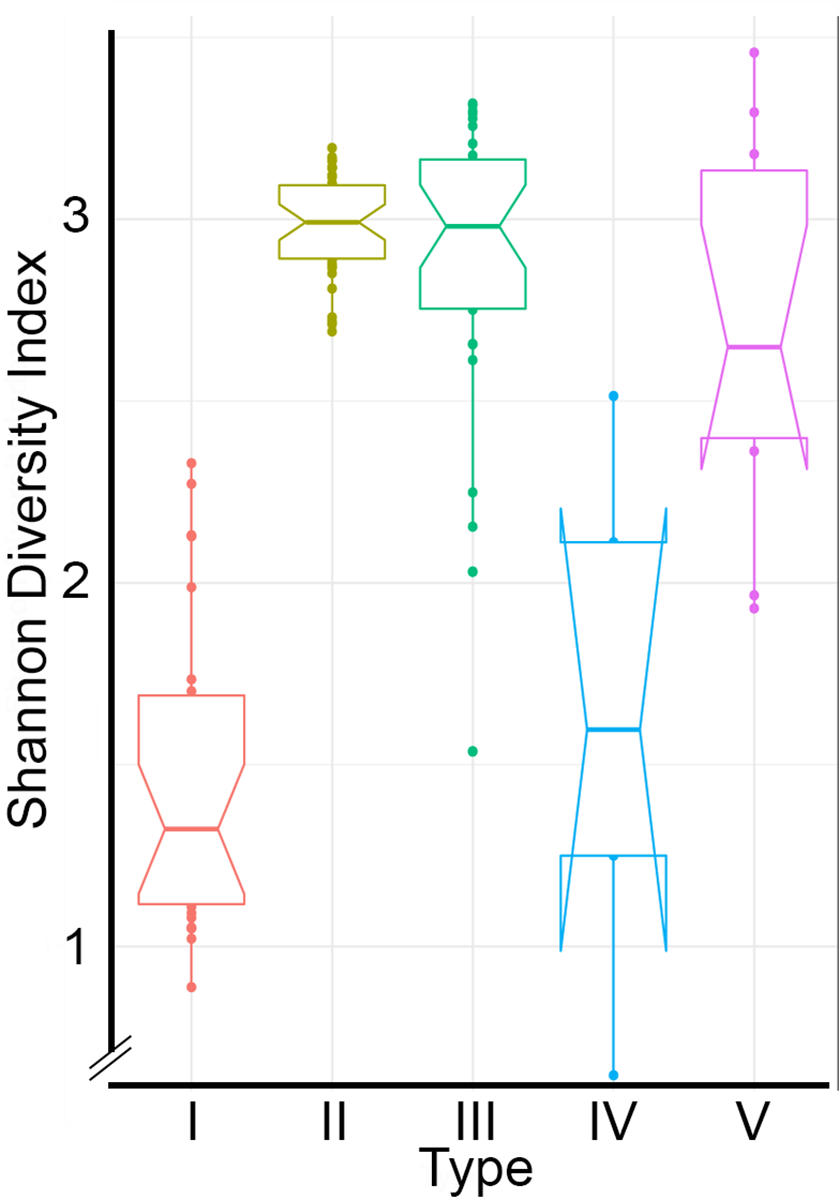

Supplement: Supplementary file 1 — Additional file 1. Figure S1: Within-sample diversity by community type. Notched boxplot of diversity index of samples grouped in community type (color indicated in legend). Within-sample diversity was estimated using Shannon diversity index using Phyloseq R package [102]. The top and bottom of the box are the lower and upper quartiles, and the band near the middle of the box represents the median. Box width is proportional to the square root of the size of the category. [file 42523_2023_225_MOESM1_ESM.tif]

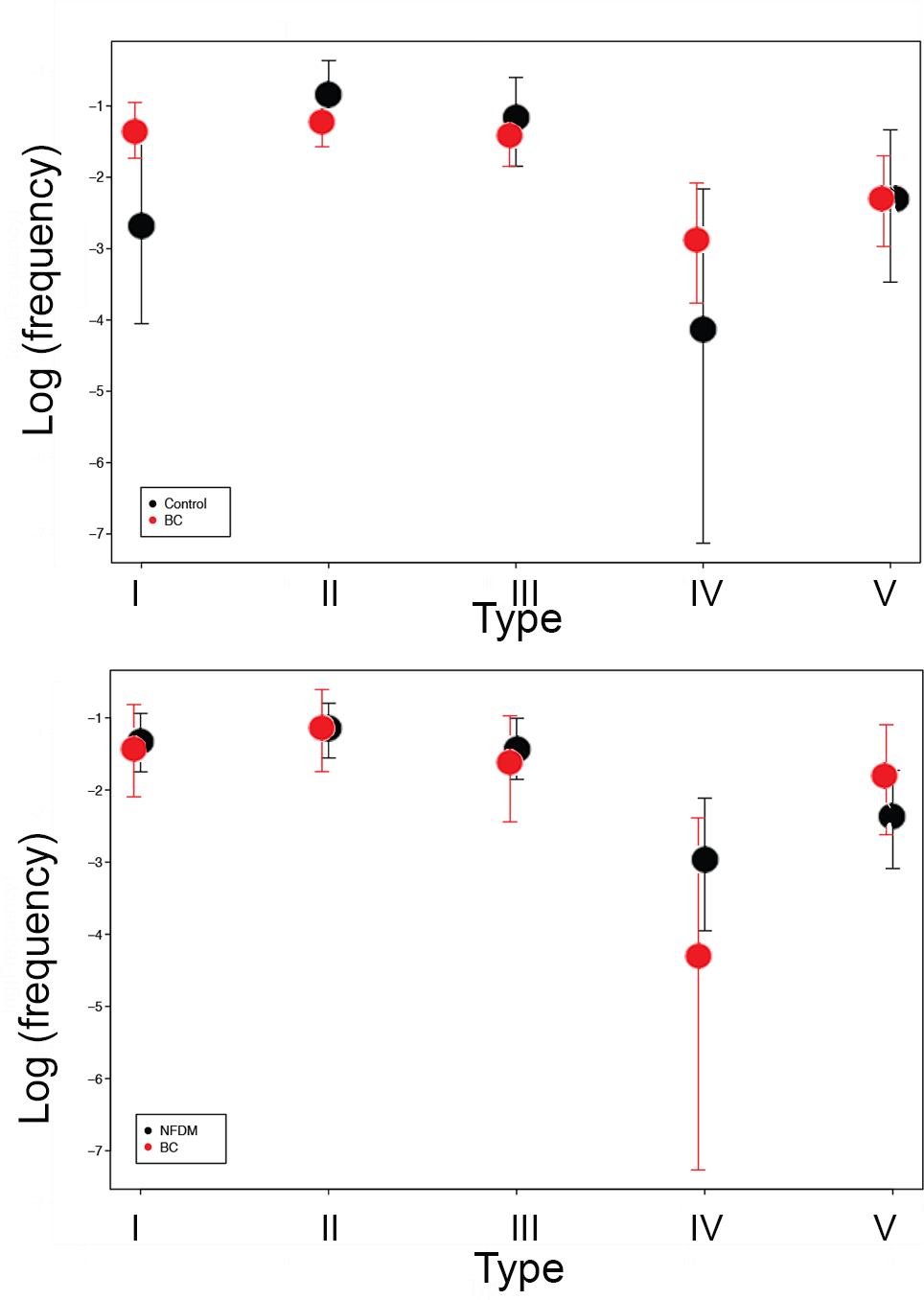

Supplement: Supplementary file 2 — Additional file 2. Figure S2: Statistical analyses of relative frequency of community types. The mean relative frequencies and their 95% credible intervals are shown. A Bayesian Poisson model was employed, and model fitting was performed using JAGS R package [114] and 100,000 iterations with the same number of burn in iterations. Abbr: DUO: duodenum; CEC: cecum; ILE: ileum. [file 42523_2023_225_MOESM2_ESM.tif]

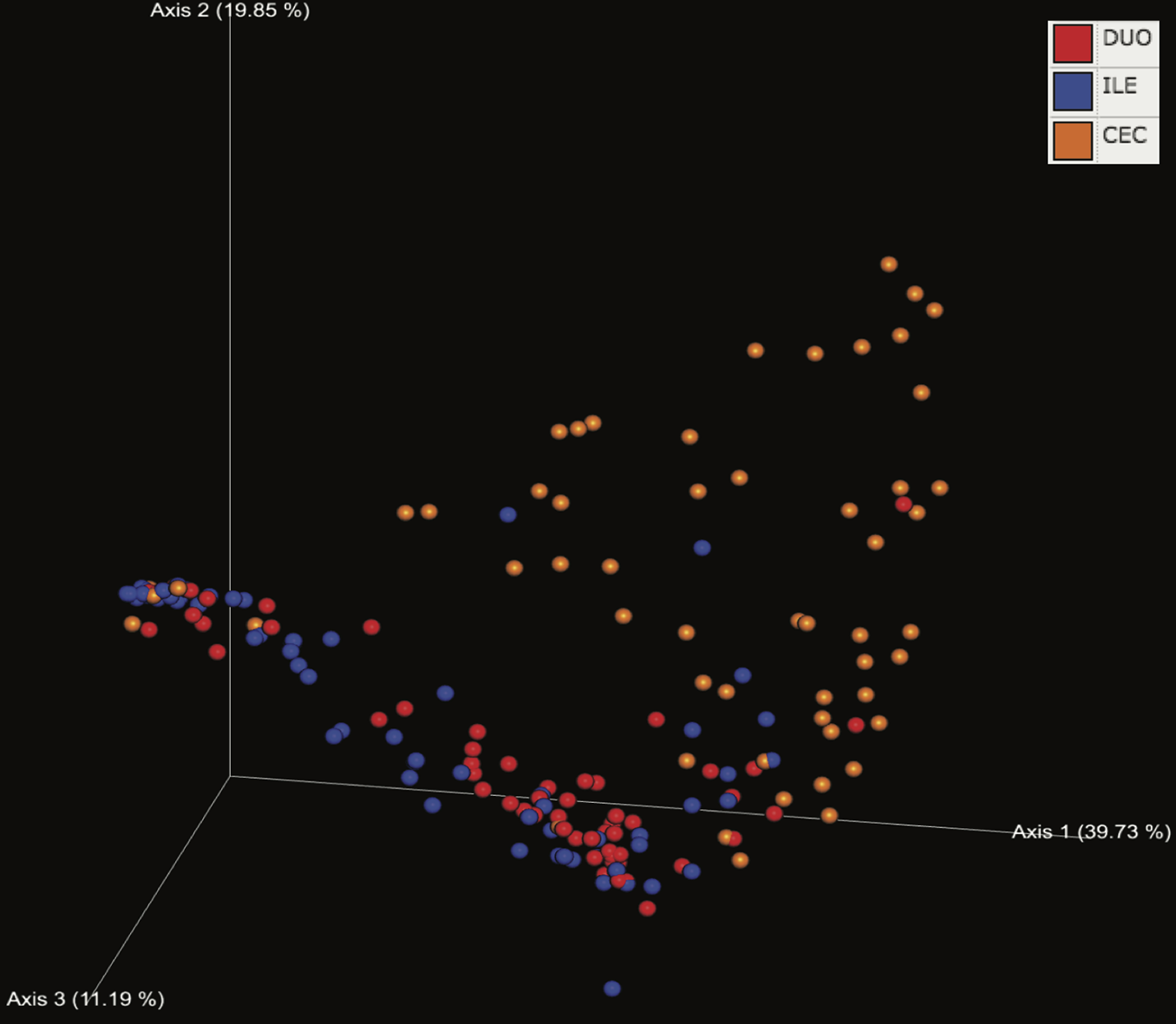

Supplement: Supplementary file 3 — Additional file 3. Figure S3: Community diversity analyses using weighted UniFrac principal coordinates analysis (PCoA) analyses using QIIME2 (v2019.10) [117]. Each symbol represents a sample colored by its different anatomical sites. The scatterplot is of principal coordinate 1 (PC1) plotting against principal coordinate 2 (PC2), and the percentage of the variation described by the plotted principal coordinates in indicated on the axes. [file 42523_2023_225_MOESM3_ESM.tif]

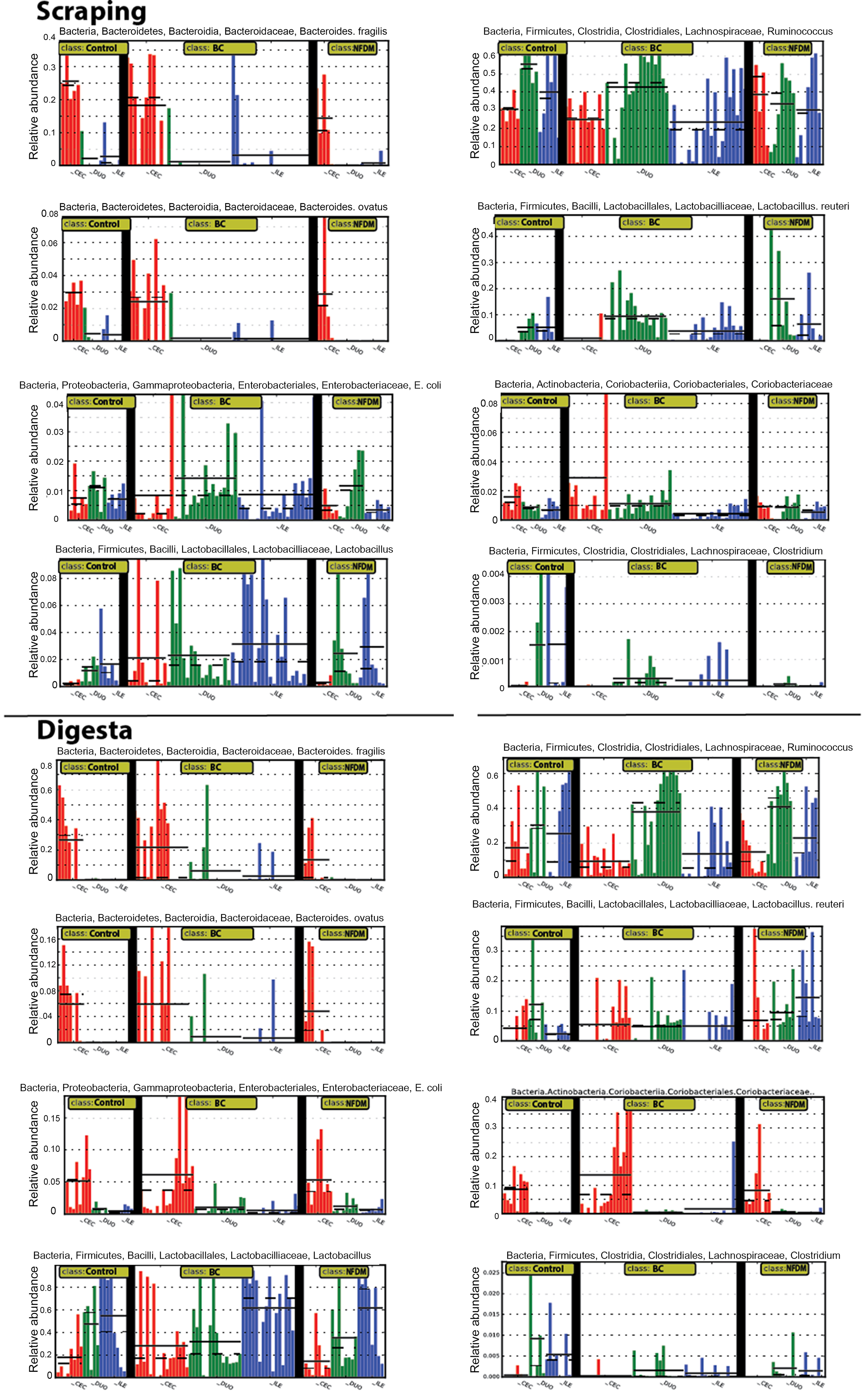

Supplement: Supplementary file 4 — Additional file 4. Figure S4: Relative abundance of phylotype biomarkers. Analyses were performed using program LEfSe [105]. Bars represent the relative abundance of a phylotype in each sample. Dotted line represents mean, solid line represents median relative abundance. The alpha value for the non-parametric factorial Kruskal-Wallis (KW) sum-rank test was 0.05 and the threshold for the logarithmic LDA model ([107]) score for discriminative features was set at 2.0. Abbr: DUO: duodenum; CEC: cecum; ILE: ileum. [file 42523_2023_225_MOESM4_ESM.tif]
