# Supplementary material for: Short-term feeding of defatted bovine colostrum mitigates inflammation in the gut via changes in metabolites and microbiota in a chicken animal model
Source: Anim Microbiome. 2023 Jan 26;5:6. doi: 10.1186/s42523-023-00225-z (PMC9878500; doi:10.1186/s42523-023-00225-z)
Supplement: Supplementary file 10 — Additional file 10. Reference database obtained using a Boolean construct of the terms: polyphenol, flavonoids, bile acids, amino acids, fatty acids, or oligosaccharides along with small intestine, metabolite, and inflammation. Other relevant references have already been incorporated into the main reference information. [file 42523_2023_225_MOESM10_ESM.docx]

**Supplementary References 1**

Output from the Boolean keyword combination search: polyphenol, flavonoids, bile acids, amino acids, fatty acids, mitochondrial energy metabolites, tocopherols, or oligosaccharides along with small intestine, metabolite, inflammation, and anti-inflammatory

1. Atanasova BD, Li AC, Bjarnason I, Tzatchev KN, Simpson RJ. Duodenal ascorbate and ferric reductase in human iron deficiency. *Am J Clin Nutr* **81**, 130-133 (2005).

2. Athanasiadis S. [Restorative proctocolectomy with an ileal pouch. Functional and biochemical results]. *Langenbecks Arch Chir* **360**, 207-220 (1983).

3. Atwood L, James C, Morris GP, Vanner S. Cellular pathways of mast cell- and capsaicin-sensitive nerve-evoked ileal submucosal arteriolar dilations. *Am J Physiol* **275**, G1063-1072 (1998).

4. Au Yeung KJ*, et al.* Impact of vitamin E or selenium deficiency on nematode-induced alterations in murine intestinal function. *Exp Parasitol* **109**, 201-208 (2005).

5. Aube AC, Cherbut C, Barbier M, Xing JH, Roze C, Galmiche JP. Altered myoelectrical activity in noninflamed ileum of rats with colitis induced by trinitrobenzene sulphonic acid. *Neurogastroenterol Motil* **11**, 55-62 (1999).

6. Auchère D, Tardivel S, Gounelle JC, Lacour B. [Stimulation of ileal transport of calcium by sorbitol in in situ perfused loop in rats]. *Gastroenterol Clin Biol* **21**, 960-966 (1997).

7. Autore G, Capasso F, Mascolo N. Phenolphthalein stimulates the formation of histamine, 5-hydroxytryptamine and prostaglandin-like material by rat jejunum, ileum and colon. *Br J Pharmacol* **81**, 347-349 (1984).

8. Awada M*, et al.* Dietary oxidized n-3 PUFA induce oxidative stress and inflammation: role of intestinal absorption of 4-HHE and reactivity in intestinal cells. *J Lipid Res* **53**, 2069-2080 (2012).

9. Ayajiki K, Okamura T, Fujioka H, Nakayama K, Tsuji K, Toda N. Functional studies on blockade by neosurugatoxin of nicotinic receptors in nitroxidergic and sensory nerve terminals and intramural ganglionic cells. *Jpn J Pharmacol* **78**, 217-223 (1998).

10. Azevedo MF, Lima CF, Fernandes-Ferreira M, Almeida MJ, Wilson JM, Pereira-Wilson C. Rosmarinic acid, major phenolic constituent of Greek sage herbal tea, modulates rat intestinal SGLT1 levels with effects on blood glucose. *Mol Nutr Food Res* **55 Suppl 1**, S15-25 (2011).

11. Baccari MC, Nistri S, Vannucchi MG, Calamai F, Bani D. Reversal by relaxin of altered ileal spontaneous contractions in dystrophic (mdx) mice through a nitric oxide-mediated mechanism. *Am J Physiol Regul Integr Comp Physiol* **293**, R662-668 (2007).

12. Bäckström T, Liska J, Oldner A, Lockowandt U, Franco-Cereceda A. Splanchnic metabolism during gut ischemia and short-term endotoxin and hemorrhagic shock as evaluated by intravasal microdialysis. *Shock* **21**, 572-578 (2004).

13. Badary OA, Awad AS, Sherief MA, Hamada FM. In vitro and in vivo effects of ferulic acid on gastrointestinal motility: inhibition of cisplatin-induced delay in gastric emptying in rats. *World J Gastroenterol* **12**, 5363-5367 (2006).

14. Bae EH*, et al.* Farnesoid X receptor ligand prevents cisplatin-induced kidney injury by enhancing small heterodimer partner. *PLoS One* **9**, e86553 (2014).

15. Bagcivan I*, et al.* Investigation of relaxant effects of propofol on sheep sphincter of Oddi. *Pancreatology* **7**, 174-179 (2007).

16. Bagnenko SF, Sinenchenko GI, Kurygin AA, Chupris VG. [Correction of a reperfusion dysfunction in acute intestinal obstruction]. *Vestn Khir Im I I Grek* **167**, 32-35 (2008).

17. Bahia SS, McMahon RF, Hobbiss J, Taylor TV, Stoddart RW. Pelvic ileo-anal reservoirs: a lectin histochemical study. *Histochem J* **25**, 392-400 (1993).

18. Bains Y, Gugliucci A, Caccavello R. Advanced glycation endproducts form during ovalbumin digestion in the presence of fructose: Inhibition by chlorogenic acid. *Fitoterapia* **120**, 1-5 (2017).

19. Baker RA, Saccone GT, Brookes SJ, Toouli J. Nitric oxide mediates nonadrenergic, noncholinergic neural relaxation in the Australian possum. *Gastroenterology* **105**, 1746-1753 (1993).

20. Baláz P*, et al.* Preservation injury of jejunal grafts and its modulation by custodiol and university of wisconsin perfusion solutions in wistar rats. *Eur Surg Res* **36**, 192-197 (2004).

21. Ballabeni V, Barocelli E, Bertoni S, Impicciatore M. Alterations of intestinal motor responsiveness in a model of mild mesenteric ischemia/reperfusion in rats. *Life Sci* **71**, 2025-2035 (2002).

22. Ballal MA, Sanford PA. The physiology of the biliary tree. Motility of the gallbladder--part 1. *Saudi J Gastroenterol* **5**, 93-105 (1999).

23. Balogh N, Krausz F, Lévai P, Ribiczeyné PS, Vajdovich P, Gaál T. Effect of deferoxamine and L-arginine treatment on lipid peroxidation in an intestinal ischaemia-reperfusion model in rats. *Acta Vet Hung* **50**, 343-356 (2002).

24. Balsiger BM*, et al.* Nitric oxide pathways in circular muscle of the rat jejunum before and after small bowel transplantation. *J Gastrointest Surg* **4**, 86-92 (2000).

25. Balvers MG*, et al.* Time-dependent effect of in vivo inflammation on eicosanoid and endocannabinoid levels in plasma, liver, ileum and adipose tissue in C57BL/6 mice fed a fish-oil diet. *Int Immunopharmacol* **13**, 204-214 (2012).

26. Bambouskova M*, et al.* Electrophilic properties of itaconate and derivatives regulate the IkappaBzeta-ATF3 inflammatory axis. *Nature* **556**, 501-504 (2018).

27. Bani D*, et al.* Relaxin depresses small bowel motility through a nitric oxide-mediated mechanism. Studies in mice. *Biol Reprod* **66**, 778-784 (2002).

28. Banning A*, et al.* GPx2 counteracts PGE2 production by dampening COX-2 and mPGES-1 expression in human colon cancer cells. *Antioxid Redox Signal* **10**, 1491-1500 (2008).

29. Barajas-Espinosa A, Ochoa-Cortes F, Moos MP, Ramirez FD, Vanner SJ, Funk CD. Characterization of the cysteinyl leukotriene 2 receptor in novel expression sites of the gastrointestinal tract. *Am J Pathol* **178**, 2682-2689 (2011).

30. Barchetta I*, et al.* Neurotensin Is a Lipid-Induced Gastrointestinal Peptide Associated with Visceral Adipose Tissue Inflammation in Obesity. *Nutrients* **10**, (2018).

31. Bardowell SA, Ding X, Parker RS. Disruption of P450-mediated vitamin E hydroxylase activities alters vitamin E status in tocopherol supplemented mice and reveals extra-hepatic vitamin E metabolism. *J Lipid Res* **53**, 2667-2676 (2012).

32. Bare LN, Wiseman RF. DELAYED APPEARANCE OF LACTOBACILLI IN THE INTESTINES OF CHICKS REARED IN A "NEW" ENVIRONMENT. *Appl Microbiol* **12**, 457-459 (1964).

33. Barnard JA, Ghishan FK. Methylprednisolone accelerates the ontogeny of sodium-taurocholate cotransport in rat ileal brush border membranes. *J Lab Clin Med* **108**, 549-555 (1986).

34. Barnes CJ, Lee M. Chemoprevention of spontaneous intestinal adenomas in the adenomatous polyposis coli Min mouse model with aspirin. *Gastroenterology* **114**, 873-877 (1998).

35. Barrett TA, Musch MW, Chang EB. Chemotactic peptide effects on intestinal electrolyte transport. *Am J Physiol* **259**, G947-954 (1990).

36. Bartho L, Holzer P. The inhibitory modulation of guinea-pig intestinal peristalsis caused by capsaicin involves calcitonin gene-related peptide and nitric oxide. *Naunyn Schmiedebergs Arch Pharmacol* **353**, 102-109 (1995).

37. Bartik L*, et al.* Curcumin: a novel nutritionally derived ligand of the vitamin D receptor with implications for colon cancer chemoprevention. *J Nutr Biochem* **21**, 1153-1161 (2010).

38. Barua AB. Absorption and conversion of a single oral dose of beta-carotene in corn oil to vitamin A in Sprague-Dawley rats with low reserve of vitamin A. *Int J Vitam Nutr Res* **73**, 267-273 (2003).

39. Barua AB, Olson JA. beta-carotene is converted primarily to retinoids in rats in vivo. *J Nutr* **130**, 1996-2001 (2000).

40. Basu NK, Kole L, Kubota S, Owens IS. Human UDP-glucuronosyltransferases show atypical metabolism of mycophenolic acid and inhibition by curcumin. *Drug Metab Dispos* **32**, 768-773 (2004).

41. Batist G*, et al.* Enzymatic defense against radiation damage in mice. Effect of selenium and vitamin E depletion. *Biochem Pharmacol* **35**, 601-606 (1986).

42. Batt RM, Peters TJ. Effects of prednisolone on the small intestinal mucosa of the rat. *Clin Sci Mol Med* **50**, 511-523 (1976).

43. Batt RM, Rutgers HC, Sancak AA. Enteric bacteria: friend or foe? *J Small Anim Pract* **37**, 261-267 (1996).

44. Bauchart-Thevret C, Stoll B, Chacko S, Burrin DG. Sulfur amino acid deficiency upregulates intestinal methionine cycle activity and suppresses epithelial growth in neonatal pigs. *Am J Physiol Endocrinol Metab* **296**, E1239-1250 (2009).

45. Baxter GM. Alterations of endothelium-dependent digital vascular responses in horses given low-dose endotoxin. *Vet Surg* **24**, 87-96 (1995).

46. Baxter MFA, Greene ES, Kidd MT, Tellez-Isaias G, Orlowski S, Dridi S. Water amino acid-chelated trace mineral supplementation decreases circulating and intestinal HSP70 and proinflammatory cytokine gene expression in heat-stressed broiler chickens. *J Anim Sci* **98**, (2020).

47. Bayguinov O, Sanders KM. Role of nitric oxide as an inhibitory neurotransmitter in the canine pyloric sphincter. *Am J Physiol* **264**, G975-983 (1993).

48. Bayguinov O, Vogalis F, Morris B, Sanders KM. Patterns of electrical activity and neural responses in canine proximal duodenum. *Am J Physiol* **263**, G887-894 (1992).

49. Bazzocco S, Mattila I, Guyot S, Renard CM, Aura AM. Factors affecting the conversion of apple polyphenols to phenolic acids and fruit matrix to short-chain fatty acids by human faecal microbiota in vitro. *Eur J Nutr* **47**, 442-452 (2008).

50. Beall PT, Karnaky KJ, Jr., Garretson LT, Kuo YJ, Shanbour LL. Inhibition of active sodium transport by cytochalasin B in rat jejunum in vitro. *Biochim Biophys Acta* **763**, 19-26 (1983).

51. Beaulieu JF, Calvert R. Role of thyroxine and insulin on the development of the fetal mouse duodenum in organ culture. *Can J Physiol Pharmacol* **64**, 1137-1142 (1986).

52. Beaulieu JF, Calvert R. Hormonal regulation of epithelial cell proliferation in the fetal mouse duodenum in vitro. *Anat Rec* **217**, 250-255 (1987).

53. Bebenek IG, Solaimani P, Bui P, Hankinson O. CYP2S1 is negatively regulated by corticosteroids in human cell lines. *Toxicol Lett* **209**, 30-34 (2012).

54. Bechi P*, et al.* Folate deficiency in operated terminal ileitis (Crohn's disease). *Ital J Surg Sci* **13**, 13-19 (1983).

55. Bedirli A, Sözüer EM, Muhtaroğlu S, Alper M. The role of oxygen free radicals and nitric oxide in organ injury following hemorrhagic shock and reinfusion. *Int J Surg Investig* **2**, 275-284 (2000).

56. Bedwani JR, Okpako DT. Effects of crude and pure cholera toxin on prostaglandin. *Prostaglandins* **10**, 117-127 (1975).

57. Beharry S*, et al.* Long-term docosahexaenoic acid therapy in a congenic murine model of cystic fibrosis. *Am J Physiol Gastrointest Liver Physiol* **292**, G839-848 (2007).

58. Benderev TV. Acetylcysteine for urinary tract mucolysis. *J Urol* **139**, 353-354 (1988).

59. Bengmark S. Colonic food: pre- and probiotics. *Am J Gastroenterol* **95**, S5-7 (2000).

60. Bengmark S. Nutrition of the critically ill &#8212; a 21st-century perspective. *Nutrients* **5**, 162-207 (2013).

61. Benkó R*, et al.* P2 purinoceptor antagonists inhibit the non-adrenergic, non-cholinergic relaxation of the human colon in vitro. *Neuroscience* **147**, 146-152 (2007).

62. Bennett A, Eley KG, Stockley HL. Modulation by prostaglandins of contractions in guinea-pig ileum. *Prostaglandins* **9**, 377-384 (1975).

63. Bennett A, Eley KG, Stockley HL. Inhibition of peristalsis in guinea-pig isolated ileum and colon by drugs that block prostaglandin synthesis. *Br J Pharmacol* **57**, 335-340 (1976).

64. Bentley-Hewitt KL, Perrott M, Butts CA, Hedderley DI, Stoklosinski HM, Parkar SG. Influence of kiwifruit on gastric and duodenal inflammation-related gene expression in aspirin-induced gastric mucosal damage in rats. *Sci Rep* **10**, 13055 (2020).

65. Beppu F, Kondo H, Kasatani S, Aoki Y, Gotoh N. Quantitative Analysis of the Accumulation of Marine-derived Tocopherol in the Tissue of Mice Fed with Salmon Roe Oil Using HPLC-fluorescence. *J Oleo Sci* **67**, 283-288 (2018).

66. Berg-Candolfi M, Candolfi E, Benet LZ. Suppression of intestinal and hepatic cytochrome P4503A in murine Toxoplasma infection. Effects of N-acetylcysteine and N(G)-monomethyl-L-arginine on the hepatic suppression. *Xenobiotica* **26**, 381-394 (1996).

67. Berko RM, Dubin A. Identification and characterization of prostaglandin-binding components in rat ileum. *Clin Physiol Biochem* **3**, 307-313 (1985).

68. Berlin I, Decroix D, Molinier P, Herrmann MA, Peraudeau P, Scheck F. [Comparison between effects of morning and evening administration of effervescent calcium carbasalate (equivalent of 160 mg aspirin) on the gastroduodenal mucosa in healthy volunteers]. *Pathol Biol (Paris)* **45**, 514-520 (1997).

69. Bersimbaev RI, Yugai YE, Hanson PJ, Tzoy IG. Effect of nitric oxide on apoptotic activity in the rat gastrointestinal tract. *Eur J Pharmacol* **423**, 9-16 (2001).

70. Bertacco A*, et al.* Modulation of Intestinal Microbiome Prevents Intestinal Ischemic Injury. *Front Physiol* **8**, 1064 (2017).

71. Bertelsen LS, Bukhave K. Oxidant-stimulated chloride secretion in rat jejunum in vitro is mediated by eicosanoids. *Dig Dis Sci* **48**, 598-604 (2003).

72. Bertelsen LS, Eckmann L, Barrett KE. Prolonged interferon-gamma exposure decreases ion transport, NKCC1, and Na+-K+-ATPase expression in human intestinal xenografts in vivo. *Am J Physiol Gastrointest Liver Physiol* **286**, G157-165 (2004).

73. Bertrand J*, et al.* Enteral glutamine infusion modulates ubiquitination of heat shock proteins, Grp-75 and Apg-2, in the human duodenal mucosa. *Amino Acids* **46**, 1059-1067 (2014).

74. Bertrand RL*, et al.* A Western diet increases serotonin availability in rat small intestine. *Endocrinology* **152**, 36-47 (2011).

75. Bertuccini L*, et al.* Lactoferrin prevents invasion and inflammatory response following E. coli strain LF82 infection in experimental model of Crohn's disease. *Dig Liver Dis* **46**, 496-504 (2014).

76. Beubler E, Hinterleitner T, Horina G. Protein kinase C and intestinal fluid secretion: involvement of prostaglandin E2 but not of 5-hydroxytryptamine. *Eur J Pharmacol* **182**, 543-548 (1990).

77. Beubler E, Juan H. The function of prostaglandins in transmucosal water movement and blood flow in the rat jejunum. *Naunyn Schmiedebergs Arch Pharmacol* **299**, 89-94 (1977).

78. Beubler E, Juan H. PGE-release, blood flow and transmucosal water movement after mechanical stimulation of the rat jejunal mucosa. *Naunyn Schmiedebergs Arch Pharmacol* **305**, 91-95 (1978).

79. Beubler E, Kollar G, Saria A, Bukhave K, Rask-Madsen J. Involvement of 5-hydroxytryptamine, prostaglandin E2, and cyclic adenosine monophosphate in cholera toxin-induced fluid secretion in the small intestine of the rat in vivo. *Gastroenterology* **96**, 368-376 (1989).

80. Beubler E, Schuligoi R. Mechanisms of cholera toxin-induced diarrhea. *Ann N Y Acad Sci* **915**, 339-346 (2000).

81. Beubler E, Schuligoi R, Chopra AK, Ribardo DA, Peskar BA. Cholera toxin induces prostaglandin synthesis via post-transcriptional activation of cyclooxygenase-2 in the rat jejunum. *J Pharmacol Exp Ther* **297**, 940-945 (2001).

82. Bhattacharya D*, et al.* Anti-virulence activity of polyphenolic fraction isolated from Kombucha against Vibrio cholerae. *Microb Pathog* **140**, 103927 (2020).

83. Bhattacharyya S, Ghosh S, Shant J, Ganguly NK, Majumdar S. Role of the W07-toxin on Vibrio cholerae-induced diarrhoea. *Biochim Biophys Acta* **1670**, 69-80 (2004).

84. Bian XC, Bertrand PP, Furness JB, Bornstein JC. Evidence for functional NK1-tachykinin receptors on motor neurones supplying the circular muscle of guinea-pig small and large intestine. *Neurogastroenterol Motil* **12**, 307-315 (2000).

85. Bianchi J, Wilson FA, Rose RC. Dehydroascorbic acid and ascorbic acid transport systems in the guinea pig ileum. *Am J Physiol* **250**, G461-468 (1986).

86. Bianchi Porro G, Lazzaroni M, Petrillo M. Double-blind, double-dummy endoscopic comparison of the mucosal protective effects of misoprostol versus ranitidine on naproxen-induced mucosal injury to the stomach and duodenum in rheumatic patients. *Am J Gastroenterol* **92**, 663-667 (1997).

87. Bianchi-Santamaria A, Dell'orti M, Frigoli G, Gobbi M, Arnaboldi A, Santamaria L. beta-Carotene storage in rat organs following carrier mediated supplementation. *Int J Vitam Nutr Res* **64**, 15-20 (1994).

88. Bias P, Buchner A, Klesser B, Laufer S. The gastrointestinal tolerability of the LOX/COX inhibitor, licofelone, is similar to placebo and superior to naproxen therapy in healthy volunteers: results from a randomized, controlled trial. *Am J Gastroenterol* **99**, 611-618 (2004).

89. Bieger J*, et al.* Tissue distribution of quercetin in pigs after long-term dietary supplementation. *J Nutr* **138**, 1417-1420 (2008).

90. Bieniek K. Effect of duodenal glucose infusion on blood glucose concentration in endotoxin-treated calves. *Zentralbl Veterinarmed A* **45**, 481-490 (1998).

91. Bilski J, Konturek SJ. Role of nitric oxide in gastroduodenal alkaline secretion. *J Physiol Pharmacol* **45**, 541-553 (1994).

92. Bissonnette N*, et al.* Effect of a post-weaning diet supplemented with functional feed additives on ileal transcriptome activity and serum cytokines in piglets challenged with lipopolysaccharide. *Vet Immunol Immunopathol* **182**, 136-149 (2016).

93. Bjarnason I*, et al.* Intestinal inflammation, ileal structure and function in HIV. *Aids* **10**, 1385-1391 (1996).

94. Bjersing JL, Telemo E, Dahlgren U, Hanson LA. Loss of ileal IgA+ plasma cells and of CD4+ lymphocytes in ileal Peyer's patches of vitamin A deficient rats. *Clin Exp Immunol* **130**, 404-408 (2002).

95. Björkman AC, Mobacken H, Kastrup W, Andersson H. Changes in food consumption and its nutritional quality when on a gluten-free diet for dermatitis herpetiformis. *Hum Nutr Appl Nutr* **39**, 124-129 (1985).

96. Bjørneboe A, Bjørneboe GE, Bodd E, Hagen BF, Kveseth N, Drevon CA. Transport and distribution of alpha-tocopherol in lymph, serum and liver cells in rats. *Biochim Biophys Acta* **889**, 310-315 (1986).

97. Black BL. Morphological development of the epithelium of the embryonic chick intestine in culture: influence of thyroxine and hydrocortisone. *Am J Anat* **153**, 573-599 (1978).

98. Black BL. Influence of hormones on glycogen and glucose metabolism in embryonic chick intestine. *Am J Physiol* **254**, G65-73 (1988).

99. Black BL, Moog F. Goblet cells in embryonic intestine: accelerated differentiation in culture. *Science* **197**, 368-370 (1977).

100. Black DD, Wang H, Hunter F, Zhan R. Intestinal expression of apolipoprotein A-IV and C-III is coordinately regulated by dietary lipid in newborn swine. *Biochem Biophys Res Commun* **221**, 619-624 (1996).

101. Blahos J, Care AD, Sommerville BA. The effect of betamethasone on duodenal calcium absorption and 1,25-dihydroxy vitamin D3 production in the chick. *Horm Metab Res* **15**, 197-200 (1983).

102. Blake HH, Henning SJ. Weaning in the rat: a study of hormonal influences. *Am J Physiol* **244**, R537-543 (1983).

103. Blikslager A, Hunt E, Guerrant R, Rhoads M, Argenzio R. Glutamine transporter in crypts compensates for loss of villus absorption in bovine cryptosporidiosis. *Am J Physiol Gastrointest Liver Physiol* **281**, G645-653 (2001).

104. Blikslager AT, Roberts MC, Argenzio RA. Prostaglandin-induced recovery of barrier function in porcine ileum is triggered by chloride secretion. *Am J Physiol* **276**, G28-36 (1999).

105. Blikslager AT, Roberts MC, Rhoads JM, Argenzio RA. Prostaglandins I2 and E2 have a synergistic role in rescuing epithelial barrier function in porcine ileum. *J Clin Invest* **100**, 1928-1933 (1997).

106. Blikslager AT, Zimmel DN, Young KM, Campbell NB, Little D, Argenzio RA. Recovery of ischaemic injured porcine ileum: evidence for a contributory role of COX-1 and COX-2. *Gut* **50**, 615-623 (2002).

107. Blockwood JM, Hsieh J, Fewel J, Rush BF, Jr. Tissue metabolites in endotoxin and hemorrhagic shock; a comparison. *Arch Surg* **107**, 181-185 (1973).

108. Blower P. The science--equivalent efficacy and diminished risk. *Eur J Rheumatol Inflamm* **11**, 29-37 (1991).

109. Blumenstein I, Keserü B, Wolter F, Stein J. The chemopreventive agent resveratrol stimulates cyclic AMP-dependent chloride secretion in vitro. *Clin Cancer Res* **11**, 5651-5656 (2005).

110. Bodd M, Kim CY, Lundin KE, Sollid LM. T-cell response to gluten in patients with HLA-DQ2.2 reveals requirement of peptide-MHC stability in celiac disease. *Gastroenterology* **142**, 552-561 (2012).

111. Boeckxstaens GE, De Man JG, De Winter BY, Herman AG, Pelckmans PA. Pharmacological similarity between nitric oxide and the nitrergic neurotransmitter in the canine ileocolonic junction. *Eur J Pharmacol* **264**, 85-89 (1994).

112. Boeckxstaens GE, De Man JG, De Winter BY, Moreels TG, Herman AG, Pelckmans PA. Bioassay and pharmacological characterization of the nitrergic neurotransmitter. *Arch Int Pharmacodyn Ther* **329**, 11-26 (1995).

113. Boeckxstaens GE*, et al.* Evidence for VIP(1)/PACAP receptors in the afferent pathway mediating surgery-induced fundic relaxation in the rat. *Br J Pharmacol* **131**, 705-710 (2000).

114. Boeckxstaens GE, Pelckmans PA, Bult H, De Man JG, Herman AG, Van Maercke YM. Non-adrenergic non-cholinergic relaxation mediated by nitric oxide in the canine ileocolonic junction. *Eur J Pharmacol* **190**, 239-246 (1990).

115. Boeckxstaens GE, Pelckmans PA, Bult H, De Man JG, Herman AG, van Maercke YM. Evidence for nitric oxide as mediator of non-adrenergic non-cholinergic relaxations induced by ATP and GABA in the canine gut. *Br J Pharmacol* **102**, 434-438 (1991).

116. Bogers JJ, Pelckmans PA, Boeckxstaens GE, De Man JG, Herman AG, Van Maercke YM. The role of nitric oxide in serotonin-induced relaxations in the canine terminal ileum and ileocolonic junction. *Naunyn Schmiedebergs Arch Pharmacol* **344**, 716-719 (1991).

117. Boguski MS, Elshourbagy N, Taylor JM, Gordon JI. Rat apolipoprotein A-IV contains 13 tandem repetitions of a 22-amino acid segment with amphipathic helical potential. *Proc Natl Acad Sci U S A* **81**, 5021-5025 (1984).

118. Bohlen HG. Mechanism of increased vessel wall nitric oxide concentrations during intestinal absorption. *Am J Physiol* **275**, H542-550 (1998).

119. Bohlen HG, Nase GP. Obesity lowers hyperglycemic threshold for impaired in vivo endothelial nitric oxide function. *Am J Physiol Heart Circ Physiol* **283**, H391-397 (2002).

120. Bohn T*, et al.* Mind the gap-deficits in our knowledge of aspects impacting the bioavailability of phytochemicals and their metabolites--a position paper focusing on carotenoids and polyphenols. *Mol Nutr Food Res* **59**, 1307-1323 (2015).

121. Bolten W, Gomes JA, Stead H, Geis GS. The gastroduodenal safety and efficacy of the fixed combination of diclofenac and misoprostol in the treatment of osteoarthritis. *Br J Rheumatol* **31**, 753-758 (1992).

122. Bonfá G*, et al.* CCR5 controls immune and metabolic functions during Toxoplasma gondii infection. *PLoS One* **9**, e104736 (2014).

123. Boontiam W, Jung B, Kim YY. Effects of lysophospholipid supplementation to lower nutrient diets on growth performance, intestinal morphology, and blood metabolites in broiler chickens. *Poult Sci* **96**, 593-601 (2017).

124. Borel P*, et al.* [Recent knowledge about intestinal absorption and cleavage of carotenoids]. *Ann Biol Clin (Paris)* **63**, 165-177 (2005).

125. Borel P*, et al.* Processing of vitamin A and E in the human gastrointestinal tract. *Am J Physiol Gastrointest Liver Physiol* **280**, G95-g103 (2001).

126. Borges EL, Cabral BM, Braga AA, Neves MJ, Santos RA, Rogana E. Effect of angiotensin-(1-7) on jejunal absorption of water in rats. *Peptides* **23**, 51-56 (2002).

127. Botchlett R*, et al.* Glucose and Palmitate Differentially Regulate PFKFB3/iPFK2 and Inflammatory Responses in Mouse Intestinal Epithelial Cells. *Sci Rep* **6**, 28963 (2016).

128. Botting JH, Salzmann R. The effect of indomethacin on the release of prostaglandin E2 and acetylcholine from guinea-pig isolated ileum at rest and during field stimulation. *Br J Pharmacol* **50**, 119-124 (1974).

129. Bouchard MJ, Chorfi Y, Létourneau-Montminy MP, Guay F. Effects of deoxynivalenol and sodium meta-bisulphite on nutrient digestibility in growing pigs. *Arch Anim Nutr* **73**, 360-373 (2019).

130. Boughton-Smith NK, Evans SM, Laszlo F, Whittle BJ, Moncada S. The induction of nitric oxide synthase and intestinal vascular permeability by endotoxin in the rat. *Br J Pharmacol* **110**, 1189-1195 (1993).

131. Boukhettala N*, et al.* Methotrexate induces intestinal mucositis and alters gut protein metabolism independently of reduced food intake. *Am J Physiol Endocrinol Metab* **296**, E182-190 (2009).

132. Bouquet J, Sinaasappel M, Neijens HJ. Malabsorption in cystic fibrosis: mechanisms and treatment. *J Pediatr Gastroenterol Nutr* **7 Suppl 1**, S30-35 (1988).

133. Boušová I*, et al.* Influence of diet supplementation with green tea extract on drug-metabolizing enzymes in a mouse model of monosodium glutamate-induced obesity. *Eur J Nutr* **55**, 361-371 (2016).

134. Bouteloup-Demange C, Claeyssens S, Maillot C, Lavoinne A, Lerebours E, Dechelotte P. Effects of enteral glutamine on gut mucosal protein synthesis in healthy humans receiving glucocorticoids. *Am J Physiol Gastrointest Liver Physiol* **278**, G677-681 (2000).

135. Boza JJ*, et al.* Effect of glutamine supplementation of the diet on tissue protein synthesis rate of glucocorticoid-treated rats. *Nutrition* **17**, 35-40 (2001).

136. Brackett DJ*, et al.* An assessment of plasma histamine concentrations during documented endotoxic shock. *Agents Actions* **31**, 263-274 (1990).

137. Brammer GL. Duodenum is not a consistent source of melatonin in rats. *Life Sci* **55**, 775-787 (1994).

138. Brandt A, Jin CJ, Nolte K, Sellmann C, Engstler AJ, Bergheim I. Short-Term Intake of a Fructose-, Fat- and Cholesterol-Rich Diet Causes Hepatic Steatosis in Mice: Effect of Antibiotic Treatment. *Nutrients* **9**, (2017).

139. Brandt A*, et al.* Consumption of decaffeinated coffee protects against the development of early non-alcoholic steatohepatitis: Role of intestinal barrier function. *Redox Biol* **21**, 101092 (2019).

140. Brencher L, Verhaegh R, Kirsch M. Attenuation of intestinal ischemia-reperfusion-injury by β-alanine: a potentially glycine-receptor mediated effect. *J Surg Res* **211**, 233-241 (2017).

141. Brennan KM*, et al.* Comparison of gene expression profiles of the jejunum of broilers supplemented with a yeast cell wall-derived mannan oligosaccharide versus bacitractin methylene disalicylate. *Br Poult Sci* **54**, 238-246 (2013).

142. Brewer LM, Corradino RA. Hydrocortisone and 1,25(OH)2D3: role in proliferative responses of the embryonic chick duodenum in organ culture. *Horm Metab Res* **15**, 557-561 (1983).

143. Bridén S, Flemström G. Alkaline secretion by Necturus proximal duodenal mucosa. *Acta Physiol Scand* **137**, 481-487 (1989).

144. Brown DR, Southern LL. Effect of citric and ascorbic acids on performance and intestinal pH of chicks. *Poult Sci* **64**, 1399-1401 (1985).

145. Brown MA, Smith PL. Endothelin: a potent stimulator of intestinal ion secretion in vitro. *Regul Pept* **36**, 1-19 (1991).

146. Brown RO, Forloines-Lynn S, Cross RE, Heizer WD. Chromium deficiency after long-term total parenteral nutrition. *Dig Dis Sci* **31**, 661-664 (1986).

147. Bruce D, Cantorna MT. Intrinsic requirement for the vitamin D receptor in the development of CD8αα-expressing T cells. *J Immunol* **186**, 2819-2825 (2011).

148. Bruins MJ, Luiking YC, Soeters PB, Lamers WH, Akkermans LM, Deutz NE. Effects of long-term intravenous and intragastric L-arginine intervention on jejunal motility and visceral nitric oxide production in the hyperdynamic compensated endotoxaemic pig. *Neurogastroenterol Motil* **16**, 819-828 (2004).

149. Brun P*, et al.* Herpes Simplex Virus Type 1 Infects Enteric Neurons and Triggers Gut Dysfunction via Macrophage Recruitment. *Front Cell Infect Microbiol* **8**, 74 (2018).

150. Brunsden AM, Grundy D. Sensitization of visceral afferents to bradykinin in rat jejunum in vitro. *J Physiol* **521 Pt 2**, 517-527 (1999).

151. Brunsson I, Sjöqvist A, Jodal M, Lundgren O. Mechanisms underlying the intestinal fluid secretion evoked by nociceptive serosal stimulation of the rat. *Naunyn Schmiedebergs Arch Pharmacol* **328**, 439-445 (1985).

152. Bruun S*, et al.* Satiety Factors Oleoylethanolamide, Stearoylethanolamide, and Palmitoylethanolamide in Mother's Milk Are Strongly Associated with Infant Weight at Four Months of Age-Data from the Odense Child Cohort. *Nutrients* **10**, (2018).

153. Buchholz BM*, et al.* Hydrogen-enriched preservation protects the isogeneic intestinal graft and amends recipient gastric function during transplantation. *Transplantation* **92**, 985-992 (2011).

154. Bukhave K, Rask-Madsen J. Prostaglandin E2 in jejunal fluids and its potential diagnostic value for selecting patients with indomethacin-sensitive diarrhoea. *Eur J Clin Invest* **11**, 191-197 (1981).

155. Bulbuller N, Dogru O, Yekeler H, Cetinkaya Z, Ilhan N, Kirkil C. Effect of melatonin on wound healing in normal and pinealectomized rats. *J Surg Res* **123**, 3-7 (2005).

156. Büller HA, Rings EH, Montgomery RK, Sasak WV, Grand RJ. Further studies of glycosylation and intracellular transport of lactase-phlorizin hydrolase in rat small intestine. *Biochem J* **263**, 249-254 (1989).

157. Bult H, Boeckxstaens GE, Pelckmans PA, Jordaens FH, Van Maercke YM, Herman AG. Nitric oxide as an inhibitory non-adrenergic non-cholinergic neurotransmitter. *Nature* **345**, 346-347 (1990).

158. Burakoff R, Nastos E, Won S, Percy WH. Comparison of the effects of leukotrienes B4 and D4 on distal colonic motility in the rabbit in vivo. *Am J Physiol* **257**, G860-864 (1989).

159. Burcu B, Kanter M, Orhon ZN, Yarali O, Karabacak R. Protective Effects of Vitamin E on Methotrexate-Induced Jejunal Mucosal Damage in Rats. *Anal Quant Cytopathol Histpathol* **38**, 87-94 (2016).

160. Burghuber OC*, et al.* Leukotriene inhibitors attenuate rat lung injury induced by hydrogen peroxide. *Am Rev Respir Dis* **131**, 778-785 (1985).

161. Burka JF, Eyre P. A pharmacological study of SRS-A on the bovine cutaneous vasculature. *Can J Physiol Pharmacol* **55**, 904-908 (1977).

162. Burn J, Mathers J, Bishop DT. Genetics, inheritance and strategies for prevention in populations at high risk of colorectal cancer (CRC). *Recent Results Cancer Res* **191**, 157-183 (2013).

163. Buts JP, De Meyer R. Intestinal development in the suckling rat: effects of weaning, diet composition, and glucocorticoids on thymidine kinase activity and DNA synthesis. *Pediatr Res* **18**, 145-150 (1984).

164. Buts JP, Delacroix DL. Ontogenic changes in secretory component expression by villous and crypt cells of rat small intestine. *Immunology* **54**, 181-187 (1985).

165. Caglikulekci M*, et al.* [Effect of antithrombin-III (AT-III) on intestinal epithelium changes related to obstructive icterus: experimental study in rats]. *Ann Chir* **129**, 273-277 (2004).

166. Cai J, Zhang Q, Wastney ME, Weaver CM. Calcium bioavailability and kinetics of calcium ascorbate and calcium acetate in rats. *Exp Biol Med (Maywood)* **229**, 40-45 (2004).

167. Cakir M, Mungan I, Karahan C, Can G, Okten A. Necrotizing enterocolitis increases the bone resorption in premature infants. *Early Hum Dev* **82**, 405-409 (2006).

168. Camacho RC*, et al.* Portal venous hyperinsulinemia does not stimulate gut glucose absorption in the conscious dog. *Metabolism* **53**, 1290-1295 (2004).

169. Campbell DJ, Habener JF. Angiotensinogen gene is expressed and differentially regulated in multiple tissues of the rat. *J Clin Invest* **78**, 31-39 (1986).

170. Cancelas J*, et al.* Glucagon-like peptide 1 content of intestinal tract in adult rats injected with streptozotocin either during neonatal period or 7 d before sacrifice. *Endocrine* **19**, 279-286 (2002).

171. Canning DA, Perman JA, Jeffs RD, Gearhart JP. Nutritional consequences of bowel segments in the lower urinary tract. *J Urol* **142**, 509-511; discussion 520-501 (1989).

172. Cao H, Xu J, Liu H, Meng FB, Qiu JF, Wu ZY. Influence of nitric oxide synthase and cyclooxygenase blockade on expression of cyclooxygenase and hemodynamics in rats with portal hypertension. *Hepatobiliary Pancreat Dis Int* **5**, 564-569 (2006).

173. Capasso A, Pinto A, Sorrentino L, Cirino G. Dexamethasone inhibition of acute opioid physical dependence in vitro is reverted by anti-lipocortin-1 and mimicked by anti-type II extracellular PLA2 antibodies. *Life Sci* **61**, Pl 127-134 (1997).

174. Capasso A, Sorrentino L, Pinto A. The role of nitric oxide in the development of opioid withdrawal induced by naloxone after acute treatment with mu- and kappa-opioid receptor agonists. *Eur J Pharmacol* **359**, 127-131 (1998).

175. Capasso F, Mascolo N, Autore G. Enhancement by levamisole of the contractions induced by prostaglandin E2 in the guinea-pig isolated ileum. *Prostaglandins* **23**, 427-432 (1982).

176. Capasso F, Mascolo N, Romano V, Parkinson N, Bennett A. Potentiation by phenolphthalein of the responses of guinea-pig ileum and rat stomach strip to PGE2 and other agonists. *Eur J Pharmacol* **145**, 1-5 (1988).

177. Carlsen H, Alexander G, Austenaa LM, Ebihara K, Blomhoff R. Molecular imaging of the transcription factor NF-kappaB, a primary regulator of stress response. *Mutat Res* **551**, 199-211 (2004).

178. Carnathan GW, Sanner JH, Thompson JM, Prusa CM, Miyano M. Antagonism of the in vivo and in vitro effects of leukotriene D4 by SC-39070 in guinea pigs. *Agents Actions* **20**, 124-132 (1987).

179. Cartwright JA*, et al.* Vitamin D Receptor Expression in Dogs. *J Vet Intern Med* **32**, 764-774 (2018).

180. Castellano PM, Pastoriza MA, Martińez de Bertorello M, Bertorello HE, Horas JA. In vitro sodium salicylate release from a sucrose polyester matrix. *J Pharm Sci* **78**, 561-562 (1989).

181. Castilla-Cortazar I*, et al.* Impaired intestinal sugar transport in cirrhotic rats: correction by low doses of insulin-like growth factor I. *Gastroenterology* **113**, 1180-1187 (1997).

182. Castro GA, Harari Y. Immunoregulation of endometrial and jejunal epithelia sensitized by infection. *Int Arch Allergy Appl Immunol* **95**, 184-190 (1991).

183. Castro IC*, et al.* Arginine decreases Cryptosporidium parvum infection in undernourished suckling mice involving nitric oxide synthase and arginase. *Nutrition* **28**, 678-685 (2012).

184. Catanoso M*, et al.* Gastro-intestinal permeability is increased in patients with limited systemic sclerosis. *Scand J Rheumatol* **30**, 77-81 (2001).

185. Catarzi S*, et al.* Oxidative state and IL-6 production in intestinal myofibroblasts of Crohn's disease patients. *Inflamm Bowel Dis* **17**, 1674-1684 (2011).

186. Celano P, Jumawan J, Horowitz C, Lau H, Koldovsky O. Prenatal induction of sucrase activity in rat jejunum. *Biochem J* **162**, 469-472 (1977).

187. Cermak R, Landgraf S, Wolffram S. Quercetin glucosides inhibit glucose uptake into brush-border-membrane vesicles of porcine jejunum. *Br J Nutr* **91**, 849-855 (2004).

188. Cesaro A*, et al.* Differential expression and regulation of ADAM17 and TIMP3 in acute inflamed intestinal epithelia. *Am J Physiol Gastrointest Liver Physiol* **296**, G1332-1343 (2009).

189. Cetinkaya Z*, et al.* Influence of some substances on bacterial translocation in the rat. *World J Surg* **26**, 9-12 (2002).

190. Cetta F, Lombardo F, Rossi S. Large foreign body as a nidus for a common duct stone in a patient without spontaneous biliary enteric fistula or previous abdominal surgery. *HPB Surg* **6**, 235-242 (1993).

191. Ceulemans LJ*, et al.* Farnesoid X Receptor Activation Attenuates Intestinal Ischemia Reperfusion Injury in Rats. *PLoS One* **12**, e0169331 (2017).

192. Chadwick VS, Schlup MM, Cooper BT, Broom MF. Enzymes degrading bacterial chemotactic F-met peptides in human ileal and colonic mucosa. *J Gastroenterol Hepatol* **5**, 375-381 (1990).

193. Chakravortty D, Kumar KS. Induction of cell proliferation and collagen synthesis in human small intestinal lamina propria fibroblasts by lipopolysaccharide: possible involvement of nitric oxide. *Biochem Biophys Res Commun* **240**, 458-463 (1997).

194. Chan SD, Chiu DK, Atkins D. Mechanism of the regulation of the 1 alpha,25-dihydroxyvitamin D3 receptor in the rat jejunum by glucocorticoids. *J Endocrinol* **103**, 295-300 (1984).

195. Chandrasekharan B*, et al.* Interactions Between Commensal Bacteria and Enteric Neurons, via FPR1 Induction of ROS, Increase Gastrointestinal Motility in Mice. *Gastroenterology* **157**, 179-192.e172 (2019).

196. Chang EB, Musch MW, Drabik-Arvans D, Rao MC. Phorbol ester inhibition of chicken intestinal brush-border sodium-proton exchange. *Am J Physiol* **260**, C1264-1272 (1991).

197. Chang X, Wang LL, Lian SJ, Tang Q, Chen P, Wang H. [Effect of oral glutamine on intestinal barrier function in young rats with endotoxemia]. *Zhongguo Dang Dai Er Ke Za Zhi* **12**, 809-811 (2010).

198. Chau RM, Patel BA. Determination of serotonin, melatonin and metabolites in gastrointestinal tissue using high-performance liquid chromatography with electrochemical detection. *Biomed Chromatogr* **23**, 175-181 (2009).

199. Chaudhuri L, Vedasiromoni JR, Besra SE, Ganguly DK. Role of nitric oxide in gastrokinetic effects of the new benzoxepine derivative exepanol hydrochloride. *Arzneimittelforschung* **46**, 923-925 (1996).

200. Chen CF, Leu FJ, Chen HI, Wang D, Chou SJ. Ischemia/reperfusion-induced low reactivity of the rat superior mesenteric vascular bed is associated with expression of nitric oxide synthases. *Transplant Proc* **38**, 2216-2220 (2006).

201. Chen D*, et al.* Oxytocin evokes a pulsatile PGE2 release from ileum mucosa and is required for repair of intestinal epithelium after injury. *Sci Rep* **5**, 11731 (2015).

202. Chen F, Fan GQ, Zhang Z, Zhang R, Deng ZY, McClements DJ. Encapsulation of omega-3 fatty acids in nanoemulsions and microgels: Impact of delivery system type and protein addition on gastrointestinal fate. *Food Res Int* **100**, 387-395 (2017).

203. Chen HH*, et al.* Oral administration of acarbose ameliorates imiquimod-induced psoriasis-like dermatitis in a mouse model. *Int Immunopharmacol* **33**, 70-82 (2016).

204. Chen J, Cai S, Ye S, Xie Q, Yu F. [Antiallergic effects of tranilast in rats and guinea pigs]. *Hua Xi Yi Ke Da Xue Xue Bao* **28**, 179-183 (1997).

205. Chen J*, et al.* Dysbiosis of intestinal microbiota and decrease in paneth cell antimicrobial peptide level during acute necrotizing pancreatitis in rats. *PLoS One* **12**, e0176583 (2017).

206. Chen JC*, et al.* Selective inhibition of inducible nitric oxide in ischemia-reperfusion of rat small intestine. *J Formos Med Assoc* **99**, 213-218 (2000).

207. Chen X*, et al.* Leukotriene A4 hydrolase in rat and human esophageal adenocarcinomas and inhibitory effects of bestatin. *J Natl Cancer Inst* **95**, 1053-1061 (2003).

208. Chen X, Qin FJ, Sun YH. [An observation of the effects of Tiopronin on the oxygen free radicals in severely scalded rats receiving delayed fluid resuscitation]. *Zhonghua Shao Shang Za Zhi* **19**, 109-111 (2003).

209. Chen X, Valente JF, Alexander JW. The effect of sennosides on bacterial translocation and survival in a model of acute hemorrhagic pancreatitis. *Pancreas* **18**, 39-46 (1999).

210. Chen X, Yang G, Ding WY, Bondoc F, Curtis SK, Yang CS. An esophagogastroduodenal anastomosis model for esophageal adenocarcinogenesis in rats and enhancement by iron overload. *Carcinogenesis* **20**, 1801-1808 (1999).

211. Chen YT, Tsai SH, Sheu SY, Tsai LH. Ghrelin improves LPS-induced gastrointestinal motility disturbances: roles of NO and prostaglandin E2. *Shock* **33**, 205-212 (2010).

212. Cheng J, Krausz KW, Tanaka N, Gonzalez FJ. Chronic exposure to rifaximin causes hepatic steatosis in pregnane X receptor-humanized mice. *Toxicol Sci* **129**, 456-468 (2012).

213. Cheng JT, Shinozuka K. Participation of prostaglandin E2 in the purinergic neurotransmission of gut. *Gut* **28**, 1605-1608 (1987).

214. Cherkasova TD, Vengrov PR, Melikhov VI, Avrorov VP, Iurkiv VA. [Levels of prostaglandin E1 and F2 alpha in the dynamics of toxic-infectious shock induced by Yersinia pestis]. *Biull Eksp Biol Med* **105**, 313-315 (1988).

215. Chernova MN*, et al.* Acute regulation of the SLC26A3 congenital chloride diarrhoea anion exchanger (DRA) expressed in Xenopus oocytes. *J Physiol* **549**, 3-19 (2003).

216. Chikwati EM*, et al.* Interaction of soyasaponins with plant ingredients in diets for Atlantic salmon, Salmo salar L. *Br J Nutr* **107**, 1570-1590 (2012).

217. Chiu JH, Lui WY, Chen YL, Hong CY. Local somatothermal stimulation inhibits the motility of sphincter of Oddi in cats, rabbits and humans through nitrergic neural release of nitric oxide. *Life Sci* **63**, 413-428 (1998).

218. Choi KM*, et al.* Regulation of interstitial cells of Cajal in the mouse gastric body by neuronal nitric oxide. *Neurogastroenterol Motil* **19**, 585-595 (2007).

219. Choi Y*, et al.* Vitamin E (α-tocopherol) consumption influences gut microbiota composition. *Int J Food Sci Nutr* **71**, 221-225 (2020).

220. Chow A, Zhou W, Jacobson R. Regulation of AE2 Cl-/HCO3- exchanger during intestinal development. *Am J Physiol* **271**, G330-337 (1996).

221. Christopher TA, Ma XL, Lefer AM. Beneficial actions of S-nitroso-N-acetylpenicillamine, a nitric oxide donor, in murine traumatic shock. *Shock* **1**, 19-24 (1994).

222. Chung WSF*, et al.* Prebiotic potential of pectin and pectic oligosaccharides to promote anti-inflammatory commensal bacteria in the human colon. *FEMS Microbiol Ecol* **93**, (2017).

223. Cifelli CJ, Green JB, Green MH. Use of model-based compartmental analysis to study vitamin A kinetics and metabolism. *Vitam Horm* **75**, 161-195 (2007).

224. Cifelli CJ, Ross AC. All-trans-retinoic acid distribution and metabolism in vitamin A-marginal rats. *Am J Physiol Gastrointest Liver Physiol* **291**, G195-202 (2006).

225. Cifelli CJ, Ross AC. Chronic vitamin A status and acute repletion with retinyl palmitate are determinants of the distribution and catabolism of all-trans-retinoic acid in rats. *J Nutr* **137**, 63-70 (2007).

226. Cimini M, Boughner DR, Ronald JA, Johnston DE, Rogers KA. Dermal fibroblasts cultured on small intestinal submucosa: Conditions for the formation of a neotissue. *J Biomed Mater Res A* **75**, 895-906 (2005).

227. Citelli M, Bittencourt LL, da Silva SV, Pierucci AP, Pedrosa C. Vitamin A modulates the expression of genes involved in iron bioavailability. *Biol Trace Elem Res* **149**, 64-70 (2012).

228. Civalleri D*, et al.* [Behavior of serum proteins and lipids after jejuno-ileal bypass for obesity]. *Minerva Chir* **33**, 775-788 (1978).

229. Clark RM, Yao L, She L, Furr HC. A comparison of lycopene and astaxanthin absorption from corn oil and olive oil emulsions. *Lipids* **35**, 803-806 (2000).

230. Clark SB. The uptake of oleic acid by rat small intestine: a comparison of methodologies. *J Lipid Res* **12**, 43-55 (1971).

231. Claro da Silva T, Hiller C, Gai Z, Kullak-Ublick GA. Vitamin D3 transactivates the zinc and manganese transporter SLC30A10 via the Vitamin D receptor. *J Steroid Biochem Mol Biol* **163**, 77-87 (2016).

232. Clemens V, Regen F, Le Bret N, Heuser I, Hellmann-Regen J. Anti-inflammatory effects of minocycline are mediated by retinoid signaling. *BMC Neurosci* **19**, 58 (2018).

233. Clemente CM*, et al.* Sildenafil inhibits duodenal contractility via activation of the NO-K+ channel pathway. *Fundam Clin Pharmacol* **22**, 61-67 (2008).

234. Clerici C*, et al.* Local and systemic effects of intraduodenal exposure to topical gallstone solvents ethyl propionate and methyl tert-butyl ether in the rabbit. *Dig Dis Sci* **42**, 497-502 (1997).

235. Coëffier M*, et al.* Acute enteral glutamine infusion enhances heme oxygenase-1 expression in human duodenal mucosa. *J Nutr* **132**, 2570-2573 (2002).

236. Coëffier M, Marion R, Ducrotté P, Déchelotte P. Modulating effect of glutamine on IL-1beta-induced cytokine production by human gut. *Clin Nutr* **22**, 407-413 (2003).

237. Coëffier M, Marion R, Leplingard A, Lerebours E, Ducrotté P, Déchelotte P. Glutamine decreases interleukin-8 and interleukin-6 but not nitric oxide and prostaglandins e(2) production by human gut in-vitro. *Cytokine* **18**, 92-97 (2002).

238. Coelho JC, Gouma DJ, Li YF, Moody FG, Schlegel JF. Effect of 16,16-dimethyl prostaglandin E2 on the myoelectric activity of the gastrointestinal tract of the opossum. *J Physiol* **377**, 421-427 (1986).

239. Cohen MM, MacDonald WC. Mechanism of aspirin injury to human gastroduodenal mucosa. *Prostaglandins Leukot Med* **9**, 241-255 (1982).

240. Cohen MM, McCready DR, Clark L, Sevelius H. Protection against aspirin-induced antral and duodenal damage with enprostil. A double-blind endoscopic study. *Gastroenterology* **88**, 382-386 (1985).

241. Colette C, Monnier L, Pares Herbute N, Blotman F, Mirouze J. Calcium absorption in corticoid treated subjects effects of a single oral dose of calcitriol. *Horm Metab Res* **19**, 335-338 (1987).

242. Collares EF, Troncon LEA. Effects of dipyrone on the digestive tract. *Braz J Med Biol Res* **52**, e8103 (2019).

243. Colucci M*, et al.* Guinea pig ileum motility stimulation elicited by N-formyl-Met-Leu-Phe (fMLF) involves neurotransmitters and prostanoids. *Peptides* **32**, 266-271 (2011).

244. Combs GF, Jr., Pesti GM. Influence of ascorbic acid on selenium nutrition in the chick. *J Nutr* **106**, 958-966 (1976).

245. Connor BA, Shlim DR, Scholes JV, Rayburn JL, Reidy J, Rajah R. Pathologic changes in the small bowel in nine patients with diarrhea associated with a coccidia-like body. *Ann Intern Med* **119**, 377-382 (1993).

246. Coon S, Shao G, Wisel S, Vulaupalli R, Sundaram U. Mechanism of regulation of rabbit intestinal villus cell brush border membrane Na/H exchange by nitric oxide. *Am J Physiol Gastrointest Liver Physiol* **292**, G475-481 (2007).

247. Copeman M, Matuz J, Leonard AJ, Pearson JP, Dettmar PW, Allen A. The gastroduodenal mucus barrier and its role in protection against luminal pepsins: the effect of 16,16 dimethyl prostaglandin E2, carbopol-polyacrylate, sucralfate and bismuth subsalicylate. *J Gastroenterol Hepatol* **9 Suppl 1**, S55-59 (1994).

248. Cordes T, Lucas A, Divakaruni AS, Murphy AN, Cabrales P, Metallo CM. Itaconate modulates tricarboxylic acid and redox metabolism to mitigate reperfusion injury. *Mol Metab* **32**, 122-135 (2020).

249. Corpe CP*, et al.* Vitamin C transporter Slc23a1 links renal reabsorption, vitamin C tissue accumulation, and perinatal survival in mice. *J Clin Invest* **120**, 1069-1083 (2010).

250. Corradino RA. Hydrocortisone and vitamin D3 stimulation of 32Pi-phosphate accumulation by organ-cultured chick embryo duodenum. *Horm Metab Res* **11**, 519-523 (1979).

251. Corradino RA. Effects of verapamil and dexamethasone on the 1,25-dihydroxyvitamin D3-mediated calcium absorptive mechanism in the organ-cultured embryonic chick duodenum. *Biochem Pharmacol* **34**, 1971-1974 (1985).

252. Correia NA, Cavalcanti PM, Oliveira RB, Ballejo G. Effect of hydroquinone, hydroxocobalamin and carboxy-PTIO on non-adrenergic non-cholinergic nerve mediated relaxations of the rat duodenum. *J Auton Pharmacol* **19**, 233-240 (1999).

253. Corsonello A*, et al.* Adverse Events of Proton Pump Inhibitors: Potential Mechanisms. *Curr Drug Metab* **19**, 142-154 (2018).

254. Costa MD, Bosc LV, Majowicz MP, Vidal NA, Balaszczuk AM, Arranz CT. Atrial natriuretic peptide modifies arterial blood pressure through nitric oxide pathway in rats. *Hypertension* **35**, 1119-1123 (2000).

255. Courtois P, Meuris S, Sener A, Malaisse WJ, Scott FW. Invertase, maltase, lactase, and peroxidase activities in duodenum of BB rats. *Endocrine* **19**, 293-300 (2002).

256. Cowles RA*, et al.* Reversal of intestinal failure-associated liver disease in infants and children on parenteral nutrition: experience with 93 patients at a referral center for intestinal rehabilitation. *J Pediatr Surg* **45**, 84-87; discussion 87-88 (2010).

257. Cox D, Maree AO, Dooley M, Conroy R, Byrne MF, Fitzgerald DJ. Effect of enteric coating on antiplatelet activity of low-dose aspirin in healthy volunteers. *Stroke* **37**, 2153-2158 (2006).

258. Crain FD, Lotspeich FJ, Krause RF. Biosynthesis of retinoic acid by intestinal enzymes of the rat. *J Lipid Res* **8**, 249-254 (1967).

259. Crake T, Crisp AJ, Shearing M, Record CO, Sandle GI. Effect of intraluminal hydrocortisone on solute and water absorption in the human jejunum. *Clin Sci (Lond)* **67**, 105-110 (1984).

260. Crane RJ, Jones KD, Berkley JA. Environmental enteric dysfunction: an overview. *Food Nutr Bull* **36**, S76-87 (2015).

261. Cristol JP, Sirois P. Comparative activity of leukotriene D4, 5,6-dihydroxy-eicosatetraenoic acid and lipoxin A on guinea pig lung parenchyma and ileum smooth muscle. *Res Commun Chem Pathol Pharmacol* **59**, 423-426 (1988).

262. Crouser ED, Julian MW, Weinstein DM, Fahy RJ, Bauer JA. Endotoxin-induced ileal mucosal injury and nitric oxide dysregulation are temporally dissociated. *Am J Respir Crit Care Med* **161**, 1705-1712 (2000).

263. Crowe SE, Sestini P, Perdue MH. Allergic reactions of rat jejunal mucosa. Ion transport responses to luminal antigen and inflammatory mediators. *Gastroenterology* **99**, 74-82 (1990).

264. Croxatto HR, Silva R, Figueroa X, Albertini R, Roblero J, Boric MP. A peptide released by pepsin from kininogen domain 1 is a potent blocker of ANP-mediated diuresis-natriuresis in the rat. *Hypertension* **30**, 897-904 (1997).

265. Cryer B, Feldman M. Effects of very low dose daily, long-term aspirin therapy on gastric, duodenal, and rectal prostaglandin levels and on mucosal injury in healthy humans. *Gastroenterology* **117**, 17-25 (1999).

266. Cryer B, Kliewer D, Sie H, McAllister L, Feldman M. Effects of cutaneous aspirin on the human stomach and duodenum. *Proc Assoc Am Physicians* **111**, 448-456 (1999).

267. Cryer B, Lee E, Feldman M. Factors influencing gastroduodenal mucosal prostaglandin concentrations: roles of smoking and aging. *Ann Intern Med* **116**, 636-640 (1992).

268. Cullen JJ, Doty RC, Ephgrave KS, Hinkhouse MM, Broadhurst K. Changes in intestinal transit and absorption during endotoxemia are dose dependent. *J Surg Res* **81**, 81-86 (1999).

269. Cullen JJ, Hemann LL, Ephgrave KS, Hinkhouse MM. Endotoxin temporarily impairs canine jejunal absorption of water, electrolytes, and glucose. *J Gastrointest Surg* **1**, 286-291 (1997).

270. Cullen JJ, Ledlow A, Murray JA, Conklin JL. The effect of ethanol on sphincter of Oddi motility in vitro. *J Surg Res* **67**, 58-61 (1997).

271. Cupp MS, Combs GF, Jr., Corradino RA. Ascorbate interacts with sodium selenite to increase glutathione peroxidase activity in selenium-deficient chick duodena cultured in vitro. *Biol Trace Elem Res* **20**, 87-94 (1989).

272. Curtis CG, Powell GM, Bradbury A, Rhodes C. The fate of fenclozic acid in the gut and its effect on some aspects of gut metabolism. *Xenobiotica* **13**, 483-496 (1983).

273. Cuzzocrea S, Costantino G, Mazzon E, Caputi AP. Protective effect of N-acetylcysteine on multiple organ failure induced by zymosan in the rat. *Crit Care Med* **27**, 1524-1532 (1999).

274. Cuzzocrea S, Costantino G, Mazzon E, De Sarro A, Caputi AP. Beneficial effects of Mn(III)tetrakis (4-benzoic acid) porphyrin (MnTBAP), a superoxide dismutase mimetic, in zymosan-induced shock. *Br J Pharmacol* **128**, 1241-1251 (1999).

275. Cuzzocrea S*, et al.* IL-6 knock-out mice exhibit resistance to splanchnic artery occlusion shock. *J Leukoc Biol* **66**, 471-480 (1999).

276. Cuzzocrea S, Mazzon E, Costantino G, Serraino I, De Sarro A, Caputi AP. Effects of n-acetylcysteine in a rat model of ischemia and reperfusion injury. *Cardiovasc Res* **47**, 537-548 (2000).

277. Cuzzocrea S*, et al.* Protective effects of a new stable, highly active SOD mimetic, M40401 in splanchnic artery occlusion and reperfusion. *Br J Pharmacol* **132**, 19-29 (2001).

278. Cuzzocrea S*, et al.* Beneficial effects of peroxynitrite decomposition catalyst in a rat model of splanchnic artery occlusion and reperfusion. *Faseb j* **14**, 1061-1072 (2000).

279. Cuzzocrea S, Zingarelli B, Costantino G, Caputi AP. Protective effect of melatonin in a non-septic shock model induced by zymosan in the rat. *J Pineal Res* **25**, 24-33 (1998).

280. Cuzzocrea S, Zingarelli B, Costantino G, Sottile A, Teti D, Caputi AP. Protective effect of poly(ADP-ribose) synthetase inhibition on multiple organ failure after zymosan-induced peritonitis in the rat. *Crit Care Med* **27**, 1517-1523 (1999).

281. D'Alessandro AM*, et al.* Prolongation of canine intestinal allograft survival with RS-61443, cyclosporine, and prednisone. *Transplantation* **55**, 695-700; discussion 700-691 (1993).

282. D'Argenio G*, et al.* Overactivity of the intestinal endocannabinoid system in celiac disease and in methotrexate-treated rats. *J Mol Med (Berl)* **85**, 523-530 (2007).

283. D'Souza A*, et al.* Effects of probiotics, prebiotics, and synbiotics on messenger RNA expression of caveolin-1, NOS, and genes regulating oxidative stress in the terminal ileum of formula-fed neonatal rats. *Pediatr Res* **67**, 526-531 (2010).

284. Dabrowski K. Absorption of ascorbic acid and ascorbic sulfate and ascorbate metabolism in common carp (Cyprinus carpio L.). *J Comp Physiol B* **160**, 549-561 (1990).

285. Dafoe DC, Campbell DA, Jr., Marks WH, Borgstrom A, Lloyd RV, Turcotte JG. Association of inclusion of the donor spleen in pancreaticoduodenal transplantation with rejection. *Transplantation* **40**, 579-584 (1985).

286. Dahan A, Amidon GL. Small intestinal efflux mediated by MRP2 and BCRP shifts sulfasalazine intestinal permeability from high to low, enabling its colonic targeting. *Am J Physiol Gastrointest Liver Physiol* **297**, G371-377 (2009).

287. Dalmasso G, Nguyen HT, Yan Y, Charrier-Hisamuddin L, Sitaraman SV, Merlin D. Butyrate transcriptionally enhances peptide transporter PepT1 expression and activity. *PLoS One* **3**, e2476 (2008).

288. Dalmasso G*, et al.* MicroRNA-92b regulates expression of the oligopeptide transporter PepT1 in intestinal epithelial cells. *Am J Physiol Gastrointest Liver Physiol* **300**, G52-59 (2011).

289. Dalziel JE, Fraser K, Young W, McKenzie CM, Bassett SA, Roy NC. Gastroparesis and lipid metabolism-associated dysbiosis in Wistar-Kyoto rats. *Am J Physiol Gastrointest Liver Physiol* **313**, G62-g72 (2017).

290. Dampf Stone A*, et al.* Resveratrol potentiates vitamin D and nuclear receptor signaling. *J Cell Biochem* **116**, 1130-1143 (2015).

291. Dänicke S, Jeroch H, Simon O. Endogenous N-losses in broilers estimated by a [15N]-isotope dilution technique: effect of dietary fat type and xylanase addition. *Arch Tierernahr* **53**, 75-97 (2000).

292. Daniel EE*, et al.* Do gap junctions play a role in nerve transmissions as well as pacing in mouse intestine? *Am J Physiol Gastrointest Liver Physiol* **292**, G734-745 (2007).

293. Danielsen EM. Tyrosine sulfation, a post-translational modification of microvillar enzymes in the small intestinal enterocyte. *Embo j* **6**, 2891-2896 (1987).

294. Das KM. Pharmacotherapy of inflammatory bowel disease. Part 1. Sulfasalazine. *Postgrad Med* **74**, 141-148, 150-141 (1983).

295. Das M, Ganguly DK. Effect of prostaglandin E2 on acetylcholine release from some peripheral cholinergic nerve terminals. *Eur J Pharmacol* **100**, 41-46 (1984).

296. Dash RP, Ellendula B, Agarwal M, Nivsarkar M. Increased intestinal P-glycoprotein expression and activity with progression of diabetes and its modulation by epigallocatechin-3-gallate: Evidence from pharmacokinetic studies. *Eur J Pharmacol* **767**, 67-76 (2015).

297. Dasilva G, Boller M, Medina I, Storch J. Relative levels of dietary EPA and DHA impact gastric oxidation and essential fatty acid uptake. *J Nutr Biochem* **55**, 68-75 (2018).

298. Davenpeck KL, Gauthier TW, Lefer AM. Inhibition of endothelial-derived nitric oxide promotes P-selectin expression and actions in the rat microcirculation. *Gastroenterology* **107**, 1050-1058 (1994).

299. Davidsson L, Almgren A, Juillerat MA, Hurrell RF. Manganese absorption in humans: the effect of phytic acid and ascorbic acid in soy formula. *Am J Clin Nutr* **62**, 984-987 (1995).

300. Davis WL*, et al.* The immunocytochemical localization of superoxide dismutase in the enterocytes of the avian intestine: the effect of vitamin D3. *Histochem J* **21**, 194-202 (1989).

301. Day G, Evans K, Wharton B. Abnormalities of insulin and growth hormone secreton in children with coeliac disease. *Arch Dis Child* **48**, 41-46 (1973).

302. De Angelis M*, et al.* Salivary and fecal microbiota and metabolome of celiac children under gluten-free diet. *Int J Food Microbiol* **239**, 125-132 (2016).

303. De Backer O, Lefebvre RA. Investigation of a possible interaction between the heme oxygenase/biliverdin reductase and nitric oxide synthase pathway in murine gastric fundus and jejunum. *Eur J Pharmacol* **590**, 369-376 (2008).

304. de Cock P. Erythritol Functional Roles in Oral-Systemic Health. *Adv Dent Res* **29**, 104-109 (2018).

305. De Freitas P, Natali MR, Pereira RV, Miranda Neto MH, Zanoni JN. Myenteric neurons and intestinal mucosa of diabetic rats after ascorbic acid supplementation. *World J Gastroenterol* **14**, 6518-6524 (2008).

306. de Hingh IH, van Goor H, de Man BM, Lomme RM, Bleichrodt RP, Hendriks T. Selective cyclo-oxygenase 2 inhibition affects ileal but not colonic anastomotic healing in the early postoperative period. *Br J Surg* **93**, 489-497 (2006).

307. de Hostos EL, Choy RK, Nguyen T. Developing novel antisecretory drugs to treat infectious diarrhea. *Future Med Chem* **3**, 1317-1325 (2011).

308. De Jonge F*, et al.* Effect of intestinal inflammation on capsaicin-sensitive afferents in the ileum of Schistosoma mansoni-infected mice. *Histochem Cell Biol* **119**, 477-484 (2003).

309. de Kivit S*, et al.* Dietary, nondigestible oligosaccharides and Bifidobacterium breve M-16V suppress allergic inflammation in intestine via targeting dendritic cell maturation. *J Leukoc Biol* **102**, 105-115 (2017).

310. De Luca A, Rand MJ. Responses of rat isolated intestinal segments to stimulation of perivascular mesenteric nerve fibres. *J Auton Pharmacol* **10**, 323-331 (1990).

311. De Man JG*, et al.* Comparison of the pharmacological profile of S-nitrosothiols, nitric oxide and the nitrergic neurotransmitter in the canine ileocolonic junction. *Br J Pharmacol* **114**, 1179-1184 (1995).

312. De Man JG, Boeckxstaens GE, Pelckmans PP, De Winter BY, Herman AG, Van Maercke YM. Prejunctional modulation of the nitrergic innervation of the canine ileocolonic junction via potassium channels. *Br J Pharmacol* **110**, 559-564 (1993).

313. de Oliveira GP*, et al.* Intravenous glutamine administration reduces lung and distal organ injury in malnourished rats with sepsis. *Shock* **41**, 222-232 (2014).

314. De Preter V*, et al.* Pouchitis, similar to active ulcerative colitis, is associated with impaired butyrate oxidation by intestinal mucosa. *Inflamm Bowel Dis* **15**, 335-340 (2009).

315. de Theije CG*, et al.* Intestinal inflammation in a murine model of autism spectrum disorders. *Brain Behav Immun* **37**, 240-247 (2014).

316. De Winter BY, Boeckxstaens GE, De Man JG, Moreels TG, Herman AG, Pelckmans PA. Effect of adrenergic and nitrergic blockade on experimental ileus in rats. *Br J Pharmacol* **120**, 464-468 (1997).

317. Deigner HP, Freyberg CE, Laufer S. Distribution and excretion of [14C]-labelled [2,2-dimethyl-6-(4-chlorophenyl)-7-phenyl-2,3-dihydro-1H-pyrrolizine-5- yl]- [2'-14C]-acetic acid in rats. *Arzneimittelforschung* **45**, 272-276 (1995).

318. Dekkers JA, Akkermans LM, Kroese AB. Effects of the inflammatory mediator prostaglandin E2 on myenteric neurons in guinea pig ileum. *Am J Physiol* **272**, G1451-1456 (1997).

319. del Balzo U, Polley MJ, Levi R. C3a-induced contraction of guinea pig ileum consists of two components: fast histamine-mediated and slow prostanoid-mediated. *J Pharmacol Exp Ther* **248**, 1003-1009 (1989).

320. DeLano FA, Schmid-Schönbein GW. Pancreatic digestive enzyme blockade in the small intestine prevents insulin resistance in hemorrhagic shock. *Shock* **41**, 55-61 (2014).

321. Demacker PN, van Heijst PJ, Stalenhoef AF. A study of the chylomicron metabolism in WHHL rabbits after fat loading. Discrepancy between results based on measurement of apoprotein B-48 or retinyl palmitate. *Biochem J* **285 ( Pt 2)**, 641-646 (1992).

322. Demedts I*, et al.* Neural mechanisms of early postinflammatory dysmotility in rat small intestine. *Neurogastroenterol Motil* **18**, 1102-1111 (2006).

323. Demling RH. Enteral glutamine administration prevents the decrease in cell energy charge potential produced in ileum after a skin burn in the rat. *J Burn Care Rehabil* **21**, 275-279; discussion 274 (2000).

324. Denisov EN. [Results of the clinical use of 75Se-methionine in gastroenterology with different modes of administration]. *Med Radiol (Mosk)* **31**, 5-9 (1986).

325. DeRoover A*, et al.* Luminal contact with University of Wisconsin solution improves human small bowel preservation. *Transplant Proc* **36**, 273-275 (2004).

326. Deschner EE, Alcock N, Okamura T, DeCosse JJ, Sherlock P. Tissue concentrations and proliferative effects of massive doses of ascorbic acid in the mouse. *Nutr Cancer* **4**, 241-246 (1983).

327. Di Marzo V, Izzo AA. Endocannabinoid overactivity and intestinal inflammation. *Gut* **55**, 1373-1376 (2006).

328. Di Paola R*, et al.* Absence of peroxisome proliferators-activated receptors (PPAR)alpha enhanced the multiple organ failure induced by zymosan. *Shock* **26**, 477-484 (2006).

329. Di Paola R, Genovese T, Caputi AP, Threadgill M, Thiemermann C, Cuzzocrea S. Beneficial effects of 5-aminoisoquinolinone, a novel, potent, water-soluble, inhibitor of poly (ADP-ribose) polymerase, in a rat model of splanchnic artery occlusion and reperfusion. *Eur J Pharmacol* **492**, 203-210 (2004).

330. Dickerman RM, Twiest MW, Crudup JW, Turcotte JG. Transplantation of the pancreas into a retroperitoneal jejunal loop. *Am J Surg* **129**, 48-54 (1975).

331. Dickson EJ*, et al.* An enteric occult reflex underlies accommodation and slow transit in the distal large bowel. *Gastroenterology* **132**, 1912-1924 (2007).

332. Ding LA, Li JS. Effects of glutamine on intestinal permeability and bacterial translocation in TPN-rats with endotoxemia. *World J Gastroenterol* **9**, 1327-1332 (2003).

333. Ding Y, Yanagi K, Cheng C, Alaniz RC, Lee K, Jayaraman A. Interactions between gut microbiota and non-alcoholic liver disease: The role of microbiota-derived metabolites. *Pharmacol Res* **141**, 521-529 (2019).

334. Dinis-Oliveira RJ, Pontes H, Bastos ML, Remião F, Duarte JA, Carvalho F. An effective antidote for paraquat poisonings: the treatment with lysine acetylsalicylate. *Toxicology* **255**, 187-193 (2009).

335. Dinning PG, Szczesniak M, Cook IJ. Removal of tonic nitrergic inhibition is a potent stimulus for human proximal colonic propagating sequences. *Neurogastroenterol Motil* **18**, 37-44 (2006).

336. Diotallevi C, Fava F, Gobbetti M, Tuohy K. Healthy dietary patterns to reduce obesity-related metabolic disease: polyphenol-microbiome interactions unifying health effects across geography. *Curr Opin Clin Nutr Metab Care* **23**, 437-444 (2020).

337. Dirlik M*, et al.* Effect of ornithine on the ileal histology, nitric oxide production and lipid peroxidation in LPS-induced endotoxemia. *Acta Med Okayama* **57**, 117-122 (2003).

338. Dmitrovskiĭ AA, Ershov Iu V, Bykhovskiĭ V. [Stabilization and protection of an enzymatic system, participating in the transformation of beta-carotene into retinal, during its isolation]. *Prikl Biokhim Mikrobiol* **28**, 199-204 (1992).

339. do Nascimento Bonato Panizzon CP, de Miranda Neto MH, Ramalho FV, Longhini R, de Mello JCP, Zanoni JN. Ethyl Acetate Fraction from Trichilia catigua Confers Partial Neuroprotection in Components of the Enteric Innervation of the Jejunum in Diabetic Rats. *Cell Physiol Biochem* **53**, 76-86 (2019).

340. Dociu N, Cojocel C, Baumann K. Sex differences in nephrotoxic and gastrointestinal effects of phenylbutazone. *Toxicology* **54**, 163-175 (1989).

341. Dogan A, Celik I, Kaya MS. Antidiabetic properties of lyophilized extract of acorn (Quercus brantii Lindl.) on experimentally STZ-induced diabetic rats. *J Ethnopharmacol* **176**, 243-251 (2015).

342. Doguet F*, et al.* Changes in mesenteric vascular reactivity and inflammatory response after cardiopulmonary bypass in a rat model. *Ann Thorac Surg* **77**, 2130-2137; author reply 2137 (2004).

343. Dolmatova LS. [The effect of exogenous prostaglandins E1 and F2 alpha on the adenosine triphosphatase activity in the ileal mucosa of rats]. *Fiziol Zh SSSR Im I M Sechenova* **76**, 778-781 (1990).

344. Dolmatova LS. [The effect of indomethacin on the activity of Na+,K+- and HCO3--ATPases and prostaglandin levels in the rat ileum mucosa after exposure to cholera exotoxin]. *Vopr Med Khim* **36**, 18-19 (1990).

345. Domagała-Rodacka R, Cibor D, Szczeklik K, Rodacki T, Mach T, Owczarek D. Gastrointestinal tract as a side-effect target of medications. *Przegl Lek* **73**, 652-658 (2016).

346. Dong P, Tao Y, Yang Y, Wang W. Expression of retinoic acid receptors in intestinal mucosa and the effect of vitamin A on mucosal immunity. *Nutrition* **26**, 740-745 (2010).

347. Doo E, Krishnamurthy GT, Eklem MJ, Gilbert S, Brown PH. Quantification of hepatobiliary function as an integral part of imaging with technetium-99m-mebrofenin in health and disease. *J Nucl Med* **32**, 48-57 (1991).

348. Dooley CP, Mello WD, Valenzuela JE. Effects of aspirin and prostaglandin E2 on interdigestive motility complex and duodenogastric reflux in man. *Dig Dis Sci* **30**, 513-521 (1985).

349. Dos Santos S, Lioté F. Osteoarticular manifestations of celiac disease and non-celiac gluten hypersensitivity. *Joint Bone Spine* **84**, 263-266 (2017).

350. Downard CD, Matheson PJ, Shepherd JA, Maki AC, Garrison RN. Direct peritoneal resuscitation augments ileal blood flow in necrotizing enterocolitis via a novel mechanism. *J Pediatr Surg* **47**, 1128-1134 (2012).

351. Drabińska N, Jarocka-Cyrta E, Ratcliffe NM, Krupa-Kozak U. The Profile of Urinary Headspace Volatile Organic Compounds After 12-Week Intake of Oligofructose-Enriched Inulin by Children and Adolescents with Celiac Disease on a Gluten-Free Diet: Results of a Pilot, Randomized, Placebo-Controlled Clinical Trial. *Molecules* **24**, (2019).

352. Dragoni S, Gee J, Bennett R, Valoti M, Sgaragli G. Red wine alcohol promotes quercetin absorption and directs its metabolism towards isorhamnetin and tamarixetin in rat intestine in vitro. *Br J Pharmacol* **147**, 765-771 (2006).

353. Dressler J, Hör G, Buttermann G, Pabst HW. [Diagnosis of pancreatic function by the selenomethionine--75Se test]. *MMW Munch Med Wochenschr* **117**, 237-240 (1975).

354. Dridi I*, et al.* Gastrointestinal toxicity of mycophenolate mofetil in rats: Effect of administration time. *Chronobiol Int* **32**, 1373-1384 (2015).

355. Drozdowski L, Iordache C, Clandinin MT, Wild G, Todd Z, Thomson AB. Dexamethasone and GLP-2 given to lactating rat dams influence glucose uptake in suckling and postweanling offspring. *JPEN J Parenter Enteral Nutr* **33**, 433-439 (2009).

356. Drozdowski LA*, et al.* Maternal dexamethasone and GLP-2 have early effects on intestinal sugar transport in their suckling rat offspring. *J Nutr Biochem* **20**, 771-782 (2009).

357. Du JF*, et al.* [Cloning, expression and functional analysis of CuZnSOD gene in swine.]. *Yi Chuan* **32**, 1037-1042 (2010).

358. Duane WC, Xiong W, Lofgren J. Transactivation of the human apical sodium-dependent bile acid transporter gene by human serum. *J Steroid Biochem Mol Biol* **108**, 137-148 (2008).

359. Dublineau I*, et al.* Modifications of inflammatory pathways in rat intestine following chronic ingestion of depleted uranium. *Toxicol Sci* **98**, 458-468 (2007).

360. DuPont HL. Review article: the antimicrobial effects of rifaximin on the gut microbiota. *Aliment Pharmacol Ther* **43 Suppl 1**, 3-10 (2016).

361. During A, Dawson HD, Harrison EH. Carotenoid transport is decreased and expression of the lipid transporters SR-BI, NPC1L1, and ABCA1 is downregulated in Caco-2 cells treated with ezetimibe. *J Nutr* **135**, 2305-2312 (2005).

362. Duszka C, Grolier P, Azim EM, Alexandre-Gouabau MC, Borel P, Azais-Braesco V. Rat intestinal beta-carotene dioxygenase activity is located primarily in the cytosol of mature jejunal enterocytes. *J Nutr* **126**, 2550-2556 (1996).

363. Echeverría F, Ortiz M, Valenzuela R, Videla LA. Hydroxytyrosol and Cytoprotection: A Projection for Clinical Interventions. *Int J Mol Sci* **18**, (2017).

364. Eckmann L*, et al.* Nitric oxide production by human intestinal epithelial cells and competition for arginine as potential determinants of host defense against the lumen-dwelling pathogen Giardia lamblia. *J Immunol* **164**, 1478-1487 (2000).

365. Ehrenpreis S, Greenberg J, Comaty JE. Block of electrically induced contractions of guinea pig longitudinal muscle by prostaglandin synthetase and receptor inhibitors. *Eur J Pharmacol* **39**, 331-340 (1976).

366. Ekblad E, Sundler F. Motor responses in rat ileum evoked by nitric oxide donors vs. field stimulation: modulation by pituitary adenylate cyclase-activating peptide, forskolin and guanylate cyclase inhibitors. *J Pharmacol Exp Ther* **283**, 23-28 (1997).

367. el Saghir NS*, et al.* Combination chemotherapy for primary small intestinal lymphoma in the Middle East. *Eur J Cancer Clin Oncol* **25**, 851-856 (1989).

368. El-Yazbi AF, Cho WJ, Boddy G, Schulz R, Daniel EE. Impact of caveolin-1 knockout on NANC relaxation in circular muscles of the mouse small intestine compared with longitudinal muscles. *Am J Physiol Gastrointest Liver Physiol* **290**, G394-403 (2006).

369. El-Zaatari M, Kao JY. Role of Dietary Metabolites in Regulating the Host Immune Response in Gastrointestinal Disease. *Front Immunol* **8**, 51 (2017).

370. Elliott G*, et al.* Effect of 16,16-dimethyl PGE2 on renal papillary necrosis and gastrointestinal ulcerations (gastric, duodenal, intestinal) produced in rats by mefenamic acid. *Life Sci* **39**, 423-432 (1986).

371. Ellison CA, Natuik SA, McIntosh AR, Scully SA, Danilenko DM, Gartner JG. The role of interferon-gamma, nitric oxide and lipopolysaccharide in intestinal graft-versus-host disease developing in F1-hybrid mice. *Immunology* **109**, 440-449 (2003).

372. Elsasser TH, Kahl S, MacLeod C, Nicholson B, Sartin JL, Li C. Mechanisms underlying growth hormone effects in augmenting nitric oxide production and protein tyrosine nitration during endotoxin challenge. *Endocrinology* **145**, 3413-3423 (2004).

373. Elvas L, Areia M, Brito D, Alves S, Saraiva S, Cadime AT. Premedication with simethicone and N-acetylcysteine in improving visibility during upper endoscopy: a double-blind randomized trial. *Endoscopy* **49**, 139-145 (2017).

374. Emani R*, et al.* Casein hydrolysate diet controls intestinal T cell activation, free radical production and microbial colonisation in NOD mice. *Diabetologia* **56**, 1781-1791 (2013).

375. Empey LR, Papp JD, Jewell LD, Fedorak RN. Mucosal protective effects of vitamin E and misoprostol during acute radiation-induced enteritis in rats. *Dig Dis Sci* **37**, 205-214 (1992).

376. Epriliati I, D'Arcy B, Gidley M. Nutriomic analysis of fresh and processed fruit products. 2. During in vitro simultaneous molecular passages using Caco-2 cell monolayers. *J Agric Food Chem* **57**, 3377-3388 (2009).

377. Erbil Y*, et al.* The effect of glutamine on radiation-induced organ damage. *Life Sci* **78**, 376-382 (2005).

378. Erdem T*, et al.* Vitamin and mineral deficiency in children newly diagnosed with celiac disease. *Turk J Med Sci* **45**, 833-836 (2015).

379. Eremina E, Bondarenko VM, Zvereva SI, Nikitina OI, Shaposhnikova LI. [Dysbiotic manifestations during eradication therapy of Helicobacter pylori and their corrections]. *Zh Mikrobiol Epidemiol Immunobiol*, 62-66 (2008).

380. Erickson RA, Bezabah S, Jonas G, Lifrak E, Tarnawski AS. Chronic omeprazole treatment increases duodenal susceptibility to ethanol injury in rats. *Dig Dis Sci* **36**, 897-904 (1991).

381. Erickson RA, Rivera N. Effect of difluoromethylornithine (DFMO) on NSAID-induced intestinal injury in rats. *Dig Dis Sci* **37**, 1833-1839 (1992).

382. Eriksson KE, Yang T, Carlström M, Weitzberg E. Organ uptake and release of inorganic nitrate and nitrite in the pig. *Nitric Oxide* **75**, 16-26 (2018).

383. Erkelens MN*, et al.* Intestinal Macrophages Balance Inflammatory Expression Profiles via Vitamin A and Dectin-1-Mediated Signaling. *Front Immunol* **11**, 551 (2020).

384. Eskandari MK, Kalff JC, Billiar TR, Lee KK, Bauer AJ. LPS-induced muscularis macrophage nitric oxide suppresses rat jejunal circular muscle activity. *Am J Physiol* **277**, G478-486 (1999).

385. Espinoza A, Morales S, Arredondo M. Effects of acute dietary iron overload in pigs (Sus scrofa) with induced type 2 diabetes mellitus. *Biol Trace Elem Res* **158**, 342-352 (2014).

386. Fabian MA, Bollinger RR, Wyble CW, Watkins WD, Canada AT. Evaluation of solutions for small intestinal preservation. Biochemical changes as a function of storage time. *Transplantation* **52**, 794-799 (1991).

387. Fagerholm U, Johansson M, Lennernäs H. Comparison between permeability coefficients in rat and human jejunum. *Pharm Res* **13**, 1336-1342 (1996).

388. Fagundes DS*, et al.* Mechanism of action of Trolox on duodenal contractility. *J Physiol Pharmacol* **64**, 705-710 (2013).

389. Falzon M, Milton AS, Burke MD. Are the decreases in hepatic cytochrome P-450 and other drug-metabolising enzymes caused by indomethacin in vivo mediated by intestinal bacterial endotoxins? 16,16-Dimethylprostaglandin F2 alpha prevents decreases in hepatic drug-metabolising enzymes due to exogenous endotoxin. *Biochem Pharmacol* **33**, 1285-1292 (1984).

390. Fan K, Kurihara N, Abe S, Ho CT, Ghai G, Yang K. Chemopreventive effects of orange peel extract (OPE). I: OPE inhibits intestinal tumor growth in ApcMin/+ mice. *J Med Food* **10**, 11-17 (2007).

391. Fan P, Li L, Rezaei A, Eslamfam S, Che D, Ma X. Metabolites of Dietary Protein and Peptides by Intestinal Microbes and their Impacts on Gut. *Curr Protein Pept Sci* **16**, 646-654 (2015).

392. Fan WQ, Smolich JJ, Wild J, Yu VY, Walker AM. Nitric oxide modulates regional blood flow differences in the fetal gastrointestinal tract. *Am J Physiol* **271**, G598-604 (1996).

393. Fändriks L, von Bothmer C, Johansson B, Holm M, Bölin I, Pettersson A. Water extract of Helicobacter pylori inhibits duodenal mucosal alkaline secretion in anesthetized rats. *Gastroenterology* **113**, 1570-1575 (1997).

394. Fang J. Classification of fruits based on anthocyanin types and relevance to their health effects. *Nutrition* **31**, 1301-1306 (2015).

395. Fang W, Xue H, Chen X, Chen K, Ling W. Supplementation with Sodium Butyrate Modulates the Composition of the Gut Microbiota and Ameliorates High-Fat Diet-Induced Obesity in Mice. *J Nutr* **149**, 747-754 (2019).

396. Farah MH, Samuelsson G. Pharmacologically active phenylpropanoids from Senra incana. *Planta Med* **58**, 14-18 (1992).

397. Farnetti S, Zocco MA, Garcovich M, Gasbarrini A, Capristo E. Functional and metabolic disorders in celiac disease: new implications for nutritional treatment. *J Med Food* **17**, 1159-1164 (2014).

398. Faseleh Jahromi M*, et al.* Dietary supplementation of a mixture of Lactobacillus strains enhances performance of broiler chickens raised under heat stress conditions. *Int J Biometeorol* **60**, 1099-1110 (2016).

399. Fatehi M, Tanomand A, Fatehi-Hassanabad Z. Effects of endotoxin-induced shock on withdrawal contractions in the Guinea-pig isolated ileum. *Clin Exp Pharmacol Physiol* **29**, 608-611 (2002).

400. Fatehi-Hassanabad Z, Fatehi M. Characterisation of some pharmacological effects of the venom from Vipera lebetina. *Toxicon* **43**, 385-391 (2004).

401. Faure M*, et al.* The chronic colitis developed by HLA-B27 transgenic rats is associated with altered in vivo mucin synthesis. *Dig Dis Sci* **49**, 339-346 (2004).

402. Favus MJ, Kimberg DV, Millar GN, Gershon E. Effects of cortisone administration on the metabolism and localization of 25-hydroxycholecalciferol in the rat. *J Clin Invest* **52**, 1328-1335 (1973).

403. Favus MJ, Walling MW, Kimberg DV. Effects of 1,25-dihydroxycholecalciferol on intestinal calcium transport in cortisone-treated rats. *J Clin Invest* **52**, 1680-1685 (1973).

404. Feher JJ, Wasserman RH. Intestinal calcium-binding protein and calcium absorption in cortisol-treated chicks: effects of vitamin D3 and 1,25-dihydroxyvitamin D3. *Endocrinology* **104**, 547-551 (1979).

405. Feigen LP, King LW, Ray J, Beckett W, Kadowitz PJ. Differential effects of ibuprofen and indomethacin in the regional circulation of the dog. *J Pharmacol Exp Ther* **219**, 679-684 (1981).

406. Fein M, Fuchs KH, Stopper H, Diem S, Herderich M. Duodenogastric reflux and foregut carcinogenesis: analysis of duodenal juice in a rodent model of cancer. *Carcinogenesis* **21**, 2079-2084 (2000).

407. Feng Y*, et al.* Ulva prolifera Extract Alleviates Intestinal Oxidative Stress via Nrf2 Signaling in Weaned Piglets Challenged With Hydrogen Peroxide. *Front Immunol* **11**, 599735 (2020).

408. Ferencz A*, et al.* Changes and effect of PACAP-38 on intestinal ischemia-reperfusion and autotransplantation. *Transplant Proc* **41**, 57-59 (2009).

409. Fernandez SC, Budowski P, Ascarelli I, Neumark H, Bondi A. Low utilization of carotene by sheep. *Int J Vitam Nutr Res* **46**, 446-453 (1976).

410. Ferreira SH, Herman AG, Vane JR. Prostaglandin production by rabbit isolated jejunum and its relationship to the inherent tone of the preparation. *Br J Pharmacol* **56**, 469-477 (1976).

411. Ferreira TM*, et al.* Oral supplementation of butyrate reduces mucositis and intestinal permeability associated with 5-Fluorouracil administration. *Lipids* **47**, 669-678 (2012).

412. Ferretti G, Bacchetti T, Masciangelo S, Saturni L. Celiac disease, inflammation and oxidative damage: a nutrigenetic approach. *Nutrients* **4**, 243-257 (2012).

413. Figini M*, et al.* Substance P and bradykinin stimulate plasma extravasation in the mouse gastrointestinal tract and pancreas. *Am J Physiol* **272**, G785-793 (1997).

414. Filpa V*, et al.* Interaction between NMDA glutamatergic and nitrergic enteric pathways during in vitro ischemia and reperfusion. *Eur J Pharmacol* **750**, 123-131 (2015).

415. Fink MP, Kaups KL, Wang H, Rothschild HR. Ibuprofen improves survival but does not ameliorate increased gut mucosal permeability in endotoxic pigs. *Arch Surg* **127**, 49-53; discussion 53-44 (1992).

416. Fink MP, Morrissey PE, Stein KL, Clement RE, Fiallo V, Gardiner WM. Systemic and regional hemodynamic effects of cyclo-oxygenase and thromboxane synthetase inhibition in normal and hyperdynamic endotoxemic rabbits. *Circ Shock* **26**, 41-57 (1988).

417. Fiorentini MT*, et al.* Physiology of ileoanal anastomosis with ileal reservoir for ulcerative colitis and adenomatosis coli. *Dis Colon Rectum* **30**, 267-272 (1987).

418. Fiorucci S*, et al.* Co-administration of nitric oxide-aspirin (NCX-4016) and aspirin prevents platelet and monocyte activation and protects against gastric damage induced by aspirin in humans. *J Am Coll Cardiol* **44**, 635-641 (2004).

419. Fischer CD, Beatty JK, Zvaigzne CG, Morck DW, Lucas MJ, Buret AG. Anti-Inflammatory benefits of antibiotic-induced neutrophil apoptosis: tulathromycin induces caspase-3-dependent neutrophil programmed cell death and inhibits NF-kappaB signaling and CXCL8 transcription. *Antimicrob Agents Chemother* **55**, 338-348 (2011).

420. FitzGerald GA, Lupinetti M, Charman SA, Charman WN. Presystemic acetylation of platelets by aspirin: reduction in rate of drug delivery to improve biochemical selectivity for thromboxane A2. *J Pharmacol Exp Ther* **259**, 1043-1049 (1991).

421. Fleisch JH*, et al.* Pharmacologic analysis of two novel inhibitors of leukotriene (slow reacting substance) release. *J Pharmacol Exp Ther* **229**, 681-689 (1984).

422. Flemström G. Stimulation of HCO3- transport in isolated proximal bullfrog duodenum by prostaglandins. *Am J Physiol* **239**, G198-203 (1980).

423. Flemström G, Bengtsson MW, Mäkelä K, Herzig KH. Effects of short-term food deprivation on orexin-A-induced intestinal bicarbonate secretion in comparison with related secretagogues. *Acta Physiol (Oxf)* **198**, 373-380 (2010).

424. Flemström G, Bergman A, Bridén S. Stimulation of mucosal bicarbonate secretion in rat duodenum in vivo by BW755C. *Acta Physiol Scand* **121**, 39-43 (1984).

425. Flemström G, Garner A, Nylander O, Hurst BC, Heylings JR. Surface epithelial HCO3(-) transport by mammalian duodenum in vivo. *Am J Physiol* **243**, G348-358 (1982).

426. Flemström G, Kivilaakso E. Demonstration of a pH gradient at the luminal surface of rat duodenum in vivo and its dependence on mucosal alkaline secretion. *Gastroenterology* **84**, 787-794 (1983).

427. Flemström G, Nylander O. Stimulation of duodenal epithelial HCO3- transport in the guinea pig and cat by luminal prostaglandin E2. *Prostaglandins* **21 Suppl**, 47-52 (1981).

428. Flynn NE, Wu G. Enhanced metabolism of arginine and glutamine in enterocytes of cortisol-treated pigs. *Am J Physiol* **272**, G474-480 (1997).

429. Flynn NE, Wu G. Glucocorticoids play an important role in mediating the enhanced metabolism of arginine and glutamine in enterocytes of postweaning pigs. *J Nutr* **127**, 732-737 (1997).

430. Fodor I, Marx JJ. Lipid peroxidation of rabbit small intestinal microvillus membrane vesicles by iron complexes. *Biochim Biophys Acta* **961**, 96-102 (1988).

431. Foltzer-Jourdainne C, Garaud JC, Nsi-Emvo E, Raul F. Epidermal growth factor and the maturation of intestinal sucrase in suckling rats. *Am J Physiol* **265**, G459-466 (1993).

432. Foltzer-Jourdainne C, Kedinger M, Raul F. Perinatal expression of brush-border hydrolases in rat colon: hormonal and tissue regulations. *Am J Physiol* **257**, G496-503 (1989).

433. Foltzer-Jourdainne C, Raul F. Effect of epidermal growth factor on the expression of digestive hydrolases in the jejunum and colon of newborn rats. *Endocrinology* **127**, 1763-1769 (1990).

434. Forester SC, Gu Y, Lambert JD. Inhibition of starch digestion by the green tea polyphenol, (-)-epigallocatechin-3-gallate. *Mol Nutr Food Res* **56**, 1647-1654 (2012).

435. Förstermann U, Neufang B. C-6-sulfidopeptide leukotrienes are unlikely to be involved in the endothelium dependent relaxation of rabbit aorta by acetylcholine. *Prostaglandins* **27**, 181-193 (1984).

436. Forsyth CB*, et al.* Alcohol Feeding in Mice Promotes Colonic Hyperpermeability and Changes in Colonic Organoid Stem Cell Fate. *Alcohol Clin Exp Res* **41**, 2100-2113 (2017).

437. Foureaux Rde C*, et al.* Effects of probiotic therapy on metabolic and inflammatory parameters of rats with ligature-induced periodontitis associated with restraint stress. *J Periodontol* **85**, 975-983 (2014).

438. Fournel A*, et al.* Apelin targets gut contraction to control glucose metabolism via the brain. *Gut* **66**, 258-269 (2017).

439. Fousekis FS, Beka ET, Mitselos IV, Milionis H, Christodoulou DK. Thromboembolic complications and cardiovascular events associated with celiac disease. *Ir J Med Sci*, (2020).

440. Fox J, Ross R, Care AD. Effects of acute and chronic treatment with glucocorticoids on the intestinal absorption of calcium and phosphate and on plasma 1,25-dihydroxyvitamin D levels in pigs. *Clin Sci (Lond)* **69**, 553-559 (1985).

441. Fox-Threlkeld JE, Woskowska Z, Daniel EE. Sites of nitric oxide (NO) actions in control of circular muscle motility of the perfused isolated canine ileum. *Can J Physiol Pharmacol* **75**, 1340-1349 (1997).

442. Fracaro L*, et al.* Walker 256 tumor-bearing rats demonstrate altered interstitial cells of Cajal. Effects on ICC in the Walker 256 tumor model. *Neurogastroenterol Motil* **28**, 101-115 (2016).

443. Francavilla R, Cristofori F, Stella M, Borrelli G, Naspi G, Castellaneta S. Treatment of celiac disease: from gluten-free diet to novel therapies. *Minerva Pediatr* **66**, 501-516 (2014).

444. Franchi-Micheli S, Luzzi S, Ciuffi M, Zilletti L. The effect of lipoxygenase inhibitors and leukotriene antagonists on anaphylaxis. *Agents Actions* **18**, 242-244 (1986).

445. Franco F, Snart RS. Role of cyclic AMP in steroid action in rat intestine. *Biochim Biophys Acta* **970**, 287-291 (1988).

446. Franklin MA, Mathew AG, Vickers JR, Clift RA. Characterization of microbial populations and volatile fatty acid concentrations in the jejunum, ileum, and cecum of pigs weaned at 17 vs 24 days of age. *J Anim Sci* **80**, 2904-2910 (2002).

447. Franks CM, Hardcastle J, Hardcastle PT. Involvement of nitric oxide in the response to 5-hydroxytryptamine in the rat in-vivo. *J Pharm Pharmacol* **46**, 387-389 (1994).

448. Freerksen N*, et al.* PAR-2 activating peptide-induced stimulation of pregnant rat myometrium contractile activity partly involves the other membrane receptors. *Eur J Obstet Gynecol Reprod Biol* **130**, 51-59 (2007).

449. Fricker M*, et al.* Chronic cigarette smoke exposure induces systemic hypoxia that drives intestinal dysfunction. *JCI Insight* **3**, (2018).

450. Friedman H, Seckman CE, Schwartz JH, Lanza FL, Royer GL, Stubbs CM. The effects of flurbiprofen, aspirin, cimetidine, and antacids on the gastric and duodenal mucosa of normal volunteers. An endoscopic and photographic study. *J Clin Pharmacol* **29**, 559-562 (1989).

451. Fu H*, et al.* Persistent cholestasis resulting from duodenal papillary carcinoma in an adolescent male: A case report. *Medicine (Baltimore)* **98**, e15708 (2019).

452. Fu TL, Zhang WT, Chen QP, Gao Y, Hu YH, Zhang DL. Effects of L-arginine on serum nitric oxide, nitric oxide synthase and mucosal Na+-K+-ATPase and nitric oxide synthase activity in segmental small-bowel autotransplantation model. *World J Gastroenterol* **11**, 3605-3609 (2005).

453. Fujimoto S, Mori M, Tsushima H, Kunimatsu M. Capsaicin-induced, capsazepine-insensitive relaxation of the guinea-pig ileum. *Eur J Pharmacol* **530**, 144-151 (2006).

454. Fujimoto W*, et al.* Niflumic acid-sensitive ion channels play an important role in the induction of glucose-stimulated insulin secretion by cyclic AMP in mice. *Diabetologia* **52**, 863-872 (2009).

455. Fujiwara T, Kawarasaki H, Fonkalsrud EW. Endorectal ileal pullthrough procedure after chemical debridement of the rectal mucosa. *Surg Gynecol Obstet* **158**, 437-442 (1984).

456. Fukui E, Kurohara H, Kageyu A, Kurosaki Y, Nakayama T, Kimura T. Enhancing effect of medium-chain triglycerides on intestinal absorption of d-alpha-tocopherol acetate from lecithin-dispersed preparations in the rat. *J Pharmacobiodyn* **12**, 80-86 (1989).

457. Fukunaga Y, Mine Y, Yoshikawa S, Takeuchi T, Hata F, Yagasaki O. Role of prostacyclin in acetylcholine release from myenteric plexus of guinea-pig ileum. *Eur J Pharmacol* **233**, 237-242 (1993).

458. Fukunaka N, Sagae S, Kudo R, Endo T, Hirafuji M, Minami M. Effects of granisetron and its combination with dexamethasone on cisplatin-induced delayed emesis in the ferret. *Gen Pharmacol* **31**, 775-781 (1998).

459. Funakoshi A, Tateishi K, Shinozaki H, Miyasaka K, Ito T, Wakasugi H. Plasma pancreastatin responses after intrajejunal infusion of liquid meal in patients with chronic pancreatitis. *Dig Dis Sci* **35**, 721-725 (1990).

460. Funk C, Weber P, Thilker J, Grabber JH, Steinhart H, Bunzel M. Influence of lignification and feruloylation of maize cell walls on the adsorption of heterocyclic aromatic amines. *J Agric Food Chem* **54**, 1860-1867 (2006).

461. Furukawa O, Kawauchi S, Mimaki H, Takeuchi K. Stimulation by nitric oxide of HCO3- secretion in bullfrog duodenum in vitro--roles of cyclooxygenase-1 and prostaglandins. *Med Sci Monit* **6**, 454-459 (2000).

462. Furukawa O, Kitamura M, Sugamoto S, Takeuchi K. Stimulatory effect of nitric oxide on bicarbonate secretion in bullfrog duodenums in vitro. *Digestion* **60**, 324-331 (1999).

463. Gaboriau-Routhiau V*, et al.* The key role of segmented filamentous bacteria in the coordinated maturation of gut helper T cell responses. *Immunity* **31**, 677-689 (2009).

464. Gabra BH, Afify EA, Daabees TT, Abou Zeit-Har MS. The role of the NO/NMDA pathways in the development of morphine withdrawal induced by naloxone in vitro. *Pharmacol Res* **51**, 319-327 (2005).

465. Gadaleta RM*, et al.* Farnesoid X receptor activation inhibits inflammation and preserves the intestinal barrier in inflammatory bowel disease. *Gut* **60**, 463-472 (2011).

466. Gaion RM, Gambarotto L. Target sites for the inhibition of prostacyclin effect in guinea-pig ileum. *Naunyn Schmiedebergs Arch Pharmacol* **336**, 445-452 (1987).

467. Gallavan RH, Jr., Chou CC. Prostaglandin synthesis inhibition and postprandial intestinal hyperemia. *Am J Physiol* **242**, G140-146 (1982).

468. Gallavan RH, Jr., Chou CC. The effects of mefenamic acid on postprandial intestinal carbohydrate metabolism. *Prostaglandins* **31**, 1069-1076 (1986).

469. Ganapathy V*, et al.* Sodium-coupled monocarboxylate transporters in normal tissues and in cancer. *Aaps j* **10**, 193-199 (2008).

470. Gangadaran S, Cheema SK. A high fat diet enriched with sea cucumber gut powder provides cardio-protective and anti-obesity effects in C57BL/6 mice. *Food Res Int* **99**, 799-806 (2017).

471. Gao F, Horie T. A synthetic analog of prostaglandin E(1) prevents the production of reactive oxygen species in the intestinal mucosa of methotrexate-treated rats. *Life Sci* **71**, 1091-1099 (2002).

472. Gao JH*, et al.* Inhibition of cyclooxygenase-2 alleviates liver cirrhosis via improvement of the dysfunctional gut-liver axis in rats. *Am J Physiol Gastrointest Liver Physiol* **310**, G962-972 (2016).

473. Gao Y*, et al.* Effects of titanium dioxide nanoparticles on nutrient absorption and metabolism in rats: distinguishing the susceptibility of amino acids, metal elements, and glucose. *Nanotoxicology* **14**, 1301-1323 (2020).

474. García-Barrios A*, et al.* Nitric oxide involved in the IL-1β-induced inhibition of fructose intestinal transport. *J Cell Biochem* **111**, 1321-1329 (2010).

475. Gardiner K, Barbul A. The role of the imino transporter protein in sepsis-impaired intestinal proline absorption. *JPEN J Parenter Enteral Nutr* **17**, 507-512 (1993).

476. Gardiner KR, Gardiner RE, Barbul A. Reduced intestinal absorption of arginine during sepsis. *Crit Care Med* **23**, 1227-1232 (1995).

477. Gardiner PJ. Characterization of prostanoid relaxant/inhibitory receptors (psi) using a highly selective agonist, TR4979. *Br J Pharmacol* **87**, 45-56 (1986).

478. Garrait G, Jarrige JF, Blanquet S, Beyssac E, Alric M. Recombinant Saccharomyces cerevisiae strain expressing a model cytochrome P450 in the rat digestive environment: viability and bioconversion activity. *Appl Environ Microbiol* **73**, 3566-3574 (2007).

479. Generoso Sde V*, et al.* Dietary supplementation with omega-3 fatty acid attenuates 5-fluorouracil induced mucositis in mice. *Lipids Health Dis* **14**, 54 (2015).

480. Geor RJ, Petrie L, Papich MG, Rousseaux C. The protective effects of sucralfate and ranitidine in foals experimentally intoxicated with phenylbutazone. *Can J Vet Res* **53**, 231-238 (1989).

481. George F*, et al.* Occurrence and Dynamism of Lactic Acid Bacteria in Distinct Ecological Niches: A Multifaceted Functional Health Perspective. *Front Microbiol* **9**, 2899 (2018).

482. Gerber M, Walch C, Löffler B, Tischendorf K, Reischl U, Ackermann G. Effect of sub-MIC concentrations of metronidazole, vancomycin, clindamycin and linezolid on toxin gene transcription and production in Clostridium difficile. *J Med Microbiol* **57**, 776-783 (2008).

483. Gerson CD, Lowe EH, Lindenbaum J. Bioavailability of digoxin tablets in patients with gastrointestinal dysfunction. *Am J Med* **69**, 43-49 (1980).

484. Gessell-Lee DL, Popov VL, Boldogh I, Olano JP, Peterson JW. Role of cyclooxygenase enzymes in a murine model of experimental cholera. *Infect Immun* **71**, 6234-6242 (2003).

485. Gessner DK*, et al.* Supplementation of a grape seed and grape marc meal extract decreases activities of the oxidative stress-responsive transcription factors NF-κB and Nrf2 in the duodenal mucosa of pigs. *Acta Vet Scand* **55**, 18 (2013).

486. Ghorbani A. Mechanisms of antidiabetic effects of flavonoid rutin. *Biomed Pharmacother* **96**, 305-312 (2017).

487. Ghosal A, Lambrecht N, Subramanya SB, Kapadia R, Said HM. Conditional knockout of the Slc5a6 gene in mouse intestine impairs biotin absorption. *Am J Physiol Gastrointest Liver Physiol* **304**, G64-71 (2013).

488. Gianello R, Hall WC, Kennepohl E, Libinaki R, Ogru E. Subchronic oral toxicity study of mixed tocopheryl phosphates in rats. *Int J Toxicol* **26**, 475-490 (2007).

489. Gibbs MA. Ascorbic acid use in hyporesponders to Epoetin alfa. *Nephrol Nurs J* **27**, 413-415 (2000).

490. Gil-Cardoso K, Ginés I, Pinent M, Ardévol A, Terra X, Blay M. A cafeteria diet triggers intestinal inflammation and oxidative stress in obese rats. *Br J Nutr* **117**, 218-229 (2017).

491. Gil-Iturbe E*, et al.* Effect of aging and obesity on GLUT12 expression in small intestine, adipose tissue, muscle, and kidney and its regulation by docosahexaenoic acid and exercise in mice. *Appl Physiol Nutr Metab* **45**, 957-967 (2020).

492. Gillin JS*, et al.* Malabsorption and mucosal abnormalities of the small intestine in the acquired immunodeficiency syndrome. *Ann Intern Med* **102**, 619-622 (1985).

493. Gillum MP*, et al.* N-acylphosphatidylethanolamine, a gut- derived circulating factor induced by fat ingestion, inhibits food intake. *Cell* **135**, 813-824 (2008).

494. Gisbert-González SL, Torres-Molina F. Zinc uptake in five sectors of the rat gastrointestinal tract: kinetic study in the whole colon. *Pharm Res* **13**, 1154-1161 (1996).

495. Glasgow I, Mattar K, Krantis A. Rat gastroduodenal motility in vivo: involvement of NO and ATP in spontaneous motor activity. *Am J Physiol* **275**, G889-896 (1998).

496. Gmoshinskiĭ IV, Mazo VK. [Mineral substance in human nutrition. Selenium: absorption and bioavailability]. *Vopr Pitan* **75**, 15-21 (2006).

497. Gobbetti T*, et al.* Serine protease inhibition reduces post-ischemic granulocyte recruitment in mouse intestine. *Am J Pathol* **180**, 141-152 (2012).

498. Gobbetti T*, et al.* Polyunsaturated fatty acid metabolism signature in ischemia differs from reperfusion in mouse intestine. *PLoS One* **8**, e75581 (2013).

499. Göcer F, Yaris E, Tuncer M. The action of amyl nitrite and isosorbide dinitrate on the contractility of sphincter of Oddi of guinea-pigs. *Gen Pharmacol* **25**, 995-999 (1994).

500. Göçer F, Yariş E, Tuncer M. The effects of PGE2 and PGF2 alpha on rhythmic contractions of sphincter of Oddi. *Gen Pharmacol* **26**, 1397-1401 (1995).

501. Goerg KJ, Wanitschke R, Becker U, Meyer zum Büschenfelde KH. Effect of the stable prostacyclin analogue iloprost on water and electrolyte transfer of the rat ileum and colon in vivo. *Eur J Clin Invest* **18**, 124-127 (1988).

502. Goerg KJ, Wanitschke R, Diehl PH, Meyer zum Büschenfelde KH. Secretory effect of azodisalicylate (azodisal sodium) on the short circuited mucosa of the rat ileum in vitro. *Gut* **29**, 336-341 (1988).

503. Goldhill JM, Finkelman FD, Morris SC, Shea-Donohue T. Neural control of mouse small intestinal longitudinal muscle: interactions with inflammatory mediators. *J Pharmacol Exp Ther* **274**, 72-77 (1995).

504. Goldhill JM, Stojadinovic A, Kiang J, Smallridge R, Shea-Donohue T. Hyperthermia prevents functional, histological and biochemical abnormalities induced during ileitis. *Neurogastroenterol Motil* **11**, 69-76 (1999).

505. Goldin E*, et al.* No correlation between indomethacin-induced gastroduodenal damage and inhibition of gastric prostanoid synthesis. *Aliment Pharmacol Ther* **2**, 369-375 (1988).

506. Goldstein SR, Yang GY, Chen X, Curtis SK, Yang CS. Studies of iron deposits, inducible nitric oxide synthase and nitrotyrosine in a rat model for esophageal adenocarcinoma. *Carcinogenesis* **19**, 1445-1449 (1998).

507. Gong HZ*, et al.* Effects of laying breeder hens dietary β-carotene, curcumin, allicin, and sodium butyrate supplementation on the growth performance, immunity, and jejunum morphology of their offspring chicks. *Poult Sci* **99**, 151-162 (2020).

508. Goñi I, Serrano J, Saura-Calixto F. Bioaccessibility of beta-carotene, lutein, and lycopene from fruits and vegetables. *J Agric Food Chem* **54**, 5382-5387 (2006).

509. Gonzalez Alvarez R, Kazimierczak W. On the mechanism of suppressive action of some drugs influencing calcium ions on the histamine-induced contraction of guinea-pig ileum. *Pol J Pharmacol Pharm* **32**, 715-723 (1980).

510. Gonzalo S, Grasa L, Fagundes DS, Arruebo MP, Plaza M, Murillo MD. Intestinal effects of lipopolysaccharide in rabbit are mediated by cyclooxygenase-2 through p38 mitogen activated protein kinase. *Eur J Pharmacol* **648**, 171-178 (2010).

511. Gonzalo S*, et al.* Roles of Toll-Like Receptor 4, IκB Kinase, and the Proteasome in the Intestinal Alterations Caused by Sepsis. *Dig Dis Sci* **60**, 1223-1231 (2015).

512. Good M*, et al.* The human milk oligosaccharide 2'-fucosyllactose attenuates the severity of experimental necrotising enterocolitis by enhancing mesenteric perfusion in the neonatal intestine. *Br J Nutr* **116**, 1175-1187 (2016).

513. Gookin JL, Foster DM, Coccaro MR, Stauffer SH. Oral delivery of L-arginine stimulates prostaglandin-dependent secretory diarrhea in Cryptosporidium parvum-infected neonatal piglets. *J Pediatr Gastroenterol Nutr* **46**, 139-146 (2008).

514. Gookin JL, Rhoads JM, Argenzio RA. Inducible nitric oxide synthase mediates early epithelial repair of porcine ileum. *Am J Physiol Gastrointest Liver Physiol* **283**, G157-168 (2002).

515. Gookin JL, Stauffer SH, Stone MR. Induction of arginase II by intestinal epithelium promotes the uptake of L-arginine from the lumen of Cryptosporidium parvum-infected porcine ileum. *J Pediatr Gastroenterol Nutr* **47**, 417-427 (2008).

516. Goren I*, et al.* Starch Consumption May Modify Antiglycan Antibodies and Fecal Fungal Composition in Patients With Ileo-Anal Pouch. *Inflamm Bowel Dis* **25**, 742-749 (2019).

517. Goswami SK*, et al.* Anti-Ulcer Efficacy of Soluble Epoxide Hydrolase Inhibitor TPPU on Diclofenac-Induced Intestinal Ulcers. *J Pharmacol Exp Ther* **357**, 529-536 (2016).

518. Govendir M, Canfield PJ, Emslie DR, Watson AD, Church DB. Evaluation of d,l-ethionine as a mechanism for pancreatic islet regeneration in dogs. *Aust Vet J* **80**, 75-82; discussion 82 (2002).

519. Gowda SGB*, et al.* Identification of short-chain fatty acid esters of hydroxy fatty acids (SFAHFAs) in a murine model by nontargeted analysis using ultra-high-performance liquid chromatography/linear ion trap quadrupole-Orbitrap mass spectrometry. *Rapid Commun Mass Spectrom* **34**, e8831 (2020).

520. Grabinger T, Glaus Garzon JF, Hausmann M, Geirnaert A, Lacroix C, Hennet T. Alleviation of Intestinal Inflammation by Oral Supplementation With 2-Fucosyllactose in Mice. *Front Microbiol* **10**, 1385 (2019).

521. Graham JP, Boland JJ, Silbergeld E. Growth promoting antibiotics in food animal production: an economic analysis. *Public Health Rep* **122**, 79-87 (2007).

522. Graham MF, Drucker DE, Diegelmann RF, Elson CO. Collagen synthesis by human intestinal smooth muscle cells in culture. *Gastroenterology* **92**, 400-405 (1987).

523. Graham MF, Willey A, Adams J, Yager D, Diegelmann RF. Role of ascorbic acid in procollagen expression and secretion by human intestinal smooth muscle cells. *J Cell Physiol* **162**, 225-233 (1995).

524. Granstam SO, Flemström G, Nylander O. Bicarbonate secretion by the rabbit duodenum in vivo: effects of prostaglandins, vagal stimulation and some drugs. *Acta Physiol Scand* **131**, 377-385 (1987).

525. Grasa L, Arruebo MP, Plaza MA, Murillo MD. The role of tyrosine kinase in prostaglandin E2 and vanadate-evoked contractions in rabbit duodenum in vitro. *J Physiol Pharmacol* **57**, 279-289 (2006).

526. Grasa L, Rebollar E, Arruebo MP, Plaza MA, Murillo MD. The role of NO in the contractility of rabbit small intestine in vitro: effect of K+ channels. *J Physiol Pharmacol* **56**, 407-419 (2005).

527. Grattagliano I*, et al.* Effect of dietary restriction and N-acetylcysteine supplementation on intestinal mucosa and liver mitochondrial redox status and function in aged rats. *Exp Gerontol* **39**, 1323-1332 (2004).

528. Graves JP*, et al.* Quantitative Polymerase Chain Reaction Analysis of the Mouse Cyp2j Subfamily: Tissue Distribution and Regulation. *Drug Metab Dispos* **43**, 1169-1180 (2015).

529. Green RP, Birkenmeier EH, Beamer WG, Maltais LJ, Gordon JI. The hypothyroid (hyt/hyt) mouse: a model system for studying the effects of thyroid hormone on developmental changes in gene expression. *Proc Natl Acad Sci U S A* **85**, 5592-5596 (1988).

530. Grider JR, Jin JG. Vasoactive intestinal peptide release and L-citrulline production from isolated ganglia of the myenteric plexus: evidence for regulation of vasoactive intestinal peptide release by nitric oxide. *Neuroscience* **54**, 521-526 (1993).

531. Grider JR, Murthy KS. Autoinhibition of endothelial nitric oxide synthase (eNOS) in gut smooth muscle by nitric oxide. *Regul Pept* **151**, 75-79 (2008).

532. Griesbacher T. Kinin-induced relaxations of the rat duodenum. *Naunyn Schmiedebergs Arch Pharmacol* **346**, 102-107 (1992).

533. Griesbacher T, Sametz W, Legat FJ, Diethart S, Hammer S, Juan H. Effects of the non-peptide B2 antagonist FR173657 on kinin-induced smooth muscle contraction and relaxation, vasoconstriction and prostaglandin release. *Br J Pharmacol* **121**, 469-476 (1997).

534. Grimble GK. Adverse gastrointestinal effects of arginine and related amino acids. *J Nutr* **137**, 1693s-1701s (2007).

535. Gronwall R, Engelking LR. Effect of glucose administration on equine fasting hyperbilirubinemia. *Am J Vet Res* **43**, 801-803 (1982).

536. Gross Margolis K*, et al.* Enteric serotonin and oxytocin: endogenous regulation of severity in a murine model of necrotizing enterocolitis. *Am J Physiol Gastrointest Liver Physiol* **313**, G386-g398 (2017).

537. Grossi L, Falcucci M, Lapenna D, Marzio L. Effect of nitric oxide on propagated clusters of spontaneous motor waves in an ex vivo rabbit intestinal preparation. *Neurogastroenterol Motil* **8**, 201-205 (1996).

538. Gruber L*, et al.* High fat diet accelerates pathogenesis of murine Crohn's disease-like ileitis independently of obesity. *PLoS One* **8**, e71661 (2013).

539. Gruchlik A, Turek A, Polechoński J, Dzierżewicz Z. EFFECTS OF 300 mT STATIC MAGNETIC FIELD ON IL-8 SECRETION IN NORMAL HUMAN COLON MYOFIBROBLASTS. *Acta Pol Pharm* **72**, 713-717 (2015).

540. Grudziński IP, Frankiewicz-Jóźko A. Nitric oxide synthase inhibitors reduced lipid peroxidation in N-nitrosodiethylamine-treated rats. *Rocz Panstw Zakl Hig* **52**, 89-95 (2001).

541. Gu X*, et al.* Neutral ceramidase mediates nonalcoholic steatohepatitis by regulating monounsaturated fatty acids and gut IgA(+) B cells. *Hepatology*, (2020).

542. Gu XH, Hao Y, Wang XL. Overexpression of heat shock protein 70 and its relationship to intestine under acute heat stress in broilers: 2. Intestinal oxidative stress. *Poult Sci* **91**, 790-799 (2012).

543. Guandalini S*, et al.* Unconjugated bilirubin and the bile from light exposed Gunn rats inhibit intestinal water and electrolyte absorption. *Gut* **29**, 366-371 (1988).

544. Gubatan J, Moss AC. Vitamin D in inflammatory bowel disease: more than just a supplement. *Curr Opin Gastroenterol* **34**, 217-225 (2018).

545. Guerra DD, Bok R, Vyas V, Orlicky DJ, Lorca RA, Hurt KJ. Akt phosphorylation of neuronal nitric oxide synthase regulates gastrointestinal motility in mouse ileum. *Proc Natl Acad Sci U S A* **116**, 17541-17546 (2019).

546. Guida F*, et al.* Antibiotic-induced microbiota perturbation causes gut endocannabinoidome changes, hippocampal neuroglial reorganization and depression in mice. *Brain Behav Immun* **67**, 230-245 (2018).

547. Gulgun M*, et al.* Effect of proanthocyanidin, arginine and glutamine supplementation on methotrexate-induced gastrointestinal toxicity in rats. *Methods Find Exp Clin Pharmacol* **32**, 657-661 (2010).

548. Gultekin H, Erdem SR, Emre-Aydingoz S, Tuncer M. The role of nitric oxide in the electrical field stimulation-induced contractions of sphincter of oddi and gallbladder strips in Guinea pigs. *J Pharmacol Sci* **101**, 240-244 (2006).

549. Guo M*, et al.* Role of non-muscle myosin light chain kinase in neutrophil-mediated intestinal barrier dysfunction during thermal injury. *Shock* **38**, 436-443 (2012).

550. Guo S*, et al.* Anti-inflammatory effect of xanthomicrol, a major colonic metabolite of 5-demethyltangeretin. *Food Funct* **9**, 3104-3113 (2018).

551. Guo Y*, et al.* Dietary fat increases quercetin bioavailability in overweight adults. *Mol Nutr Food Res* **57**, 896-905 (2013).

552. Gurleyik E, Coskun O, Ustundag N, Ozturk E. Prostaglandin E1 maintains structural integrity of intestinal mucosa and prevents bacterial translocation during experimental obstructive jaundice. *J Invest Surg* **19**, 283-289 (2006).

553. Guschlbauer M, Klinger S, Burmester M, Horn J, Kulling SE, Breves G. trans-Resveratrol and ε-viniferin decrease glucose absorption in porcine jejunum and ileum in vitro. *Comp Biochem Physiol A Mol Integr Physiol* **165**, 313-318 (2013).

554. Gustafsson BI, Delbro DS. Neurogenic inhibition of duodenal and jejunal motility in the anaesthetized rat. *Eur J Pharmacol* **257**, 227-233 (1994).

555. Gustafsson L, Hedqvist P, Lundgren G. Pre- and postjunctional effects of prostaglandin E2, prostaglandin synthetase inhibitors and atropine on cholinergic neurotransmission in guinea pig ileum and bovine iris. *Acta Physiol Scand* **110**, 401-411 (1980).

556. Gustafsson LE, Wiklund CU, Wiklund NP, Persson MG, Moncada S. Modulation of autonomic neuroeffector transmission by nitric oxide in guinea pig ileum. *Biochem Biophys Res Commun* **173**, 106-110 (1990).

557. Guyan PM, Uden S, Braganza JM. Heightened free radical activity in pancreatitis. *Free Radic Biol Med* **8**, 347-354 (1990).

558. Haag M, Kearns SD, Magada ON, Mphata PR, Claassen N, Kruger MC. Effect of arachidonic acid on duodenal enterocyte ATPases. *Prostaglandins Other Lipid Mediat* **66**, 53-63 (2001).

559. Hagenlocher Y, Bergheim I, Zacheja S, Schäffer M, Bischoff SC, Lorentz A. Cinnamon extract inhibits degranulation and de novo synthesis of inflammatory mediators in mast cells. *Allergy* **68**, 490-497 (2013).

560. Haglind E, Malmlof K, Fan J, Lang CH. Insulin-like growth factor-I and growth hormone administration in intestinal ischemia shock in the rat. *Shock* **10**, 62-68 (1998).

561. Hahn M, Raithel M, Hagel A, Biermann T, Manger B. Chronic calcium pyrophosphate crystal inflammatory arthritis induced by extreme hypomagnesemia in short bowel syndrome. *BMC Gastroenterol* **12**, 129 (2012).

562. Haidry RJ*, et al.* Duodenal mucosal resurfacing: proof-of-concept, procedural development, and initial implementation in the clinical setting. *Gastrointest Endosc* **90**, 673-681.e672 (2019).

563. Hajjaji N, Besson P, Bougnoux P. Tumor and non-tumor tissues differential oxidative stress response to supplemental DHA and chemotherapy in rats. *Cancer Chemother Pharmacol* **70**, 17-23 (2012).

564. Halawa A, Dänicke S, Kersten S, Breves G. Effects of deoxynivalenol and lipopolysaccharide on electrophysiological parameters in growing pigs. *Mycotoxin Res* **28**, 243-252 (2012).

565. Hall AK, Bishop JE, Norman AW. Inhibitory and stimulatory effects of dexamethasone and 1,25-dihydroxyvitamin D3 on chick intestinal calbindin-D28K and its mRNA. *Mol Cell Endocrinol* **51**, 25-31 (1987).

566. Hall WJ, O'Neill P, Sheehan JD. The role of prostaglandins in cholinergic neurotransmission in the guinea pig. *Eur J Pharmacol* **34**, 39-47 (1975).

567. Hällgren A, Flemström G, Nylander O. Interaction between neurokinin A, VIP, prostanoids, and enteric nerves in regulation of duodenal function. *Am J Physiol* **275**, G95-103 (1998).

568. Hällgren A, Flemström G, Sababi M, Nylander O. Effects of nitric oxide inhibition on duodenal function in rat: involvement of neural mechanisms. *Am J Physiol* **269**, G246-254 (1995).

569. Hällgren A, Wilander E, Nylander O. Acid-induced increase in duodenal mucosal permeability is augmented by nitric oxide inhibition and vasopressin. *Acta Physiol Scand* **160**, 363-370 (1997).

570. Hames TK*, et al.* A comparison between the use of a shadow shield whole body counter and an uncollimated gamma camera ain the assessment of the seven-day retention of SeHCAT. *Br J Radiol* **57**, 581-584 (1984).

571. Han YM*, et al.* Mitigation of indomethacin-induced gastrointestinal damages in fat-1 transgenic mice via gate-keeper action of ω-3-polyunsaturated fatty acids. *Sci Rep* **6**, 33992 (2016).

572. Hanefeld M, Schaper F, Koehler C. Effect of acarbose on vascular disease in patients with abnormal glucose tolerance. *Cardiovasc Drugs Ther* **22**, 225-231 (2008).

573. Hannan JM*, et al.* Soluble dietary fibre fraction of Trigonella foenum-graecum (fenugreek) seed improves glucose homeostasis in animal models of type 1 and type 2 diabetes by delaying carbohydrate digestion and absorption, and enhancing insulin action. *Br J Nutr* **97**, 514-521 (2007).

574. Hansen CHF*, et al.* Targeting gut microbiota and barrier function with prebiotics to alleviate autoimmune manifestations in NOD mice. *Diabetologia* **62**, 1689-1700 (2019).

575. Hansen MB, Jaffe BM. Involvement of eicosanoids and inositol 1,4,5-trisphosphate in serotonin-induced secretion in rat ileum in vivo. *Comp Biochem Physiol C Comp Pharmacol Toxicol* **105**, 77-82 (1993).

576. Hanson WR, Thomas C. 16, 16-dimethyl prostaglandin E2 increases survival of murine intestinal stem cells when given before photon radiation. *Radiat Res* **96**, 393-398 (1983).

577. Hao Y, Gu XH, Wang XL. Overexpression of heat shock protein 70 and its relationship to intestine under acute heat stress in broilers: 1. Intestinal structure and digestive function. *Poult Sci* **91**, 781-789 (2012).

578. Hara H, Mitani N, Adachi T. Inhibitory effect of nitric oxide on the induction of cytochrome P450 3A4 mRNA by 1,25-dihydroxyvitamin D3 in Caco-2 cells. *Free Radic Res* **33**, 279-285 (2000).

579. Hardesty JE*, et al.* Transcriptional signatures of the small intestinal mucosa in response to ethanol in transgenic mice rich in endogenous n3 fatty acids. *Sci Rep* **10**, 19930 (2020).

580. Hardin J, Kroeker K, Chung B, Gall DG. Effect of proinflammatory interleukins on jejunal nutrient transport. *Gut* **47**, 184-191 (2000).

581. Harrison-Findik DD*, et al.* Alcohol metabolism-mediated oxidative stress down-regulates hepcidin transcription and leads to increased duodenal iron transporter expression. *J Biol Chem* **281**, 22974-22982 (2006).

582. Hartmann F, Bissell DM. Metabolism of heme and bilirubin in rat and human small intestinal mucosa. *J Clin Invest* **70**, 23-29 (1982).

583. Hatazawa R, Ohno R, Tanigami M, Tanaka A, Takeuchi K. Roles of endogenous prostaglandins and cyclooxygenase isozymes in healing of indomethacin-induced small intestinal lesions in rats. *J Pharmacol Exp Ther* **318**, 691-699 (2006).

584. Hayashi E, Haga Y, Shinozuka K, Takeda M. The effect of ascorbate on the acetylcholine release from guinea-pig ileal myenteric plexus. *J Pharm Pharmacol* **38**, 232-235 (1986).

585. Hayashi E, Shinozuka K, Maeda T, Takeda M. Effect of ascorbate on the contractile response induced by DMPP in guinea-pig ileal longitudinal muscle strip. *Eur J Pharmacol* **89**, 229-234 (1983).

586. Hayat MM*, et al.* Anti-diabetic and spasmolytic potential of Farsetia hamiltonii Royle from Cholistan desert. *J Ethnopharmacol* **156**, 347-352 (2014).

587. Hazinedaroglu SM*, et al.* N-acetylcysteine in intestinal reperfusion injury: an experimental study in rats. *ANZ J Surg* **74**, 676-678 (2004).

588. Hazra J. Evidence against prostaglandin E having a physiological role in acetylcholine liberation from Auerbach's plexus of guinea-pig ileum. *Experientia* **31**, 565-566 (1975).

589. He C*, et al.* Vitamin A prevents lipopolysaccharide-induced injury on tight junctions in mice. *Food Sci Nutr* **8**, 1942-1948 (2020).

590. He GZ, Dong LG, Cui XY, Chen XF, Zhang R. [Impact of glutamine and ω-3 polyunsaturated fatty acids on intestinal permeability and lung cell apoptosis during intestinal ischemia-reperfusion injury in a rat model]. *Zhonghua Wei Chang Wai Ke Za Zhi* **15**, 484-489 (2012).

591. He Q*, et al.* Intrauterine growth restriction alters the metabonome of the serum and jejunum in piglets. *Mol Biosyst* **7**, 2147-2155 (2011).

592. He W, Li X, Ding K, Li Y, Li W. Ascorbic Acid can Reverse the Inhibition of Phytic Acid, Sodium Oxalate and Sodium Silicate on Iron Absorption in Caco-2 cells. *Int J Vitam Nutr Res* **88**, 65-72 (2018).

593. He XD, Goyal RK. Nitric oxide involvement in the peptide VIP-associated inhibitory junction potential in the guinea-pig ileum. *J Physiol* **461**, 485-499 (1993).

594. Hebeiss K, Kilbinger H. Nitric oxide-sensitive guanylyl cyclase inhibits acetylcholine release and excitatory motor transmission in the guinea-pig ileum. *Neuroscience* **82**, 623-629 (1998).

595. Hebeiss K, Kilbinger H. Cholinergic and GABAergic regulation of nitric oxide synthesis in the guinea pig ileum. *Am J Physiol* **276**, G862-866 (1999).

596. Hébuterne X, Wang XD, Johnson EJ, Krinsky NI, Russell RM. Intestinal absorption and metabolism of 9-cis-beta-carotene in vivo: biosynthesis of 9-cis-retinoic acid. *J Lipid Res* **36**, 1264-1273 (1995).

597. Heinemann A, Shahbazian A, Holzer P. Cannabinoid inhibition of guinea-pig intestinal peristalsis via inhibition of excitatory and activation of inhibitory neural pathways. *Neuropharmacology* **38**, 1289-1297 (1999).

598. Heller T*, et al.* Effect of the antioxidant idebenone on adverse events under mycophenolate mofetil therapy in a rat model. *Transplantation* **85**, 739-747 (2008).

599. Hellgren S, Flemström G, Nylander O. Nitric oxide prevents rat duodenal contractions induced by potentially noxious agents. *Acta Physiol Scand* **169**, 237-247 (2000).

600. Hepgül G*, et al.* Preventive effect of pentoxifylline on acute radiation damage via antioxidant and anti-inflammatory pathways. *Dig Dis Sci* **55**, 617-625 (2010).

601. Herman EH, Ferrans VJ, Myers CE, Van Vleet JF. Comparison of the effectiveness of (+/-)-1,2-bis(3,5-dioxopiperazinyl-1-yl)propane (ICRF-187) and N-acetylcysteine in preventing chronic doxorubicin cardiotoxicity in beagles. *Cancer Res* **45**, 276-281 (1985).

602. Hernández I, Carbonell LF, Quesada T, Fenoy FJ. Role of angiotensin II in modulating the hemodynamic effects of nitric oxide synthesis inhibition. *Am J Physiol* **277**, R104-111 (1999).

603. Herrmann BW, Cullen JJ, Ledlow A, Murray JA, Conklin JL. The effect of peroxynitrite on sphincter of Oddi motility. *J Surg Res* **81**, 55-58 (1999).

604. Hidiroglou M, Butler G, Ivan M. Plasma vitamin E response in sheep dosed intraruminally or intraduodenally with various alpha-tocopherol compounds. *Int J Vitam Nutr Res* **60**, 331-337 (1990).

605. Hierholzer C, Kalff JC, Billiar TR, Bauer AJ, Tweardy DJ, Harbrecht BG. Induced nitric oxide promotes intestinal inflammation following hemorrhagic shock. *Am J Physiol Gastrointest Liver Physiol* **286**, G225-233 (2004).

606. Hirafuji M, Shinoda H. Increased prostaglandin I2 and thromboxane A2 production by rat dental pulp after intravenous administration of endotoxin. *Arch Oral Biol* **39**, 995-1000 (1994).

607. Hirota CL*, et al.* Epidermal growth factor receptor transactivation is required for proteinase-activated receptor-2-induced COX-2 expression in intestinal epithelial cells. *Am J Physiol Gastrointest Liver Physiol* **303**, G111-119 (2012).

608. Ho Do M, Seo YS, Park HY. Polysaccharides: bowel health and gut microbiota. *Crit Rev Food Sci Nutr*, 1-13 (2020).

609. Hock FJ*, et al.* Hoe 140 a new potent and long acting bradykinin-antagonist: in vitro studies. *Br J Pharmacol* **102**, 769-773 (1991).

610. Hogan DL, Ballesteros MA, Koss MA, Isenberg JI. Cyclooxygenase inhibition with indomethacin increases human duodenal mucosal response to prostaglandin E1. *Dig Dis Sci* **34**, 1855-1859 (1989).

611. Hokari R*, et al.* Nitric oxide modulates T-lymphocyte migration in Peyer's patches and villous submucosa of rat small intestine. *Gastroenterology* **115**, 618-627 (1998).

612. Hollander D, Dadufalza V. Lymphatic and portal absorption of vitamin E in aging rats. *Dig Dis Sci* **34**, 768-772 (1989).

613. Hollman PC, Bijsman MN, van Gameren Y, Cnossen EP, de Vries JH, Katan MB. The sugar moiety is a major determinant of the absorption of dietary flavonoid glycosides in man. *Free Radic Res* **31**, 569-573 (1999).

614. Holm M, Johansson B, Pettersson A, Fändriks L. Acid-induced duodenal mucosal nitric oxide output parallels bicarbonate secretion in the anaesthetized pig. *Acta Physiol Scand* **162**, 461-468 (1998).

615. Holm M, Johansson B, Pettersson A, Fändriks L. Carbon dioxide mediates duodenal mucosal alkaline secretion in response to luminal acidity in the anesthetized rat. *Gastroenterology* **115**, 680-685 (1998).

616. Holm M, Johansson B, von Bothmer C, Jönson C, Pettersson A, Fändriks L. Acid-induced increase in duodenal mucosal alkaline secretion in the rat involves the L-arginine/NO pathway. *Acta Physiol Scand* **161**, 527-532 (1997).

617. Holota YV, Olefir YA, Dovbynchuk TV, Tolstanova GM. Carbohydrate composition of rat intestine surface mucus layer after ceftriaxone treatment. *Ukr Biochem J* **88**, 35-44 (2016).

618. Holzer P. Involvement of nitric oxide in the substance P-induced inhibition of intestinal peristalsis. *Neuroreport* **8**, 2857-2860 (1997).

619. Holzer P, Lippe IT, Tabrizi AL, Lènárd L, Jr., Barthó L. Dual excitatory and inhibitory effect of nitric oxide on peristalsis in the guinea pig intestine. *J Pharmacol Exp Ther* **280**, 154-161 (1997).

620. Hombach J, Hoyer H, Bernkop-Schnürch A. Thiolated chitosans: development and in vitro evaluation of an oral tobramycin sulphate delivery system. *Eur J Pharm Sci* **33**, 1-8 (2008).

621. Hong KH, Bonventre JC, O'Leary E, Bonventre JV, Lander ES. Deletion of cytosolic phospholipase A(2) suppresses Apc(Min)-induced tumorigenesis. *Proc Natl Acad Sci U S A* **98**, 3935-3939 (2001).

622. Hopkins BJ, Hodgson WC, Sutherland SK. Pharmacological studies of stonefish (Synanceja trachynis) venom. *Toxicon* **32**, 1197-1210 (1994).

623. Hopkins BJ, Hodgson WC, Sutherland SK. Evidence for adrenergic and tachykinin activity in venom of the stonefish (Synanceja trachynis). *Toxicon* **34**, 541-554 (1996).

624. Hori M*, et al.* Upregulation of iNOS by COX-2 in muscularis resident macrophage of rat intestine stimulated with LPS. *Am J Physiol Gastrointest Liver Physiol* **280**, G930-938 (2001).

625. Horie S*, et al.* Involvement of cyclooxygenase-dependent pathway in contraction of isolated ileum by urotensin II. *Peptides* **26**, 323-329 (2005).

626. Horio F, Shibata T, Naito Y, Nishikimi M, Yagi K, Yoshida A. L-gulono-gamma-lactone oxidase is not induced in rats by xenobiotics stimulating L-ascorbic acid biosynthesis. *J Nutr Sci Vitaminol (Tokyo)* **39**, 1-9 (1993).

627. Horrobin DF, Manku MS, Franks DJ, Hamet P. Methyl xanthine phosphodiesterase inhibitors behave as prostaglandin antagonists in a perfused rat mesenteric artery preparation. *Prostaglandins* **13**, 33-40 (1977).

628. Hosseini SM, Soltanizadeh N, Mirmoghtadaee P, Banavand P, Mirmoghtadaie L, Shojaee-Aliabadi S. Gluten-free products in celiac disease: Nutritional and technological challenges and solutions. *J Res Med Sci* **23**, 109 (2018).

629. Hou XL*, et al.* Possible inhibitory mechanism of Curcuma drugs on CYP3A4 in 1alpha,25 dihydroxyvitamin D3 treated Caco-2 cells. *Int J Pharm* **337**, 169-177 (2007).

630. Hou Y, Wang L, Yi D, Wu G. N-acetylcysteine and intestinal health: a focus on its mechanism of action. *Front Biosci (Landmark Ed)* **20**, 872-891 (2015).

631. Hou Y, Wu Z, Dai Z, Wang G, Wu G. Protein hydrolysates in animal nutrition: Industrial production, bioactive peptides, and functional significance. *J Anim Sci Biotechnol* **8**, 24 (2017).

632. Houdijk AP*, et al.* Glutamine-enriched enteral diet increases splanchnic blood flow in the rat. *Am J Physiol* **267**, G1035-1040 (1994).

633. Houston JB, Wilkens HJ, Levy G. Potentiation of isoproterenol effect by ascorbic acid. *Res Commun Chem Pathol Pharmacol* **14**, 643-650 (1976).

634. Houston SA, Cerovic V, Thomson C, Brewer J, Mowat AM, Milling S. The lymph nodes draining the small intestine and colon are anatomically separate and immunologically distinct. *Mucosal Immunol* **9**, 468-478 (2016).

635. Hryhorenko LM, Woskowska Z, Fox-Threlkeld JA. Nitric oxide (NO) inhibits release of acetylcholine from nerves of isolated circular muscle of the canine ileum: relationship to motility and release of nitric oxide. *J Pharmacol Exp Ther* **271**, 918-926 (1994).

636. Hsieh NK, Chang HR, Hu CT, Chen HI. Effects of nitric oxide donor and nitric oxide synthase inhibitor on the resistance, exchange and capacitance functions of the canine intestinal vasculature. *Vascul Pharmacol* **48**, 122-128 (2008).

637. Hu C, Zhang G, Sun D, Han H, Hu S. Duodenal-jejunal bypass improves glucose metabolism and adipokine expression independently of weight loss in a diabetic rat model. *Obes Surg* **23**, 1436-1444 (2013).

638. Hu S, Che JW, Du Y, Bao CM. [Observation on the effect of vitamin C in alleviating peroxidative damage in gut of dogs during enteral fluid resuscitation of severe burn shock]. *Zhonghua Shao Shang Za Zhi* **25**, 451-453 (2009).

639. Hu X*, et al.* ATF4 Deficiency Promotes Intestinal Inflammation in Mice by Reducing Uptake of Glutamine and Expression of Antimicrobial Peptides. *Gastroenterology* **156**, 1098-1111 (2019).

640. Huang BB, Li GF, Luo JH, Duan L, Nobuaki K, Akira Y. Permeabilities of rebamipide via rat intestinal membranes and its colon specific delivery using chitosan capsule as a carrier. *World J Gastroenterol* **14**, 4928-4937 (2008).

641. Huang CY, Hsiao JK, Lu YZ, Lee TC, Yu LC. Anti-apoptotic PI3K/Akt signaling by sodium/glucose transporter 1 reduces epithelial barrier damage and bacterial translocation in intestinal ischemia. *Lab Invest* **91**, 294-309 (2011).

642. Huang H*, et al.* Vitamin D receptor interacts with NLRP3 to restrict the allergic response. *Clin Exp Immunol* **194**, 17-26 (2018).

643. Huang JQ, Lu XH, Mai CR. [Effect of drugs on endoscopic manometry of the sphincter of Oddi]. *Zhonghua Nei Ke Za Zhi* **30**, 699-702, 731 (1991).

644. Huang Q*, et al.* Protective effects of leucine on redox status and mitochondrial-related gene abundance in the jejunum of intrauterine growth-retarded piglets during early weaning period. *Arch Anim Nutr* **71**, 93-107 (2017).

645. Huang ZH, Lin HW, Li Z, Feng HM, Sun YG, Zhang QG. [L-arginine decreases P-selectin expression in traumatic shock]. *Di Yi Jun Yi Da Xue Xue Bao* **23**, 777-780 (2003).

646. Huang ZH, Sun YG, Feng HM, Lei HY, Lin HW, Song HJ. [Therapeutic effect of L-arginine on traumatic shock in rats]. *Di Yi Jun Yi Da Xue Xue Bao* **23**, 46-49 (2003).

647. Hugi D, Bruckmaier RM, Blum JW. Insulin resistance, hyperglycemia, glucosuria, and galactosuria in intensively milk-fed calves: dependency on age and effects of high lactose intake. *J Anim Sci* **75**, 469-482 (1997).

648. Hulme HE*, et al.* Mass spectrometry imaging identifies palmitoylcarnitine as an immunological mediator during Salmonella Typhimurium infection. *Sci Rep* **7**, 2786 (2017).

649. Humbert B, Nguyen P, Dumon H, Deschamps JY, Darmaun D. Does enteral glutamine modulate whole-body leucine kinetics in hypercatabolic dogs in a fed state? *Metabolism* **51**, 628-635 (2002).

650. Hunt JN, Knox MT. The slowing of gastric emptying by four strong acids and three weak acids. *J Physiol* **222**, 187-208 (1972).

651. Husemann B, Groitl H, Zirngibl H. [Metabolic and surgical aspects of total gastrectomy (author's transl)]. *MMW Munch Med Wochenschr* **120**, 561-564 (1978).

652. Hutcheson IR, Whittle BJ, Boughton-Smith NK. Role of nitric oxide in maintaining vascular integrity in endotoxin-induced acute intestinal damage in the rat. *Br J Pharmacol* **101**, 815-820 (1990).

653. Hyland NP, Rybicka JM, Ho W, Pittman QJ, Macnaughton WK, Sharkey KA. Adaptation of intestinal secretomotor function and nutrient absorption in response to diet-induced obesity. *Neurogastroenterol Motil* **22**, 602-e171 (2010).

654. Hylton DJ, Phillips LM, Hoffman SM, Fleming SD. Hemorrhage-induced intestinal damage is complement-independent in Helicobacter hepaticus-infected mice. *Shock* **34**, 467-474 (2010).

655. Hyre CE, Unthank JL, Dalsing MC. Direct in vivo measurement of flow-dependent nitric oxide production in mesenteric resistance arteries. *J Vasc Surg* **27**, 726-732 (1998).

656. Iannotti FA*, et al.* Analysis of the "endocannabinoidome" in peripheral tissues of obese Zucker rats. *Prostaglandins Leukot Essent Fatty Acids* **89**, 127-135 (2013).

657. Ibbotson GC, Wallace JL. Beneficial effects of prostaglandin E2 in endotoxic shock are unrelated to effects on PAF-acether synthesis. *Prostaglandins* **37**, 237-250 (1989).

658. Ibrahim A, Gilzad-kohan MH, Aghazadeh-Habashi A, Jamali F. Absorption and bioavailability of glucosamine in the rat. *J Pharm Sci* **101**, 2574-2583 (2012).

659. Imaeda H, Fujimoto T, Takahashi K, Kasumi E, Fujiyama Y, Andoh A. Terminal-restriction fragment length polymorphism (T-RFLP) analysis for changes in the gut microbiota profiles of indomethacin- and rebamipide-treated mice. *Digestion* **86**, 250-257 (2012).

660. Inada I, Satoh H, Inatomi N, Nagaya H, Maki Y. Spizofurone, a new anti-ulcer agent, increases alkaline secretion in isolated bullfrog duodenal mucosa. *Eur J Pharmacol* **124**, 149-155 (1986).

661. Inkinen J, Sand J, Arvola P, Pörsti I, Nordback I. Direct effect of thyroxine on pig sphincter of Oddi contractility. *Dig Dis Sci* **46**, 182-186 (2001).

662. Inuzuka K*, et al.* Effect of hyperbarically oxygenated-perfluorochemical with University of Wisconsin solution on preservation of rat small intestine using an original pressure-resistant portable apparatus. *Surgery* **142**, 57-66 (2007).

663. Iordache C, Drozdowski L, Clandinin MT, Wild G, Todd Z, Thomson AB. Treatment of suckling rats with GLP-2 plus dexamethasone increases the ileal uptake of fatty acids in later life. *Am J Physiol Gastrointest Liver Physiol* **288**, G54-59 (2005).

664. Irie K, Fujii E, Uchida Y, Muraki T. Involvement of endogenous nitric oxide in non-adrenergic, non-cholinergic contraction elicited by [Met5]-enkephalin in rat isolated duodenum. *Neuropharmacology* **33**, 1333-1338 (1994).

665. Isenberg JI, Hogan DL. Human duodenal mucosal bicarbonate secretion--physiological and clinical aspects. *J Intern Med Suppl* **732**, 113-117 (1990).

666. Isenberg JI, Smedfors B, Johansson C. Effect of graded doses of intraluminal H+, prostaglandin E2, and inhibition of endogenous prostaglandin synthesis on proximal duodenal bicarbonate secretion in unanesthetized rat. *Gastroenterology* **88**, 303-307 (1985).

667. Ishii R, Sakai E, Nakajima K, Matsuhashi N, Ohata K. Non-occlusive mesenteric ischemia induced by a polyethylene glycol with ascorbate-based colonic bowel preparation. *Clin J Gastroenterol* **12**, 403-406 (2019).

668. Ishikawa H, Watanabe S. Cattle bile aggravates diclofenac sodium-induced small intestinal injury in mice. *Evid Based Complement Alternat Med* **2011**, 315858 (2011).

669. Ishikawa N, Horii Y, Oinuma T, Suganuma T, Nawa Y. Goblet cell mucins as the selective barrier for the intestinal helminths: T-cell-independent alteration of goblet cell mucins by immunologically 'damaged' Nippostrongylus brasiliensis worms and its significance on the challenge infection with homologous and heterologous parasites. *Immunology* **81**, 480-486 (1994).

670. Ishizawa M. Biphasic response of circular muscle to prostaglandin E2 in guinea-pig colon. *Arch Int Pharmacodyn Ther* **295**, 282-290 (1988).

671. Islam MN, Chanda S, Mitra C. Effects of different intensities of cold stress on certain physiological phenomena related to skeletal health in a hypogonadal rat model. *J Physiol Pharmacol* **51**, 857-870 (2000).

672. Islam N, Chanda S, Ghosh TK, Mitra C. Cold stress facilitates calcium mobilization from bone in an ovariectomized rat model of osteoporosis. *Jpn J Physiol* **48**, 49-55 (1998).

673. Ito Y*, et al.* Nonsteroidal anti-inflammatory drug-induced visible and invisible small intestinal injury. *J Clin Biochem Nutr* **53**, 55-59 (2013).

674. Ivancheva C, Itzev D, Lolova I, Radomirov R. Contribution of nitric oxide and substance P to nonadrenergic, noncholinergic transmission in the guinea pig ileum. *Gen Pharmacol* **31**, 101-105 (1998).

675. Ivancheva C, Itzev D, Radomirov R. Functional antagonism between nitric oxide and ATP in the motor responses of guinea-pig ileum. *J Auton Pharmacol* **20**, 147-156 (2000).

676. Ivins JK, Penning TM. Radiochemical detection of dihydrodiol dehydrogenase: distribution of the enzyme in male Sprague-Dawley rat tissues and its sensitivity to inhibition by indomethacin and 6-medroxyprogesterone acetate. *Cancer Res* **47**, 680-684 (1987).

677. Iwahara S*, et al.* Purification, characterization, and cloning of a heme-binding protein (23 kDa) in rat liver cytosol. *Biochemistry* **34**, 13398-13406 (1995).

678. Iwama M, Amano A, Shimokado K, Maruyama N, Ishigami A. Ascorbic acid levels in various tissues, plasma and urine of mice during aging. *J Nutr Sci Vitaminol (Tokyo)* **58**, 169-174 (2012).

679. Izzo AA*, et al.* Inhibitory effect of cannabichromene, a major non-psychotropic cannabinoid extracted from Cannabis sativa, on inflammation-induced hypermotility in mice. *Br J Pharmacol* **166**, 1444-1460 (2012).

680. Izzo AA, Mascolo N, Maiolino P, Capasso F. Nitric oxide-donating compounds and cyclic GMP depress the spontaneous contractile activity of the isolated rabbit jejunum. *Pharmacology* **53**, 109-113 (1996).

681. Izzo AA*, et al.* Basal and fasting/refeeding-regulated tissue levels of endogenous PPAR-alpha ligands in Zucker rats. *Obesity (Silver Spring)* **18**, 55-62 (2010).

682. Izzo RS, Witkon K, Chen AI, Hadjiyane C, Weinstein MI, Pellecchia C. Interleukin-8 and neutrophil markers in colonic mucosa from patients with ulcerative colitis. *Am J Gastroenterol* **87**, 1447-1452 (1992).

683. Jabri MA*, et al.* Fatty acid composition and mechanisms of the protective effects of myrtle berry seed aqueous extract in alcohol-induced peptic ulcer in rat. *Can J Physiol Pharmacol* **95**, 510-521 (2017).

684. Jacob JV. Nicorandil-induced colonic ulceration. *BMJ Case Rep* **2015**, (2015).

685. Jager LP, Zijlstra FJ, Hoogendoorn A, Nabuurs MJ. Enteropooling in piglets induced by soya-peptone mediated via an increased biosynthesis of prostanoids. *Vet Res Commun* **10**, 407-412 (1986).

686. Jakobsdottir G*, et al.* Formation of short-chain Fatty acids, excretion of anthocyanins, and microbial diversity in rats fed blackcurrants, blackberries, and raspberries. *J Nutr Metab* **2013**, 202534 (2013).

687. Jakobsson S, Ahlberg K, Taft C, Ekman T. Exploring a link between fatigue and intestinal injury during pelvic radiotherapy. *Oncologist* **15**, 1009-1015 (2010).

688. Jakse G, Reisegger W, Frick J. [Gas in the draining urinary tract (author's transl)]. *Urologe A* **15**, 133-134 (1976).

689. Jama HA, Beale A, Shihata WA, Marques FZ. The effect of diet on hypertensive pathology: is there a link via gut microbiota-driven immunometabolism? *Cardiovasc Res* **115**, 1435-1447 (2019).

690. Jankowski J, Tykałowski B, Ognik K, Koncicki A, Kubińska M, Zduńczyk Z. The effect of different dietary levels of DL-methionine and DL-hydroxy analogue on the antioxidant status of young turkeys infected with the haemorrhagic enteritis virus. *BMC Vet Res* **14**, 404 (2018).

691. Jansson L, Carlsson PO, Bodin B, Källskog O. Flow distribution during infusion of UW and HTK solution in anaesthetised rats. *Langenbecks Arch Surg* **396**, 677-683 (2011).

692. Jarry A*, et al.* Heterogeneity of subordination of the IL-18/IFN-γ axis to caspase-1 among patients with Crohn's disease. *Lab Invest* **95**, 1207-1217 (2015).

693. Jaworek J, Nawrot-Porabka K, Leja-Szpak A, Konturek SJ. Brain-gut axis in the modulation of pancreatic enzyme secretion. *J Physiol Pharmacol* **61**, 523-531 (2010).

694. Jeanneton O, Delvaux M, Botella A, Frexinos J, Bueno L. Homologous desensitization of PAF receptors via a PGE2-dependent pathway on intestinal smooth muscle. *J Lipid Mediat Cell Signal* **10**, 331-344 (1994).

695. Jeanneton O, Delvaux M, Frexinos J, Bueno L. Desensitization of platelet-activating factor receptors, induced by inflammation in guinea pig ileal smooth muscle cells. *Gastroenterology* **108**, 1666-1675 (1995).

696. Jeanneton O*, et al.* Correlation of desensitisation of platelet activating factor (PAF) receptors with intensity of inflammation and intestinal PAF content during experimental ileitis in guinea pig. *Gut* **43**, 356-364 (1998).

697. Jebbink MC, Jansen JB, Mooy DM, Rovati LC, Lamers CB. Effect of the specific cholecystokinin-receptor antagonist loxiglumide on bombesin stimulated pancreatic enzyme secretion in man. *Regul Pept* **32**, 361-368 (1991).

698. Jeong SY, Im YN, Youm JY, Lee HK, Im SY. l-Glutamine Attenuates DSS-Induced Colitis via Induction of MAPK Phosphatase-1. *Nutrients* **10**, (2018).

699. Jeremy JY, Mikhailidis DP, Dandona P. The effect of tiaprofenic acid and indomethacin on in vitro prostaglandin synthesis by rat, rabbit and human stomach tissue. *Agents Actions* **17**, 205-208 (1985).

700. Jewell C, O'Brien NM. Effect of dietary supplementation with carotenoids on xenobiotic metabolizing enzymes in the liver, lung, kidney and small intestine of the rat. *Br J Nutr* **81**, 235-242 (1999).

701. Jia L, Stamler JS. Dual actions of S-nitrosylated derivative of vasoactive intestinal peptide as a vasoactive intestinal peptide-like mediator and a nitric oxide carrier. *Eur J Pharmacol* **366**, 79-86 (1999).

702. Jiang W*, et al.* Relationship between contents of adrenomedullin and distributions of neutral endopeptidase in blood and tissues of rats in septic shock. *Regul Pept* **118**, 199-208 (2004).

703. Jiao J, Zhang X, Wang M, Zhou C, Yan Q, Tan Z. Linkages between Epithelial Microbiota and Host Transcriptome in the Ileum during High-Grain Challenges: Implications for Gut Homeostasis in Goats. *J Agric Food Chem* **67**, 551-561 (2019).

704. Jin CJ, Sellmann C, Engstler AJ, Ziegenhardt D, Bergheim I. Supplementation of sodium butyrate protects mice from the development of non-alcoholic steatohepatitis (NASH). *Br J Nutr* **114**, 1745-1755 (2015).

705. Jing F, Liu M, Yang N, Liu Y, Li X, Li J. Relaxant effect of chloroquine in rat ileum: possible involvement of nitric oxide and BKCa. *J Pharm Pharmacol* **65**, 847-854 (2013).

706. Jing K, Sun M. [Relationship between the regulation of intestinal NF-κB and TNF-α by glutamine and the protective effects of glutamine against intestinal injury]. *Zhongguo Dang Dai Er Ke Za Zhi* **13**, 661-664 (2011).

707. Jlali M*, et al.* Nutrigenetics of carotenoid metabolism in the chicken: a polymorphism at the β,β-carotene 15,15'-mono-oxygenase 1 (BCMO1) locus affects the response to dietary β-carotene. *Br J Nutr* **111**, 2079-2088 (2014).

708. Jo SH, Ha KS, Moon KS, Lee OH, Jang HD, Kwon YI. In vitro and in vivo anti-hyperglycemic effects of Omija (Schizandra chinensis) fruit. *Int J Mol Sci* **12**, 1359-1370 (2011).

709. Johnson JH*, et al.* Duodenal perforation in a cheetah (Acinonyx jubilatus). *J Zoo Wildl Med* **28**, 481-484 (1997).

710. Johnson LM, Harrison JH, Riley RE. Estimation of the flow of microbial nitrogen to the duodenum using urinary uric acid or allantoin. *J Dairy Sci* **81**, 2408-2420 (1998).

711. Johnson MA, Hill RG, Hughes J. A possible role for prostaglandins in the expression of morphine dependence in guinea-pig isolated ileum. *Br J Pharmacol* **93**, 932-936 (1988).

712. Johnston JK, Freeman DE, Gillette D, Soma LR. Effects of superoxide dismutase on injury induced by anoxia and reoxygenation in equine small intestine in vitro. *Am J Vet Res* **52**, 2050-2054 (1991).

713. Jones RC, Sugie S, Braley J, Weisburger JH. Dietary beta-carotene in rat models of gastrointestinal cancer. *J Nutr* **119**, 508-514 (1989).

714. Joyce SA, Gahan CG. The gut microbiota and the metabolic health of the host. *Curr Opin Gastroenterol* **30**, 120-127 (2014).

715. Ju J*, et al.* Inhibition of intestinal tumorigenesis in Apcmin/+ mice by (-)-epigallocatechin-3-gallate, the major catechin in green tea. *Cancer Res* **65**, 10623-10631 (2005).

716. Jung SE, Youn YK, Lim YS, Song HG, Rhee JE, Suh GJ. Combined administration of glutamine and growth hormone synergistically reduces bacterial translocation in sepsis. *J Korean Med Sci* **18**, 17-22 (2003).

717. Juresa D, Blanusa M, Francesconi KA, Kienzl N, Kuehnelt D. Biological availability of selenosugars in rats. *Chem Biol Interact* **168**, 203-210 (2007).

718. Jurgoński A, Juśkiewicz J, Sójka M, Król B, Rój E, Zduńczyk Z. [Black currant seeds after supercritical carbon dioxide extraction as a potential dietary supplement]. *Rocz Panstw Zakl Hig* **59**, 421-427 (2008).

719. Kachur JF, Won-Kim S, Anglin C, Gaginella TS. Eicosanoids and histamine mediate C5a-induced electrolyte secretion in guinea pig ileal mucosa. *Inflammation* **19**, 717-725 (1995).

720. Kadlec O, Masek K, Růzicka V, Seferna I. Possible role of prostaglandins in post-tetanic potentiation at the nerve-muscle junction in the longitudinal muscle strip of guinea-pig ileum. *J Pharm Pharmacol* **36**, 65-68 (1984).

721. Kadlec O, Masek K, Seferna I. A modulating role of prostaglandins in contractions of the guinea-pig ileum. *Br J Pharmacol* **51**, 565-570 (1974).

722. Kadlec O, Masek K, Seferna I. Post-tetanic potentiation in the innervated smooth muscle preparation of the guinea-pig ileum. *Eur J Pharmacol* **60**, 383-386 (1979).

723. Kågström J, Holmgren S. Vip-induced relaxation of small arteries of the rainbow trout, Oncorhynchus mykiss, involves prostaglandin synthesis but not nitric oxide. *J Auton Nerv Syst* **63**, 68-76 (1997).

724. Kahle K, Huemmer W, Kempf M, Scheppach W, Erk T, Richling E. Polyphenols are intensively metabolized in the human gastrointestinal tract after apple juice consumption. *J Agric Food Chem* **55**, 10605-10614 (2007).

725. Kaithwas G, Majumdar DK. Evaluation of antiulcer and antisecretory potential of Linum usitatissimum fixed oil and possible mechanism of action. *Inflammopharmacology* **18**, 137-145 (2010).

726. Kajarabille N, Brown C, Cucliciu A, Thapaliya G, Latunde-Dada GO. Bioavailability of iron multi-amino acid chelate preparation in mice and human duodenal HuTu 80 cells. *Br J Nutr* **117**, 767-774 (2017).

727. Kaji I, Karaki S, Kuwahara A. Effects of luminal thymol on epithelial transport in human and rat colon. *Am J Physiol Gastrointest Liver Physiol* **300**, G1132-1143 (2011).

728. Kajimoto T, Dinning PG, Gibb DB, de Carle DJ, Cook IJ. Neurogenic pathways mediating ascending and descending reflexes at the porcine ileocolonic junction. *Neurogastroenterol Motil* **12**, 125-134 (2000).

729. Kalloo NB, Jeffs RD, Gearhart JP. Long-term nutritional consequences of bowel segment use for lower urinary tract reconstruction in pediatric patients. *Urology* **50**, 967-971 (1997).

730. Kalogeris TJ, Monroe F, Tso P. Stimulation of intestinal apolipoprotein A-IV by lipid is independent of capsaicin-sensitive afferent signals. *Am J Physiol* **273**, R981-990 (1997).

731. Kalogeris TJ, Painter RG, Holden VR. Ileal lipid infusion stimulates jejunal synthesis of apolipoprotein A-IV without affecting mRNA levels. *Proc Soc Exp Biol Med* **223**, 198-202 (2000).

732. Kalogeris TJ, Tsuchiya T, Fukagawa K, Wolf R, Tso P. Apolipoprotein A-IV synthesis in proximal jejunum is stimulated by ileal lipid infusion. *Am J Physiol* **270**, G277-286 (1996).

733. Kam YL, Rhee SJ, Choo HY. Solution-phase combinatorial synthesis of nonpeptide bradykinin antagonists. *Bioorg Med Chem* **12**, 3543-3552 (2004).

734. Kamishikiryo J*, et al.* N-terminal domain of the cholesterol transporter Niemann-Pick C1-like 1 (NPC1L1) is essential for α-tocopherol transport. *Biochem Biophys Res Commun* **486**, 476-480 (2017).

735. Kamp ME*, et al.* G Protein-Coupled Receptor 43 Modulates Neutrophil Recruitment during Acute Inflammation. *PLoS One* **11**, e0163750 (2016).

736. Kamycheva E, Goto T, Camargo CA, Jr. Celiac disease is associated with reduced bone mineral density and increased FRAX scores in the US National Health and Nutrition Examination Survey. *Osteoporos Int* **28**, 781-790 (2017).

737. Kanada A, Hosokawa M, Suthamnatpong N, Maehara T, Takeuchi T, Hata F. Neuronal pathway involved in nitric oxide-mediated descending relaxation in rat ileum. *Eur J Pharmacol* **250**, 59-66 (1993).

738. Kandil HM, Berschneider HM, Argenzio RA. Tumour necrosis factor alpha changes porcine intestinal ion transport through a paracrine mechanism involving prostaglandins. *Gut* **35**, 934-940 (1994).

739. Kaneko C*, et al.* Fructose suppresses uric acid excretion to the intestinal lumen as a result of the induction of oxidative stress by NADPH oxidase activation. *Biochim Biophys Acta Gen Subj* **1861**, 559-566 (2017).

740. Kang M, Hashimoto A, Gade A, Akbarali HI. Interaction between hydrogen sulfide-induced sulfhydration and tyrosine nitration in the KATP channel complex. *Am J Physiol Gastrointest Liver Physiol* **308**, G532-539 (2015).

741. Kang SH*, et al.* Aronia Berry Extract Ameliorates the Severity of Dextran Sodium Sulfate-Induced Ulcerative Colitis in Mice. *J Med Food* **20**, 667-675 (2017).

742. Kania BF, Matczuk J, Romanowicz K, Buéno L, Fioramonti J, Kania K. CCK1 central receptor antagonist prevented the intestinal stress symptoms in sheep. *Pol J Vet Sci* **5**, 35-42 (2002).

743. Kanter M, Akpolat M. Vitamin C protects against ionizing radiation damage to goblet cells of the ileum in rats. *Acta Histochem* **110**, 481-490 (2008).

744. Kao J, Iwata F, Zhang XY, Baker M, Seno K, Leung FW. Inhibition of endogenous nitric oxide reduces basal mesenteric vascular tone but does not alter intraduodenal hydrochloric acid-induced intestinal hyperemia in rats. *Dig Dis Sci* **40**, 1729-1737 (1995).

745. Kaputlu I, Ozdem S, Sadan G, Gökalp O. Effects of diabetes on non-adrenergic, non-cholinergic relaxation induced by GABA and electrical stimulation in the rat isolated duodenum. *Clin Exp Pharmacol Physiol* **26**, 724-728 (1999).

746. Kaputlu I, Sadan G. Evidence that nitric oxide mediates non-adrenergic non-cholinergic relaxation induced by GABA and electrical stimulation in the rat isolated duodenum. *J Auton Pharmacol* **16**, 177-182 (1996).

747. Karatabanova NA, Spirichev VB, Sokolova SV, Isaeva VA. [Effect of phosphorus intake on calcium homeostasis and bone tissue status in rats receiving hydrocortisone]. *Vopr Pitan*, 32-39 (1983).

748. Karatepe O*, et al.* Effects of glutamine and curcumin on bacterial translocation in jaundiced rats. *World J Gastroenterol* **16**, 4313-4320 (2010).

749. Karbach U. Segmental heterogeneity of cellular and paracellular calcium transport across the rat duodenum and jejunum. *Gastroenterology* **100**, 47-58 (1991).

750. Karbach U, Rummel W. Cellular and paracellular calcium transport in the rat ileum and the influence of 1 alpha, 25-dihydroxyvitamin D3 and dexamethasone. *Naunyn Schmiedebergs Arch Pharmacol* **336**, 117-124 (1987).

751. Karbach U, Schmitt A, Saner FH. Different mechanism of magnesium and calcium transport across rat duodenum. *Dig Dis Sci* **36**, 1611-1618 (1991).

752. Karbach U, Singe CC, Mähtricht M, Ewe K. Comparison of maximal postprandial serum cholylglycine concentration with the retention of 75Se-homotaurocholic acid in ileal dysfunction. *Z Gastroenterol* **27**, 258-262 (1989).

753. Karimov K, Daminov Sh N, Khoroshaev VA. [The effect of perftoran on the morphofunctional status of the digestive system organs in experimental duodenal ulcer]. *Eksp Klin Farmakol* **62**, 33-36 (1999).

754. Karpov LM. [The role of Na K ATPase in thiamine and lipoic acid interrelations during their absorption in the gastrointestinal tract of mice]. *Fiziol Zh* **35**, 51-57 (1989).

755. Kaske M. [Physiological function of the gastrointestinal tract and pathophysiological changes in neonatal diarrhea of calves]. *Dtsch Tierarztl Wochenschr* **100**, 434-439 (1993).

756. Kasparek MS, Linden DR, Farrugia G, Sarr MG. Hydrogen sulfide modulates contractile function in rat jejunum. *J Surg Res* **175**, 234-242 (2012).

757. Katerelos C, Constantopoulos A, Agathopoulos A, Constantzas N, Zannos-Mariolea L, Matsaniotis N. Serum levels of retinol, retinol-binding protein, carotenoids and triglycerides in children with beta-thalassemia major. *Acta Haematol* **62**, 100-105 (1979).

758. Kato S, Tanaka A, Kunikata T, Umeda M, Takeuchi K. Protective effect of lafutidine against indomethacin-induced intestinal ulceration in rats: relation to capsaicin-sensitive sensory neurons. *Digestion* **61**, 39-46 (2000).

759. Katz KD*, et al.* Intestinal permeability in patients with Crohn's disease and their healthy relatives. *Gastroenterology* **97**, 927-931 (1989).

760. Kauffman GL, Jr., Kolve E, Walfisch S, Mangus J. Role of prostanoids in experimental duodenal ulcer in rat. *Dig Dis Sci* **33**, 667-672 (1988).

761. Kaufman HS, Shermak MA, May CA, Pitt HA, Lillemoe KD. Nitric oxide inhibits resting sphincter of Oddi activity. *Am J Surg* **165**, 74-80 (1993).

762. Kaur M, Kaur J, Ojha S, Mahmood A. Ethanol effects on lipid peroxidation and glutathione-mediated defense in rat small intestine: role of dietary fats. *Alcohol* **15**, 65-69 (1998).

763. Kaur T, Singh S, Dhawan V, Ganguly NK. Shigella dysenteriae type 1 toxin induced lipid peroxidation in enterocytes isolated from rabbit ileum. *Mol Cell Biochem* **178**, 169-179 (1998).

764. Kaur T, Singh S, Verma M, Ganguly NK. Calcium and protein kinase C play a significant role in response to Shigella toxin in rabbit ileum both in vivo and in vitro. *Biochim Biophys Acta* **1361**, 75-91 (1997).

765. Kaushik S, Kaur J. Chronic cold exposure affects the antioxidant defense system in various rat tissues. *Clin Chim Acta* **333**, 69-77 (2003).

766. Kawade N, Murai A, Suzuki W, Tokuda Y, Kobayashi M, Horio F. Ascorbic acid deficiency increases hepatic expression of acute phase proteins through the intestine-derived IL-6 and hepatic STAT3 pathway in ODS rats. *J Nutr Biochem* **70**, 116-124 (2019).

767. Kawashima Y, Takeyoshi I, Furukawa H, Lee RG, Starzl TE, Todo S. Cold preservation of the human colon and ileum with University of Wisconsin solution. *Clin Transplant* **13**, 420-425 (1999).

768. Kayhan N*, et al.* The adenosine deaminase inhibitor erythro-9-[2-hydroxyl-3-nonyl]-adenine decreases intestinal permeability and protects against experimental sepsis: a prospective, randomised laboratory investigation. *Crit Care* **12**, R125 (2008).

769. Keller MD, Pollitt CC, Marx UC. Nuclear magnetic resonance-based metabonomic study of early time point laminitis in an oligofructose-overload model. *Equine Vet J* **43**, 737-743 (2011).

770. Keller R, Jones VE. Immunological and pharmacological analysis of the primary and secondary reagin response to Nippostrongylus brasiliensis in the rat. *Immunology* **21**, 565-574 (1971).

771. Kellet GL, Barker ED, Beach NL, Dempster JA. Effect of nordihydroguaiaretic acid on glucose absorption, metabolism and (Na(+)+K+)-ATPase activity in rat jejunum. *Biochem Pharmacol* **45**, 1932-1935 (1993).

772. Kelly V*, et al.* Rapid, quantitative analysis of 3'- and 6'-sialyllactose in milk by flow-injection analysis-mass spectrometry: screening of milks for naturally elevated sialyllactose concentration. *J Dairy Sci* **96**, 7684-7691 (2013).

773. Kesici S, Turkmen UA, Kesici U, Altan A, Polat E. Effects of enteral and parenteral glutamine on intestinal mucosa and on levels of blood glutamine, tumor necrosis factor-alpha, and interleukin-10 in an experimental sepsis model. *Saudi Med J* **33**, 262-271 (2012).

774. Kettunen HL, Kettunen AS, Rautonen NE. Intestinal immune responses in wild-type and Apcmin/+ mouse, a model for colon cancer. *Cancer Res* **63**, 5136-5142 (2003).

775. Khajali F, Moghaddam MH, Hassanpour H. An L-Arginine supplement improves broiler hypertensive response and gut function in broiler chickens reared at high altitude. *Int J Biometeorol* **58**, 1175-1179 (2014).

776. Khan J, Iiboshi Y, Cui L, Wasa M, Okada A. Role of intestinal mucus on the uptake of latex beads by Peyer's patches and on their transport to mesenteric lymph nodes in rats. *JPEN J Parenter Enteral Nutr* **23**, 19-23 (1999).

777. Khomenko T*, et al.* STAT3 and importins are novel mediators of early molecular and cellular responses in experimental duodenal ulceration. *Dig Dis Sci* **59**, 297-306 (2014).

778. Khurana RK, Gaspar BL, Welsby G, Katare OP, Singh KK, Singh B. Improving the biopharmaceutical attributes of mangiferin using vitamin E-TPGS co-loaded self-assembled phosholipidic nano-mixed micellar systems. *Drug Deliv Transl Res* **8**, 617-632 (2018).

779. Khurana S, Ganguly NK, Khullar M, Panigrahi D, Walia BN. Studies on the mechanism of Salmonella typhimurium enterotoxin-induced diarrhoea. *Biochim Biophys Acta* **1097**, 171-176 (1991).

780. Kilbinger H, Ginap T, Erbelding D. GABAergic inhibition of nitric oxide-mediated relaxation of guinea-pig ileum. *Naunyn Schmiedebergs Arch Pharmacol* **359**, 500-504 (1999).

781. Kim CH. Retinoic acid, immunity, and inflammation. *Vitam Horm* **86**, 83-101 (2011).

782. Kim H*, et al.* Clostridium difficile toxin A regulates inducible cyclooxygenase-2 and prostaglandin E2 synthesis in colonocytes via reactive oxygen species and activation of p38 MAPK. *J Biol Chem* **280**, 21237-21245 (2005).

783. Kim HB*, et al.* Prostaglandin E(2) Activates YAP and a Positive-Signaling Loop to Promote Colon Regeneration After Colitis but Also Carcinogenesis in Mice. *Gastroenterology* **152**, 616-630 (2017).

784. Kim JP*, et al.* Co-carcinogenic effects of several Korean foods on gastric cancer induced by N-methyl-N'-nitro-N-nitrosoguanidine in rats. *Jpn J Surg* **15**, 427-437 (1985).

785. Kim KT, Lee JY, Park JH, Cho HJ, Yoon IS, Kim DD. Capmul MCM/Solutol HS15-Based Microemulsion for Enhanced Oral Bioavailability of Rebamipide. *J Nanosci Nanotechnol* **17**, 2340-2344 (2017).

786. Kim MH, Lee GS, Jung EM, Choi KC, Jeung EB. The negative effect of dexamethasone on calcium-processing gene expressions is associated with a glucocorticoid-induced calcium-absorbing disorder. *Life Sci* **85**, 146-152 (2009).

787. Kim MH, Lee GS, Jung EM, Choi KC, Oh GT, Jeung EB. Dexamethasone differentially regulates renal and duodenal calcium-processing genes in calbindin-D9k and -D28k knockout mice. *Exp Physiol* **94**, 138-151 (2009).

788. Kim S, An BS, Yang H, Jeung EB. Effects of octylphenol and bisphenol A on the expression of calcium transport genes in the mouse duodenum and kidney during pregnancy. *Toxicology* **303**, 99-106 (2013).

789. Kim SJ, Kim MS. Inhibitory effects of cimicifugae rhizoma extracts on histamine, bradykinin and COX-2 mediated inflammatory actions. *Phytother Res* **14**, 596-600 (2000).

790. Kim SK*, et al.* Evaluation of absorption of heparin-DOCA conjugates on the intestinal wall using a surface plasmon resonance. *J Pharm Biomed Anal* **39**, 861-870 (2005).

791. Kimberg DV, Field M, Gershon E, Henderson A. Effects of prostaglandins and cholera enterotoxin on intestinal mucosal cyclic AMP accumulation. Evidence against an essential role for prostaglandins in the action of toxin. *J Clin Invest* **53**, 941-949 (1974).

792. Kimura RE, Reinersman GT. Intestinal glucose metabolism during development. II. The role of glucocorticoids and weaning. *Pediatr Res* **19**, 1313-1317 (1985).

793. Kiriliuk AG. [Effect of adrenaline and hydrocortisone on thiamine absorption in the intestine]. *Vopr Pitan*, 53-56 (1979).

794. Kissei M, Itoh T, Narawa T. Effect of epigallocatechin gallate on drug transport mediated by the proton-coupled folate transporter. *Drug Metab Pharmacokinet* **29**, 367-372 (2014).

795. Kistler EB, Hugli TE, Schmid-Schönbein GW. The pancreas as a source of cardiovascular cell activating factors. *Microcirculation* **7**, 183-192 (2000).

796. Kivilaakso E, Flemström G. HCO3- secretion and surface pH gradient in rat duodenum exposed to luminal acid. *Scand J Gastroenterol Suppl* **92**, 51-54 (1984).

797. Kiyose C, Muramatsu R, Fujiyama-Fujiwara Y, Ueda T, Igarashi O. Biodiscrimination of alpha-tocopherol stereoisomers during intestinal absorption. *Lipids* **30**, 1015-1018 (1995).

798. Kiziltaş S*, et al.* Corticosteroid therapy augments gastroduodenal permeability to sucrose. *Am J Gastroenterol* **93**, 2420-2425 (1998).

799. Kıroğlu OE, Aydinoglu F, Oğülener N. The effects of thiol modulators on nitrergic nerve- and S-nitrosothiols-induced relaxation in duodenum. *J Basic Clin Physiol Pharmacol* **24**, 143-150 (2013).

800. Klain GJ, Turnbull JD, Omaye ST. Oxidation of 1-14C-ascorbic acid in the guinea pig: effect of the route of administration. *Int J Vitam Nutr Res* **51**, 39-46 (1981).

801. Klemm K, Moody FG. Regional intestinal blood flow and nitric oxide synthase inhibition during sepsis in the rat. *Ann Surg* **227**, 126-133 (1998).

802. Klinger S, Breves G. Resveratrol Inhibits Porcine Intestinal Glucose and Alanine Transport: Potential Roles of Na⁺/K⁺-ATPase Activity, Protein Kinase A, AMP-Activated Protein Kinase and the Association of Selected Nutrient Transport Proteins with Detergent Resistant Membranes. *Nutrients* **10**, (2018).

803. Knight J, Taylor GW, Wright P, Clare AS, Rowley AF. Eicosanoid biosynthesis in an advanced deuterostomate invertebrate, the sea squirt (Ciona intestinalis). *Biochim Biophys Acta* **1436**, 467-478 (1999).

804. Knutson L, Knutson TW, Flemström G. Endogenous dopamine and duodenal bicarbonate secretion in humans. *Gastroenterology* **104**, 1409-1413 (1993).

805. Knutson L, Nimbratt C, Flemström G. Effects of leukotriene D4, the antagonist L-649-923, and arachidonic acid on duodenal bicarbonate secretion in the rat in vivo. *Scand J Gastroenterol* **23**, 1225-1231 (1988).

806. Kobayashi Y*, et al.* Green tea polyphenols inhibit the sodium-dependent glucose transporter of intestinal epithelial cells by a competitive mechanism. *J Agric Food Chem* **48**, 5618-5623 (2000).

807. Koch KL, Dwyer A. Effects of acetylsalicylic acid on electromechanical activity of in vivo rabbit ileum. *Dig Dis Sci* **33**, 962-968 (1988).

808. Koch KL, Dwyer A, Jeffries GH. Dose-response effects of indomethacin and PGE2 on electromechanical activity of in vivo rabbit ileum. *Am J Physiol* **250**, G135-139 (1986).

809. Koch TR, Fink JG, Ruan E, Petro A, Opara EC. Chronic glutathione depletion alters expression of enteric inhibitory neurochemicals in the mouse. *Neurosci Lett* **235**, 77-80 (1997).

810. Kochetygov NI, Gerbut KA, Remizova MI, Grishina GV. [Use of regulators of the nitric oxide synthesis in experimental hemorrhagic shock and its infusion therapy]. *Patol Fiziol Eksp Ter*, 35-39 (2011).

811. Kodentsova VM, Klimova OA, Sokol'nikov AA, Kha KM, Sergeev IN. [Enzymatic activity of the microsomal fraction of the mucosa of the small intestine in guinea pigs with vitamin D and C deficiencies]. *Vopr Pitan*, 41-45 (1988).

812. Koepsell H. The SLC22 family with transporters of organic cations, anions and zwitterions. *Mol Aspects Med* **34**, 413-435 (2013).

813. Koivusalo A*, et al.* Intraluminal casein model of necrotizing enterocolitis for assessment of mucosal destruction, bacterial translocation, and the effects of allopurinol and N-acetylcysteine. *Pediatr Surg Int* **18**, 712-717 (2002).

814. Kolb E. [Recent findings on the importance of vitamin A and its metabolism in man and laboratory animals]. *Z Gesamte Inn Med* **36**, 897-902 (1981).

815. Kolesnikova LI*, et al.* Oxidative Stress as a Mechanisms of Reduced Glucose Absorption under Conditions of Immobilization Stress. *Bull Exp Biol Med* **164**, 132-135 (2017).

816. Komatsu H, Ujiie A, Naito J. [Effect of tranilast on the release of slow reacting substance of anaphylaxis (SRS-A) and contraction of the smooth muscle]. *Nihon Yakurigaku Zasshi* **82**, 47-55 (1983).

817. Kondo Y*, et al.* Potato chip intake increases ascorbic acid levels and decreases reactive oxygen species in SMP30/GNL knockout mouse tissues. *J Agric Food Chem* **62**, 9286-9295 (2014).

818. Kong X, Wu G, Yin Y. Roles of phytochemicals in amino acid nutrition. *Front Biosci (Schol Ed)* **3**, 372-384 (2011).

819. Konturek SJ, Brzozowski T, Pytko-Polonczyk J, Drozdowicz D. Exogenous and endogenous cholecystokinin protects gastric mucosa against the damage caused by ethanol in rats. *Eur J Pharmacol* **273**, 57-62 (1995).

820. Konturek SJ, Obtulowicz W, Sito E, Oleksy J, Wilkon S, Kiec-Dembinska A. Distribution of prostaglandins in gastric and duodenal mucosa of healthy subjects and duodenal ulcer patients: effects of aspirin and paracetamol. *Gut* **22**, 283-289 (1981).

821. Konturek SJ, Tasler J, Bilski J, Kamińska A, Laskiewicz J. Role of prostaglandins in alkaline secretion from the gastroduodenal mucosa exposed to acid and taurocholate. *Scand J Gastroenterol Suppl* **92**, 69-74 (1984).

822. Konturek SJ, Tasler J, Bilski J, Kania J. Prostaglandins and alkaline secretion from oxyntic, antral, and duodenal mucosa of the dog. *Am J Physiol* **245**, G539-546 (1983).

823. Kopec RE, Gleize B, Borel P, Desmarchelier C, Caris-Veyrat C. Are lutein, lycopene, and β-carotene lost through the digestive process? *Food Funct* **8**, 1494-1503 (2017).

824. Korkmaz S, Maupoil V, Sobry C, Brunet C, Chevalier S, Freslon JL. An increased regional blood flow precedes mesenteric inflammation in rats treated by a phosphodiesterase 4 inhibitor. *Toxicol Sci* **107**, 298-305 (2009).

825. Kosik-Bogacka DI*, et al.* The inflammatory effect of infection with Hymenolepis diminuta via the increased expression and activity of COX-1 and COX-2 in the rat jejunum and colon. *Exp Parasitol* **169**, 69-76 (2016).

826. Kosmala M, Carter SR, Konturek SJ, Slomiany A, Slomiany BL. Mucus glycoprotein secretion by duodenal mucosa in response to luminal arachidonic acid. *Biochim Biophys Acta* **884**, 419-428 (1986).

827. Kostka P, Jang E, Watson EG, Stewart JL, Daniel EE. Nitric oxide synthase in the autonomic nervous system of canine ileum. *J Pharmacol Exp Ther* **264**, 234-239 (1993).

828. Koyama I*, et al.* Partial breakdown of glycated alkaline phosphatases mediated by reactive oxygen species. *Clin Chim Acta* **275**, 27-41 (1998).

829. Koyluoglu G*, et al.* Alterations in spontaneous contractions of rat ileum and jejunum after peritonitis. *Eur J Pharmacol* **580**, 250-255 (2008).

830. Köylüoğlu G, Kaya T, Bagcivan I, Yildiz T. Effect of L-NAME on decreased ileal muscle contractility induced by peritonitis in rats. *J Pediatr Surg* **37**, 901-905 (2002).

831. Koyuturk M, Bolkent S, Ozdil S, Arbak S, Yanardag R. The protective effect of vitamin C, vitamin E and selenium combination therapy on ethanol-induced duodenal mucosal injury. *Hum Exp Toxicol* **23**, 391-398 (2004).

832. Kozakova H, Hanson LA, Stepankova R, Kahu H, Dahlgren UI, Wiedermann U. Vitamin A deficiency leads to severe functional disturbance of the intestinal epithelium enzymes associated with diarrhoea and increased bacterial translocation in gnotobiotic rats. *Microbes Infect* **5**, 405-411 (2003).

833. Krakauer T, Buckley M. Doxycycline is anti-inflammatory and inhibits staphylococcal exotoxin-induced cytokines and chemokines. *Antimicrob Agents Chemother* **47**, 3630-3633 (2003).

834. Kreiss C, Birder LA, Kiss S, VanBibber MM, Bauer AJ. COX-2 dependent inflammation increases spinal Fos expression during rodent postoperative ileus. *Gut* **52**, 527-534 (2003).

835. Krimi RB, Letteron P, Chedid P, Nazaret C, Ducroc R, Marie JC. Resistin-like molecule-beta inhibits SGLT-1 activity and enhances GLUT2-dependent jejunal glucose transport. *Diabetes* **58**, 2032-2038 (2009).

836. Kubes P. Nitric oxide-induced microvascular permeability alterations: a regulatory role for cGMP. *Am J Physiol* **265**, H1909-1915 (1993).

837. Kubes P, Granger DN. Nitric oxide modulates microvascular permeability. *Am J Physiol* **262**, H611-615 (1992).

838. Kubota M, Ito Y, Domae M. Actions of prostaglandins and indomethacin on the electrical and mechanical properties of smooth muscle cells of the guinea-pig ileocecal junction. *Pflugers Arch* **394**, 347-354 (1982).

839. Kudo C*, et al.* The effects of nabumetone, a cyclooxygenase-2 inhibitor, on cisplatin-induced 5-hydroxytryptamine release from the isolated rat ileum. *Res Commun Mol Pathol Pharmacol* **110**, 117-132 (2001).

840. Kudsk KA. Effect of route and type of nutrition on intestine-derived inflammatory responses. *Am J Surg* **185**, 16-21 (2003).

841. Kuiken SD, Tytgat GN, Boeckxstaens GE. Role of endogenous nitric oxide in regulating antropyloroduodenal motility in humans. *Am J Gastroenterol* **97**, 1661-1667 (2002).

842. Kuiken SD, Vergeer M, Heisterkamp SH, Tytgat GN, Boeckxstaens GE. Role of nitric oxide in gastric motor and sensory functions in healthy subjects. *Gut* **51**, 212-218 (2002).

843. Kulkarni PS, Srinivasan BD. Synthesis of slow-reacting substance-like activity in human anterior uvea. *Invest Ophthalmol Vis Sci* **28**, 1201-1204 (1987).

844. Kulkarni PS, Srinivason BD. Synthesis of slow reacting substance-like activity in rabbit conjunctiva and anterior uvea. *Invest Ophthalmol Vis Sci* **24**, 1079-1085 (1983).

845. Kumar R, Eastwood AL, Brown ML, Laurie GW. Human genome search in celiac disease: mutated gliadin T-cell-like epitope in two human proteins promotes T-cell activation. *J Mol Biol* **319**, 593-602 (2002).

846. Kume H, Okazaki K, Takahashi T, Yamaji T. Protective effect of an immune-modulating diet comprising whey peptides and fermented milk products on indomethacin-induced small-bowel disorders in rats. *Clin Nutr* **33**, 1140-1146 (2014).

847. Kunikata T, Araki H, Takeeda M, Kato S, Takeuchi K. Prostaglandin E prevents indomethacin-induced gastric and intestinal damage through different EP receptor subtypes. *J Physiol Paris* **95**, 157-163 (2001).

848. Kuraishi Y, Kawabata S, Matsumoto T, Nakamura A, Fujita H, Satoh M. Involvement of substance P in hyperalgesia induced by intrathecal galanin. *Neurosci Res* **11**, 276-285 (1991).

849. Kurjak M*, et al.* Effect of GABA-ergic mechanisms on synaptosomal NO synthesis and the nitrergic component of NANC relaxation in rat ileum. *Neurogastroenterol Motil* **23**, e181-190 (2011).

850. Kurjak M, Fritsch R, Saur D, Schusdziarra V, Allescher HD. NO releases bombesin-like immunoreactivity from enteric synaptosomes by cross-activation of protein kinase A. *Am J Physiol* **276**, G1521-1530 (1999).

851. Kurjak M, Fritsch R, Saur D, Schusdziarra V, Allescher HD. Functional coupling between nitric oxide synthesis and VIP release within enteric nerve terminals of the rat: involvement of protein kinase G and phosphodiesterase 5. *J Physiol* **534**, 827-836 (2001).

852. Kurjak M, Hamel AM, Allescher HD, Schusdziarra V, Storr M. Differential stimulatory effects of cannabinoids on VIP release and NO synthase activity in synaptosomal fractions from rat ileum. *Neuropeptides* **42**, 623-632 (2008).

853. Kuroiwa K, Nelson JL, Boyce ST, Alexander JW, Ogle CK, Inoue S. Metabolic and immune effect of vitamin E supplementation after burn. *JPEN J Parenter Enteral Nutr* **15**, 22-26 (1991).

854. Labaronne E*, et al.* Low-dose pollutant mixture triggers metabolic disturbances in female mice leading to common and specific features as compared to a high-fat diet. *J Nutr Biochem* **45**, 83-93 (2017).

855. Labonté M, Couture P, Tremblay AJ, Hogue JC, Lemelin V, Lamarche B. Eicosapentaenoic and docosahexaenoic acid supplementation and inflammatory gene expression in the duodenum of obese patients with type 2 diabetes. *Nutr J* **12**, 98 (2013).

856. Lai CH, Lee CH, Hung CY, Lo HC. Oral Citrulline Mitigates Inflammation and Jejunal Damage via the Inactivation of Neuronal Nitric Oxide Synthase and Nuclear Factor-κB in Intestinal Ischemia and Reperfusion. *JPEN J Parenter Enteral Nutr* **41**, 422-435 (2017).

857. Lalles JP. Microbiota-host interplay at the gut epithelial level, health and nutrition. *J Anim Sci Biotechnol* **7**, 66 (2016).

858. Lallès JP. Microbiota-host interplay at the gut epithelial level, health and nutrition. *J Anim Sci Biotechnol* **7**, 66 (2016).

859. Lam WF, Gielkens HA, Coenraad M, Souverijn JH, Lamers CB, Masclee AA. Effect of insulin and glucose on basal and cholecystokinin-stimulated exocrine pancreatic secretion in humans. *Pancreas* **18**, 252-258 (1999).

860. Lam WF, Masclee AA, Souverijn JH, Lamers CB. Effect of acute hyperglycemia on basal, secretin and secretin + cholecystokinin stimulated exocrine pancreatic secretion in humans. *Life Sci* **64**, 617-626 (1999).

861. Lamarque D, Kiss J, Tankovic J, Flejou JF, Delchier JC, Whittle BJ. Induction of nitric oxide synthase in vivo and cell injury in rat duodenal epithelium by a water soluble extract of Helicobacter pylori. *Br J Pharmacol* **123**, 1073-1078 (1998).

862. Lambert JD*, et al.* Peracetylation as a means of enhancing in vitro bioactivity and bioavailability of epigallocatechin-3-gallate. *Drug Metab Dispos* **34**, 2111-2116 (2006).

863. Lambrecht W, Weinland G, Koch G, Gaedicke G. [Treatment of meconium ileus equivalent]. *Chirurg* **49**, 410-413 (1978).

864. Lampropoulou V*, et al.* Itaconate Links Inhibition of Succinate Dehydrogenase with Macrophage Metabolic Remodeling and Regulation of Inflammation. *Cell Metab* **24**, 158-166 (2016).

865. Lanas A*, et al.* Non-variceal upper gastrointestinal bleeding. *Nat Rev Dis Primers* **4**, 18020 (2018).

866. Lane DJ, Bae DH, Merlot AM, Sahni S, Richardson DR. Duodenal cytochrome b (DCYTB) in iron metabolism: an update on function and regulation. *Nutrients* **7**, 2274-2296 (2015).

867. Lane HW, Shirley RL, Cerda JJ. Glutathione peroxidase acitvity in intestinal and liver tissues of rats fed various levels of selenium, sulfur and alpha-tocopherol. *J Nutr* **109**, 444-452 (1979).

868. Langford AM, Pounder DJ. Possible markers for postmortem drug redistribution. *J Forensic Sci* **42**, 88-92 (1997).

869. Lanza F, Peace K, Gustitus L, Rack MF, Dickson B. A blinded endoscopic comparative study of misoprostol versus sucralfate and placebo in the prevention of aspirin-induced gastric and duodenal ulceration. *Am J Gastroenterol* **83**, 143-146 (1988).

870. Lanza FL. A double-blind study of prophylactic effect of misoprostol on lesions of gastric and duodenal mucosa induced by oral administration of tolmetin in healthy subjects. *Dig Dis Sci* **31**, 131s-136s (1986).

871. Lanza FL, Robinson MG, Isenberg JI, Basuk PM, Karlin DA. Effect of enprostil on the gastroduodenal mucosa of healthy volunteers. *Aliment Pharmacol Ther* **4**, 601-613 (1990).

872. Larrosa M, Azorin-Ortuno M, Yanez-Gascon MJ, Garcia-Conesa MT, Tomas-Barberan F, Espin JC. Lack of effect of oral administration of resveratrol in LPS-induced systemic inflammation. *Eur J Nutr* **50**, 673-680 (2011).

873. Larsen IS*, et al.* Human Paneth cell α-defensin-5 treatment reverses dyslipidemia and improves glucoregulatory capacity in diet-induced obese mice. *Am J Physiol Endocrinol Metab* **317**, E42-e52 (2019).

874. Larsen R, Hansen MB, Bindslev N. Duodenal secretion in humans mediated by the EP4 receptor subtype. *Acta Physiol Scand* **185**, 133-140 (2005).

875. Larsen TW, Yang A, Tume RK. The in vitro destruction of rumen fluid carotenoids by plant lipoxygenases. *Biochem Mol Biol Int* **30**, 197-207 (1993).

876. Larsson D, Anderson D, Smith NM, Nemere I. 24,25-dihydroxyvitamin D3 binds to catalase. *J Cell Biochem* **97**, 1259-1266 (2006).

877. Larussa T, Imeneo M, Luzza F. Potential role of nutraceutical compounds in inflammatory bowel disease. *World J Gastroenterol* **23**, 2483-2492 (2017).

878. László F, Morschl E, Pávó I, Whittle BJ. Nitric oxide modulates the gastrointestinal plasma extravasation following intraabdominal surgical manipulation in rats. *Eur J Pharmacol* **375**, 211-215 (1999).

879. László F, Whittle BJ. Constitutive nitric oxide modulates the injurious actions of vasopressin on rat intestinal microcirculation in acute endotoxaemia. *Eur J Pharmacol* **260**, 265-268 (1994).

880. László F, Whittle BJ, Moncada S. Attenuation by nitrosothiol NO donors of acute intestinal microvascular dysfunction in the rat. *Br J Pharmacol* **115**, 498-502 (1995).

881. Lattimer JM, Haub MD. Effects of dietary fiber and its components on metabolic health. *Nutrients* **2**, 1266-1289 (2010).

882. Laudanno OM, Finkelstein D, Capdepon E. [Prostaglandin E1 (misoprostol) and S-adenosylmethionine in the prevention of hemorrhagic gastritis induced by aspirin in the human. Endoscopic, histologic and histochemical study]. *Acta Gastroenterol Latinoam* **14**, 289-293 (1984).

883. Lautenschläger I*, et al.* Quinidine, but not eicosanoid antagonists or dexamethasone, protect the gut from platelet activating factor-induced vasoconstriction, edema and paralysis. *PLoS One* **10**, e0120802 (2015).

884. Lawson LD, Powell DW. Bradykinin-stimulated eicosanoid synthesis and secretion by rabbit ileal components. *Am J Physiol* **252**, G783-790 (1987).

885. Lazarov J. Effects of adrenalectomy and prednisolone on the absorption and phosphorylation of thiamine in rats. *J Endocrinol* **76**, 385-389 (1978).

886. Lazarov Y, Stanchev H, Piperova L. Absorption of 35S-thiamine in jejunum and its brain and liver content in chickens on different selenium level diets. *Rev Esp Fisiol* **36**, 377-381 (1980).

887. Lazuga A, Szember B, Wawrzeńska M, Nagórna-Stasiak B. [Effect of vitamin C on cholesterol content of the intestinal wall in rabbits]. *Pol Arch Weter* **23**, 39-47 (1982).

888. Leander P, Golman K, Månsson S, Höglund P. Orally administered manganese with and without ascorbic acid as a liver-specific contrast agent and bowel marker for magnetic resonance imaging: phase I clinical trial assessing efficacy and safety. *Invest Radiol* **45**, 559-564 (2010).

889. Lebenthal E, Sunshine P, Kretchmer N. Effect of carbohydrate and corticosteroids on activity of -glucosidases in intestine of the infant rat. *J Clin Invest* **51**, 1244-1250 (1972).

890. Lebrun LJ*, et al.* Enteroendocrine L Cells Sense LPS after Gut Barrier Injury to Enhance GLP-1 Secretion. *Cell Rep* **21**, 1160-1168 (2017).

891. Lecleire S*, et al.* Modulation of nitric oxide and cytokines production by L-arginine in human gut mucosa. *Clin Nutr* **24**, 353-359 (2005).

892. Lee SI, Kang KS. Function of capric acid in cyclophosphamide-induced intestinal inflammation, oxidative stress, and barrier function in pigs. *Sci Rep* **7**, 16530 (2017).

893. Lee SY, Belmonte AA. Effect of sodium deoxycholate on the dissolution and absorption of copper-indomethacin and zinc-indomethacin. *J Pharm Sci* **83**, 1107-1109 (1994).

894. Lee Y, Kim H, Kim W, Yoon JH, Jeong SH, Jung Y. Colon-specific delivery of celecoxib is a potential strategy to improve toxicological and pharmacological properties of the selective Cox-2 inhibitor: implication in treatment of familiar adenomatous polyposis. *J Drug Target* **20**, 524-534 (2012).

895. Leech B, Schloss J, Steel A. Treatment Interventions for the Management of Intestinal Permeability: A Cross-Sectional Survey of Complementary and Integrative Medicine Practitioners. *J Altern Complement Med* **25**, 623-636 (2019).

896. Leelakusolvong S, Sarr MG, Miller SM, Phillips SF, Bharucha AE. Role of extrinsic innervation in modulating nitrergic transmission in the canine ileocolonic region. *Am J Physiol Gastrointest Liver Physiol* **283**, G230-239 (2002).

897. Leeper LL, Henning SJ. Hormonal control of postnatal development of ileal neuraminidase and acid beta-galactosidase. *Biol Neonate* **44**, 28-35 (1983).

898. Leeper LL, McDonald MC, Heath JP, Henning SJ. Sucrase-isomaltase ontogeny: synergism between glucocorticoids and thyroxine reflects increased mRNA and no change in cell migration. *Biochem Biophys Res Commun* **246**, 765-770 (1998).

899. Legat FJ, Griesbacher T, Lembeck F. CP-96,345, a non-peptide antagonist of substance P: I. Effects on the actions mediated by substance P and related tachykinins on the guinea-pig ileum and rabbit jejunum. *Naunyn Schmiedebergs Arch Pharmacol* **346**, 315-322 (1992).

900. Legen I, Zakelj S, Kristl A. Polarised transport of monocarboxylic acid type drugs across rat jejunum in vitro: the effect of mucolysis and ATP-depletion. *Int J Pharm* **256**, 161-166 (2003).

901. Lehmann C*, et al.* Intravenous free and dipeptide-bound glutamine maintains intestinal microcirculation in experimental endotoxemia. *Nutrition* **28**, 588-593 (2012).

902. Leitão RF*, et al.* Role of inducible nitric oxide synthase pathway on methotrexate-induced intestinal mucositis in rodents. *BMC Gastroenterol* **11**, 90 (2011).

903. Lénárd L, Jr., Halmai V, Barthó L. Morphine contracts the guinea pig ileal circular muscle by interfering with a nitric oxide mediated tonic inhibition. *Digestion* **60**, 562-566 (1999).

904. Leng S*, et al.* Hepatocyte nuclear factor-4 mediates apolipoprotein A-IV transcriptional regulation by fatty acid in newborn swine enterocytes. *Am J Physiol Gastrointest Liver Physiol* **293**, G475-483 (2007).

905. Lennernäs H, Ahrenstedt O, Hällgren R, Knutson L, Ryde M, Paalzow LK. Regional jejunal perfusion, a new in vivo approach to study oral drug absorption in man. *Pharm Res* **9**, 1243-1251 (1992).

906. Leocádio PC*, et al.* L-arginine pretreatment reduces intestinal mucositis as induced by 5-FU in mice. *Nutr Cancer* **67**, 486-493 (2015).

907. Leppert PS, Fix JA. Use of everted intestinal rings for in vitro examination of oral absorption potential. *J Pharm Sci* **83**, 976-981 (1994).

908. Lesueur C, Bôle-Feysot C, Bekri S, Husson A, Lavoinne A, Brasse-Lagnel C. Glutamine induces nuclear degradation of the NF-κB p65 subunit in Caco-2/TC7 cells. *Biochimie* **94**, 806-815 (2012).

909. Leung FW, Iwata F, Kao J, Seno K, Itoh M, Leung JW. Ruthenium red-sensitive cation channels, but not calcitonin gene-related peptide or substance P-mediated mechanisms, protect duodenal villi against acid-induced damage. *Life Sci* **71**, 2617-2624 (2002).

910. Leung FW, Iwata F, Kao J, Seno K, Itoh M, Leung JW. Role of bradykinin in acid-induced mesenteric hyperemia and duodenal villous damage. *Life Sci* **70**, 779-790 (2002).

911. Leung K. Hyperpolarized [1-(13)C]dehydroascorbic acid. In: *Molecular Imaging and Contrast Agent Database (MICAD)*). National Center for Biotechnology Information (US) (2004).

912. Levens NR, Peach MJ, Carey RM, Poat JA, Munday KA. Response of rat jejunum to angiotensin II: role of norepinephrine and prostaglandins. *Am J Physiol* **240**, G17-24 (1981).

913. Levy B, Mansart A, Bollaert PE, Franck P, Mallie JP. Effects of epinephrine and norepinephrine on hemodynamics, oxidative metabolism, and organ energetics in endotoxemic rats. *Intensive Care Med* **29**, 292-300 (2003).

914. Lewis RA, Austen KF. Molecular determinants for functional responses to the sulfidopeptide leukotrienes: metabolism and receptor subclasses. *J Allergy Clin Immunol* **74**, 369-372 (1984).

915. Lexhaller B, Ludwig C, Scherf KA. Comprehensive Detection of Isopeptides between Human Tissue Transglutaminase and Gluten Peptides. *Nutrients* **11**, (2019).

916. Lheureux O, Preiser JC. Year in review 2013: Critical Care--metabolism. *Crit Care* **18**, 571 (2014).

917. Li B*, et al.* Effects of inulin supplementation to piglets in the suckling period on growth performance, postileal microbial and immunological traits in the suckling period and three weeks after weaning. *Arch Anim Nutr* **72**, 425-442 (2018).

918. Li C, Zhu Y, Shenoy M, Pai R, Liu L, Pasricha PJ. Anatomical and functional characterization of a duodeno-pancreatic neural reflex that can induce acute pancreatitis. *Am J Physiol Gastrointest Liver Physiol* **304**, G490-500 (2013).

919. Li JY*, et al.* [Change in intestinal function in sepsis in rat]. *Zhongguo Wei Zhong Bing Ji Jiu Yi Xue* **16**, 352-354 (2004).

920. Li L, Somerset S. Digestive system dysfunction in cystic fibrosis: challenges for nutrition therapy. *Dig Liver Dis* **46**, 865-874 (2014).

921. Li N*, et al.* Butyrate and type 1 diabetes mellitus: can we fix the intestinal leak? *J Pediatr Gastroenterol Nutr* **51**, 414-417 (2010).

922. Li N*, et al.* Glutamine decreases lipopolysaccharide-induced intestinal inflammation in infant rats. *Am J Physiol Gastrointest Liver Physiol* **286**, G914-921 (2004).

923. Li N*, et al.* Spatial heterogeneity of bacterial colonization across different gut segments following inter-species microbiota transplantation. *Microbiome* **8**, 161 (2020).

924. Li Q, Schmitz-Esser S, Loving CL, Gabler NK, Gould SA, Patience JF. Exogenous carbohydrases added to a starter diet reduced markers of systemic immune activation and decreased Lactobacillus in weaned pigs1. *J Anim Sci* **97**, 1242-1253 (2019).

925. Li R*, et al.* Effect of exposure to atmospheric ultrafine particles on production of free fatty acids and lipid metabolites in the mouse small intestine. *Environ Health Perspect* **123**, 34-41 (2015).

926. Li R*, et al.* β-carotene attenuates weaning-induced apoptosis via inhibition of PERK-CHOP and IRE1-JNK/p38 MAPK signalling pathways in piglet jejunum. *J Anim Physiol Anim Nutr (Berl)* **104**, 280-290 (2020).

927. Li T, Chiang JYL. Bile acid-based therapies for non-alcoholic steatohepatitis and alcoholic liver disease. *Hepatobiliary Surg Nutr* **9**, 152-169 (2020).

928. Li X, Li S, Chen M, Wang J, Xie B, Sun Z. (-)-Epigallocatechin-3-gallate (EGCG) inhibits starch digestion and improves glucose homeostasis through direct or indirect activation of PXR/CAR-mediated phase II metabolism in diabetic mice. *Food Funct* **9**, 4651-4663 (2018).

929. Li XH*, et al.* Delta-tocotrienol protects mice from radiation-induced gastrointestinal injury. *Radiat Res* **180**, 649-657 (2013).

930. Li Y, Song Z, Kerr KA, Moeser AJ. Chronic social stress in pigs impairs intestinal barrier and nutrient transporter function, and alters neuro-immune mediator and receptor expression. *PLoS One* **12**, e0171617 (2017).

931. Li Y, Wang X, Li N, Li J. The study of n-3PUFAs protecting the intestinal barrier in rat HS/R model. *Lipids Health Dis* **13**, 146 (2014).

932. Li YF, Weisbrodt NW, Lodato RF, Moody FG. Nitric oxide is involved in muscle relaxation but not in changes in short-circuit current in rat ileum. *Am J Physiol* **266**, G554-559 (1994).

933. Liang S*, et al.* The Comparative Study of the Therapeutic Effects and Mechanism of Baicalin, Baicalein, and Their Combination on Ulcerative Colitis Rat. *Front Pharmacol* **10**, 1466 (2019).

934. Liang T*, et al.* Roles of Sphincter of Oddi Laxity in Bile Duct Microenvironment in Patients with Cholangiolithiasis: From the Perspective of the Microbiome and Metabolome. *J Am Coll Surg* **222**, 269-280.e210 (2016).

935. Liang Y, Yu B, Wang Y, Qiao Z, Cao T, Zhang P. Duodenal long noncoding RNAs are associated with glycemic control after bariatric surgery in high-fat diet-induced diabetic mice. *Surg Obes Relat Dis* **13**, 1212-1226 (2017).

936. Liang YC, Liu HJ, Chen SH, Chen CC, Chou LS, Tsai LH. Effect of lipopolysaccharide on diarrhea and gastrointestinal transit in mice: roles of nitric oxide and prostaglandin E2. *World J Gastroenterol* **11**, 357-361 (2005).

937. Lichtenberger LM, Dial EJ, Romero JJ, Lechago J, Jarboe LA, Wolfe MM. Role of luminal ammonia in the development of gastropathy and hypergastrinemia in the rat. *Gastroenterology* **108**, 320-329 (1995).

938. Lillemoe KD, Johnson LF, Harmon JW. Alkaline esophagitis: a comparison of the ability of components of gastroduodenal contents to injure the rabbit esophagus. *Gastroenterology* **85**, 621-628 (1983).

939. Lin J, Gao XN, Yan GT, Xue H, Hao XH, Wang LH. Endogenous leptin fluctuates in hepatic ischemia/reperfusion injury and represents a potential therapeutic target. *World J Gastroenterol* **16**, 5424-5434 (2010).

940. Linard C, Marquette C, Strup C, Aigueperse J, Mathe D. Involvement of primary afferent nerves after abdominal irradiation: consequences on ileal contractile activity and inflammatory mediator release in the rat. *Dig Dis Sci* **48**, 688-697 (2003).

941. Lincoln J, Messersmith WA. Conditions required for the measurement of nitric oxide synthase activity in a myenteric plexus/smooth muscle preparation from the rat ileum. *J Neurosci Methods* **59**, 191-197 (1995).

942. Lindahl A*, et al.* Jejunal permeability and hepatic extraction of fluvastatin in humans. *Clin Pharmacol Ther* **60**, 493-503 (1996).

943. Lindley KJ, Goss-Sampson MA, Muller DP, Milla PJ. Lipid peroxidation and electrogenic ion transport in the jejunum of the vitamin E deficient rat. *Gut* **35**, 34-39 (1994).

944. Lindqvist A, Sharvill J, Sharvill DE, Andersson S. Loss-of-function mutation in carotenoid 15,15'-monooxygenase identified in a patient with hypercarotenemia and hypovitaminosis A. *J Nutr* **137**, 2346-2350 (2007).

945. Lippert E*, et al.* Regulation of galectin-3 function in mucosal fibroblasts: potential role in mucosal inflammation. *Clin Exp Immunol* **152**, 285-297 (2008).

946. Lisboa MJS*, et al.* Supplementation action with ascorbic acid in the morphology of the muscular layer and reactive acetylcholinesterase neurons of ileum of mdx mice. *Auton Neurosci* **205**, 57-66 (2017).

947. Little TJ*, et al.* Plasma endocannabinoid levels in lean, overweight, and obese humans: relationships to intestinal permeability markers, inflammation, and incretin secretion. *Am J Physiol Endocrinol Metab* **315**, E489-e495 (2018).

948. Liu CY, Mueller MH, Grundy D, Kreis ME. Vagal modulation of intestinal afferent sensitivity to systemic LPS in the rat. *Am J Physiol Gastrointest Liver Physiol* **292**, G1213-1220 (2007).

949. Liu H*, et al.* New synthetic routes to chain-extended selenium, sulfur, and nitrogen analogues of the naturally occurring glucosidase inhibitor salacinol and their inhibitory activities against recombinant human maltase glucoamylase. *J Org Chem* **72**, 6562-6572 (2007).

950. Liu HP*, et al.* Chemoprevention of intestinal adenomatous polyposis by acetyl-11-keto-beta-boswellic acid in APC(Min/+) mice. *Int J Cancer* **132**, 2667-2681 (2013).

951. Liu J*, et al.* Beneficial effects of butyrate in intestinal injury. *J Pediatr Surg* **55**, 1088-1093 (2020).

952. Liu L, Song Z, Sheikhahmadi A, Jiao H, Lin H. Effect of corticosterone on gene expression of feed intake regulatory peptides in laying hens. *Comp Biochem Physiol B Biochem Mol Biol* **162**, 81-87 (2012).

953. Liu S*, et al.* Sodium butyrate inhibits the production of HMGB1 and attenuates severe burn plus delayed resuscitation-induced intestine injury via the p38 signaling pathway. *Burns* **45**, 649-658 (2019).

954. Liu SX, Huang ZH. The value of radionuclide hepatobiliary scintigraphy in combination with determination of bilirubin from duodenal drainage in differential diagnosis of infantile persistent jaundice. *Front Med China* **4**, 342-345 (2010).

955. Liu X, Wang P, Zou YX, Luo ZG, Tamer TM. Co-encapsulation of Vitamin C and β-Carotene in liposomes: Storage stability, antioxidant activity, and in vitro gastrointestinal digestion. *Food Res Int* **136**, 109587 (2020).

956. Liu Z, Zheng X, Guo Y, Qin W, Hua L, Yang Y. Quantitatively metabolic profiles of salvianolic acids in rats after gastric-administration of Salvia miltiorrhiza extract. *Fitoterapia* **113**, 27-34 (2016).

957. Liu ZY, Wang XL, Ou SQ, Hou DX, He JH. Sanguinarine modulate gut microbiome and intestinal morphology to enhance growth performance in broilers. *PLoS One* **15**, e0234920 (2020).

958. Liyanage DS*, et al.* Identification of thioredoxin domain-containing protein 17 from big-belly seahorse Hippocampus abdominalis: Molecular insights, immune responses, and functional characterization. *Fish Shellfish Immunol* **86**, 301-310 (2019).

959. Löbermann M, Hemmer C, Andree H, Mau R, Grimm K, Reisinger EC. [Factitious recurrent septicemia and gastrointestinal corrosive burns]. *Dtsch Med Wochenschr* **135**, 1368-1371 (2010).

960. Loeschke K*, et al.* Bacterial overgrowth in ileal reservoirs (Koch pouch): extended functional studies. *Hepatogastroenterology* **27**, 310-316 (1980).

961. Loirdighi N, Ménard D, Delvin D, Levy E. Selective effects of hydrocortisone on intestinal lipoprotein and apolipoprotein synthesis in the human fetus. *J Cell Biochem* **66**, 65-76 (1997).

962. Lonovics J, Jakab I, Szilvássy J, Szilvássy Z. Regional differences in nitric oxide-mediated relaxation of the rabbit sphincter of Oddi. *Eur J Pharmacol* **255**, 117-122 (1994).

963. Lopes A*, et al.* Evaluation of the effects of fructose on oxidative stress and inflammatory parameters in rat brain. *Mol Neurobiol* **50**, 1124-1130 (2014).

964. Lopez Y, Fioramonti J, Bueno L. Action of endogenous prostaglandins on postprandial pyloric motility: a possible modulation by fats. *Prostaglandins* **42**, 313-320 (1991).

965. Lördal M, Hällgren A, Nylander O, Hellström PM. Tachykinins increase vascular permeability in the gastrointestinal tract of the rat. *Acta Physiol Scand* **156**, 489-494 (1996).

966. Loven DP, Schedl HP, Oberley LW, Wilson HD, Bruch L, Niehaus CL. Superoxide dismutase activity in the intestine of the streptozotocin-diabetic rat. *Endocrinology* **111**, 737-742 (1982).

967. Lowe PP*, et al.* Reduced gut microbiome protects from alcohol-induced neuroinflammation and alters intestinal and brain inflammasome expression. *J Neuroinflammation* **15**, 298 (2018).

968. Lu H*, et al.* Glucuronides of tea catechins: enzymology of biosynthesis and biological activities. *Drug Metab Dispos* **31**, 452-461 (2003).

969. Lu HH, Thomas J, Fleisher D. Influence of D-glucose-induced water absorption on rat jejunal uptake of two passively absorbed drugs. *J Pharm Sci* **81**, 21-25 (1992).

970. Lu R, Zhang YG, Xia Y, Sun J. Imbalance of autophagy and apoptosis in intestinal epithelium lacking the vitamin D receptor. *Faseb j* **33**, 11845-11856 (2019).

971. Lubrano R*, et al.* [Intestinal absorption of vitamin E in children with atrophy of the jejunal mucosa]. *Minerva Pediatr* **42**, 537-539 (1990).

972. Lugea A, Mourelle M, Guarner F, Domingo A, Salas A, Malagelada JR. Phosphatidylcholines as mediators of adaptive cytoprotection of the rat duodenum. *Gastroenterology* **107**, 720-727 (1994).

973. Lugea A, Salas A, Guarner F, Azpiroz F, Malagelada JR. Duodenal mucosal resistance to intraluminal acid in the rat: role of adaptive cytoprotection. *Gastroenterology* **102**, 1129-1135 (1992).

974. Lugea A, Salas A, Guarner F, Malagelada JR. Influence of dietary fat on duodenal resistance to acid. *Gut* **34**, 1303-1309 (1993).

975. Lugea A, Salas A, Guarner F, Malagelada JR. Adaptive cytoprotection of the rat duodenum is not dependent on nitric oxide-induced changes in blood flow. *Am J Physiol* **264**, G994-1000 (1993).

976. Lundåsen T*, et al.* The PPAR pan-agonist tetradecylthioacetic acid promotes redistribution of plasma cholesterol towards large HDL. *PLoS One* **15**, e0229322 (2020).

977. Lunn JC*, et al.* The effect of haem in red and processed meat on the endogenous formation of N-nitroso compounds in the upper gastrointestinal tract. *Carcinogenesis* **28**, 685-690 (2007).

978. Luo M*, et al.* Prospective analysis of serum carotenoids, vitamin A, and tocopherols in adults with short bowel syndrome undergoing intestinal rehabilitation. *Nutrition* **25**, 400-407 (2009).

979. Luo Z, Morgan MR, Day AJ. Transport of trans-tiliroside (kaempferol-3-β-D-(6"-p-coumaroyl-glucopyranoside) and related flavonoids across Caco-2 cells, as a model of absorption and metabolism in the small intestine. *Xenobiotica* **45**, 722-730 (2015).

980. Luz ABS*, et al.* Adipocytes and intestinal epithelium dysfunctions linking obesity to inflammation induced by high glycemic index pellet-diet in Wistar rats. *Biosci Rep* **38**, (2018).

981. Ma LM, Xu F, Wang JZ, Shang MY, Liu GX, Cai SQ. In vivo metabolism of 8,2'-diprenylquercetin 3-methyl ether and the distribution of its metabolites in rats by HPLC-ESI-IT-TOF-MS(n). *Fitoterapia* **137**, 104191 (2019).

982. Ma LQ, Hu HZ, Tian Q, Wang CD, Wang XM, Yu CG. [Effect of beta-adrenoceptor on NO-induced attenuation in spontaneous contractions of ileum in mice]. *Zhongguo Ying Yong Sheng Li Xue Za Zhi* **23**, 111-115 (2007).

983. MacDonald L, Thumser AE, Sharp P. Decreased expression of the vitamin C transporter SVCT1 by ascorbic acid in a human intestinal epithelial cell line. *Br J Nutr* **87**, 97-100 (2002).

984. Machado ER*, et al.* Cyclooxygenase-derived mediators regulate the immunological control of Strongyloides venezuelensis infection. *FEMS Immunol Med Microbiol* **59**, 18-32 (2010).

985. Macherey HJ, Petersen KU. Rapid decrease in electrical conductance of mammalian duodenal mucosa in vitro. Combined effects of prostaglandin E2 and bicarbonate. *Gastroenterology* **97**, 1448-1460 (1989).

986. Macherey HJ, Petersen KU. Acid-induced increase in electrical conductance of guinea pig duodenal mucosa in vitro. Temporary protection by combined effects of bicarbonate and prostaglandin E2. *Gastroenterology* **100**, 648-662 (1991).

987. Macherey HJ, Sprakties G, Petersen KU. HCO3- reduces paracellular permeability of guinea pig duodenal mucosa by a Ca2+ (prostaglandin)-dependent action. *Am J Physiol* **264**, G126-136 (1993).

988. MacLeod RJ, Lembessis P, Hamilton JR, Powell WS. 5-Oxo-6,8,11,14-eicosatetraenoic acid stimulates isotonic volume reduction of guinea pig jejunal crypt epithelial cells. *J Pharmacol Exp Ther* **291**, 511-516 (1999).

989. MacNaughton WK, Gall DG. Mechanisms of platelet-activating factor-induced electrolyte transport in the rat jejunum. *Eur J Pharmacol* **200**, 17-23 (1991).

990. MacNaughton WK, Leach KE, Prud'homme-Lalonde L, Harding RK. Exposure to ionizing radiation increases responsiveness to neural secretory stimuli in the ferret jejunum in vitro. *Int J Radiat Biol* **72**, 219-226 (1997).

991. Madeddu P*, et al.* Role of calcitonin gene-related peptide and kinins in post-ischemic intestinal reperfusion. *Peptides* **22**, 915-922 (2001).

992. Mader R, Adawi M, Schonfeld S. Malabsorption in systemic lupus erythematosus. *Clin Exp Rheumatol* **15**, 659-661 (1997).

993. Madero M, Perez-Pozo SE, Jalal D, Johnson RJ, Sánchez-Lozada LG. Dietary fructose and hypertension. *Curr Hypertens Rep* **13**, 29-35 (2011).

994. Madesh M, Anup R, Benard O, Balasubramanian KA. Apoptosis in the monkey small intestinal epithelium: structural and functional alterations in the mitochondria. *Free Radic Biol Med* **26**, 836-843 (1999).

995. Madsen JE, Vetvik K, Aase S. Helicobacter-associated duodenitis and gastric metaplasia in duodenal ulcer patients. *Apmis* **99**, 997-1000 (1991).

996. Maeda S, Maeda S, Shibata S, Chimura N, Fukata T. Molecular cloning of canine protease-activated receptor-2 and its expression in normal dog tissues and atopic skin lesions. *J Vet Med Sci* **71**, 577-582 (2009).

997. Maeda S*, et al.* Molecular cloning and expression analysis of the canine chemokine receptor CCR9. *Vet Immunol Immunopathol* **145**, 534-539 (2012).

998. Maeda S, Okayama T, Ohmori K, Masuda K, Ohno K, Tsujimoto H. Molecular cloning of the feline thymus and activation-regulated chemokine cDNA and its expression in lesional skin of cats with eosinophilic plaque. *J Vet Med Sci* **65**, 275-278 (2003).

999. Maggi CA, Patacchini R, Meini S, Giuliani S. Nitric oxide is the mediator of tachykinin NK3 receptor-induced relaxation in the circular muscle of the guinea-pig ileum. *Eur J Pharmacol* **240**, 45-50 (1993).

1000. Maggi CA, Patacchini R, Meini S, Giuliani S. Evidence for the presence of a septide-sensitive tachykinin receptor in the circular muscle of the guinea-pig ileum. *Eur J Pharmacol* **235**, 309-311 (1993).

1001. Magnusson KE, Dahlgren C, Sjölander A. Effect of N-formylated methionyl-phenylalanine (FMP) and methionyl-leucyl-phenylalanine (FMLP) on gut permeability. A model of local inflammatory process. *Inflammation* **9**, 365-373 (1985).

1002. Magro F, Fraga S, Ribeiro T, Soares-da-Silva P. Decreased availability of intestinal dopamine in transmural colitis may relate to inhibitory effects of interferon-gamma upon L-DOPA uptake. *Acta Physiol Scand* **180**, 379-386 (2004).

1003. Maher MM, Gontarek JD, Jimenez RE, Cahill PA, Yeo CJ. Endogenous nitric oxide promotes ileal absorption. *J Surg Res* **58**, 687-692 (1995).

1004. Mahmood A, Torres-Pinedo R. Effect of hormone administration on the sialylation and fucosylation of intestinal microvillus membranes of suckling rats. *Pediatr Res* **19**, 899-902 (1985).

1005. Mahmoud MS, Habib FS. Role of nitric oxide in host defense against Hymenolepis nana infection. *J Egypt Soc Parasitol* **33**, 485-496 (2003).

1006. Mähr G. [Chemical Investigation of Chronic Pancreatitis]. *MMW Munch Med Wochenschr* **118**, 409-414 (1976).

1007. Maier S*, et al.* Epoprostenol improves mucosal tissue oxygen tension in an acute endotoxemic pig model. *Shock* **31**, 104-110 (2009).

1008. Mailman D. Differential effects of lumenal L-arginine and NG-nitro L-arginine on blood flow and water fluxes in rat ileum. *Br J Pharmacol* **112**, 304-310 (1994).

1009. Mailman D. Ileal luminal nitric oxide synthase inhibitors and E. coli lipopolysaccharide effects in the anesthetized rat. *Dig Dis Sci* **47**, 190-200 (2002).

1010. Maish GO, 3rd, Shumate ML, Ehrlich HP, Cooney RN. Tumor necrosis factor binding protein improves incisional wound healing in sepsis. *J Surg Res* **78**, 108-117 (1998).

1011. Makino E*, et al.* Potent inhibitory activity of HSR-6071, a new antiallergic agent, on passive cutaneous anaphylaxis (PCA). *Jpn J Pharmacol* **52**, 87-94 (1990).

1012. Makino T, Kanemaru M, Okuyama S, Shimizu R, Tanaka H, Mizukami H. Anti-allergic effects of enzymatically modified isoquercitrin (α-oligoglucosyl quercetin 3-O-glucoside), quercetin 3-O-glucoside, α-oligoglucosyl rutin, and quercetin, when administered orally to mice. *J Nat Med* **67**, 881-886 (2013).

1013. Malekinejad H, Cheraghi H, Alizadeh A, Khadem-Ansari MH, Tehrani AA, Varasteh S. Nitric oxide and acute phase proteins are involved in pathogenesis of mycophenolate mofetil-induced gastrointestinal disorders in rats. *Transplant Proc* **43**, 2741-2746 (2011).

1014. Malmezat T, Breuillé D, Capitan P, Mirand PP, Obled C. Glutathione turnover is increased during the acute phase of sepsis in rats. *J Nutr* **130**, 1239-1246 (2000).

1015. Malmström RE*, et al.* Intestinal nitric oxide in the normal and endotoxemic pig. *Shock* **18**, 456-460 (2002).

1016. Malo C, Ménard D. Opposite effects of one and three injections of cortisone or thyroxine on intestinal lactase activity in suckling mice. *Experientia* **35**, 493-494 (1979).

1017. Maltas J, Wood KB. Pervasive and diverse collateral sensitivity profiles inform optimal strategies to limit antibiotic resistance. *PLoS Biol* **17**, e3000515 (2019).

1018. Maltbaek JS*, et al.* Secretory response to cholera toxin in the porcine jejunum under different types of general anaesthesia. *Exp Physiol* **83**, 523-531 (1998).

1019. Mameya S, Sawa T, Taniyama K. Arachidonic acid cascade and stimulation of acetylcholine release by human recombinant interleukin-1 beta in guinea pig ileum. *J Pharmacol Exp Ther* **275**, 319-324 (1995).

1020. Mandalari G*, et al.* Release of protein, lipid, and vitamin E from almond seeds during digestion. *J Agric Food Chem* **56**, 3409-3416 (2008).

1021. Mandery K*, et al.* Influence of cyclooxygenase inhibitors on the function of the prostaglandin transporter organic anion-transporting polypeptide 2A1 expressed in human gastroduodenal mucosa. *J Pharmacol Exp Ther* **332**, 345-351 (2010).

1022. Mang CF, Truempler S, Erbelding D, Kilbinger H. Modulation by NO of acetylcholine release in the ileum of wild-type and NOS gene knockout mice. *Am J Physiol Gastrointest Liver Physiol* **283**, G1132-1138 (2002).

1023. Mangino MJ, Mangino JE, Kotadia B, Sielczak M. Effects of the 5-lipoxygenase inhibitor A-64077 on intestinal hypothermic organ preservation injury. *J Pharmacol Exp Ther* **281**, 950-956 (1997).

1024. Manjari V, Das UN. Effect of polyunsaturated fatty acids on dexamethasone-induced gastric mucosal damage. *Prostaglandins Leukot Essent Fatty Acids* **62**, 85-96 (2000).

1025. Manoharan P, Coon S, Baseler W, Sundaram S, Kekuda R, Sundaram U. Prostaglandins, not the leukotrienes, regulate Cl(-)/HCO(3)(-) exchange (DRA, SLC26A3) in villus cells in the chronically inflamed rabbit ileum. *Biochim Biophys Acta* **1828**, 179-186 (2013).

1026. Manokas T, Fromkes JJ, Sundaram U. Effect of chronic inflammation on ileal short-chain fatty acid/bicarbonate exchange. *Am J Physiol Gastrointest Liver Physiol* **278**, G585-590 (2000).

1027. Manousos ON*, et al.* Alpha-chain disease with clinical, immunological, and histological recovery. *Br Med J* **2**, 409-412 (1974).

1028. Mantyh CR*, et al.* Substance P activation of enteric neurons in response to intraluminal Clostridium difficile toxin A in the rat ileum. *Gastroenterology* **111**, 1272-1280 (1996).

1029. Maran RR*, et al.* Farnesoid X receptor deficiency in mice leads to increased intestinal epithelial cell proliferation and tumor development. *J Pharmacol Exp Ther* **328**, 469-477 (2009).

1030. Marciani P, Lindi C, Marzo A, Arrigoni Martelli E, Cardace G, Esposito G. L-carnitine and carnitine ester transport in the rat small intestine. *Pharmacol Res* **23**, 157-162 (1991).

1031. Marciano R*, et al.* Effects of prebiotic supplementation on the expression of proteins regulating iron absorption in anaemic growing rats. *Br J Nutr* **113**, 901-908 (2015).

1032. Margaritis VG*, et al.* Effect of oral glutamine administration on bacterial tanslocation, endotoxemia, liver and ileal morphology, and apoptosis in rats with obstructive jaundice. *World J Surg* **29**, 1329-1334 (2005).

1033. Marík T, Bíbr B, Kselíková M, Dobrovský K, Kolínská J, Lener J. Structural analogy among mammalian spectrins and spectrin-like proteins revealed by molybdenum labeling. *Comp Biochem Physiol B* **86**, 531-535 (1987).

1034. Marion R, Coëffier MM, Gargala G, Ducrotté P, Déchelotte PP. Glutamine and CXC chemokines IL-8, Mig, IP-10 and I-TAC in human intestinal epithelial cells. *Clin Nutr* **23**, 579-585 (2004).

1035. Mármol F, Sánchez J, López D, Martínez N, Mitjavila MT, Puig-Parellada P. Oxidative stress, nitric oxide and prostaglandin E2 levels in the gastrointestinal tract of aging rats. *J Pharm Pharmacol* **61**, 201-206 (2009).

1036. Marotta F, Chui DH, Fesce E, Rezakovic I, Zhong GG, Ideo G. Duodenal bicarbonate secretion induced by human epidermal growth factor in rats is partially mediated by prostaglandins. *Digestion* **54**, 19-23 (1993).

1037. Marshall R. Protective effect of disodium cromoglycate on rat peritoneal mast cells. *Thorax* **27**, 38-43 (1972).

1038. Mårtensson J, Jain A, Meister A. Glutathione is required for intestinal function. *Proc Natl Acad Sci U S A* **87**, 1715-1719 (1990).

1039. Martin A*, et al.* [Leukotriene B4 in gastritis and peptic ulcer]. *Minerva Gastroenterol Dietol* **37**, 163-167 (1991).

1040. Martín MT, Goñalons E, Fernández E. Contribution of inhibitory neurotransmitters to the CCK induced relaxation of the circular muscle of avian ileum. *Life Sci* **62**, 937-946 (1998).

1041. Martinez JA*, et al.* Celecoxib use and circulating oxylipins in a colon polyp prevention trial. *PLoS One* **13**, e0196398 (2018).

1042. Martinez V, Jimenez M, Goñalons E, Vergara P. Mechanism of action of CCK in avian gastroduodenal motility: evidence for nitric oxide involvement. *Am J Physiol* **265**, G842-850 (1993).

1043. Martinez-Cuesta MA, Esplugues JV, Whittle BJ. Modulation by nitric oxide of spontaneous motility of the rat isolated duodenum: role of tachykinins. *Br J Pharmacol* **118**, 1335-1340 (1996).

1044. Martinez-Cuesta MA, Massuda H, Whittle BJ, Moncada S. Impairment of nitrergic-mediated relaxation of rat isolated duodenum by experimental diabetes. *Br J Pharmacol* **114**, 919-924 (1995).

1045. Martínez-Herrero S, Martínez A. Adrenomedullin regulates intestinal physiology and pathophysiology. *Domest Anim Endocrinol* **56 Suppl**, S66-83 (2016).

1046. Martins HA*, et al.* l-Glutamine supplementation promotes an improved energetic balance in Walker-256 tumor-bearing rats. *Tumour Biol* **39**, 1010428317695960 (2017).

1047. Martins SR, Bicudo R, Oliveira RB, Ballejo G. Evidence for the participation of the L-arginine-nitric oxide pathway in neurally induced relaxation of the isolated rat duodenum. *Braz J Med Biol Res* **26**, 1325-1335 (1993).

1048. Mascolo N, Izzo AA, Gaginella TS, Capasso F. Relationship between nitric oxide and platelet-activating factor in castor-oil induced mucosal injury in the rat duodenum. *Naunyn Schmiedebergs Arch Pharmacol* **353**, 680-684 (1996).

1049. Massaro ER, Simpson RU, DeLuca HF. Glucocorticoids and appearance of 1,25-dihydroxyvitamin D3 receptor in rat intestine. *Am J Physiol* **244**, E230-235 (1983).

1050. Massart J*, et al.* Pentoxifylline aggravates fatty liver in obese and diabetic ob/ob mice by increasing intestinal glucose absorption and activating hepatic lipogenesis. *Br J Pharmacol* **165**, 1361-1374 (2012).

1051. Mastboom WJ, Hendriks T, de Man BM, de Boer HH. Influence of methylprednisolone on the healing of intestinal anastomoses in rats. *Br J Surg* **78**, 54-56 (1991).

1052. Mastboom WJ, Hendriks T, van Elteren P, de Boer HH. Piroxicam affects collagen changes around experimental intestinal anastomoses. *Eur Surg Res* **21**, 305-312 (1989).

1053. Mastrototaro L, Sponder G, Saremi B, Aschenbach JR. Gastrointestinal methionine shuttle: Priority handling of precious goods. *IUBMB Life* **68**, 924-934 (2016).

1054. Masuda D, Yamashita S. Postprandial Hyperlipidemia and Remnant Lipoproteins. *J Atheroscler Thromb* **24**, 95-109 (2017).

1055. Matheson PJ, Li N, Harris PD, Zakaria el R, Garrison RN. Glucose-induced intestinal vasodilation via adenosine A1 receptors requires nitric oxide but not K(+)(ATP) channels. *J Surg Res* **168**, 179-187 (2011).

1056. Matheson PJ, Wilson MA, Spain DA, Harris PD, Anderson GL, Garrison RN. Glucose-induced intestinal hyperemia is mediated by nitric oxide. *J Surg Res* **72**, 146-154 (1997).

1057. Mathias JR, Carlson GM, Bertiger G, Martin JL, Cohen S. Migrating action potential complex of cholera: a possible prostaglandin-induced response. *Am J Physiol* **232**, E529-534 (1977).

1058. Mathias PM, Harries JT, Peters TJ, Muller DP. Studies on the in vivo absorption of micellar solutions of tocopherol and tocopheryl acetate in the rat: demonstration and partial characterization of a mucosal esterase localized to the endoplasmic reticulum of the enterocyte. *J Lipid Res* **22**, 829-837 (1981).

1059. Mathison R, Davison JS. Capsaicin sensitive nerves in the jejunum of Nippostrongylus brasiliensis-sensitized rats participate in a cardiovascular depressor reflex. *Naunyn Schmiedebergs Arch Pharmacol* **348**, 638-642 (1993).

1060. Mathison R, Davison JS. Regulation of jejunal arterioles by capsaicin-sensitive nerves in Nippostrongylus brasiliensis-sensitized rats. *J Pharmacol Exp Ther* **273**, 337-343 (1995).

1061. Matoušková P, Bártíková H, Boušová I, Levorová L, Szotáková B, Skálová L. Drug-metabolizing and antioxidant enzymes in monosodium L-glutamate obese mice. *Drug Metab Dispos* **43**, 258-265 (2015).

1062. Matsuda NM, Feitosa RL, Jr., Lemos MC, De Oliveira RB, Ballejo G. In vitro and in vivo effects of nitric oxide synthase inhibitors and nitric oxide inactivators on the South American opossum ileocolonic junction. *Neurogastroenterol Motil* **10**, 245-252 (1998).

1063. Matsumoto T, Iida M, Kuroki F, Hizawa K, Koga H, Fujishima M. Effects of diet on experimentally induced intestinal ulcers in rats: morphology and tissue leukotrienes. *Gut* **35**, 1058-1063 (1994).

1064. Matsunaga H*, et al.* Omega-3 polyunsaturated fatty acids ameliorate the severity of ileitis in the senescence accelerated mice (SAM)P1/Yit mice model. *Clin Exp Immunol* **158**, 325-333 (2009).

1065. Matsuno Y*, et al.* Measurement of prostaglandin metabolites is useful in diagnosis of small bowel ulcerations. *World J Gastroenterol* **25**, 1753-1763 (2019).

1066. Matsushita N*, et al.* Pharmacological studies on the novel antiallergic drug HQL-79: II. Elucidation of mechanisms for antiallergic and antiasthmatic effects. *Jpn J Pharmacol* **78**, 11-22 (1998).

1067. Matsuyama H*, et al.* Peptidergic and nitrergic inhibitory neurotransmissions in the hamster jejunum: regulation of vasoactive intestinal peptide release by nitric oxide. *Neuroscience* **110**, 779-788 (2002).

1068. Matsuyama H, Unno T, Komori S, Takewaki T. Nitrergic inhibition of tachykininergic neuro-muscular transmission via cyclic GMP in the hamster ileum. *J Vet Med Sci* **73**, 453-458 (2011).

1069. Matuchansky C, Mary JY, Bernier JJ. Further studies on prostaglandin E1-induced jejunal secretion of water and electrolytes in man, with special reference to the influence of ethacrynic acid, furosemide, and aspirin. *Gastroenterology* **71**, 274-281 (1976).

1070. Matuszek MA, Hodgson WC, Sutherland SK, King RG. Pharmacological studies of jumper ant (Myrmecia pilosula) venom: evidence for the presence of histamine, and haemolytic and eicosanoid-releasing factors. *Toxicon* **30**, 1081-1091 (1992).

1071. Maubach KA, Grundy D. The role of prostaglandins in the bradykinin-induced activation of serosal afferents of the rat jejunum in vitro. *J Physiol* **515 ( Pt 1)**, 277-285 (1999).

1072. Maurício AC, Ferreira KT. Activation of ionic channels by deoxycholate in frog and human cell lines. *Exp Physiol* **84**, 489-499 (1999).

1073. Mayer S, Weiss J, McClements DJ. Behavior of vitamin E acetate delivery systems under simulated gastrointestinal conditions: lipid digestion and bioaccessibility of low-energy nanoemulsions. *J Colloid Interface Sci* **404**, 215-222 (2013).

1074. Mazmanian SK. Gut immune balance is as easy as S-F-B. *Immunity* **31**, 536-538 (2009).

1075. Mazzeo MF, Lippolis R, Sorrentino A, Liberti S, Fragnito F, Siciliano RA. Lactobacillus acidophilus-Rutin Interplay Investigated by Proteomics. *PLoS One* **10**, e0142376 (2015).

1076. McArdle AH. Protection from radiation injury by elemental diet: does added glutamine change the effect? *Gut* **35**, S60-64 (1994).

1077. McCarthy DM, Kreel L, Agnew JE, Bouchier IA. Value of hypotonic duodenography as an adjunct to pancreatic scanning. *Gut* **10**, 665-673 (1969).

1078. McCracken BA, Zijlstra RT, Donovan SM, Odle J, Lien EL, Gaskins HR. Neither intact nor hydrolyzed soy proteins elicit intestinal inflammation in neonatal piglets. *JPEN J Parenter Enteral Nutr* **22**, 91-97 (1998).

1079. McCready DR, Wallace JL, Cohen MM. Prostaglandin biosynthesis by gastric mucosa. II. Studies in man. *Clin Biochem* **17**, 183-187 (1984).

1080. McKay DM, Lu J, Jedrzkiewicz S, Ho W, Sharkey KA. Nitric oxide participates in the recovery of normal jejunal epithelial ion transport following exposure to the superantigen, Staphylococcus aureus enterotoxin B. *J Immunol* **163**, 4519-4526 (1999).

1081. McVey DC, Vigna SR. The capsaicin VR1 receptor mediates substance P release in toxin A-induced enteritis in rats. *Peptides* **22**, 1439-1446 (2001).

1082. Meddings JB, Wallace JL, Sutherland LR. Sucrose Permeability: A Novel Means of Detecting Gastroduodenal Damage Noninvasively. *Am J Ther* **2**, 843-849 (1995).

1083. Melli M. Assessment of plasma leukotriene and prostaglandin levels during adjuvant arthritis and kaolin-induced paw oedema in rats. *Prostaglandins Leukot Essent Fatty Acids* **33**, 173-178 (1988).

1084. Mellors AJ, Nahrwold DL, Rose RC. Ascorbic acid flux across mucosal border of guinea pig and human ileum. *Am J Physiol* **233**, E374-379 (1977).

1085. Meng FW, Slivka PF, Dearth CL, Badylak SF. Solubilized extracellular matrix from brain and urinary bladder elicits distinct functional and phenotypic responses in macrophages. *Biomaterials* **46**, 131-140 (2015).

1086. Mensinga TT, Speijers GJ, Meulenbelt J. Health implications of exposure to environmental nitrogenous compounds. *Toxicol Rev* **22**, 41-51 (2003).

1087. Mercier S, Breuillé D, Mosoni L, Obled C, Patureau Mirand P. Chronic inflammation alters protein metabolism in several organs of adult rats. *J Nutr* **132**, 1921-1928 (2002).

1088. Merendino N, Dwinell MB, Varki N, Eckmann L, Kagnoff MF. Human intestinal epithelial cells express receptors for platelet-activating factor. *Am J Physiol* **277**, G810-818 (1999).

1089. Merkle P, Bindewald H, Breitig D. [Functional and metabolic changes of the mucosa during the occlusion of the small bowel of the rat (author's transl)]. *Langenbecks Arch Chir* **338**, 291-297 (1975).

1090. Merlin D*, et al.* Colonic epithelial hPepT1 expression occurs in inflammatory bowel disease: transport of bacterial peptides influences expression of MHC class 1 molecules. *Gastroenterology* **120**, 1666-1679 (2001).

1091. Meschter CL, Gilbert M, Krook L, Maylin G, Corradino R. The effects of phenylbutazone on the intestinal mucosa of the horse: a morphological, ultrastructural and biochemical study. *Equine Vet J* **22**, 255-263 (1990).

1092. Metwaly MS, Dkhil MA, Al-Quraishy S. The potential role of Phoenix dactylifera on Eimeria papillata-induced infection in mice. *Parasitol Res* **111**, 681-687 (2012).

1093. Metz T*, et al.* A new method for targeted drug delivery using polymeric microcapsules: implications for treatment of Crohn's disease. *Cell Biochem Biophys* **43**, 77-85 (2005).

1094. Meyer TA, Wang J, Tiao GM, Ogle CK, Fischer JE, Hasselgren PO. Sepsis and endotoxemia stimulate intestinal interleukin-6 production. *Surgery* **118**, 336-342 (1995).

1095. Miao FJ, Green PG, Levine JD. Mechanosensitive duodenal afferents contribute to vagal modulation of inflammation in the rat. *J Physiol* **554**, 227-235 (2004).

1096. Michels AJ, Hagen TM. Hepatocyte nuclear factor 1 is essential for transcription of sodium-dependent vitamin C transporter protein 1. *Am J Physiol Cell Physiol* **297**, C1220-1227 (2009).

1097. Miklavcic JJ*, et al.* Increased catabolism and decreased unsaturation of ganglioside in patients with inflammatory bowel disease. *World J Gastroenterol* **21**, 10080-10090 (2015).

1098. Miller MJ*, et al.* Nitric oxide: the Jekyll and Hyde of gut inflammation. *Agents Actions* **39 Spec No**, C180-182 (1993).

1099. Miller MJ*, et al.* Treatment of gastric ulcers and diarrhea with the Amazonian herbal medicine sangre de grado. *Am J Physiol Gastrointest Liver Physiol* **279**, G192-200 (2000).

1100. Miller MJ, Sadowska-Krowicka H, Chotinaruemol S, Kakkis JL, Clark DA. Amelioration of chronic ileitis by nitric oxide synthase inhibition. *J Pharmacol Exp Ther* **264**, 11-16 (1993).

1101. Miller MJ*, et al.* Substance P levels in experimental ileitis in guinea pigs: effects of misoprostol. *Am J Physiol* **265**, G321-330 (1993).

1102. Miller MJ*, et al.* Rabbit gut permeability in response to histamine chloramines and chemotactic peptide. *Gastroenterology* **103**, 1537-1546 (1992).

1103. Miller SJ, Coppinger BJ, Zhou X, Unthank JL. Antioxidants reverse age-related collateral growth impairment. *J Vasc Res* **47**, 108-114 (2010).

1104. Mills EL*, et al.* Itaconate is an anti-inflammatory metabolite that activates Nrf2 via alkylation of KEAP1. *Nature* **556**, 113-117 (2018).

1105. Milusheva EA, Kuneva VI, Itzev DE, Kortezova NI, Sperlagh B, Mizhorkova ZN. Glutamate stimulation of acetylcholine release from myenteric plexus is mediated by endogenous nitric oxide. *Brain Res Bull* **66**, 229-234 (2005).

1106. Mitchell GW, Wood JD. Effects of inhibitors of prostaglandin synthesis on rebound excitation of guinea-pig small bowel. *Eur J Pharmacol* **40**, 63-66 (1976).

1107. Mitchison HC, al Mardini H, Gillespie S, Laker M, Zaitoun A, Record CO. A pilot study of fluticasone propionate in untreated coeliac disease. *Gut* **32**, 260-265 (1991).

1108. Mitsuoka H, Kistler EB, Schmid-Schönbein GW. Protease inhibition in the intestinal lumen: attenuation of systemic inflammation and early indicators of multiple organ failure in shock. *Shock* **17**, 205-209 (2002).

1109. Miura S*, et al.* Microcirculatory disturbance in indomethacin-induced intestinal ulcer. *Am J Physiol* **261**, G213-219 (1991).

1110. Miura Y, Yoshikawa I, Ohe K. [Characteristics of the effect of 16,16-dimethyl prostaglandin E2 on the alkaline secretion of the proximal duodenal mucosa in rats--a comparison with the effect of secretin]. *J uoeh* **11**, 313-322 (1989).

1111. Miyakuni T*, et al.* Cystine and Theanine Improve Survival after Gut Ischemia-Reperfusion. *Ann Nutr Metab* **73**, 131-137 (2018).

1112. Miyamoto K, Katsuragi T, Abdu P, Furukawa T. Effects of baicalein on prostanoid generation from the lung and contractile responses of the trachea in guinea pig. *Am J Chin Med* **25**, 37-50 (1997).

1113. Mizhorkova Z, Batova M, Milusheva EA. Participation of endogenous nitric oxide in the effect of hypoxia in vitro on neuro-effector transmission in guinea-pig ileum. *Brain Res Bull* **55**, 453-458 (2001).

1114. Mizhorkova Z, Kortezova N, Bredy-Dobreva G, Papasova M. Role of nitric oxide in mediating non-adrenergic non-cholinergic relaxation of the cat ileocecal sphincter. *Eur J Pharmacol* **265**, 77-82 (1994).

1115. Mizhorkova Z, Milusheva E, Papasova M. Involvement of nitric oxide in extrinsic nervous control of ileal contractile activity. *Brain Res Bull* **47**, 151-154 (1998).

1116. Mizoguchi H, Ogawa Y, Kanatsu K, Tanaka A, Kato S, Takeuchi K. Protective effect of rebamipide on indomethacin-induced intestinal damage in rats. *J Gastroenterol Hepatol* **16**, 1112-1119 (2001).

1117. Mizutani N*, et al.* Dose-dependent differential regulation of cytokine secretion from macrophages by fractalkine. *J Immunol* **179**, 7478-7487 (2007).

1118. Moazedi AA, Dabir N, Naseri MK, Zadkarami MR. The role of NO and cGMP in antispasmodic activity of Ruta chalepensis leaf extract on rat ileum. *Pak J Biol Sci* **13**, 83-87 (2010).

1119. Mobley CB*, et al.* Herbal adaptogens combined with protein fractions from bovine colostrum and hen egg yolk reduce liver TNF-α expression and protein carbonylation in Western diet feeding in rats. *Nutr Metab (Lond)* **11**, 19 (2014).

1120. Mobraten K, Haug TM, Kleiveland CR, Lea T. Omega-3 and omega-6 PUFAs induce the same GPR120-mediated signalling events, but with different kinetics and intensity in Caco-2 cells. *Lipids Health Dis* **12**, 101 (2013).

1121. Moens F*, et al.* A four-strain probiotic exerts positive immunomodulatory effects by enhancing colonic butyrate production in vitro. *Int J Pharm* **555**, 1-10 (2019).

1122. Moeser AJ, Nighot PK, Engelke KJ, Ueno R, Blikslager AT. Recovery of mucosal barrier function in ischemic porcine ileum and colon is stimulated by a novel agonist of the ClC-2 chloride channel, lubiprostone. *Am J Physiol Gastrointest Liver Physiol* **292**, G647-656 (2007).

1123. Moilanen E, Thomsen LL, Miles DW, Happerfield DW, Knowles RG, Moncada S. Persistent induction of nitric oxide synthase in tumours from mice treated with the anti-tumour agent 5,6-dimethylxanthenone-4-acetic acid. *Br J Cancer* **77**, 426-433 (1998).

1124. Moine L, Díaz de Barboza G, Pérez A, Benedetto M, Tolosa de Talamoni N. Glutamine protects intestinal calcium absorption against oxidative stress and apoptosis. *Comp Biochem Physiol A Mol Integr Physiol* **212**, 64-71 (2017).

1125. Mojibian M*, et al.* Insulin-producing intestinal K cells protect nonobese diabetic mice from autoimmune diabetes. *Gastroenterology* **147**, 162-171.e166 (2014).

1126. Möller A, Grabbe J, Rosenbach T, Wilhelms OH, Czarnetzki BM. In vitro generation of smooth muscle-contracting leukotrienes from isolated epidermal cells. *Skin Pharmacol* **4**, 165-168 (1991).

1127. Monagas M*, et al.* Insights into the metabolism and microbial biotransformation of dietary flavan-3-ols and the bioactivity of their metabolites. *Food Funct* **1**, 233-253 (2010).

1128. Mondello S*, et al.* Glutamine treatment attenuates the development of ischaemia/reperfusion injury of the gut. *Eur J Pharmacol* **643**, 304-315 (2010).

1129. Monk JM*, et al.* Fish oil supplementation to a high-fat diet improves both intestinal health and the systemic obese phenotype. *J Nutr Biochem* **72**, 108216 (2019).

1130. Monteiro-Sepulveda M*, et al.* Jejunal T Cell Inflammation in Human Obesity Correlates with Decreased Enterocyte Insulin Signaling. *Cell Metab* **22**, 113-124 (2015).

1131. Monteleone G, Pender SL, Wathen NC, MacDonald TT. Interferon-alpha drives T cell-mediated immunopathology in the intestine. *Eur J Immunol* **31**, 2247-2255 (2001).

1132. Montgomery A, Källén R, Fält K, Haglund U, Borgström A. Reperfusion of duodenum and pancreas following 24 hr of cold storage in Perfadex or UW solution. *J Surg Res* **55**, 615-621 (1993).

1133. Moog F, Goellner JJ. Chick embryo intestine in culture: influence of insulin and other hormones on sucrase, maltase, and alkaline phosphatase. *J Pediatr Gastroenterol Nutr* **1**, 401-410 (1982).

1134. Mooney MH, Abdel-Wahab YH, McKillop AM, O'Harte FP, Flatt PR. Evaluation of glycated glucagon-like peptide-1(7-36)amide in intestinal tissue of normal and diabetic animal models. *Biochim Biophys Acta* **1569**, 75-80 (2002).

1135. Moore JG, Bjorkman DJ, Mitchell MD, Avots-Avotins A. Age does not influence acute aspirin-induced gastric mucosal damage. *Gastroenterology* **100**, 1626-1629 (1991).

1136. Moran AE, Hunt DH, Javid SH, Redston M, Carothers AM, Bertagnolli MM. Apc deficiency is associated with increased Egfr activity in the intestinal enterocytes and adenomas of C57BL/6J-Min/+ mice. *J Biol Chem* **279**, 43261-43272 (2004).

1137. Morand C, Manach C, Crespy V, Remesy C. Respective bioavailability of quercetin aglycone and its glycosides in a rat model. *Biofactors* **12**, 169-174 (2000).

1138. Morand C, Manach C, Crespy V, Remesy C. Quercetin 3-O-beta-glucoside is better absorbed than other quercetin forms and is not present in rat plasma. *Free Radic Res* **33**, 667-676 (2000).

1139. Moreels TG*, et al.* Effect of Schistosoma mansoni-induced granulomatous inflammation on murine gastrointestinal motility. *Am J Physiol Gastrointest Liver Physiol* **280**, G1030-1042 (2001).

1140. Moreels TG, De Man JG, De Winter BY, Timmermans JP, Herman AG, Pelckmans PA. Effect of 2,4,6-trinitrobenzenesulphonic acid (TNBS)-induced ileitis on the motor function of non-inflamed rat gastric fundus. *Neurogastroenterol Motil* **13**, 339-352 (2001).

1141. Moretton MA*, et al.* Novel nelfinavir mesylate loaded d-α-tocopheryl polyethylene glycol 1000 succinate micelles for enhanced pediatric anti HIV therapy: In vitro characterization and in vivo evaluation. *Colloids Surf B Biointerfaces* **123**, 302-310 (2014).

1142. Morin CL, Thompson MW, Jackson SH, Sass-Kortsak A. Biochemical and genetic studies in cystinuria: observations on double heterozygotes of genotype I-II. *J Clin Invest* **50**, 1961-1976 (1971).

1143. Moritoki H, Ishida Y. Aspects of the spasmogenic effects of acetate esters on ileal smooth muscle. *Eur J Pharmacol* **42**, 347-354 (1977).

1144. Motte J*, et al.* Capsaicin-enriched diet ameliorates autoimmune neuritis in rats. *J Neuroinflammation* **15**, 122 (2018).

1145. Mourad FH*, et al.* Interplay between nitric oxide and vasoactive intestinal polypeptide in inducing fluid secretion in rat jejunum. *J Physiol* **550**, 863-871 (2003).

1146. Mourad FH, Barada KA, Bou Rached NA, Khoury CI, Saadé NE, Nassar CF. Inhibitory effect of experimental colitis on fluid absorption in rat jejunum: role of the enteric nervous system, VIP, and nitric oxide. *Am J Physiol Gastrointest Liver Physiol* **290**, G262-268 (2006).

1147. Mourad FH, Hamdi T, Barada KA, Saadé NE. Experimental colitis in rats induces de novo synthesis of cytokines at distant intestinal sites: role of capsaicin-sensitive primary afferent fibers. *Eur Cytokine Netw* **27**, 41-53 (2016).

1148. Mourad FH*, et al.* L-Arginine, nitric oxide, and intestinal secretion: studies in rat jejunum in vivo. *Gut* **39**, 539-544 (1996).

1149. Mourão JL*, et al.* Effect of dietary dehydrated pasture and citrus pulp on the performance and meat quality of broiler chickens. *Poult Sci* **87**, 733-743 (2008).

1150. Mourelle M, Guarner F, Moncada S, Malagelada JR. The arginine/nitric oxide pathway modulates sphincter of Oddi motor activity in guinea pigs and rabbits. *Gastroenterology* **105**, 1299-1305 (1993).

1151. Mowat C, McColl KE. Alterations in intragastric nitrite and vitamin C levels during acid inhibitory therapy. *Best Pract Res Clin Gastroenterol* **15**, 523-537 (2001).

1152. Mtabaji JP, Manku MS, Horrobin DF. Actions of the tricyclic antidepressant clomipramine on responses to pressor agents. Interactions with prostaglandin E2. *Prostaglandins* **14**, 125-132 (1977).

1153. Mueller MH, Kampitoglou D, Glatzle J, Hahn J, Kreis ME. Systemic capsaicin inhibits neuronal activation in the brainstem during postoperative ileus in the mouse. *Langenbecks Arch Surg* **391**, 88-95 (2006).

1154. Müller P, Dammann HG, Bergdolt H, Simon B. [The effect of glycine on the gastroduodenal tolerability of acetylsalicylic acid. An endoscopic, controlled double-blind study in healthy subjects]. *Arzneimittelforschung* **41**, 812-814 (1991).

1155. Müller P, Simon B. Effects of ibuprofen lysinate and acetylsalicylic acid on gastric and duodenal mucosa. Randomized single-blind placebo-controlled endoscopic study in healthy volunteers. *Arzneimittelforschung* **44**, 840-843 (1994).

1156. Munck LK, Mertz-Nielsen A, Westh H, Bukhave K, Beubler E, Rask-Madsen J. Prostaglandin E2 is a mediator of 5-hydroxytryptamine induced water and electrolyte secretion in the human jejunum. *Gut* **29**, 1337-1341 (1988).

1157. Munday JS, Bentall H, Aberdein D, Navarro M, Uzal FA, Brown S. Death of a neonatal lamb due to Clostridium perfringens type B in New Zealand. *N Z Vet J* **68**, 242-246 (2020).

1158. Muniz CK*, et al.* Nutritional status, fecal elastase-1, and 13C-labeled mixed triglyceride breath test in the long-term after pancreaticoduodenectomy. *Pancreas* **43**, 445-450 (2014).

1159. Muñoz-Arrebola P, Madrid JA, Salido GM, Martinez de Victoria E. Modifications of gamma-glutamyl transpeptidase activity in duodenal mucosa of rats treated with different antiulcer drugs. *Arch Int Physiol Biochim* **97**, 231-234 (1989).

1160. Murakami AE, Sakamoto MI, Natali MR, Souza LM, Franco JR. Supplementation of glutamine and vitamin E on the morphometry of the intestinal mucosa in broiler chickens. *Poult Sci* **86**, 488-495 (2007).

1161. Murakami M*, et al.* Enteral siRNA delivery technique for therapeutic gene silencing in the liver via the lymphatic route. *Sci Rep* **5**, 17035 (2015).

1162. Murthy S, Mathur S, Bishop WP, Field EJ. Inhibition of apolipoprotein B secretion by IL-6 is mediated by EGF or an EGF-like molecule in CaCo-2 cells. *J Lipid Res* **38**, 206-216 (1997).

1163. Musch MW, Miller RJ, Field M, Siegel MI. Stimulation of colonic secretion by lipoxygenase metabolites of arachidonic acid. *Science* **217**, 1255-1256 (1982).

1164. Mutanen A, Lohi J, Heikkilä P, Jalanko H, Pakarinen MP. Loss of ileum decreases serum fibroblast growth factor 19 in relation to liver inflammation and fibrosis in pediatric onset intestinal failure. *J Hepatol* **62**, 1391-1397 (2015).

1165. Mutlu E, Keshavarzian A, Engen P, Forsyth CB, Sikaroodi M, Gillevet P. Intestinal dysbiosis: a possible mechanism of alcohol-induced endotoxemia and alcoholic steatohepatitis in rats. *Alcohol Clin Exp Res* **33**, 1836-1846 (2009).

1166. Mutlu-Türkoğlu U, Erbil Y, Oztezcan S, Olgaç V, Toker G, Uysal M. The effect of selenium and/or vitamin E treatments on radiation-induced intestinal injury in rats. *Life Sci* **66**, 1905-1913 (2000).

1167. Myers CP, Hogan D, Yao B, Koss M, Isenberg JI, Barrett KE. Inhibition of rabbit duodenal bicarbonate secretion by ulcerogenic agents: histamine-dependent and -independent effects. *Gastroenterology* **114**, 527-535 (1998).

1168. Mykkanen HM, Wasserman RH. Uptake of 75Se-selenite by brush border membrane vesicles from chick duodenum stimulated by vitamin D. *J Nutr* **119**, 242-247 (1989).

1169. Mykkanen HM, Wasserman RH. Relationship of membrane-bound sulfhydryl groups to vitamin D-stimulated uptake of [75Se]Selenite by the brush border membrane vesicles from chick duodenum. *J Nutr* **120**, 882-888 (1990).

1170. N'Dow J, Robson CN, Matthews JN, Neal DE, Pearson JP. Reducing mucus production after urinary reconstruction: a prospective randomized trial. *J Urol* **165**, 1433-1440 (2001).

1171. Nagahama M, Semba R, Tsuzuki M, Aoki E. L-arginine immunoreactive enteric glial cells in the enteric nervous system of rat ileum. *Biol Signals Recept* **10**, 336-340 (2001).

1172. Nagórna-Stasiak B, Lazuga-Adamczyk A, Kołodyńska M. Alpha-amylase in the serum and tissues of rabbits and the effect of vitamin C on its activity. *Acta Physiol Pol* **38**, 15-21 (1987).

1173. Nagórna-Stasiak B, Wawrzeńska M. [Effect of vitamins A, D3 and E on intestinal motility in rabbits in chronic experiments and in vitro]. *Pol Arch Weter* **24**, 215-227 (1984).

1174. Nagórna-Stasiak B, Wawrzeńska M, Kopeć R, Lietz W. [Effect of cholesterol on the level of ascorbic acid in the plasma and tissues of rabbits]. *Pol Arch Weter* **25**, 247-251 (1987).

1175. Naini A, Kaufmann P, Shanske S, Engelstad K, De Vivo DC, Schon EA. Hypocitrullinemia in patients with MELAS: an insight into the "MELAS paradox". *J Neurol Sci* **229-230**, 187-193 (2005).

1176. Nakabou Y, Kubota M, Takada K, Ojima M. A possible approach to the suppression of side effects induced by PGE1. *Prostaglandins Leukot Essent Fatty Acids* **52**, 17-20 (1995).

1177. Nakahata N, Ono T, Nakanishi H. Possible involvement of a product of the 5-lipoxygenase pathway in mediation of indomethacin-induced inhibition of cholinergic transmission in guinea-pig ileum. *Eur J Pharmacol* **104**, 133-138 (1984).

1178. Nakai Y*, et al.* Functional characterization of human proton-coupled folate transporter/heme carrier protein 1 heterologously expressed in mammalian cells as a folate transporter. *J Pharmacol Exp Ther* **322**, 469-476 (2007).

1179. Nakajima Y, Baudry N, Duranteau J, Vicaut E. Effects of vasopressin, norepinephrine, and L-arginine on intestinal microcirculation in endotoxemia. *Crit Care Med* **34**, 1752-1757 (2006).

1180. Nakanishi H*, et al.* Involvement of nitric oxide released from microglia-macrophages in pathological changes of cathepsin D-deficient mice. *J Neurosci* **21**, 7526-7533 (2001).

1181. Nakata K, Osumi Y, Fujiwara M. Prostaglandins and the contractility of the guinea pig biliary system. *Pharmacology* **22**, 24-30 (1981).

1182. Napoli JL, Race KR. Biogenesis of retinoic acid from beta-carotene. Differences between the metabolism of beta-carotene and retinal. *J Biol Chem* **263**, 17372-17377 (1988).

1183. Narushima K, Takada T, Yamanashi Y, Suzuki H. Niemann-pick C1-like 1 mediates alpha-tocopherol transport. *Mol Pharmacol* **74**, 42-49 (2008).

1184. Nase GP, Boegehold MA. Endothelium-derived nitric oxide limits sympathetic neurogenic constriction in intestinal microcirculation. *Am J Physiol* **273**, H426-433 (1997).

1185. Nase GP, Tuttle J, Bohlen HG. Reduced perivascular PO2 increases nitric oxide release from endothelial cells. *Am J Physiol Heart Circ Physiol* **285**, H507-515 (2003).

1186. Nasser Y, Keenan CM, Ma AC, McCafferty DM, Sharkey KA. Expression of a functional metabotropic glutamate receptor 5 on enteric glia is altered in states of inflammation. *Glia* **55**, 859-872 (2007).

1187. Nassuato G*, et al.* Biliary excretion of simultaneously administered bilirubin and chenodeoxycholic acid in rats. *Boll Soc Ital Biol Sper* **57**, 891-895 (1981).

1188. Nassuato G*, et al.* Biliary excretion of simulateously administered bilirubin and chenodeoxycholic acid in rats with porta-caval shunt. *Boll Soc Ital Biol Sper* **57**, 896-899 (1981).

1189. Naumov I*, et al.* CD24 knockout prevents colorectal cancer in chemically induced colon carcinogenesis and in APC(Min)/CD24 double knockout transgenic mice. *Int J Cancer* **135**, 1048-1059 (2014).

1190. Navab M*, et al.* D-4F-mediated reduction in metabolites of arachidonic and linoleic acids in the small intestine is associated with decreased inflammation in low-density lipoprotein receptor-null mice. *J Lipid Res* **53**, 437-445 (2012).

1191. Navab M*, et al.* High-density lipoprotein and 4F peptide reduce systemic inflammation by modulating intestinal oxidized lipid metabolism: novel hypotheses and review of literature. *Arterioscler Thromb Vasc Biol* **32**, 2553-2560 (2012).

1192. Naveh Y, Ken-Dror A, Zinder O, Berkovich D. Comparative reliability of D-xylose absorption and serum beta-carotene measurements in small intestinal disease. *J Pediatr Gastroenterol Nutr* **5**, 210-213 (1986).

1193. Naveh Y, Lee-Ambrose LM, Samuelson DA, Cousins RJ. Malabsorption of zinc in rats with acetic acid-induced enteritis and colitis. *J Nutr* **123**, 1389-1395 (1993).

1194. Naveh Y, Shalata A, Shenker L, Coleman R. Absorption of iron in rats with experimental enteritis. *Biometals* **13**, 29-35 (2000).

1195. Nawrot-Porąbka K, Jaworek J, Leja-Szpak A, Szklarczyk J, Konturek SJ, Reiter RJ. Luminal melatonin stimulates pancreatic enzyme secretion via activation of serotonin-dependent nerves. *Pharmacol Rep* **65**, 494-504 (2013).

1196. Nawrot-Porabka K*, et al.* Involvement of vagal nerves in the pancreatostimulatory effects of luminal melatonin, or its precursor L-tryptophan. Study in the rats. *J Physiol Pharmacol* **58 Suppl 6**, 81-95 (2007).

1197. Neale JR, James S, Callaghan J, Patel P. Premedication with N-acetylcysteine and simethicone improves mucosal visualization during gastroscopy: a randomized, controlled, endoscopist-blinded study. *Eur J Gastroenterol Hepatol* **25**, 778-783 (2013).

1198. Needleman DS, Leeper LL, Nanthakumar NN, Henning SJ. Hormonal regulation of the mRNA for cysteine-rich intestinal protein in rat jejunum during maturation. *J Pediatr Gastroenterol Nutr* **16**, 15-22 (1993).

1199. Needleman P, Key SL, Denny SE, Isakson PC, Marshall GR. Mechanism and modification of bradykinin-induced coronary vasodilation. *Proc Natl Acad Sci U S A* **72**, 2060-2063 (1975).

1200. Nelson SM, Shay AE, James JL, Carlson BA, Urban JF, Jr., Prabhu KS. Selenoprotein Expression in Macrophages Is Critical for Optimal Clearance of Parasitic Helminth Nippostrongylus brasiliensis. *J Biol Chem* **291**, 2787-2798 (2016).

1201. Nelson YB, Pande D, McCormick J, Balloch KJ, Lucas ML. Lack of evidence in vivo for nitrergic inhibition by Escherichia coli (STa) enterotoxin of fluid absorption from rat proximal jejunum. *J Physiol Biochem* **63**, 231-237 (2007).

1202. Nemere I, Feld C, Norman AW. 1,25-Dihydroxyvitamin D3-mediated alterations in microtubule proteins isolated from chick intestinal epithelium: analyses by isoelectric focusing. *J Cell Biochem* **47**, 369-379 (1991).

1203. Nemoto S, Otsuka M, Arakawa N. A positive correlation between catalase activity and ascorbate uptake in the tissues of guinea pigs and cultured cells of mammals. *J Nutr Sci Vitaminol (Tokyo)* **42**, 387-395 (1996).

1204. Newberry RD, McDonough JS, Stenson WF, Lorenz RG. Spontaneous and continuous cyclooxygenase-2-dependent prostaglandin E2 production by stromal cells in the murine small intestine lamina propria: directing the tone of the intestinal immune response. *J Immunol* **166**, 4465-4472 (2001).

1205. Newmark HL, Huang MT, Reddy BS. Mixed tocopherols inhibit azoxymethane-induced aberrant crypt foci in rats. *Nutr Cancer* **56**, 82-85 (2006).

1206. Nguyen DN, Li Y, Sangild PT, Bering SB, Chatterton DE. Effects of bovine lactoferrin on the immature porcine intestine. *Br J Nutr* **111**, 321-331 (2014).

1207. Nguyen TD, Hållenius FF, Lin X, Nyman M, Prykhodko O. Monobutyrin and Monovalerin Affect Brain Short-Chain Fatty Acid Profiles and Tight-Junction Protein Expression in ApoE-Knockout Rats Fed High-Fat Diets. *Nutrients* **12**, (2020).

1208. Nicholls RJ, Belliveau P, Neill M, Wilks M, Tabaqchali S. Restorative proctocolectomy with ileal reservoir: a pathophysiological assessment. *Gut* **22**, 462-468 (1981).

1209. Nickdel MB, Roberts F, Brombacher F, Alexander J, Roberts CW. Counter-protective role for interleukin-5 during acute Toxoplasma gondii infection. *Infect Immun* **69**, 1044-1052 (2001).

1210. Nigam SK*, et al.* The organic anion transporter (OAT) family: a systems biology perspective. *Physiol Rev* **95**, 83-123 (2015).

1211. Nigro ND. A strategy for prevention of cancer of the large bowel. *Dis Colon Rectum* **25**, 755-758 (1982).

1212. Niida H, Okada M, Takeuchi K, Okabe S. A new model of duodenal ulcers induced in rats by diethyldithiocarbamate, a superoxide dismutase inhibitor. *Scand J Gastroenterol Suppl* **162**, 116-119 (1989).

1213. Nijhout HF*, et al.* A mathematical model gives insights into the effects of vitamin B-6 deficiency on 1-carbon and glutathione metabolism. *J Nutr* **139**, 784-791 (2009).

1214. Nijs G, de Witte P, Geboes K, Meulemans A, Schuurkes J, Lemli J. Influence of rhein anthrone on peristaltic reflex of guinea-pig isolated ileum: involvement of prostaglandins. *Br J Pharmacol* **108**, 269-273 (1993).

1215. Nijs G, de Witte P, Geboes K, Meulemans A, Schuurkes J, Lemli J. In vitro demonstration of a positive effect of rhein anthrone on peristaltic reflex of guinea pig ileum. *Pharmacology* **47 Suppl 1**, 40-48 (1993).

1216. Nikawa T*, et al.* Vitamin A prevents the decline in immunoglobulin A and Th2 cytokine levels in small intestinal mucosa of protein-malnourished mice. *J Nutr* **129**, 934-941 (1999).

1217. Nilsson A, Duan RD. Pancreatic and mucosal enzymes in choline phospholipid digestion. *Am J Physiol Gastrointest Liver Physiol* **316**, G425-g445 (2019).

1218. Nishihata T, Lee CS, Yamamoto M, Rytting JH, Higuchi T. The effects of salicylate on the rectal absorption of phenylalanine and some peptides, and the effects of these peptides on the rectal absorption of cefoxitin and cefmetazole. *J Pharm Sci* **73**, 1326-1328 (1984).

1219. Nishiwaki H, Takeuchi K, Okada M, Tanaka H, Okabe S. Stimulation of gastric alkaline secretion by histamine in rats: possible involvement of histamine H2-receptors and endogenous prostaglandins. *J Pharmacol Exp Ther* **248**, 793-798 (1989).

1220. Nishiyama A*, et al.* Role of nitric oxide in regional blood flow in angiotensin II-induced hypertensive rats. *Hypertens Res* **24**, 421-427 (2001).

1221. Nissan A, Zhang JM, Lin Z, Haskel Y, Freund HR, Hanani M. The contribution of inflammatory mediators and nitric oxide to lipopolysaccharide-induced intussusception in mice. *J Surg Res* **69**, 205-207 (1997).

1222. Nissinen E, Lindén IB, Schultz E, Kaakkola S, Männistö PT, Pohto P. Inhibition of catechol-O-methyltransferase activity by two novel disubstituted catechols in the rat. *Eur J Pharmacol* **153**, 263-269 (1988).

1223. Niu L*, et al.* Different alterations in rat intestinal glutamine transport during the progression of CLP- and LPS-induced sepsis. *J Surg Res* **169**, 284-291 (2011).

1224. Njoku OO, Leitch GJ. Separation of cholera enterotoxin-induced mucus secretion from electrolyte secretion in rabbit ileum by acetazolamide, colchicine, cycloheximide, cytochalasin B and indomethacin. *Digestion* **27**, 174-184 (1983).

1225. Nobili F, Vignolini F, Figus E, Mengheri E. Treatment of rats with dexamethasone or thyroxine reverses zinc deficiency-induced intestinal damage. *J Nutr* **127**, 1807-1813 (1997).

1226. Noda S, Yamada A, Nakaoka K, Goseki-Sone M. 1-alpha,25-Dihydroxyvitamin D(3) up-regulates the expression of 2 types of human intestinal alkaline phosphatase alternative splicing variants in Caco-2 cells and may be an important regulator of their expression in gut homeostasis. *Nutr Res* **46**, 59-67 (2017).

1227. Noguchi Y, James JH, Fischer JE, Hasselgren PO. Increased glutamine consumption in small intestine epithelial cells during sepsis in rats. *Am J Surg* **173**, 199-205 (1997).

1228. Nogueira MC, Haibara AS, Borges EL. Effect of L-NAME microinjected into the nucleus tractus solitarius on jejunal glucose and electrolyte absorption in anesthetized rats. *Brain Res* **1359**, 107-115 (2010).

1229. Noh SK, Koo SI. Intraduodenal infusion of lysophosphatidylcholine restores the intestinal absorption of vitamins A and E in rats fed a low-zinc diet. *Exp Biol Med (Maywood)* **226**, 342-348 (2001).

1230. Nolan-Clark D, Tapsell LC, Hu R, Han DY, Ferguson LR. Effects of dairy products on crohn's disease symptoms are influenced by fat content and disease location but not lactose content or disease activity status in a New Zealand population. *J Am Diet Assoc* **111**, 1165-1172 (2011).

1231. Norlander B, Gotthard R, Ström M. Steady-state pharmacokinetics of enteric coated 5-amino-salicylic acid tablets in healthy volunteers and in patients with Crohn's disease or ulcerative colitis. *Aliment Pharmacol Ther* **5**, 291-300 (1991).

1232. Norman P, Abram TS, Cuthbert NJ, Gardiner PJ. The inhibition of [3H]leukotriene D4 binding to guinea-pig lung membranes. The correlation of binding affinity with activity on the guinea-pig ileum. *Eur J Pharmacol* **182**, 301-312 (1990).

1233. Noshiro H*, et al.* Gallstone formation and gallbladder bile composition after colectomy in dogs. *Dig Dis Sci* **41**, 2423-2432 (1996).

1234. Noteborn HP, Jansen E, Benito S, Mengelers MJ. Oral absorption and metabolism of quercetin and sugar-conjugated derivatives in specific transport systems. *Cancer Lett* **114**, 175-177 (1997).

1235. Novák P, Man P, Tucková L, Tlaskalová-Hogenová H, Bezouska K, Havlícek V. Monitoring of in vitro deamidation of gliadin peptic fragment by mass spectrometry may reflect one of the molecular mechanisms taking place in celiac disease development. *J Mass Spectrom* **37**, 507-511 (2002).

1236. Nowicki MJ, Shneider BL, Paul JM, Heubi JE. Glucocorticoids upregulate taurocholate transport by ileal brush-border membrane. *Am J Physiol* **273**, G197-203 (1997).

1237. Ntourakis D*, et al.* Adhesions and Healing of Intestinal Anastomoses: The Effect of Anti-Adhesion Barriers. *Surg Innov* **23**, 266-276 (2016).

1238. Nygård G*, et al.* Intestinal site-dependent susceptibility to chronic indomethacin in the rat: a morphological and biochemical study. *Aliment Pharmacol Ther* **9**, 403-410 (1995).

1239. Nylander O, Andersson H, Wilander E, Sababi M. Prostaglandins reduce hydrochloric acid-induced increase in duodenal mucosal permeability by a mechanism not related to stimulation of alkaline secretion. *Acta Physiol Scand* **153**, 365-374 (1995).

1240. Nylander O, Hällgren A, Sababi M. COX inhibition excites enteric nerves that affect motility, alkaline secretion, and permeability in rat duodenum. *Am J Physiol Gastrointest Liver Physiol* **281**, G1169-1178 (2001).

1241. O'Neill LAJ, Artyomov MN. Itaconate: the poster child of metabolic reprogramming in macrophage function. *Nat Rev Immunol* **19**, 273-281 (2019).

1242. Oakhill JS, Marritt SJ, Gareta EG, Cammack R, McKie AT. Functional characterization of human duodenal cytochrome b (Cybrd1): Redox properties in relation to iron and ascorbate metabolism. *Biochim Biophys Acta* **1777**, 260-268 (2008).

1243. Odes HS*, et al.* Effect of somatostatin-14 on duodenal mucosal bicarbonate secretion in guinea pigs. *Dig Dis Sci* **40**, 678-684 (1995).

1244. Ogihara T, Tamai I, Tsuji A. Application of fractal kinetics for carrier-mediated transport of drugs across intestinal epithelial membrane. *Pharm Res* **15**, 620-625 (1998).

1245. Oğülener N, Ergün Y, Döndaş N, Dikmen A. The influence of nitric oxide donors on the responses to nitrergic nerve stimulation in the mouse duodenum. *Eur J Pharmacol* **421**, 121-131 (2001).

1246. Ohnmacht C*, et al.* MUCOSAL IMMUNOLOGY. The microbiota regulates type 2 immunity through RORγt⁺ T cells. *Science* **349**, 989-993 (2015).

1247. Ohno Y, Lee J, Fusunyan RD, MacDermott RP, Sanderson IR. Macrophage inflammatory protein-2: chromosomal regulation in rat small intestinal epithelial cells. *Proc Natl Acad Sci U S A* **94**, 10279-10284 (1997).

1248. Ohtsu A*, et al.* Influence of Porphyromonas gingivalis in gut microbiota of streptozotocin-induced diabetic mice. *Oral Dis* **25**, 868-880 (2019).

1249. Ohtsu Y, Nakagawa Y, Nagasawa M, Takeda S, Arakawa H, Kojima I. Diverse signaling systems activated by the sweet taste receptor in human GLP-1-secreting cells. *Mol Cell Endocrinol* **394**, 70-79 (2014).

1250. Ohtsuka Y*, et al.* omega-3 fatty acids attenuate mucosal inflammation in premature rat pups. *J Pediatr Surg* **46**, 489-495 (2011).

1251. Ohue-Kitano R*, et al.* α-Linolenic acid-derived metabolites from gut lactic acid bacteria induce differentiation of anti-inflammatory M2 macrophages through G protein-coupled receptor 40. *Faseb j* **32**, 304-318 (2018).

1252. Okay E, Mutlu O, Gocmez SS, Oz S, Utkan T. N-Acetylcysteine improves disturbed ileal contractility following partial hepatectomy in rats. *J Gastroenterol Hepatol* **25**, 203-208 (2010).

1253. Okishio Y*, et al.* Mediators of nonadrenergic, noncholinergic relaxation in Sprague Dawley rat intestine: comparison with the mediators of other strains. *J Vet Med Sci* **62**, 821-828 (2000).

1254. Okuno T, Yokomizo T. Metabolism and biological functions of 12(S)-hydroxyheptadeca-5Z,8E,10E-trienoic acid. *Prostaglandins Other Lipid Mediat* **152**, 106502 (2020).

1255. Oliveira LP*, et al.* Genetic deletion of the angiotensin-(1-7) receptor Mas leads to alterations in gut villi length modulating TLR4/PI3K/AKT and produces microbiome dysbiosis. *Neuropeptides* **82**, 102056 (2020).

1256. Oliver SR, Phillips NA, Novosad VL, Bakos MP, Talbert EE, Clanton TL. Hyperthermia induces injury to the intestinal mucosa in the mouse: evidence for an oxidative stress mechanism. *Am J Physiol Regul Integr Comp Physiol* **302**, R845-853 (2012).

1257. Olivero David R*, et al.* Fasting status and thermally oxidized sunflower oil ingestion affect the intestinal antioxidant enzyme activity and gene expression of male Wistar rats. *J Agric Food Chem* **58**, 2498-2504 (2010).

1258. Ong J, Kerr DI, Capper HR, Johnston GA. Cortisone: a potent GABAA antagonist in the guinea-pig isolated ileum. *J Pharm Pharmacol* **42**, 662-664 (1990).

1259. Ong J, Kerr DI, Johnston GA. Cortisol: a potent biphasic modulator at GABAA-receptor complexes in the guinea pig isolated ileum. *Neurosci Lett* **82**, 101-106 (1987).

1260. Onoda Y, Magaribuchi T, Tamaki H. Effects of the new anti-ulcer agent 12-sulfodehydroabietic acid monosodium salt on duodenal alkaline secretion in rats. *Arzneimittelforschung* **40**, 576-578 (1990).

1261. Ooi JH, McDaniel KL, Weaver V, Cantorna MT. Murine CD8+ T cells but not macrophages express the vitamin D 1α-hydroxylase. *J Nutr Biochem* **25**, 58-65 (2014).

1262. Orihata M, Sarna SK. Inhibition of nitric oxide synthase delays gastric emptying of solid meals. *J Pharmacol Exp Ther* **271**, 660-670 (1994).

1263. Orihata M, Sarna SK. Nitric oxide mediates mechano- and chemoreceptor-activated intestinal feedback control of gastric emptying. *Dig Dis Sci* **41**, 1303-1309 (1996).

1264. Osthaus LE, Galligan JJ. Antagonists of nitric oxide synthesis inhibit nerve-mediated relaxations of longitudinal muscle in guinea pig ileum. *J Pharmacol Exp Ther* **260**, 140-145 (1992).

1265. Otles S, Ozgoz S. Health effects of dietary fiber. *Acta Sci Pol Technol Aliment* **13**, 191-202 (2014).

1266. Owczarek D, Rodacki T, Domagała-Rodacka R, Cibor D, Mach T. Diet and nutritional factors in inflammatory bowel diseases. *World J Gastroenterol* **22**, 895-905 (2016).

1267. Özozan Ö V, Ertorul D. Is tri-iodothyronine a better choice than activated protein C in sepsis treatment? *Ulus Travma Acil Cerrahi Derg* **25**, 545-554 (2019).

1268. Pácha J, Lisá V, Miksík I. Effect of cellular differentiation on 11beta-hydroxysteroid dehydrogenase activity in the intestine. *Steroids* **67**, 119-126 (2002).

1269. Pácha J, Miksík I. 11 beta-Hydroxysteroid dehydrogenase in developing rat intestine. *J Endocrinol* **148**, 561-566 (1996).

1270. Paiva TB, Mendes GB, Paiva AC. Specific desensitization (tachyphylaxis) of the guinea pig ileum to angiotensin II. *Am J Physiol* **232**, H223-230 (1977).

1271. Pang QF, Xu WL, He J, Chen HL. [The protective effect of glutamine on endotoxemic intestinal injury and expression of heme oxygenase-1 in rats]. *Zhongguo Wei Zhong Bing Ji Jiu Yi Xue* **23**, 95-98 (2011).

1272. Park I, Pasquetti T, Malheiros RD, Ferket PR, Kim SW. Effects of supplemental L-methionine on growth performance and redox status of turkey poults compared with the use of DL-methionine. *Poult Sci* **97**, 102-109 (2018).

1273. Patel BA*, et al.* Inhibitory neuromuscular transmission to ileal longitudinal muscle predominates in neonatal guinea pigs. *Neurogastroenterol Motil* **22**, 909-918, e236-907 (2010).

1274. Patrignani F, Parolin C, D'Alessandro M, Siroli L, Vitali B, Lanciotti R. Evaluation of the fate of Lactobacillus crispatus BC4, carried in Squacquerone cheese, throughout the simulator of the human intestinal microbial ecosystem (SHIME). *Food Res Int* **137**, 109580 (2020).

1275. Patten GS, Augustin MA, Sanguansri L, Head RJ, Abeywardena MY. Site specific delivery of microencapsulated fish oil to the gastrointestinal tract of the rat. *Dig Dis Sci* **54**, 511-521 (2009).

1276. Patterson LT, Nahrwold DL, Rose RC. Ascorbic acid uptake in guinea pig intestinal mucosa. *Life Sci* **31**, 2783-2791 (1982).

1277. Paul W*, et al.* Cutaneous permeability responses to bradykinin and histamine in the guinea-pig: possible differences in their mechanism of action. *Br J Pharmacol* **111**, 159-164 (1994).

1278. Pauletzki JG, Sharkey KA, Davison JS, Bomzon A, Shaffer EA. Involvement of L-arginine-nitric oxide pathways in neural relaxation of the sphincter of Oddi. *Eur J Pharmacol* **232**, 263-270 (1993).

1279. Pavlinov SA, Isachenkov VA. [Absence of hydroxyindole O-methyltransferase, the terminal enzyme in melatonin synthesis, in the rat intestine]. *Probl Endokrinol (Mosk)* **24**, 102-106 (1978).

1280. Pawlik WW*, et al.* Microcirculatory and motor effects of endogenous nitric oxide in the rat gut. *J Physiol Pharmacol* **44**, 139-146 (1993).

1281. Pei Y*, et al.* Impact of plant extract on the gastrointestinal fate of nutraceutical-loaded nanoemulsions: phytic acid inhibits lipid digestion but enhances curcumin bioaccessibility. *Food Funct* **10**, 3344-3355 (2019).

1282. Pellicciari R*, et al.* Brush-border-enzyme-mediated intestine-specific drug delivery. Amino acid prodrugs of 5-aminosalicylic acid. *J Med Chem* **36**, 4201-4207 (1993).

1283. Pencheva N. Dependence of gamma-aminobutyric acid modulation of cholinergic transmission on nitric oxide and purines in cat terminal ileum. *Eur J Pharmacol* **339**, 193-200 (1997).

1284. Pender SL, Quinn JJ, Sanderson IR, MacDonald TT. Butyrate upregulates stromelysin-1 production by intestinal mesenchymal cells. *Am J Physiol Gastrointest Liver Physiol* **279**, G918-924 (2000).

1285. Pento JT. The influence of interrrupted vitamin D metabolism on acute low calcium adaptation in the rat. *Nutr Metab* **20**, 321-328 (1976).

1286. Pereira MA, Bagatin MC, Zanoni JN. Effects of the ascorbic acid supplementation on NADH-diaphorase myenteric neurons in the duodenum of diabetic rats. *Biocell* **30**, 295-300 (2006).

1287. Pereira RV, de Miranda-Neto MH, da Silva Souza ID, Zanoni JN. Vitamin E supplementation in rats with experimental diabetes mellitus: analysis of myosin-V and nNOS immunoreactive myenteric neurons from terminal ileum. *J Mol Histol* **39**, 595-603 (2008).

1288. Pereira RV, Linden DR, Miranda-Neto MH, Zanoni JN. Differential effects in CGRPergic, nitrergic, and VIPergic myenteric innervation in diabetic rats supplemented with 2% L-glutamine. *An Acad Bras Cienc* **88 Suppl 1**, 609-622 (2016).

1289. Pereira RV, Tronchini EA, Tashima CM, Alves EP, Lima MM, Zanoni JN. L-glutamine supplementation prevents myenteric neuron loss and has gliatrophic effects in the ileum of diabetic rats. *Dig Dis Sci* **56**, 3507-3516 (2011).

1290. Pérez-Valentín MA*, et al.* [Atypical cytomegalovirus in renal transplantation: a new form of presentation]. *Nefrologia* **22**, 381-385 (2002).

1291. Pérez-Vicente A, Gil-Izquierdo A, García-Viguera C. In vitro gastrointestinal digestion study of pomegranate juice phenolic compounds, anthocyanins, and vitamin C. *J Agric Food Chem* **50**, 2308-2312 (2002).

1292. Peri KG, Hardy P, Li DY, Varma DR, Chemtob S. Prostaglandin G/H synthase-2 is a major contributor of brain prostaglandins in the newborn. *J Biol Chem* **270**, 24615-24620 (1995).

1293. Peter G, Borbe HO. Absorption of [7,8-14C]rac-a-lipoic acid from in situ ligated segments of the gastrointestinal tract of the rat. *Arzneimittelforschung* **45**, 293-299 (1995).

1294. Petkov V, Radomirov R. Influence of indomethacin and aspirin on the contractile effects of prostaglandin F2alpha (PGF2alpha) at different Ca++ concentrations (experiments on guinea-pig ileum). *Acta Physiol Pharmacol Bulg* **3**, 18-23 (1977).

1295. Petrishchev NN, Shliakhto EV, Tsyrlin VA, Vlasov TD, Syrenskiĭ AV, Galagudza MM. [The role of oxygen free radicals in the mechanisms of local and distant ischemic myocardial preconditioning]. *Vestn Ross Akad Med Nauk*, 10-15 (2006).

1296. Petry AL, Huntley NF, Bedford MR, Patience JF. Xylanase increased the energetic contribution of fiber and improved the oxidative status, gut barrier integrity, and growth performance of growing pigs fed insoluble corn-based fiber. *J Anim Sci* **98**, (2020).

1297. Pfeiffer AFH, Keyhani-Nejad F. High Glycemic Index Metabolic Damage - a Pivotal Role of GIP and GLP-1. *Trends Endocrinol Metab* **29**, 289-299 (2018).

1298. Pfeiffer C, Bach M, Bauer T, Campos da Ponte J, Schömig E, Gründemann D. Knockout of the ergothioneine transporter ETT in zebrafish results in increased 8-oxoguanine levels. *Free Radic Biol Med* **83**, 178-185 (2015).

1299. Phillips JD, Kim CS, Fonkalsrud EW, Zeng H, Dindar H. Effects of chronic corticosteroids and vitamin A on the healing of intestinal anastomoses. *Am J Surg* **163**, 71-77 (1992).

1300. Phull PS, Price AB, Thorniley MS, Green CJ, Jacyna MR. Vitamin E concentrations in the human stomach and duodenum--correlation with Helicobacter pylori infection. *Gut* **39**, 31-35 (1996).

1301. Pianou NK, Stavrou PZ, Vlontzou E, Rondogianni P, Exarhos DN, Datseris IE. More advantages in detecting bone and soft tissue metastases from prostate cancer using (18)F-PSMA PET/CT. *Hell J Nucl Med* **22**, 6-9 (2019).

1302. Piatek-Guziewicz A*, et al.* Intestinal parameters of oxidative imbalance in celiac adults with extraintestinal manifestations. *World J Gastroenterol* **23**, 7849-7862 (2017).

1303. Pierce JV, Dignard D, Whiteway M, Kumamoto CA. Normal adaptation of Candida albicans to the murine gastrointestinal tract requires Efg1p-dependent regulation of metabolic and host defense genes. *Eukaryot Cell* **12**, 37-49 (2013).

1304. Pinto L*, et al.* Endocannabinoids as physiological regulators of colonic propulsion in mice. *Gastroenterology* **123**, 227-234 (2002).

1305. Piovani D, Danese S, Peyrin-Biroulet L, Nikolopoulos GK, Lytras T, Bonovas S. Environmental Risk Factors for Inflammatory Bowel Diseases: An Umbrella Review of Meta-analyses. *Gastroenterology* **157**, 647-659.e644 (2019).

1306. Pirgozliev VR*, et al.* Feeding dihydroquercetin and vitamin E to broiler chickens reared at standard and high ambient temperatures. *Arch Anim Nutr* **74**, 496-511 (2020).

1307. Pironi L*, et al.* Evaluation of oral administration of folic and folinic acid to prevent folate deficiency in patients with inflammatory bowel disease treated with salicylazosulfapyridine. *Int J Clin Pharmacol Res* **8**, 143-148 (1988).

1308. Pirozzi C*, et al.* Hydroxytyrosol prevents metabolic impairment reducing hepatic inflammation and restoring duodenal integrity in a rat model of NAFLD. *J Nutr Biochem* **30**, 108-115 (2016).

1309. Piva A, Pizzamiglio V, Morlacchini M, Tedeschi M, Piva G. Lipid microencapsulation allows slow release of organic acids and natural identical flavors along the swine intestine. *J Anim Sci* **85**, 486-493 (2007).

1310. Pizcueta P*, et al.* Modulation of the hyperdynamic circulation of cirrhotic rats by nitric oxide inhibition. *Gastroenterology* **103**, 1909-1915 (1992).

1311. Poćwiardowska-Ciara E, Korolkiewicz Z. Ascorbic acid, PGE2 and acetylcholine interaction: the effect on isolated smooth muscle. *Acta Physiol Pol* **37**, 18-24 (1986).

1312. Pointner H, Kletter K. Evaluation of the 75Se-l-selenomethionine test for pancreatic disease. Studies on gel filtration. *Digestion* **20**, 225-233 (1980).

1313. Pol O, Sasaki M, Jiménez N, Dawson VL, Dawson TM, Puig MM. The involvement of nitric oxide in the enhanced expression of mu-opioid receptors during intestinal inflammation in mice. *Br J Pharmacol* **145**, 758-766 (2005).

1314. Pontieri V, Lopes OU, Ferreira SH. Hypotensive effect of captopril. Role of bradykinin and prostaglandinlike substances. *Hypertension* **15**, I55-58 (1990).

1315. Poole DP*, et al.* Expression and function of the bile acid receptor GpBAR1 (TGR5) in the murine enteric nervous system. *Neurogastroenterol Motil* **22**, 814-825, e227-818 (2010).

1316. Postorino A, Serio R, Mulè F. Nitric oxide is involved in non-adrenergic, non-cholinergic inhibitory neurotransmission in rat duodenum. *J Auton Pharmacol* **15**, 65-71 (1995).

1317. Pothoulakis C*, et al.* Ketotifen inhibits Clostridium difficile toxin A-induced enteritis in rat ileum. *Gastroenterology* **105**, 701-707 (1993).

1318. Prabhu R, Anup R, Balasubramanian KA. Surgical stress induces phospholipid degradation in the intestinal brush border membrane. *J Surg Res* **94**, 178-184 (2000).

1319. Primi MP, Bueno L. Central nervous system influence of prostaglandin E2 on jejunal water and electrolyte transport in conscious dogs. *Gastroenterology* **91**, 1427-1432 (1986).

1320. Prins HA*, et al.* The effect of mild endotoxemia during low arginine plasma levels on organ blood flow in rats. *Crit Care Med* **28**, 1991-1997 (2000).

1321. Provan WM, Eyton-Jones H, Lappin G, Pritchard D, Moore RB, Green T. The incorporation of radiolabelled sulphur from captan into protein and its impact on a DNA binding study. *Chem Biol Interact* **96**, 173-184 (1995).

1322. Pushkin A*, et al.* Structural characterization, tissue distribution, and functional expression of murine aminoacylase III. *Am J Physiol Cell Physiol* **286**, C848-856 (2004).

1323. Pytka K, Głuch-Lutwin M, Knutelska J, Jakubczyk M, Waszkielewicz A, Kotańska M. HBK-14 and HBK-15 Do Not Influence Blood Pressure, Lipid Profile, Glucose Level, or Liver Enzymes Activity after Chronic Treatment in Rats. *PLoS One* **11**, e0165495 (2016).

1324. Pytkowski B. On the contribution of prostaglandin-like substances to the action of bradykinin on intestinal motility and blood flow in canine jejunal loop in situ. *Eur J Clin Invest* **9**, 391-396 (1979).

1325. Qin DP, Wei X, Fang GD, Yang F, Lai DP. [Intervention Effect of Modified Dachengqi Decoction on Intestinal Mucosal Barrier of Severe Acute Pancreatitis Model Rats]. *Zhongguo Zhong Xi Yi Jie He Za Zhi* **35**, 1482-1489 (2015).

1326. Qiu B, Pothoulakis C, Castagliuolo I, Nikulasson Z, LaMont JT. Nitric oxide inhibits rat intestinal secretion by Clostridium difficile toxin A but not Vibrio cholerae enterotoxin. *Gastroenterology* **111**, 409-418 (1996).

1327. Qiu X, Ye Q, Sun M, Wang L, Tan Y, Wu G. Saturated hydrogen improves lipid metabolism disorders and dysbacteriosis induced by a high-fat diet. *Exp Biol Med (Maywood)* **245**, 512-521 (2020).

1328. Qu J, Ko CW, Tso P, Bhargava A. Apolipoprotein A-IV: A Multifunctional Protein Involved in Protection against Atherosclerosis and Diabetes. *Cells* **8**, (2019).

1329. Quaroni A. Pre- and postnatal development of differentiated functions in rat intestinal epithelial cells. *Dev Biol* **111**, 280-292 (1985).

1330. Quick TC, Ong DE. Vitamin A metabolism in the human intestinal Caco-2 cell line. *Biochemistry* **29**, 11116-11123 (1990).

1331. Quinson N, Catalin D, Niel JP, Miolan JP. Release of nitric oxide within the coeliac plexus is involved in the organization of a gastroduodenal inhibitory reflex in the rabbit. *J Physiol* **519 Pt 1**, 223-234 (1999).

1332. Quinson N, Niel JP, Miolan JP. Nitric oxide released by gastric mechanoreceptors modulates nicotinic activation of coeliac plexus neurons in the rabbit. *Eur J Neurosci* **12**, 1521-1524 (2000).

1333. Quiros M*, et al.* Resolvin E1 is a pro-repair molecule that promotes intestinal epithelial wound healing. *Proc Natl Acad Sci U S A* **117**, 9477-9482 (2020).

1334. Radomirov R, Petkov V, Davidoff M. Neurotransmitted effect of prostaglandin F2 alpha in isolated cat jejunum. *Methods Find Exp Clin Pharmacol* **5**, 275-279 (1983).

1335. Ragy M, Elbassuoni E. The role of nitric oxide and L-type calcium channel blocker in the contractility of rabbit ileum in vitro. *J Physiol Biochem* **68**, 521-528 (2012).

1336. Rahmati R. The transient receptor potential vanilloid receptor 1, TRPV1 (VR1) inhibits peristalsis in the mouse jejunum. *Arch Iran Med* **15**, 433-438 (2012).

1337. Rai R, Saraswat VA, Dhiman RK. Gut microbiota: its role in hepatic encephalopathy. *J Clin Exp Hepatol* **5**, S29-36 (2015).

1338. Raimundo AH, Patil DH, Frost PG, Silk DB. Effects of olsalazine and sulphasalazine on jejunal and ileal water and electrolyte absorption in normal human subjects. *Gut* **32**, 270-274 (1991).

1339. Rainsford KD. Ibuprofen: pharmacology, efficacy and safety. *Inflammopharmacology* **17**, 275-342 (2009).

1340. Raj AS*, et al.* Dysbiosis of the Duodenal Mucosal Microbiota Is Associated With Increased Small Intestinal Permeability in Chronic Liver Disease. *Clin Transl Gastroenterol* **10**, e00068 (2019).

1341. Rakestraw PC, Snyder JR, Woliner MJ, Sanders KM, Shuttleworth CW. Involvement of nitric oxide in inhibitory neuromuscular transmission in equine jejunum. *Am J Vet Res* **57**, 1206-1213 (1996).

1342. Rao CV, Tokumo K, Rigotty J, Zang E, Kelloff G, Reddy BS. Chemoprevention of colon carcinogenesis by dietary administration of piroxicam, alpha-difluoromethylornithine, 16 alpha-fluoro-5-androsten-17-one, and ellagic acid individually and in combination. *Cancer Res* **51**, 4528-4534 (1991).

1343. Rao RK, Riviere PJ, Pascaud X, Junien JL, Porreca F. Tonic regulation of mouse ileal ion transport by nitric oxide. *J Pharmacol Exp Ther* **269**, 626-631 (1994).

1344. Rasmussen M, Petersen LB, Norum KR. The activity of acyl CoA: retinol acyltransferase in the rat: variation with vitamin A status. *Br J Nutr* **51**, 245-253 (1984).

1345. Raul F, Noriega R, Nsi-Emvo E, Doffoel M, Grenier JF. Lactase activity is under hormonal control in the intestine of adult rat. *Gut* **24**, 648-652 (1983).

1346. Reasbeck PG, Barbezat GO, Weber FL, Jr., Robinson MF, Thomson CD. Selenium absorption by canine jejunum. *Dig Dis Sci* **30**, 489-494 (1985).

1347. Reddy BS, Nayini J, Tokumo K, Rigotty J, Zang E, Kelloff G. Chemoprevention of colon carcinogenesis by concurrent administration of piroxicam, a nonsteroidal antiinflammatory drug with D,L-alpha-difluoromethylornithine, an ornithine decarboxylase inhibitor, in diet. *Cancer Res* **50**, 2562-2568 (1990).

1348. Reddy MB, Chidambaram MV, Fonseca J, Bates GW. Potential role of in vitro iron bioavailability studies in combatting iron deficiency: a study of the effects of phosvitin on iron mobilization from pinto beans. *Clin Physiol Biochem* **4**, 78-86 (1986).

1349. Redmond TM*, et al.* Identification, expression, and substrate specificity of a mammalian beta-carotene 15,15'-dioxygenase. *J Biol Chem* **276**, 6560-6565 (2001).

1350. Rehal S, von der Weid PY. Experimental ileitis alters prostaglandin biosynthesis in mesenteric lymphatic and blood vessels. *Prostaglandins Other Lipid Mediat* **116-117**, 37-48 (2015).

1351. Rehman H*, et al.* In vitro effects of alpha toxin from Clostridium perfringens on the electrophysiological parameters of jejunal tissues from laying hens preincubated with inulin and N-acetyl-L-cysteine. *Poult Sci* **88**, 199-204 (2009).

1352. Rehman ZU*, et al.* Supplementation of Vitamin E Protects Chickens from Newcastle Disease Virus-Mediated Exacerbation of Intestinal Oxidative Stress and Tissue Damage. *Cell Physiol Biochem* **47**, 1655-1666 (2018).

1353. Rehn M, Hild D, Diener M. Upregulation of cyclooxygenase-2 and thromboxane A2 production mediate the action of tumor necrosis factor-alpha in isolated rat myenteric ganglia. *Am J Physiol Gastrointest Liver Physiol* **289**, G586-591 (2005).

1354. Reichardt SD*, et al.* Glucocorticoids enhance intestinal glucose uptake via the dimerized glucocorticoid receptor in enterocytes. *Endocrinology* **153**, 1783-1794 (2012).

1355. Reilly MA, Schayer RW. Effect of glucocorticoids on histamine metabolism in mice. *Br J Pharmacol* **45**, 463-469 (1972).

1356. Reimers J*, et al.* Lack of insulinotropic effect of endogenous and exogenous cholecystokinin in man. *Diabetologia* **31**, 271-280 (1988).

1357. Reims A, Redfors S, Sjövall H, Strandvik B. Cysteinyl leukotrienes are secretagogues in atrophic coeliac and in normal duodenal mucosa of children. *Scand J Gastroenterol* **40**, 160-168 (2005).

1358. Reinhold JG, Garcia Estrada J, Garcia PM, Garzon P. Retention of iron by rat intestine in vivo as affected by dietary fiber, ascorbate and citrate. *J Nutr* **116**, 1007-1017 (1986).

1359. Reis E*, et al.* Effects of octreotide and a-tocopherol on bacterial translocation in experimental intestinal obstruction: a microbiological, light and electronmicroscopical study. *Hepatogastroenterology* **44**, 656-663 (1997).

1360. Reisenauer AM, Halsted CH. Human jejunal brush border folate conjugase. Characteristics and inhibition by salicylazosulfapyridine. *Biochim Biophys Acta* **659**, 62-69 (1981).

1361. Rekik M, Delvaux M, Frexinos J, Bueno L. The calcitonin gene-related peptide activates both cAMP and NO pathways to induce relaxation of circular smooth muscle cells of guinea-pig ileum. *Peptides* **18**, 1517-1522 (1997).

1362. Rémond D*, et al.* Cysteine fluxes across the portal-drained viscera of enterally fed minipigs: effect of an acute intestinal inflammation. *Amino Acids* **40**, 543-552 (2011).

1363. Ren NSX*, et al.* Haploinsufficiency of SIRT1 Enhances Glutamine Metabolism and Promotes Cancer Development. *Curr Biol* **27**, 483-494 (2017).

1364. Renzi D, Evangelista S, Mantellini P, Surrenti C. Decrease of duodenal calcitonin gene-related peptide- and substance P-like immunoreactivity in rat duodenal ulcers. *Adv Exp Med Biol* **298**, 129-135 (1991).

1365. Rhaleb NE, Carretero OA. Role of B1 and B2 receptors and of nitric oxide in bradykinin-induced relaxation and contraction of isolated rat duodenum. *Life Sci* **55**, 1351-1363 (1994).

1366. Rhee JC, Chang TM, Lee KY, Jo YH, Chey WY. Mechanism of oleic acid-induced inhibition on gastric acid secretion in rats. *Am J Physiol* **260**, G564-570 (1991).

1367. Rhoads JM, Argenzio RA, Chen W, Gomez GG. Asparagine stimulates piglet intestinal Cl- secretion by a mechanism requiring a submucosal glutamate receptor and nitric oxide. *J Pharmacol Exp Ther* **274**, 404-412 (1995).

1368. Riabchenko NI*, et al.* [Radioprotective and antistressful properties of nitric oxide production modulators]. *Radiats Biol Radioecol* **45**, 68-72 (2005).

1369. Ribaya-Mercado JD, Blanco MC, Fox JG, Russell RM. High concentrations of vitamin A esters circulate primarily as retinyl stearate and are stored primarily as retinyl palmitate in ferret tissues. *J Am Coll Nutr* **13**, 83-86 (1994).

1370. Ribaya-Mercado JD, Fox JG, Rosenblad WD, Blanco MC, Russell RM. Beta-carotene, retinol and retinyl ester concentrations in serum and selected tissues of ferrets fed beta-carotene. *J Nutr* **122**, 1898-1903 (1992).

1371. Riehl TE, Newberry RD, Lorenz RG, Stenson WF. TNFR1 mediates the radioprotective effects of lipopolysaccharide in the mouse intestine. *Am J Physiol Gastrointest Liver Physiol* **286**, G166-173 (2004).

1372. Rinsema W, Gouma DJ, von Meyenfeldt MF, Soeters PB. Reinfusion of secretions from high-output proximal stomas or fistulas. *Surg Gynecol Obstet* **167**, 372-376 (1988).

1373. Rivoira MA, Marchionatti AM, Centeno VA, Díaz de Barboza GE, Peralta López ME, Tolosa de Talamoni NG. Sodium deoxycholate inhibits chick duodenal calcium absorption through oxidative stress and apoptosis. *Comp Biochem Physiol A Mol Integr Physiol* **162**, 397-405 (2012).

1374. Robert A, Lancaster C, Davis JP, Kolbasa KP, Nezamis JE. Ulcer formation and cytoprotection by acetazolamide. *Eur J Pharmacol* **118**, 193-201 (1985).

1375. Robert A, Schultz JR, Nezamis JE, Lancaster C. Gastric antisecretory and antiulcer properties of PGE2, 15-methyl PGE2, and 16, 16-dimethyl PGE2. Intravenous, oral and intrajejunal administration. *Gastroenterology* **70**, 359-370 (1976).

1376. Robert A, Tabata K, Joffe SN, Jacobson ED. Prostaglandin deficiency by itself is not the cause of mepirizole-induced duodenal ulcers in rats. *Dig Dis Sci* **32**, 997-1003 (1987).

1377. Roberton AM, Rabel B, Stubbs L, Tasman-Jones C, Lee SP. Aspirin changes the secretion rate and amino acid composition of human small intestinal mucin in subjects with ileal conduits. *Glycoconj J* **13**, 781-789 (1996).

1378. Robinson P, Martin P, Jr., Garza A, D'Souza M, Mastrangelo MA, Tweardy D. Substance P receptor antagonism for treatment of cryptosporidiosis in immunosuppressed mice. *J Parasitol* **94**, 1150-1154 (2008).

1379. Rochell SJ, Parsons CM, Dilger RN. Effects of Eimeria acervulina infection severity on growth performance, apparent ileal amino acid digestibility, and plasma concentrations of amino acids, carotenoids, and α1-acid glycoprotein in broilers. *Poult Sci* **95**, 1573-1581 (2016).

1380. Rodenburg W*, et al.* Salmonella induces prominent gene expression in the rat colon. *BMC Microbiol* **7**, 84 (2007).

1381. Rodriguez JA, Torbati D, Washington T, Espinoza CG, Heneghan JB, O'Leary JP. Jejunoileal bypass-induced liver dysfunction and bacterial translocation: effect of intraluminal glutamine-infusion. *Am Surg* **61**, 397-402 (1995).

1382. Roels F, Espeel M, Pauwels M, De Craemer D, Egberts HJ, van der Spek P. Different types of peroxisomes in human duodenal epithelium. *Gut* **32**, 858-865 (1991).

1383. Roenspies U, Saegesser F. [Behcet's disease and toxic megacolon]. *Schweiz Med Wochenschr* **105**, 199-204 (1975).

1384. Rogers EL, Douglass W, Russell RM, Bushman L, Hubbard TB, Iber FL. Deficiency of fat soluble vitamins after jejunoileal bypass surgery for morbid obesity. *Am J Clin Nutr* **33**, 1208-1214 (1980).

1385. Rogers JO, Black BL. The effect of hydrocortisone and thyroxine on development of calcium homeostasis in embryonic intestinal epithelium. *Experientia* **52**, 558-563 (1996).

1386. Rolfe V, Levin RJ. Enterotoxin Escherichia coli STa activates a nitric oxide-dependent myenteric plexus secretory reflex in the rat ileum. *J Physiol* **475**, 531-537 (1994).

1387. Rolfe VE, Brand MP, Heales SJ, Lindley KJ, Milla PJ. Tetrahydrobiopterin regulates cyclic GMP-dependent electrogenic Cl- secretion in mouse ileum in vitro. *J Physiol* **503 ( Pt 2)**, 347-352 (1997).

1388. Roma E, Klontza D, Kairis M, Pangalis A, Karpouzas J, Matsaniotis N. Familial hypobetalipoproteinaemia. *Helv Paediatr Acta* **39**, 145-151 (1984).

1389. Romanato G*, et al.* Lipid and phospholipid profile after bowel resection for Crohn's disease. *Int J Colorectal Dis* **23**, 931-938 (2008).

1390. Roos C, Dahlgren D, Sjögren E, Sjöblom M, Hedeland M, Lennernäs H. Jejunal absorption of aprepitant from nanosuspensions: Role of particle size, prandial state and mucus layer. *Eur J Pharm Biopharm* **132**, 222-230 (2018).

1391. Roquet J, Nockels CF, Papas AM. Cattle blood plasma and red blood cell alpha-tocopherol levels in response to different chemical forms and routes of administration of vitamin E. *J Anim Sci* **70**, 2542-2550 (1992).

1392. Rosa EF, Freymüller E, Ihara SS, Aboulafia J, Nouailhetas VL. Damaging effects of intense repetitive treadmill running on murine intestinal musculature. *J Appl Physiol (1985)* **104**, 1410-1417 (2008).

1393. Rosa EF, Ribeiro RF, Pereira FM, Freymüller E, Aboulafia J, Nouailhetas VL. Vitamin C and E supplementation prevents mitochondrial damage of ileum myocytes caused by intense and exhaustive exercise training. *J Appl Physiol (1985)* **107**, 1532-1538 (2009).

1394. Rossi RE, Whyand T, Murray CD, Hamilton MI, Conte D, Caplin ME. The role of dietary supplements in inflammatory bowel disease: a systematic review. *Eur J Gastroenterol Hepatol* **28**, 1357-1364 (2016).

1395. Rufo PA, Bousvaros A. Current therapy of inflammatory bowel disease in children. *Paediatr Drugs* **8**, 279-302 (2006).

1396. Rühl A, Berezin I, Collins SM. Involvement of eicosanoids and macrophage-like cells in cytokine-mediated changes in rat myenteric nerves. *Gastroenterology* **109**, 1852-1862 (1995).

1397. Russell DA, Castro GA. Immunological regulation of colonic ion transport. *Am J Physiol* **256**, G396-403 (1989).

1398. Russo A, Fraser R, Adachi K, Horowitz M, Boeckxstaens G. Evidence that nitric oxide mechanisms regulate small intestinal motility in humans. *Gut* **44**, 72-76 (1999).

1399. Rybicki KJ, Longhurst JC, Kaufman MP. Stimulation of splanchnic afferents reflexly relaxes tracheal smooth muscle in dogs. *J Appl Physiol Respir Environ Exerc Physiol* **55**, 427-432 (1983).

1400. Saario R, Leino R, Lahesmaa R, Granfors K, Toivanen A. Function of terminal ileum in patients with Yersinia-triggered reactive arthritis. *J Intern Med* **232**, 73-76 (1992).

1401. Saavedra Y, Vergara P. Somatostatin inhibits intestinal mucosal mast cell degranulation in normal conditions and during mast cell hyperplasia. *Regul Pept* **111**, 67-75 (2003).

1402. Sababi M, Nilsson E, Holm L. Mucus and alkali secretion in the rat duodenum: effects of indomethacin, N omega-nitro-L-arginine, and luminal acid. *Gastroenterology* **109**, 1526-1534 (1995).

1403. Sababi M, Nylander O. Comparative study of the effects of nitric oxide synthase and cyclo-oxygenase inhibition on duodenal functions in rats anaesthetized with inactin, urethane or alpha-chloralose. *Acta Physiol Scand* **158**, 45-52 (1996).

1404. Saghaei F, Karimi I, Jouyban A, Samini M. Effects of captopril on the cysteamine-induced duodenal ulcer in the rat. *Exp Toxicol Pathol* **64**, 373-377 (2012).

1405. Saha L. Role of peroxisome proliferator-activated receptors alpha and gamma in gastric ulcer: An overview of experimental evidences. *World J Gastrointest Pharmacol Ther* **6**, 120-126 (2015).

1406. Said HM. Movement of biotin across the rat intestinal basolateral membrane. Studies with membrane vesicles. *Biochem J* **279 ( Pt 3)**, 671-674 (1991).

1407. Said HM, Horne DW, Wagner C. Effect of human milk folate binding protein on folate intestinal transport. *Arch Biochem Biophys* **251**, 114-120 (1986).

1408. Said HM, Redha R, Nylander W. A carrier-mediated, Na+ gradient-dependent transport for biotin in human intestinal brush-border membrane vesicles. *Am J Physiol* **253**, G631-636 (1987).

1409. Said HM, Strum WB. Cyclic adenosine-3',5'-monophosphate and folate transport in rat jejunum. *Biochem Biophys Res Commun* **115**, 756-761 (1983).

1410. Saito H*, et al.* [Studies on stable control of blood sugar by continuous administration of insulin through isolated intestinal loop]. *Nihon Geka Gakkai Zasshi* **93**, 16-25 (1992).

1411. Saito K, Crowley JS, Markey SP, Heyes MP. A mechanism for increased quinolinic acid formation following acute systemic immune stimulation. *J Biol Chem* **268**, 15496-15503 (1993).

1412. Sakkas P*, et al.* Does selection for growth rate in broilers affect their resistance and tolerance to Eimeria maxima? *Vet Parasitol* **258**, 88-98 (2018).

1413. Salmenkari H, Issakainen T, Vapaatalo H, Korpela R. Local corticosterone production and angiotensin-I converting enzyme shedding in a mouse model of intestinal inflammation. *World J Gastroenterol* **21**, 10072-10079 (2015).

1414. Sampei K, Ulatowski JA, Asano Y, Kwansa H, Bucci E, Koehler RC. Role of nitric oxide scavenging in vascular response to cell-free hemoglobin transfusion. *Am J Physiol Heart Circ Physiol* **289**, H1191-1201 (2005).

1415. Sand E, Themner-Persson A, Ekblad E. Mast cells reduce survival of myenteric neurons in culture. *Neuropharmacology* **56**, 522-530 (2009).

1416. Sand J*, et al.* The inhibitory role of nitric oxide in the control of porcine and human sphincter of Oddi activity. *Gut* **41**, 375-380 (1997).

1417. Sandberg M, Jansson L. Effects of cyclooxygenase inhibition on insulin release and pancreatic islet blood flow in rats. *Ups J Med Sci* **119**, 316-323 (2014).

1418. Sanders KM. Evidence that prostaglandins are local regulatory agents in canine ileal circular muscle. *Am J Physiol* **246**, G361-371 (1984).

1419. Sanders KM, Ross G. Effects of endogenous prostaglandin E on intestinal motility. *Am J Physiol* **234**, E204-208 (1978).

1420. Sandle GI, Keir MJ, Record CO. The effect of hydrocortisone on the transport of water, sodium, and glucose in the jejunum. Perfusion studies in normal subjects and patients with coeliac disease. *Scand J Gastroenterol* **16**, 667-671 (1981).

1421. Sandstrom P, Woods CM, Brooke-Smith M, Saccone GT, Toouli J, Svanvik J. Highly selective iNOS inhibition and sphincter of Oddi motility in the Australian possum. *Acta Physiol Scand* **181**, 321-331 (2004).

1422. Sanger GJ, Watt AJ. Some mechanism which may modulate noradrenaline release in guinea-pig isolated ileum. *J Pharm Pharmacol* **32**, 188-191 (1980).

1423. Sanghani SP, Davis WI, Dumaual NG, Mahrenholz A, Bosron WF. Identification of microsomal rat liver carboxylesterases and their activity with retinyl palmitate. *Eur J Biochem* **269**, 4387-4398 (2002).

1424. Sangild PT, Diernaes L, Christiansen IJ, Skadhauge E. Intestinal transport of sodium, glucose and immunoglobulin in neonatal pigs. Effect of glucocorticoids. *Exp Physiol* **78**, 485-497 (1993).

1425. Sann H, Dux M, Schemann M, Jancsó G. Neurogenic inflammation in the gastrointestinal tract of the rat. *Neurosci Lett* **219**, 147-150 (1996).

1426. Santos AR, Miguel OG, Yunes RA, Calixto JB. Antinociceptive properties of the new alkaloid, cis-8, 10-di-N-propyllobelidiol hydrochloride dihydrate isolated from Siphocampylus verticillatus: evidence for the mechanism of action. *J Pharmacol Exp Ther* **289**, 417-426 (1999).

1427. Santos J*, et al.* Characterisation of immune mediator release during the immediate response to segmental mucosal challenge in the jejunum of patients with food allergy. *Gut* **45**, 553-558 (1999).

1428. Santos RG*, et al.* Effects of nitric oxide synthase inhibition on glutamine action in a bacterial translocation model. *Br J Nutr* **111**, 93-100 (2014).

1429. Sarantos P, Chakrabarti R, Copeland EM, Souba WW. Dexamethasone increases jejunal glutamine synthetase expression via translational regulation. *Am J Surg* **167**, 8-13 (1994).

1430. Sarkar K, Tarafder P, Paul G. Bisphenol A inhibits duodenal movement ex vivo of rat through nitric oxide-mediated soluble guanylyl cyclase and α-adrenergic signaling pathways. *J Appl Toxicol* **36**, 131-139 (2016).

1431. Sarna SK, Otterson MF, Ryan RP, Cowles VE. Nitric oxide regulates migrating motor complex cycling and its postprandial disruption. *Am J Physiol* **265**, G749-766 (1993).

1432. Sasaki Y, Aihara E, Ise F, Kita K, Takeuchi K. Stimulatory effect of Coca-Cola on gastroduodenal HCO3- secretion in rats. *Inflammopharmacology* **15**, 223-228 (2007).

1433. Sasaki Y*, et al.* Stimulation by sparkling water of gastroduodenal HCO3- secretion in rats. *Med Sci Monit* **15**, Br349-356 (2009).

1434. Sasikaran J, Ziemski M, Zadora PK, Fleig A, Berg IA. Bacterial itaconate degradation promotes pathogenicity. *Nat Chem Biol* **10**, 371-377 (2014).

1435. Sato K*, et al.* Prostaglandin synthesis inhibitor prevents hypotension without impairing gut perfusion during normothermic cardiopulmonary bypass. *Asaio j* **48**, 503-507 (2002).

1436. Sato M, Ishizuka Y, Tanizawa H, Fukuda T, Yuizono T. [Pharmacological studies of 4-ethoxy-2-methyl-5-morpholino-3(2H)-pyridazinone (M73101). (3). General pharmacological actions (author's transl)]. *Nihon Yakurigaku Zasshi* **75**, 291-307 (1979).

1437. Sato N*, et al.* Differential induction of PPAR-gamma by luminal glutamine and iNOS by luminal arginine in the rodent postischemic small bowel. *Am J Physiol Gastrointest Liver Physiol* **290**, G616-623 (2006).

1438. Sato N*, et al.* Immune-enhancing enteral nutrients differentially modulate the early proinflammatory transcription factors mediating gut ischemia/reperfusion. *J Trauma* **58**, 455-461; discussion 461 (2005).

1439. Sato Y. [Study of Formulation Development Based on the Pharmacokinetic Properties of Functional Food Components]. *Yakugaku Zasshi* **139**, 341-347 (2019).

1440. Satoh H, Inada I, Hirata T, Maki Y. Indomethacin produces gastric antral ulcers in the refed rat. *Gastroenterology* **81**, 719-725 (1981).

1441. Satoh Y*, et al.* Mediators of nonadrenergic, noncholinergic relaxation in longitudinal muscle of the intestine of ICR mice. *J Smooth Muscle Res* **35**, 65-75 (1999).

1442. Satsu H, Awara S, Unno T, Shimizu M. Suppressive effect of nobiletin and epicatechin gallate on fructose uptake in human intestinal epithelial Caco-2 cells. *Biosci Biotechnol Biochem* **82**, 636-646 (2018).

1443. Sauls BA, Boegehold MA. Arteriolar wall PO(2) and nitric oxide release during sympathetic vasoconstriction in the rat intestine. *Am J Physiol Heart Circ Physiol* **279**, H484-491 (2000).

1444. Saura-Calixto F*, et al.* Proanthocyanidin metabolites associated with dietary fibre from in vitro colonic fermentation and proanthocyanidin metabolites in human plasma. *Mol Nutr Food Res* **54**, 939-946 (2010).

1445. Savari S*, et al.* Cysteinyl leukotriene 1 receptor influences intestinal polyp incidence in a gender-specific manner in the ApcMin/+ mouse model. *Carcinogenesis* **37**, 491-499 (2016).

1446. Scharrer E, Senn E, Wolffram S. Stimulation of mucosal uptake of selenium from selenite by some thiols at various sites of rat intestine. *Biol Trace Elem Res* **33**, 109-120 (1992).

1447. Scheithauer TP, Dallinga-Thie GM, de Vos WM, Nieuwdorp M, van Raalte DH. Causality of small and large intestinal microbiota in weight regulation and insulin resistance. *Mol Metab* **5**, 759-770 (2016).

1448. Schellekens RC, Stellaard F, Mitrovic D, Stuurman FE, Kosterink JG, Frijlink HW. Pulsatile drug delivery to ileo-colonic segments by structured incorporation of disintegrants in pH-responsive polymer coatings. *J Control Release* **132**, 91-98 (2008).

1449. Schertzer JD, Lam TKT. Peripheral and central regulation of insulin by the intestine and microbiome. *Am J Physiol Endocrinol Metab*, (2020).

1450. Schett G, Neurath MF. Resolution of chronic inflammatory disease: universal and tissue-specific concepts. *Nat Commun* **9**, 3261 (2018).

1451. Scheurlen C, Kruis W, Moser E, Paumgartner G. Accuracy of the whole body retention half-life of 75SeHCAT in the diagnosis of ileal dysfunction in patients with Crohn's disease. *Hepatogastroenterology* **35**, 136-139 (1988).

1452. Schewe M*, et al.* Secreted Phospholipases A2 Are Intestinal Stem Cell Niche Factors with Distinct Roles in Homeostasis, Inflammation, and Cancer. *Cell Stem Cell* **19**, 38-51 (2016).

1453. Schimpl G, Pesendorfer P, Steinwender G, Feierl G, Ratschek M, Höllwarth ME. Allopurinol and glutamine attenuate bacterial translocation in chronic portal hypertensive and common bile duct ligated growing rats. *Gut* **39**, 48-53 (1996).

1454. Schimpl G, Pesendorfer P, Steinwender G, Feierl G, Ratschek M, Höllwarth ME. The effect of vitamin C and vitamin E supplementation on bacterial translocation in chronic portal hypertensive and common-bile-duct-ligated rats. *Eur Surg Res* **29**, 187-194 (1997).

1455. Schirgi-Degen A, Beubler E. Significance of nitric oxide in the stimulation of intestinal fluid absorption in the rat jejunum in vivo. *Br J Pharmacol* **114**, 13-18 (1995).

1456. Schlegel L*, et al.* Bacterial dissemination and metabolic changes in rats induced by endotoxemia following intestinal E. coli overgrowth are reduced by ornithine alpha-ketoglutarate administration. *J Nutr* **130**, 2897-2902 (2000).

1457. Schmidt J*, et al.* Proinflammatory role of leukocyte-derived Egr-1 in the development of murine postoperative ileus. *Gastroenterology* **135**, 926-936, 936.e921-922 (2008).

1458. Schmidt PT, Bozkurt A, Hellström PM. Tachykinin-stimulated small bowel myoelectric pattern: sensitization by NO inhibition, reversal by neurokinin receptor blockade. *Regul Pept* **105**, 15-21 (2002).

1459. Scholz-Ahrens KE, Schrezenmeir J. Inulin and oligofructose and mineral metabolism: the evidence from animal trials. *J Nutr* **137**, 2513s-2523s (2007).

1460. Schrier BP, Lichtendonk WJ, Witjes JA. The effect of N-acetyl-L-cysteine on the viscosity of ileal neobladder mucus. *World J Urol* **20**, 64-67 (2002).

1461. Schröder B, Kaune R, Harmeyer J. Effects of calcitriol on stimulation of ion transport in pig jejunal mucosa. *J Physiol* **433**, 451-465 (1991).

1462. Schuck-Phan A*, et al.* Formula Feeding Predisposes Gut to NSAID-Induced Small Intestinal Injury. *Clin Exp Pharmacol* **6**, (2016).

1463. Schultz E, Nissinen E. Inhibition of rat liver and duodenum soluble catechol-O-methyltransferase by a tight-binding inhibitor OR-462. *Biochem Pharmacol* **38**, 3953-3956 (1989).

1464. Schulz TB, Jorde R, Burhol PG. Fasting portal vein plasma levels of gastric inhibitory polypeptide (GIP) and extractable fasting GIP in the duodenal wall in rats treated with methylprednisolone or alloxan compared with normal controls. *Scand J Gastroenterol* **17**, 487-490 (1982).

1465. Schützer KM, Haglund U, Falk A. The role of prostanoids in the feline intestinal vascular and central haemodynamic responses to i.v. infusion of live E. coli. *Acta Physiol Scand* **130**, 359-366 (1987).

1466. Schweer WP, Patience JF, Burrough ER, Kerr BJ, Gabler NK. Impact of PRRSV infection and dietary soybean meal on ileal amino acid digestibility and endogenous amino acid losses in growing pigs. *J Anim Sci* **96**, 1846-1859 (2018).

1467. Schweigert FJ, Rosival I, Rambeck WA, Gropp J. Plasma transport and tissue distribution of [14C] beta-carotene and [3H]retinol administered orally to pigs. *Int J Vitam Nutr Res* **65**, 95-100 (1995).

1468. Schweiggert RM, Carle R. Carotenoid deposition in plant and animal foods and its impact on bioavailability. *Crit Rev Food Sci Nutr* **57**, 1807-1830 (2017).

1469. Schwizer W*, et al.* Role of lipase in the regulation of upper gastrointestinal function in humans. *Am J Physiol* **273**, G612-620 (1997).

1470. Scott J, Maze M, Peters TJ. Prednisolone enhances aminopeptidase turnover in adult rat small intestine. *Biochim Biophys Acta* **719**, 464-473 (1982).

1471. Scott J, Peters TJ. Protection of epithelial function in human jejunum cultured with hydrocortisone. *Am J Physiol* **244**, G532-540 (1983).

1472. Scoville DK*, et al.* Polybrominated Diphenyl Ethers and Gut Microbiome Modulate Metabolic Syndrome-Related Aqueous Metabolites in Mice. *Drug Metab Dispos* **47**, 928-940 (2019).

1473. Seal BS*, et al.* Microbial-derived products as potential new antimicrobials. *Vet Res* **49**, 66 (2018).

1474. Secilmis MA, Kiroğlu OE, Ogulener N. Role of superoxide dismutase enzymes and ascorbate in protection of nitrergic relaxation against superoxide anions in mouse duodenum. *Acta Pharmacol Sin* **29**, 687-697 (2008).

1475. Seebach D, Gardiner J. Beta-peptidic peptidomimetics. *Acc Chem Res* **41**, 1366-1375 (2008).

1476. Seino Y, Sierra RI, Sonn YM, Jafari A, Birge SJ, Avioli LV. The duodenal 1 alpha,25-dihydroxyvitamin D3 receptor in rats with experimentally induced diabetes. *Endocrinology* **113**, 1721-1725 (1983).

1477. Sekirov I*, et al.* Antibiotic-induced perturbations of the intestinal microbiota alter host susceptibility to enteric infection. *Infect Immun* **76**, 4726-4736 (2008).

1478. Seljak KB, Berginc K, Trontelj J, Zvonar A, Kristl A, Gašperlin M. A self-microemulsifying drug delivery system to overcome intestinal resveratrol toxicity and presystemic metabolism. *J Pharm Sci* **103**, 3491-3500 (2014).

1479. Sellin JH, DeSoignie RC. Methylprednisolone increases absorptive capacity of rabbit ileum in vitro. *Am J Physiol* **245**, G562-567 (1983).

1480. Selling JA, Hogan DL, Aly A, Koss MA, Isenberg JI. Indomethacin inhibits duodenal mucosal bicarbonate secretion and endogenous prostaglandin E2 output in human subjects. *Ann Intern Med* **106**, 368-371 (1987).

1481. Sellmann C, Jin CJ, Degen C, De Bandt JP, Bergheim I. Oral Glutamine Supplementation Protects Female Mice from Nonalcoholic Steatohepatitis. *J Nutr* **145**, 2280-2286 (2015).

1482. Semba RD*, et al.* Environmental Enteric Dysfunction is Associated with Carnitine Deficiency and Altered Fatty Acid Oxidation. *EBioMedicine* **17**, 57-66 (2017).

1483. Semrad CE. Bone mass and gastrointestinal disease. *Ann N Y Acad Sci* **904**, 564-570 (2000).

1484. Sendur R, Thor P, Biernat J, Kozioł R, Pawlik WW. [Mechanism of action of neurotensin on microcirculation, metabolism and motility of the small intestine]. *Folia Med Cracov* **38**, 3-15 (1997).

1485. Senn E, Scharrer E, Wolffram S. Effects of glutathione and of cysteine on intestinal absorption of selenium from selenite. *Biol Trace Elem Res* **33**, 103-108 (1992).

1486. Sesink AL, Arts IC, Faassen-Peters M, Hollman PC. Intestinal uptake of quercetin-3-glucoside in rats involves hydrolysis by lactase phlorizin hydrolase. *J Nutr* **133**, 773-776 (2003).

1487. Seth RK*, et al.* Increased butyrate priming in the gut stalls microbiome associated-gastrointestinal inflammation and hepatic metabolic reprogramming in a mouse model of Gulf War Illness. *Toxicol Appl Pharmacol* **350**, 64-77 (2018).

1488. Shafii A, Chowdhury JR, Das KM. Absorption, enterohepatic circulation, and excretion of 5-aminosalicylic acid in rats. *Am J Gastroenterol* **77**, 297-299 (1982).

1489. Shah S, Hobbs A, Singh R, Cuevas J, Ignarro LJ, Chaudhuri G. Gastrointestinal motility during pregnancy: role of nitrergic component of NANC nerves. *Am J Physiol Regul Integr Comp Physiol* **279**, R1478-1485 (2000).

1490. Shah S, Nathan L, Singh R, Fu YS, Chaudhuri G. E2 and not P4 increases NO release from NANC nerves of the gastrointestinal tract: implications in pregnancy. *Am J Physiol Regul Integr Comp Physiol* **280**, R1546-1554 (2001).

1491. Shahbazian A, Holzer P. Differences in circular muscle contraction and peristaltic motor inhibition caused by tachykinin NK1 receptor agonists in the guinea-pig small intestine. *Neurogastroenterol Motil* **12**, 197-204 (2000).

1492. Shan R, Howlett SE, Knaus EE. Syntheses, calcium channel agonist-antagonist modulation activities, nitric oxide release, and voltage-clamp studies of 2-nitrooxyethyl 1,4-dihydro- 2,6-dimethyl-3-nitro-4-(2-trifluoromethylphenyl)pyridine-5-carboxylate enantiomers. *J Med Chem* **45**, 955-961 (2002).

1493. Shaoul R, Day AS. Nutritional regulators of intestinal inflammation. *Curr Opin Gastroenterol* **35**, 486-490 (2019).

1494. Sharma P*, et al.* Clinical, endoscopic, and histological differentiation between celiac disease and tropical sprue: A systematic review. *J Gastroenterol Hepatol* **34**, 74-83 (2019).

1495. Sharma U*, et al.* Metabolic abnormalities of gastrointestinal mucosa in celiac disease: An in vitro proton nuclear magnetic resonance spectroscopy study. *J Gastroenterol Hepatol* **30**, 1492-1498 (2015).

1496. Sharon P, Karmeli F, Rachmilewitz D. PGE2 mediates the effect of pentagastrin on intestinal adenylate cyclase and Na-K-ATPase activities. *Prostaglandins* **21 Suppl**, 81-87 (1981).

1497. Sharpe SM*, et al.* Loss of the intestinal mucus layer in the normal rat causes gut injury but not toxic mesenteric lymph nor lung injury. *Shock* **34**, 475-481 (2010).

1498. Shawcross D, Jalan R. Dispelling myths in the treatment of hepatic encephalopathy. *Lancet* **365**, 431-433 (2005).

1499. Shea-Donohue T, Goldhill JM, Montcalm-Mazzilli E, Colleton C, Piñeiro-Carrero VM, Sjogren RW. Role of sensory afferents in the myoelectric response to acute enteric inflammation in the rabbit. *Am J Physiol* **273**, G447-455 (1997).

1500. Shekhawat PS, Sonne S, Carter AL, Matern D, Ganapathy V. Enzymes involved in L-carnitine biosynthesis are expressed by small intestinal enterocytes in mice: implications for gut health. *J Crohns Colitis* **7**, e197-205 (2013).

1501. Shepherd EJ, Helliwell PA, Mace OJ, Morgan EL, Patel N, Kellett GL. Stress and glucocorticoid inhibit apical GLUT2-trafficking and intestinal glucose absorption in rat small intestine. *J Physiol* **560**, 281-290 (2004).

1502. Shi B, Song D, Xue H, Li J, Li N, Li J. Abnormal expression of the peptide transporter PepT1 in the colon of massive bowel resection rat: a potential route for colonic mucosa damage by transport of fMLP. *Dig Dis Sci* **51**, 2087-2093 (2006).

1503. Shi B, Song D, Xue H, Li N, Li J. PepT1 mediates colon damage by transporting fMLP in rats with bowel resection. *J Surg Res* **136**, 38-44 (2006).

1504. Shi J*, et al.* Protective effects of seabuckthorn pulp and seed oils against radiation-induced acute intestinal injury. *J Radiat Res* **58**, 24-32 (2017).

1505. Shi Y, Zhou J, Jiang B, Miao M. Resveratrol and inflammatory bowel disease. *Ann N Y Acad Sci* **1403**, 38-47 (2017).

1506. Shichiri M*, et al.* Radioselenium pancreozymin-secretin test as a clinical test for pancreatic exocrine function. *Am J Dig Dis* **20**, 460-468 (1975).

1507. Shiina T, Shimizu Y, Suzuki Y, Nikami H, Takewaki T. Measurement of the propelled liquid by isolated hamster ileum as a parameter to evaluate peristalsis. *Eur J Pharmacol* **517**, 120-126 (2005).

1508. Shikova LI, Kortezova NI. Participation of muscarinic acetylcholine receptors in nitric oxide pathway in cat duodenum. *Acta Physiol Pharmacol Bulg* **25**, 57-61 (2000).

1509. Shim S*, et al.* Development of a new minipig model to study radiation-induced gastrointestinal syndrome and its application in clinical research. *Radiat Res* **181**, 387-395 (2014).

1510. Shima Y, Mori M, Harano M, Tsuge H, Tanaka N, Yamazato T. Nitric oxide mediates cerulein-induced relaxation of canine sphincter of Oddi. *Dig Dis Sci* **43**, 547-553 (1998).

1511. Shima Y*, et al.* Continuous monitoring of nitric oxide release induced by cholecystokinin from the choledochal sphincter in guinea pigs. *Digestion* **61**, 135-139 (2000).

1512. Shimizu K, Nose O, Okada S. The effect of thyroid hormone on the activities of lactase in the culture of fetal rat intestine. *Cell Biol Int Rep* **14**, 1143-1148 (1990).

1513. Shimizu T, Masuo Y, Takahashi S, Nakamichi N, Kato Y. Organic cation transporter Octn1-mediated uptake of food-derived antioxidant ergothioneine into infiltrating macrophages during intestinal inflammation in mice. *Drug Metab Pharmacokinet* **30**, 231-239 (2015).

1514. Shirole RL, Shirole NL, Saraf MN. In vitro relaxant and spasmolytic effects of essential oil of Pistacia integerrima Stewart ex Brandis Galls. *J Ethnopharmacol* **168**, 61-65 (2015).

1515. Shiue SC, Huang MZ, Su TS. A transgenic approach to study argininosuccinate synthetase gene expression. *J Biomed Sci* **21**, 42 (2014).

1516. Shorrock CJ, Rees WD. Mucosal adaptation to indomethacin induced gastric damage in man--studies on morphology, blood flow, and prostaglandin E2 metabolism. *Gut* **33**, 164-169 (1992).

1517. Short SS*, et al.* Low doses of celecoxib attenuate gut barrier failure during experimental peritonitis. *Lab Invest* **93**, 1265-1275 (2013).

1518. Shotton HR, Broadbent S, Lincoln J. Prevention and partial reversal of diabetes-induced changes in enteric nerves of the rat ileum by combined treatment with alpha-lipoic acid and evening primrose oil. *Auton Neurosci* **111**, 57-65 (2004).

1519. Shoulders CC*, et al.* Abetalipoproteinemia is caused by defects of the gene encoding the 97 kDa subunit of a microsomal triglyceride transfer protein. *Hum Mol Genet* **2**, 2109-2116 (1993).

1520. Sido B, Seel C, Hochlehnert A, Breitkreutz R, Dröge W. Low intestinal glutamine level and low glutaminase activity in Crohn's disease: a rational for glutamine supplementation? *Dig Dis Sci* **51**, 2170-2179 (2006).

1521. Siener R, Petzold J, Bitterlich N, Alteheld B, Metzner C. Determinants of urolithiasis in patients with intestinal fat malabsorption. *Urology* **81**, 17-24 (2013).

1522. Silvares SG*, et al.* Histological analysis of the intestinal wall of newborn rats submitted to hypoxia and reoxygenation to evaluate the protective effect of N-Acetylcysteine. *Acta Cir Bras* **35**, e202000401 (2020).

1523. Silverstein LJ, Swanson BG, Moffett D. Procyanidin from black beans (Phaseolus vulgaris) inhibits nutrient and electrolyte absorption in isolated rat ileum and induces secretion of chloride ion. *J Nutr* **126**, 1688-1695 (1996).

1524. Simmet T, Luck W. Clotting of whole human blood induces cysteinyl-leukotriene formation. *Thromb Res* **54**, 423-433 (1989).

1525. Simon-Assmann P, Kedinger M, Haffen K. Ability of L-triiodothyronine to modulate glucocorticoid-evoked brush border enzyme activities in cultured fetal rat intestine. *Cell Biol Int Rep* **8**, 41-45 (1984).

1526. Simpson RJ, Raja KB, Peters TJ. Fe2+ uptake by mouse intestinal mucosa in vivo and by isolated intestinal brush-border membrane vesicles. *Biochim Biophys Acta* **860**, 229-235 (1986).

1527. Singh B, Khurana L, Bandyopadhyay S, Kapil R, Katare OO. Development of optimized self-nano-emulsifying drug delivery systems (SNEDDS) of carvedilol with enhanced bioavailability potential. *Drug Deliv* **18**, 599-612 (2011).

1528. Singh RK, Barrand MA. Lipid peroxidation effects of a novel iron compound, ferric maltol. A comparison with ferrous sulphate. *J Pharm Pharmacol* **42**, 276-279 (1990).

1529. Singh S, Arthur S, Sundaram U. Unique regulation of Na-glutamine cotransporter SN2/SNAT5 in rabbit intestinal crypt cells during chronic enteritis. *J Cell Mol Med* **22**, 1443-1451 (2018).

1530. Singh S, Arthur S, Talukder J, Palaniappan B, Coon S, Sundaram U. Mast cell regulation of Na-glutamine co-transporters B0AT1 in villus and SN2 in crypt cells during chronic intestinal inflammation. *BMC Gastroenterol* **15**, 47 (2015).

1531. Singh VK, Wise SY, Singh PK, Ducey EJ, Fatanmi OO, Seed TM. α-Tocopherol succinate- and AMD3100-mobilized progenitors mitigate radiation-induced gastrointestinal injury in mice. *Exp Hematol* **40**, 407-417 (2012).

1532. Singh VK*, et al.* Alpha-tocopherol succinate-mobilized progenitors improve intestinal integrity after whole body irradiation. *Int J Radiat Biol* **89**, 334-345 (2013).

1533. Sirivech S, Driskell J, Frieden E. NADH-FMN oxidoreductase activity and iron content of organs from riboflavin and iron-deficient rats. *J Nutr* **107**, 739-745 (1977).

1534. Sivarao DV, Mashimo H, Goyal RK. Pyloric sphincter dysfunction in nNOS-/- and W/Wv mutant mice: animal models of gastroparesis and duodenogastric reflux. *Gastroenterology* **135**, 1258-1266 (2008).

1535. Sjövall H, Hagman I, Abrahamsson H. Relationship between interdigestive duodenal motility and fluid transport in humans. *Am J Physiol* **259**, G348-354 (1990).

1536. Sket R*, et al.* Hypoxia and Inactivity Related Physiological Changes (Constipation, Inflammation) Are Not Reflected at the Level of Gut Metabolites and Butyrate Producing Microbial Community: The PlanHab Study. *Front Physiol* **8**, 250 (2017).

1537. Šket R*, et al.* Hypoxia and Inactivity Related Physiological Changes (Constipation, Inflammation) Are Not Reflected at the Level of Gut Metabolites and Butyrate Producing Microbial Community: The PlanHab Study. *Front Physiol* **8**, 250 (2017).

1538. Sklan D. Carotene Cleavage Activity in the corpus luteum of cattle. *Int J Vitam Nutr Res* **53**, 23-26 (1983).

1539. Sklan D, Yosefov T, Friedman A. The effects of vitamin A, beta-carotene and canthaxanthin on vitamin A metabolism and immune responses in the chick. *Int J Vitam Nutr Res* **59**, 245-250 (1989).

1540. Slivka A*, et al.* Inhibition of sphincter of Oddi function by the nitric oxide carrier S-nitroso-N-acetylcysteine in rabbits and humans. *J Clin Invest* **94**, 1792-1798 (1994).

1541. Slomiany BL, Kosmala M, Carter SR, Konturek SJ, Bilski J, Slomiany A. Intestinal release of mucin in response to HCl and taurocholate: effect of indomethacin. *Comp Biochem Physiol A Comp Physiol* **87**, 657-663 (1987).

1542. Smeaton LA, Hirst BH, Allen A, Garner A. Gastric and duodenal HCO3- transport in vivo: influence of prostaglandins. *Am J Physiol* **245**, G751-759 (1983).

1543. Smedfors B, Theodorsson E, Aly A, Johansson C. Role of VIP in local control of secretion. *J Intern Med Suppl* **732**, 133-136 (1990).

1544. Smedfors B, Theodorsson E, Johansson C. HCl-stimulated duodenal HCO3- secretion in conscious rat. Interactions among VIP, nicotinic receptor mechanisms, and prostaglandins. *Dig Dis Sci* **39**, 2134-2142 (1994).

1545. Smith AH, Waller KD. Serum beta-carotene in persons with cancer and their immediate families. *Am J Epidemiol* **133**, 661-671 (1991).

1546. Smith PL, Blumberg JB, Stoff JS, Field M. Antisecretory effects of indomethacin on rabbit ileal mucosa in vitro. *Gastroenterology* **80**, 356-365 (1981).

1547. Smith PL, Chiossone DC, McCafferty GP. Characterization of LTC4 effects on rabbit ileal mucosa in vitro. *Naunyn Schmiedebergs Arch Pharmacol* **341**, 94-100 (1990).

1548. Smith PL, Montzka DP, McCafferty GP, Wasserman MA, Fondacaro JD. Effect of sulfidopeptide leukotrienes D4 and E4 on ileal ion transport in vitro in the rat and rabbit. *Am J Physiol* **255**, G175-183 (1988).

1549. Smith SS, Penhos JC, Recant L. Hyperglucagonemia after removal of lower bowel in rats. *Am J Physiol* **239**, E346-353 (1980).

1550. Snygg J, Aneman A, Pettersson A, Fändriks L. Jejunal mucosal nitric oxide production and substrate dependency during acute mesenteric hypoperfusion in pigs. *Crit Care Med* **28**, 2563-2566 (2000).

1551. Snygg J, Casselbrant A, Pettersson A, Holm M, Fändriks L, Aneman A. Tonometric assessment of jejunal mucosal nitric oxide formation in anaesthetized pigs. *Acta Physiol Scand* **169**, 39-45 (2000).

1552. Sobko T, Reinders C, Norin E, Midtvedt T, Gustafsson LE, Lundberg JO. Gastrointestinal nitric oxide generation in germ-free and conventional rats. *Am J Physiol Gastrointest Liver Physiol* **287**, G993-997 (2004).

1553. Soeters PB, Hallemeesch MM, Bruins MJ, van Eijk HM, Deutz NE. Quantitative in vivo assessment of arginine utilization and nitric oxide production in endotoxemia. *Am J Surg* **183**, 480-488 (2002).

1554. Sofronova SI*, et al.* Endothelial nitric oxide weakens arterial contractile responses and reduces blood pressure during early postnatal development in rats. *Nitric Oxide* **55-56**, 1-9 (2016).

1555. Song MK, Adham NF. Role of prostaglandin E2 in zinc absorption in the rat. *Am J Physiol* **234**, E99-105 (1978).

1556. Song MK, Kim YY, Heng MC, Adham NF, Ament ME. Prostaglandin interacts with steroid sex hormones in the regulation of intestinal zinc transport. *Comp Biochem Physiol Comp Physiol* **101**, 477-481 (1992).

1557. Song Z, Lv J, Sheikhahmadi A, Uerlings J, Everaert N. Attenuating Effect of Zinc and Vitamin E on the Intestinal Oxidative Stress Induced by Silver Nanoparticles in Broiler Chickens. *Biol Trace Elem Res* **180**, 306-313 (2017).

1558. Sonne S, Shekhawat PS, Matern D, Ganapathy V, Ignatowicz L. Carnitine deficiency in OCTN2-/- newborn mice leads to a severe gut and immune phenotype with widespread atrophy, apoptosis and a pro-inflammatory response. *PLoS One* **7**, e47729 (2012).

1559. Sourianarayanane A, Garg G, Smith TH, Butt MI, McCullough AJ, Shen B. Risk factors of non-alcoholic fatty liver disease in patients with inflammatory bowel disease. *J Crohns Colitis* **7**, e279-285 (2013).

1560. Souza AL*, et al.* Consumption of Diet Containing Free Amino Acids Exacerbates Colitis in Mice. *Front Immunol* **8**, 1587 (2017).

1561. Spiller R. Role of motility in chronic diarrhoea. *Neurogastroenterol Motil* **18**, 1045-1055 (2006).

1562. Spiro RK, Landman ME. Meconium ileus equivalent. *Am J Gastroenterol* **69**, 302-306 (1978).

1563. Sprooten RTM*, et al.* Increased Small Intestinal Permeability during Severe Acute Exacerbations of COPD. *Respiration* **95**, 334-342 (2018).

1564. Stange EF, Preclik G, Ditschuneit H. Prostaglandin biosynthesis in gastroduodenal mucosa: methodological difficulties and their implications. *Scand J Gastroenterol Suppl* **125**, 121-127 (1986).

1565. Stange EF*, et al.* [Prostaglandin synthesis in stomach and duodenal mucosa of the human: effect of aspirin with and without antacid]. *Z Gastroenterol* **25 Suppl 3**, 162-165 (1987).

1566. Stange EF, Preclik G, Schneider A, Seiffer E, Ditschunneit H. Hormonal regulation of 3-hydroxy-3-methylglutaryl coenzyme A reductase and alkaline phosphatase in cultured intestinal mucosa. *Biochim Biophys Acta* **678**, 202-206 (1981).

1567. Stark ME, Bauer AJ, Sarr MG, Szurszewski JH. Nitric oxide mediates inhibitory nerve input in human and canine jejunum. *Gastroenterology* **104**, 398-409 (1993).

1568. Stark ME, Bauer AJ, Szurszewski JH. Effect of nitric oxide on circular muscle of the canine small intestine. *J Physiol* **444**, 743-761 (1991).

1569. Stazi AV, Trinti B. [Selenium deficiency in celiac disease: risk of autoimmune thyroid diseases]. *Minerva Med* **99**, 643-653 (2008).

1570. Stebbins CL, Smith RC, Longhurst JC. Effect of prostaglandins on bradykinin-induced visceral-cardiac reflexes. *Am J Physiol* **249**, H155-163 (1985).

1571. Stebbins CL, Symons JD, Hageman KS, Musch TI. Endogenous prostaglandins limit angiotensin-II induced regional vasoconstriction in conscious rats. *J Cardiovasc Pharmacol* **42**, 10-16 (2003).

1572. Stefano GB, Zhu W, Cadet P, Bilfinger TV, Mantione K. Morphine enhances nitric oxide release in the mammalian gastrointestinal tract via the micro(3) opiate receptor subtype: a hormonal role for endogenous morphine. *J Physiol Pharmacol* **55**, 279-288 (2004).

1573. Stein K*, et al.* A role for 12/15-lipoxygenase-derived proresolving mediators in postoperative ileus: protectin DX-regulated neutrophil extravasation. *J Leukoc Biol* **99**, 231-239 (2016).

1574. Stick JA, Arden WA, Chou CC, Parks AH, Wagner MA, Johnston CC. Effects of flunixin meglumine on jejunal blood flow, motility, and oxygen consumption in ponies. *Am J Vet Res* **49**, 1173-1178 (1988).

1575. Stiel D, Ellard KT, Hills LJ, Brooks PM. Protective effect of enprostil against aspirin-induced gastroduodenal mucosal injury in man. Comparison with cimetidine and sucralfate. *Am J Med* **81**, 54-58 (1986).

1576. Straussberg R*, et al.* Congenital intractable diarrhea of infancy in Iraqi Jews. *Clin Genet* **51**, 98-101 (1997).

1577. Strömmer L*, et al.* Early impairment of insulin secretion in rats after surgical trauma. *Eur J Endocrinol* **147**, 825-833 (2002).

1578. Strugala GJ, Overhoff H, Forth W. Bidirectional transfer and tissue accumulation of folic acid by rat intestine in vitro. *Digestion* **32**, 255-266 (1985).

1579. Strum WB. Characteristics of the transport of pteroylglutamate and amethopterin in rat jejunum. *J Pharmacol Exp Ther* **216**, 329-333 (1981).

1580. Strzałkowski AK, Godlewski MM, Hallay N, Kulasek G, Gajewski Z, Zabielski R. The effect of supplementing sow with bioactive substances on neonatal small intestinal epithelium. *J Physiol Pharmacol* **58 Suppl 3**, 115-122 (2007).

1581. Su D, May JM, Koury MJ, Asard H. Human erythrocyte membranes contain a cytochrome b561 that may be involved in extracellular ascorbate recycling. *J Biol Chem* **281**, 39852-39859 (2006).

1582. Su D*, et al.* Vitamin D Signaling through Induction of Paneth Cell Defensins Maintains Gut Microbiota and Improves Metabolic Disorders and Hepatic Steatosis in Animal Models. *Front Physiol* **7**, 498 (2016).

1583. Su HF*, et al.* Absorptive interactions of concurrent oral administration of (+)-catechin and puerarin in rats and the underlying mechanisms. *Acta Pharmacol Sin* **37**, 545-554 (2016).

1584. Su YB*, et al.* Chronic calcitriol supplementation improves the inflammatory profiles of circulating monocytes and the associated intestinal/adipose tissue alteration in a diet-induced steatohepatitis rat model. *PLoS One* **13**, e0194867 (2018).

1585. Su YC*, et al.* Effects of the nitric oxide synthase inhibitor NG-nitro-L-arginine methyl ester (L-NAME) on antropyloroduodenal motility and appetite in response to intraduodenal lipid infusion in humans. *Scand J Gastroenterol* **36**, 948-954 (2001).

1586. Subbarayan C, Lkshmanan MR, Cama HR. Metabolism and biologicalpotency of 5,-monoepoxy-beta-carotene and 5,6:5',6'-diepoxy-beta-carotene. *Biochem J* **99**, 308-311 (1966).

1587. Subramanian VS, Subramanya SB, Ghosal A, Marchant JS, Harada A, Said HM. Modulation of function of sodium-dependent vitamin C transporter 1 (SVCT1) by Rab8a in intestinal epithelial cells: studies utilizing Caco-2 cells and Rab8a knockout mice. *Dig Dis Sci* **58**, 641-649 (2013).

1588. Subratty AH, Hossany R. Does TAME induced contraction involve an endothelium dependent nitric oxide-cyclic GMP mediated pathway? *Indian J Exp Biol* **37**, 406-408 (1999).

1589. Sugamoto S, Kawauch S, Furukawa O, Mimaki TH, Takeuchi K. Role of endogenous nitric oxide and prostaglandin in duodenal bicarbonate response induced by mucosal acidification in rats. *Dig Dis Sci* **46**, 1208-1216 (2001).

1590. Sugisawa K, Komori S, Takewaki T, Ohashi H. Stimulative effect of sodium nitroprusside on peristaltic reflex in isolated guinea pig ileal segments. *Jpn J Pharmacol* **57**, 279-289 (1991).

1591. Sugita S*, et al.* Effect of the new synthetic vitamin E derivative ETS-GS on radiation enterocolitis symptoms in a rat model. *Oncol Lett* **6**, 1229-1233 (2013).

1592. Summers RW, Glenn CE, Flatt AJ, Elahmady A. Radiation and indomethacin effects on morphology, prostaglandins, and motility in dog jejunum. *Am J Physiol* **261**, G145-151 (1991).

1593. Sun X*, et al.* Effects of heat stress on the gene expression of nutrient transporters in the jejunum of broiler chickens (Gallus gallus domesticus). *Int J Biometeorol* **59**, 127-135 (2015).

1594. Sundaram U, Wisel S, Coon S. Mechanism of inhibition of proton: dipeptide co-transport during chronic enteritis in the mammalian small intestine. *Biochim Biophys Acta* **1714**, 134-140 (2005).

1595. Sundaram U, Wisel S, Fromkes JJ. Unique mechanism of inhibition of Na+-amino acid cotransport during chronic ileal inflammation. *Am J Physiol* **275**, G483-489 (1998).

1596. Suresh D, Srinivasan K. Tissue distribution & elimination of capsaicin, piperine & curcumin following oral intake in rats. *Indian J Med Res* **131**, 682-691 (2010).

1597. Sutherland LR, Church DL, Gill MJ, Kelly JK, Hwang WS, Bryant HE. Gastrointestinal function and structure in HIV-positive patients. *Cmaj* **143**, 641-646 (1990).

1598. Suzuki N, Mizuno K, Gomi Y. Role of nitric oxide in the peristalsis in the isolated guinea-pig ileum. *Eur J Pharmacol* **251**, 221-227 (1994).

1599. Suzuki R, Goda T, Takase S. Consumption of excess vitamin A, but not excess beta-carotene, causes accumulation of retinol that exceeds the binding capacity of cellular retinol-binding protein, type II in rat intestine. *J Nutr* **125**, 2074-2082 (1995).

1600. Suzuki T, Mochizuki K, Goda T. Thyroid and glucocorticoid hormones induce expression of lactase-phlorizin hydrolase gene in CDX-2/HNF-1α co-transfected IEC-6 cells. *J Nutr Sci Vitaminol (Tokyo)* **60**, 321-327 (2014).

1601. Svanes K, Halvorsen JF. Enterolith obstruction of the ileum as a complication of jejunal diverticulitis. Report of a case. *Acta Chir Scand* **141**, 816-819 (1975).

1602. Svartholm E, Arvidsson S, Fält K, Haglund U. Influence of prostanoids on gastrointestinal mucosal injury in experimental septic shock. *Apmis* **97**, 61-67 (1989).

1603. Sweeney T, Collins CB, Reilly P, Pierce KM, Ryan M, O'Doherty JV. Effect of purified β-glucans derived from Laminaria digitata, Laminaria hyperborea and Saccharomyces cerevisiae on piglet performance, selected bacterial populations, volatile fatty acids and pro-inflammatory cytokines in the gastrointestinal tract of pigs. *Br J Nutr* **108**, 1226-1234 (2012).

1604. Symons JD, Musch TI, Hageman KS, Stebbins CL. Regional blood flow responses to acute ANG II infusion: effects of nitric oxide synthase inhibition. *J Cardiovasc Pharmacol* **34**, 116-123 (1999).

1605. Syu YF, Inui A, Chen CY. A perspective on metabolic surgery from a gastroenterologist. *J Pharmacol Sci* **133**, 61-64 (2017).

1606. Szilvassy Z, Nagy I, Szilvassy J, Jakab I, Csati S, Lonovics J. Impaired nitrergic relaxation of the sphincter of Oddi of hyperlipidaemic rabbits. *Eur J Pharmacol* **301**, R17-18 (1996).

1607. Szpetnar M*, et al.* Antioxidants in patients receiving total parenteral nutrition after gastrointestinal cancer surgery. *Cell Biochem Funct* **30**, 211-216 (2012).

1608. Tabata K, Jacobson ED, Chen MH, Murphy RF, Joffe SN. Decrease in alkaline secretion during duodenal ulceration induced by mepirizole in rats. *Gastroenterology* **87**, 396-401 (1984).

1609. Tabata K, Okabe S. Effects of 16,16-dimethyl-PGE2-methyl ester on aspirin- and indomethacin-induced gastric and intestinal lesions in mini pigs. *Digestion* **26**, 61-66 (1983).

1610. Tadros T, Wobbes T, Hendriks T. Blood transfusion impairs the healing of experimental intestinal anastomoses. *Ann Surg* **215**, 276-281 (1992).

1611. Tadros T, Wobbes T, Hendriks T. Opposite effects of interleukin-2 on normal and transfusion-suppressed healing of experimental intestinal anastomoses. *Ann Surg* **218**, 800-808 (1993).

1612. Taha MO*, et al.* Role of L-arginine, a substrate of nitric oxide biosynthesis, on intestinal ischemia-reperfusion in rabbits. *Transplant Proc* **42**, 448-450 (2010).

1613. Tai A, Fujinami Y, Matsumoto K, Kawasaki D, Yamamoto I. Bioavailability of a series of novel acylated ascorbic acid derivatives, 6-O-acyl-2-O-alpha-D-glucopyranosyl-L-ascorbic acids, as an ascorbic acid supplement in rats and guinea pigs. *Biosci Biotechnol Biochem* **66**, 1628-1634 (2002).

1614. Tai A, Kawasaki D, Goto S, Gohda E, Yamamoto I. Vitamin C activity in guinea pigs of 6-O-acyl-2-O-alpha-D-glucopyranosyl-L- ascorbic acids with a branched-acyl chain. *Biosci Biotechnol Biochem* **67**, 1675-1682 (2003).

1615. Tajima S, Goda T, Takase S. Coordinated distribution patterns of three enzyme activities involved in the absorption and metabolism of beta-carotene and vitamin A along the villus-crypt axis of chick duodenum. *Life Sci* **65**, 841-848 (1999).

1616. Tajima T*, et al.* EP2 and EP4 receptors on muscularis resident macrophages mediate LPS-induced intestinal dysmotility via iNOS upregulation through cAMP/ERK signals. *Am J Physiol Gastrointest Liver Physiol* **302**, G524-534 (2012).

1617. Takada K*, et al.* Participation of nitric oxide in the mucosal injury of rat intestine induced by ischemia-reperfusion. *J Pharmacol Exp Ther* **287**, 403-407 (1998).

1618. Takahashi A*, et al.* Intestinal motility in an in vivo rat model of intestinal ischemia-reperfusion with special reference to the effects of nitric oxide on the motility changes. *J Pediatr Gastroenterol Nutr* **33**, 283-288 (2001).

1619. Takamatsu N*, et al.* Human intestinal permeability of piroxicam, propranolol, phenylalanine, and PEG 400 determined by jejunal perfusion. *Pharm Res* **14**, 1127-1132 (1997).

1620. Takase S, Suruga K, Suzuki R, Goda T. Relationship between perinatal appearance of cellular retinol-binding protein, type II and retinal reductase activity in chick liver. *Life Sci* **58**, 135-144 (1996).

1621. Takatsuka S, Kitazawa T, Morita T, Horikiri Y, Yoshino H. Enhancement of intestinal absorption of poorly absorbed hydrophilic compounds by simultaneous use of mucolytic agent and non-ionic surfactant. *Eur J Pharm Biopharm* **62**, 52-58 (2006).

1622. Takatsuka S, Morita T, Koguchi A, Horikiri Y, Yamahara H, Yoshino H. Synergistic absorption enhancement of salmon calcitonin and reversible mucosal injury by applying a mucolytic agent and a non-ionic surfactant. *Int J Pharm* **316**, 124-130 (2006).

1623. Takeda I*, et al.* Long-term outcome of living related renal transplantation in a patient with short bowel syndrome. *Tohoku J Exp Med* **221**, 113-118 (2010).

1624. Takeuchi K. [Bicarbonate secretion in the mucosal defensive mechanism of the duodenum. Acid neutralization with HCO3- in the lumen and mucus gel]. *Yakugaku Zasshi* **110**, 85-104 (1990).

1625. Takeuchi K. Prostaglandin EP receptors and their roles in mucosal protection and ulcer healing in the gastrointestinal tract. *Adv Clin Chem* **51**, 121-144 (2010).

1626. Takeuchi K, Furukawa O, Tanaka H, Nishiwaki H, Okabe S. Impairment of acid-neutralizing capacity and lesion formation in the rat duodenum during hemorrhagic shock: comparative study with indomethacin. *Jpn J Pharmacol* **44**, 163-170 (1987).

1627. Takeuchi K, Furukawa O, Tanaka H, Okabe S. A new model of duodenal ulcers induced in rats by indomethacin plus histamine. *Gastroenterology* **90**, 636-645 (1986).

1628. Takeuchi K, Hatazawa R, Tanigami M, Tanaka A, Ohno R, Yokota A. Role of endogenous nitric oxide (NO) and NO synthases in healing of indomethacin-induced intestinal ulcers in rats. *Life Sci* **80**, 329-336 (2007).

1629. Takeuchi K, Kagawa S, Mimaki H, Aoi M, Kawauchi S. COX and NOS isoforms involved in acid-induced duodenal bicarbonate secretion in rats. *Dig Dis Sci* **47**, 2116-2124 (2002).

1630. Takeuchi K, Kato S, Amagase K. Prostaglandin EP receptors involved in modulating gastrointestinal mucosal integrity. *J Pharmacol Sci* **114**, 248-261 (2010).

1631. Takeuchi K, Kita K, Hayashi S, Aihara E. Regulatory mechanism of duodenal bicarbonate secretion: Roles of endogenous prostaglandins and nitric oxide. *Pharmacol Ther* **130**, 59-70 (2011).

1632. Takeuchi K, Komatsu Y, Nakamori Y, Kotani T. A Rat Model of Ischemic Enteritis: Pathogenic Importance of Enterobacteria, iNOS/NO, and COX-2/PGE2. *Curr Pharm Des* **23**, 4048-4056 (2017).

1633. Takeuchi K, Niida H, Minami M, Okabe S. Determination of bicarbonate output using pH deflection in the rat duodenum: influences of prostaglandins and cholinergic agents. *Jpn J Pharmacol* **52**, 225-232 (1990).

1634. Takeuchi K, Niida H, Ueshima K, Okabe S. Effect of YM-14673, an analogue of thyrotropin-releasing hormone, on duodenal bicarbonate secretion in the rat. *Arch Int Pharmacodyn Ther* **314**, 133-146 (1991).

1635. Takeuchi K, Ohtsuki H, Okabe S. Mechanisms of protective activity of 16,16-dimethyl PGE2 and acetazolamide on gastric and duodenal lesions in rats. *Dig Dis Sci* **31**, 406-411 (1986).

1636. Takeuchi K, Ohuchi T, Matsumoto J, Okabe S. Regulation of gastroduodenal bicarbonate secretion by capsaicin-sensitive sensory neurons in rats. *J Clin Gastroenterol* **17 Suppl 1**, S33-39 (1993).

1637. Takeuchi K, Ohuchi T, Miyake H, Niki S, Okabe S. Effects of nitric oxide synthase inhibitors on duodenal alkaline secretion in anesthetized rats. *Eur J Pharmacol* **231**, 135-138 (1993).

1638. Takeuchi K, Ohuchi T, Miyake H, Okabe S. Stimulation by nitric oxide synthase inhibitors of gastric and duodenal HCO3- secretion in rats. *J Pharmacol Exp Ther* **266**, 1512-1519 (1993).

1639. Takeuchi K, Ohuchi T, Okabe S. Effects of nitric oxide synthase inhibitor NG-nitro-L-arginine methyl ester on duodenal alkaline secretory and ulcerogenic responses induced by mepirizole in rats. *Dig Dis Sci* **40**, 670-677 (1995).

1640. Takeuchi K, Tachibana K, Ueshima K, Matsumoto J, Okabe S. Stimulation by capsaicin of gastric alkaline secretion in anesthetized rats. *Jpn J Pharmacol* **59**, 151-157 (1992).

1641. Takeuchi K, Takehara K, Kato S, Yagi K. PACAPs stimulate duodenal bicarbonate secretion at PACAP receptors in the rat. *Am J Physiol* **272**, G646-653 (1997).

1642. Takeuchi K, Takehara K, Okabe S. Mechanisms underlying stimulation of gastroduodenal HCO3- secretion by NG-nitro-L-arginine methyl ester, an inhibitor of nitric oxide synthase, in rats. *Jpn J Pharmacol* **66**, 295-302 (1994).

1643. Takeuchi K, Yagi K, Sugamoto S, Furukawa O, Kawauchi S. Involvement of PACAP in acid-induced HCO3- response in rat duodenums. *Pharmacol Res* **38**, 475-480 (1998).

1644. Takeuchi T, Hata F, Yagasaki O. Role of cyclic AMP in prostaglandin-induced modulation of acetylcholine release from the myenteric plexus of guinea pig ileum. *Jpn J Pharmacol* **60**, 327-333 (1992).

1645. Takeuchi T*, et al.* Decrease in participation of nitric oxide in nonadrenergic, noncholinergic relaxation of rat intestine with age. *Jpn J Pharmacol* **78**, 293-302 (1998).

1646. Takeuchi T, Shimizu M, Okuda M, Yagasaki O. Contribution of external Ca2+ to the modulation by prostaglandin E2 of the release of acetylcholine from the myenteric plexus of guinea pig ileum. *Jpn J Pharmacol* **49**, 455-461 (1989).

1647. Takeuchi T, Yagasaki O. Modulation of acetylcholine release from guinea-pig Ileum myenteric plexus by arachidonic acid cascade inhibitors. *Jpn J Pharmacol* **45**, 434-437 (1987).

1648. Talukder JR, Kekuda R, Saha P, Sundaram U. Mechanism of leukotriene D4 inhibition of Na-alanine cotransport in intestinal epithelial cells. *Am J Physiol Gastrointest Liver Physiol* **295**, G1-g6 (2008).

1649. Talukder MJ, Harada E. Bovine lactoferrin protects lipopolysaccharide-induced diarrhea modulating nitric oxide and prostaglandin E2 in mice. *Can J Physiol Pharmacol* **85**, 200-208 (2007).

1650. Tan J, Applegate TJ, Liu S, Guo Y, Eicher SD. Supplemental dietary L-arginine attenuates intestinal mucosal disruption during a coccidial vaccine challenge in broiler chickens. *Br J Nutr* **112**, 1098-1109 (2014).

1651. Tanaka A, Kunikata T, Mizoguchi H, Kato S, Takeuchi K. Dual action of nitric oxide in pathogenesis of indomethacin-induced small intestinal ulceration in rats. *J Physiol Pharmacol* **50**, 405-417 (1999).

1652. Tanaka H, Takeuchi K, Okabe S, Murakami M. Pathogenesis of the earliest epithelial cell damage induced by mepirizole and cysteamine in the rat duodenum. *Jpn J Pharmacol* **51**, 509-519 (1989).

1653. Tanaka H, Ueki S, Takeuchi K, Okabe S. Effects of indomethacin on the duodenal mucosa of rats: comparative study with cysteamine. *Jpn J Pharmacol* **42**, 539-548 (1986).

1654. Tanaka M, Natsuki R. [The inhibitory effect and the mechanism of ethanol absorption by zinc complex in mouse gastrointestinal tract]. *Nihon Yakurigaku Zasshi* **111**, 327-336 (1998).

1655. Tanaka Y*, et al.* Effect of Absorption Behavior of Solubilizers on Drug Dissolution in the Gastrointestinal Tract: Evaluation Based on In Vivo Luminal Concentration-Time Profile of Cilostazol, a Poorly Soluble Drug, and Solubilizers. *J Pharm Sci* **105**, 2825-2831 (2016).

1656. Tang SQ*, et al.* N-stearoyltyrosine dipotassium ameliorates high-fat diet-induced obesity in C57BL/6 mice. *Eur J Pharm Sci* **74**, 18-26 (2015).

1657. Tang W, Xing Z, Hu W, Li C, Wang J, Wang Y. Antioxidative effects in vivo and colonization of Lactobacillus plantarum MA2 in the murine intestinal tract. *Appl Microbiol Biotechnol* **100**, 7193-7202 (2016).

1658. Tang Y, Tan SA, Iqbal A, Li J, Glover SC. STAT3 Genotypic Variant rs744166 and Increased Tyrosine Phosphorylation of STAT3 in IL-23 Responsive Innate Lymphoid Cells during Pathogenesis of Crohn's Disease. *J Immunol Res* **2019**, 9406146 (2019).

1659. Tanii H, Horie T. Uptake of barbituric acid derivatives in small intestinal brush border membrane vesicles from retinyl palmitate-treated rats. *Pharmacol Toxicol* **87**, 79-83 (2000).

1660. Taplyĭ DL, Saprykin VN. [Effect of vitamin E on the functional state of the duodenal muscles]. *Vopr Pitan*, 30-32 (1977).

1661. Tarko T, Duda-Chodak A, Soszka A. Changes in Phenolic Compounds and Antioxidant Activity of Fruit Musts and Fruit Wines during Simulated Digestion. *Molecules* **25**, (2020).

1662. Tavaf-Motamen H, Miner TJ, Starnes BW, Shea-Donohue T. Nitric oxide mediates acute lung injury by modulation of inflammation. *J Surg Res* **78**, 137-142 (1998).

1663. Taylor SD, Soudah HC, Chey WY, Scheiman JM. Duodenal acidification and secretin, but not intraduodenal fat, inhibit human gastric acid secretion via prostaglandins. *Gastroenterology* **107**, 1680-1685 (1994).

1664. Tekwe CD*, et al.* Oral administration of α-ketoglutarate enhances nitric oxide synthesis by endothelial cells and whole-body insulin sensitivity in diet-induced obese rats. *Exp Biol Med (Maywood)* **244**, 1081-1088 (2019).

1665. Ten Have GAM, Engelen M, Wolfe RR, Deutz NEP. Inhibition of jejunal protein synthesis and breakdown in Pseudomonas aeruginosa-induced sepsis pig model. *Am J Physiol Gastrointest Liver Physiol* **316**, G755-g762 (2019).

1666. Tepavcević SN, Isenović ER, Varagić VM, Milovanović SR. Sodium nitroprusside regulates the relaxation of the longitudinal muscle in the gut. *Pharmazie* **63**, 151-155 (2008).

1667. Tepperman BL, Brown JF, Whittle BJ. Nitric oxide synthase induction and intestinal epithelial cell viability in rats. *Am J Physiol* **265**, G214-218 (1993).

1668. Terada M, Hayashi S, Hayashi E. Spasmogenic effects of L-ascorbic acid on the guinea pig isolated ileum preparation. *Jpn J Pharmacol* **30**, 21-27 (1980).

1669. Theodoropoulos C, Demers C, Mirshahi A, Gascon-Barré M. 1,25-Dihydroxyvitamin D(3) downregulates the rat intestinal vitamin D(3)-25-hydroxylase CYP27A. *Am J Physiol Endocrinol Metab* **281**, E315-325 (2001).

1670. Theodorsson-Norheim E, Rosell S, Brattsand R. Dexamethasone reduces fat-induced release of neurotensin in the rat. *Acta Physiol Scand* **123**, 331-333 (1985).

1671. Thévenot J*, et al.* Enterohemorrhagic Escherichia coli infection has donor-dependent effect on human gut microbiota and may be antagonized by probiotic yeast during interaction with Peyer's patches. *Appl Microbiol Biotechnol* **99**, 9097-9110 (2015).

1672. Thimister PW*, et al.* Role of intraduodenal proteases in plasma cholecystokinin and pancreaticobiliary responses to protein and amino acids. *Gastroenterology* **110**, 567-575 (1996).

1673. Thomas FB*, et al.* Selective release of gastric inhibitory polypeptide by intraduodenal amino acid perfusion in man. *Gastroenterology* **74**, 1261-1265 (1978).

1674. Thomas S, Anup R, Susama P, Balasubramanian KA. Nitric oxide prevents intestinal mitochondrial dysfunction induced by surgical stress. *Br J Surg* **88**, 393-399 (2001).

1675. Thomas S, Prabhu R, Balasubramanian KA. Surgical manipulation of the intestine and distant organ damage-protection by oral glutamine supplementation. *Surgery* **137**, 48-55 (2005).

1676. Thompson JS, Hollingsed TC, Saxena SK. Prevention of contraction of patched intestinal defects. *Arch Surg* **123**, 428-430 (1988).

1677. Thorbøll JE, Bindslev N, Tindholdt TT, Schmidt P, Christensen P, Skadhauge E. Tachykinins mediate changes in ion transport in porcine jejunum through release of prostaglandins and neurotransmitters. *Regul Pept* **77**, 105-111 (1998).

1678. Ticho AL, Malhotra P, Dudeja PK, Gill RK, Alrefai WA. Bile Acid Receptors and Gastrointestinal Functions. *Liver Res* **3**, 31-39 (2019).

1679. Tift WL, Lloyd JK. Intestinal lymphangiectasia. Long-term results with MCT diet. *Arch Dis Child* **50**, 269-276 (1975).

1680. Tilton RG, Chang KC, LeJeune WS, Stephan CC, Brock TA, Williamson JR. Role for nitric oxide in the hyperpermeability and hemodynamic changes induced by intravenous VEGF. *Invest Ophthalmol Vis Sci* **40**, 689-696 (1999).

1681. Timmermans SJ, Jr., Johnson LM, Harrison JH, Davidson D. Estimation of the flow of microbial nitrogen to the duodenum using milk uric acid or allantoin. *J Dairy Sci* **83**, 1286-1299 (2000).

1682. Toda N, Baba H, Okamura T. Role of nitric oxide in non-adrenergic, non-cholinergic nerve-mediated relaxation in dog duodenal longitudinal muscle strips. *Jpn J Pharmacol* **53**, 281-284 (1990).

1683. Toda N, Tanobe Y, Baba H. Suppression by NG-nitro-L-arginine of relaxations induced by non-adrenergic, non-cholinergic nerve stimulation in dog duodenal longitudinal muscle. *Jpn J Pharmacol* **57**, 527-534 (1991).

1684. Todorovic D*, et al.* Effects of subchronic methionine stimulation on oxidative status and morphological changes in the rat ileum. *Gen Physiol Biophys* **38**, 535-544 (2019).

1685. Tolessa T, Gutniak M, Holst JJ, Efendic S, Hellström PM. Inhibitory effect of glucagon-like peptide-1 on small bowel motility. Fasting but not fed motility inhibited via nitric oxide independently of insulin and somatostatin. *J Clin Invest* **102**, 764-774 (1998).

1686. Tomlinson JE, Wilder BO, Young KM, Blikslager AT. Effects of flunixin meglumine or etodolac treatment on mucosal recovery of equine jejunum after ischemia. *Am J Vet Res* **65**, 761-769 (2004).

1687. Topcu I, Vatansever S, Var A, Cavus Z, Cilaker S, Sakarya M. The effect of Misoprostol, a prostaglandin E1 analog, on apoptosis in ischemia-reperfusion-induced intestinal injury. *Acta Histochem* **109**, 322-329 (2007).

1688. Torki M, Schokker D, Duijster-Lensing M, Van Krimpen MM. Effect of nutritional interventions with quercetin, oat hulls, β-glucans, lysozyme and fish oil on performance and health status related parameters of broilers chickens. *Br Poult Sci* **59**, 579-590 (2018).

1689. Tourret J, Willing BP, Dion S, MacPherson J, Denamur E, Finlay BB. Immunosuppressive Treatment Alters Secretion of Ileal Antimicrobial Peptides and Gut Microbiota, and Favors Subsequent Colonization by Uropathogenic Escherichia coli. *Transplantation* **101**, 74-82 (2017).

1690. Townsend Dt, Casey MA, Brown DR. Mediation of neurogenic ion transport by acetylcholine, prostanoids and 5-hydroxytryptamine in porcine ileum. *Eur J Pharmacol* **519**, 285-289 (2005).

1691. Trebušak T, Vrecl Fazarinc M, Salobir J, Pirman T. The Effect of Substitution of Palm Fat with Linseed Oil on the Lipid Peroxidation, Antioxidative Capacity and Intestinal Morphology in Rabbits (Oryctolagus cuniculus). *Animals (Basel)* **9**, (2019).

1692. Trevor PB, Saunders GK, Waldron DR, Leib MS. Metastatic extramedullary plasmacytoma of the colon and rectum in a dog. *J Am Vet Med Assoc* **203**, 406-409 (1993).

1693. Triadafilopoulos G, Pothoulakis C, Weiss R, Giampaolo C, Lamont JT. Comparative study of Clostridium difficile toxin A and cholera toxin in rabbit ileum. *Gastroenterology* **97**, 1186-1192 (1989).

1694. Triantafillidis JK*, et al.* A-beta-lipoproteinemia: clinical and laboratory features, therapeutic manipulations, and follow-up study of three members of a Greek family. *J Clin Gastroenterol* **26**, 207-211 (1998).

1695. Tronchini EA, Trevizan AR, Tashima CM, Pereira RV, Zanoni JN. Supplementation with 0.1% and 2% vitamin E in diabetic rats: analysis of myenteric neurons immunostained for myosin-V and nNOS in the jejunum. *Arq Gastroenterol* **49**, 284-290 (2012).

1696. Trych U, Buniowska M, Skąpska S, Starzonek S, Marszałek K. The Bioaccessibility of Antioxidants in Black Currant Puree after High Hydrostatic Pressure Treatment. *Molecules* **25**, (2020).

1697. Tsatsanidi KN, Pugaev AV, Krendal AP, Fedorko NA, Tokaev ES. [Endoscopic placement of a jejunal tube and a method of conducting enteral feeding with special mixtures]. *Vestn Khir Im I I Grek* **139**, 61-66 (1987).

1698. Tso P, Liu M, Kalogeris TJ, Thomson AB. The role of apolipoprotein A-IV in the regulation of food intake. *Annu Rev Nutr* **21**, 231-254 (2001).

1699. Tso P, Sun W, Liu M. Gastrointestinal satiety signals IV. Apolipoprotein A-IV. *Am J Physiol Gastrointest Liver Physiol* **286**, G885-890 (2004).

1700. Tsuchiya Y, Fujita R, Saitou A, Wajima N, Aizawa F, Iinuma A. [6]-gingerol induces electrogenic sodium absorption in the rat colon via the capsaicin receptor TRPV1. *J Nutr Sci Vitaminol (Tokyo)* **60**, 403-407 (2014).

1701. Tsukahara T*, et al.* Tumor necrosis factor α decreases glucagon-like peptide-2 expression by up-regulating G-protein-coupled receptor 120 in Crohn disease. *Am J Pathol* **185**, 185-196 (2015).

1702. Tsurumi K, Abe A, Fujimura H, Asai H, Nagasaka M. [General pharmacological actions of l-(m-chlorophenyl)-3-N,N-dimethylcarbamoyl-5-methoxypyrazole (PZ-177)]. *Nihon Yakurigaku Zasshi* **72**, 41-52 (1976).

1703. Tuncer FB*, et al.* Ischemic Preconditioning and Iloprost Reduces Ischemia-Reperfusion Injury in Jejunal Flaps: An Animal Model. *Plast Reconstr Surg* **144**, 124-133 (2019).

1704. Turi JL*, et al.* Duodenal cytochrome b: a novel ferrireductase in airway epithelial cells. *Am J Physiol Lung Cell Mol Physiol* **291**, L272-280 (2006).

1705. Uchida T, Nomura S, Sakuma E, Hanzawa F, Ikeda S. α-Tocopherol does not accelerate depletion of γ-tocopherol and tocotrienol or excretion of their metabolites in rats. *Lipids* **48**, 687-695 (2013).

1706. Uehara K*, et al.* The lower intestinal tract-specific induction of heme oxygenase-1 by glutamine protects against endotoxemic intestinal injury. *Crit Care Med* **33**, 381-390 (2005).

1707. Umathe SN, Kochar NI, Jain NS, Dixit PV. Gastrointestinal dysfunction in diabetic rats relates with a decline in tissue L-arginine content and consequent low levels of nitric oxide. *Nitric Oxide* **20**, 129-133 (2009).

1708. Umeda K*, et al.* Prevention of hemorrhagic shock-induced intestinal tissue injury by glutamine via heme oxygenase-1 induction. *Shock* **31**, 40-49 (2009).

1709. Undi S*, et al.* Purinergic nerves mediate the non-nitrergic relaxation of the human ileum in response to electrical field stimulation. *Brain Res Bull* **71**, 242-244 (2006).

1710. Unno T, Komori S, Ohashi H. Some evidence against the involvement of arachidonic acid in muscarinic suppression of voltage-gated calcium channel current in guinea-pig ileal smooth muscle cells. *Br J Pharmacol* **119**, 213-222 (1996).

1711. Uriarte I*, et al.* Ileal FGF15 contributes to fibrosis-associated hepatocellular carcinoma development. *Int J Cancer* **136**, 2469-2475 (2015).

1712. Uribe A. Indomethacin accelerates clearance of labeled cells and increases DNA synthesis in gastrointestinal mucosa of the rat. *Dig Dis Sci* **37**, 403-408 (1992).

1713. Uribe A, Alam M, Soderman C. Cell kinetic events in early indomethacin-induced gastrointestinal ulcerations in the rat. *Eur J Gastroenterol Hepatol* **9**, 267-273 (1997).

1714. Uribe A, Johansson C, Rubio C. Cell proliferation of the rat gastrointestinal mucosa after treatment with E2 prostaglandins and indomethacin. *Digestion* **36**, 238-245 (1987).

1715. Uribe A, Rubio C, Johansson C. Alternating proliferative capacity in the rat gastrointestinal mucosa. Effects of E2 prostaglandins and indomethacin. *Scand J Gastroenterol* **23**, 163-170 (1988).

1716. Uto-Kondo H*, et al.* S-Allyl-L-cysteine sulfoxide, a garlic odor precursor, suppresses elevation in blood ethanol concentration by accelerating ethanol metabolism and preventing ethanol absorption from gut. *Biosci Biotechnol Biochem* **82**, 724-731 (2018).

1717. Utsunomiya H*, et al.* Upregulated absorption of dietary palmitic acids with changes in intestinal transporters in non-alcoholic steatohepatitis (NASH). *J Gastroenterol* **52**, 940-954 (2017).

1718. Vadgama JV, Wu Y, Shen D, Hsia S, Block J. Effect of selenium in combination with Adriamycin or Taxol on several different cancer cells. *Anticancer Res* **20**, 1391-1414 (2000).

1719. Vahlquist A, Carlson K, Hallberg D, Rössner S. Serum carotene, vitamin A, retinol-binding protein and lipoproteins before and after jejunoileal bypass surgery. *Int J Obes* **6**, 491-497 (1982).

1720. Valentin N*, et al.* Potential mechanisms of effects of serum-derived bovine immunoglobulin/protein isolate therapy in patients with diarrhea-predominant irritable bowel syndrome. *Physiol Rep* **5**, (2017).

1721. Valentini L*, et al.* Small intestinal permeability in older adults. *Physiol Rep* **2**, e00281 (2014).

1722. Valman HB. Growth and fat absorption after resection of ileum in childhood. *J Pediatr* **88**, 41-45 (1976).

1723. van Bergenhenegouwen J*, et al.* Oral exposure to the free amino acid glycine inhibits the acute allergic response in a model of cow's milk allergy in mice. *Nutr Res* **58**, 95-105 (2018).

1724. Van Boeckel TP*, et al.* Global trends in antimicrobial use in food animals. *Proc Natl Acad Sci U S A* **112**, 5649-5654 (2015).

1725. Van Cromphaut SJ, Stockmans I, Torrekens S, Van Herck E, Carmeliet G, Bouillon R. Duodenal calcium absorption in dexamethasone-treated mice: functional and molecular aspects. *Arch Biochem Biophys* **460**, 300-305 (2007).

1726. van der Heide F. Acquired causes of intestinal malabsorption. *Best Pract Res Clin Gastroenterol* **30**, 213-224 (2016).

1727. van der Vijver RJ, van Laarhoven CJ, Lomme RM, Hendriks T. Paracetamol does not compromise early wound repair in the intestine or abdominal wall in the rat. *Anesth Analg* **115**, 1451-1456 (2012).

1728. van der Vliet A, van der Poel KI, Bast A. Intestinal smooth muscle dysfunction after intraperitoneal injection of zymosan in the rat: are oxygen radicals involved? *Gut* **33**, 336-341 (1992).

1729. Van Hecke T, Basso V, De Smet S. Lipid and Protein Oxidation during in Vitro Gastrointestinal Digestion of Pork under Helicobacter pylori Gastritis Conditions. *J Agric Food Chem* **66**, 13000-13010 (2018).

1730. van Heumen BW*, et al.* Ursodeoxycholic acid counteracts celecoxib in reduction of duodenal polyps in patients with familial adenomatous polyposis: a multicentre, randomized controlled trial. *Orphanet J Rare Dis* **8**, 118 (2013).

1731. Van Loon FP, Rabbani GH, Bukhave K, Rask-Madsen J. Indomethacin decreases jejunal fluid secretion in addition to luminal release of prostaglandin E2 in patients with acute cholera. *Gut* **33**, 643-645 (1992).

1732. Van Nassauw L, Bogers J, Van Marck E, Timmermans JP. Role of reactive nitrogen species in neuronal cell damage during intestinal schistosomiasis. *Cell Tissue Res* **303**, 329-336 (2001).

1733. van Vliet T, van Schaik F, Schreurs WH, van den Berg H. In vitro measurement of beta-carotene cleavage activity: methodological considerations and the effect of other carotenoids on beta-carotene cleavage. *Int J Vitam Nutr Res* **66**, 77-85 (1996).

1734. Van Vliet T, Van Schaik F, Van Schoonhoven J, Schrijver J. Determination of several retinoids, carotenoids and E vitamers by high-performance liquid chromatography. Application to plasma and tissues of rats fed a diet rich in either beta-carotene or canthaxanthin. *J Chromatogr* **553**, 179-186 (1991).

1735. Vanner S. Corelease of neuropeptides from capsaicin-sensitive afferents dilates submucosal arterioles in guinea pig ileum. *Am J Physiol* **267**, G650-655 (1994).

1736. Vardi N*, et al.* Potent protective effect of apricot and beta-carotene on methotrexate-induced intestinal oxidative damage in rats. *Food Chem Toxicol* **46**, 3015-3022 (2008).

1737. Varma MV, Panchagnula R. Enhanced oral paclitaxel absorption with vitamin E-TPGS: effect on solubility and permeability in vitro, in situ and in vivo. *Eur J Pharm Sci* **25**, 445-453 (2005).

1738. Vary TC, Murphy JM. Role of extra-splanchnic organs in the metabolic response to sepsis: effect of insulin. *Circ Shock* **29**, 41-57 (1989).

1739. Vatn MH. The effect of duodenal juice on the intrinsic factor (IF) activity in gastric juice. *Scand J Gastroenterol* **10**, 49-59 (1975).

1740. Veit AP, Zanoni JN. Age-related changes in myosin-V myenteric neurons, CGRP and VIP immunoreactivity in the ileum of rats supplemented with ascorbic acid. *Histol Histopathol* **27**, 123-132 (2012).

1741. Vella A*, et al.* Effect of glucagon-like peptide-1(7-36)-amide on initial splanchnic glucose uptake and insulin action in humans with type 1 diabetes. *Diabetes* **50**, 565-572 (2001).

1742. Verbeke L*, et al.* The FXR agonist obeticholic acid prevents gut barrier dysfunction and bacterial translocation in cholestatic rats. *Am J Pathol* **185**, 409-419 (2015).

1743. Vicentini GE*, et al.* Does l-glutamine-supplemented diet extenuate NO-mediated damage on myenteric plexus of Walker 256 tumor-bearing rats? *Food Res Int* **101**, 24-34 (2017).

1744. Vici G, Camilletti D, Polzonetti V. Possible Role of Vitamin D in Celiac Disease Onset. *Nutrients* **12**, (2020).

1745. Vieira EL*, et al.* Oral administration of sodium butyrate attenuates inflammation and mucosal lesion in experimental acute ulcerative colitis. *J Nutr Biochem* **23**, 430-436 (2012).

1746. Vignoli AL, Srivastava RC, Stammati A, Turco L, Tanori M, Zucco F. Nitric oxide production in Caco-2 cells exposed to different inducers, inhibitors and natural toxins. *Toxicol In Vitro* **15**, 289-295 (2001).

1747. Vignozzi L*, et al.* Cardiopulmonary protective effects of the selective FXR agonist obeticholic acid in the rat model of monocrotaline-induced pulmonary hypertension. *J Steroid Biochem Mol Biol* **165**, 277-292 (2017).

1748. Viñuales C, Gascón S, Barranquero C, Osada J, Rodríguez-Yoldi MJ. Inhibitory effect of IL-1β on galactose intestinal absorption in rabbits. *Cell Physiol Biochem* **30**, 173-186 (2012).

1749. Virden WS, Lilburn MS, Thaxton JP, Corzo A, Hoehler D, Kidd MT. The effect of corticosterone-induced stress on amino acid digestibility in Ross broilers. *Poult Sci* **86**, 338-342 (2007).

1750. Vlasov TD, Smirnov DA, Nutfullina GM. [Adaptation of the rat small intestine to ischemia]. *Ross Fiziol Zh Im I M Sechenova* **87**, 118-124 (2001).

1751. Voelker CA, Miller MJ, Zhang XJ, Eloby-Childress S, Clark DA, Pierce MR. Perinatal nitric oxide synthase inhibition retards neonatal growth by inducing hypertrophic pyloric stenosis in rats. *Pediatr Res* **38**, 768-774 (1995).

1752. Voigt MR, DeLario GT. Perspectives on abdominal organ preservation solutions: a comparative literature review. *Prog Transplant* **23**, 383-391 (2013).

1753. von Ritter C, Grisham MB, Hollwarth M, Inauen W, Granger DN. Neutrophil-derived oxidants mediate formyl-methionyl-leucyl-phenylalanine-induced increases in mucosal permeability in rats. *Gastroenterology* **97**, 778-780 (1989).

1754. Vongtau HO*, et al.* Pharmacological effects of the aqueous extract of Neorautanenia mitis in rodents. *J Ethnopharmacol* **72**, 207-214 (2000).

1755. Vukosavljevic N, Jaron D, Barbee KA, Buerk DG. Quantifying the L-arginine paradox in vivo. *Microvasc Res* **71**, 48-54 (2006).

1756. Wali FA. Possible involvement of substance P in the contraction produced by periarterial nerve stimulation in the rat ileum. *J Auton Pharmacol* **5**, 143-148 (1985).

1757. Walker J, Dieleman L, Mah D, Park K, Meddings J, Vethanayagam D. High prevalence of abnormal gastrointestinal permeability in moderate-severe asthma. *Clin Invest Med* **37**, E53-57 (2014).

1758. Walker J, Jijon HB, Diaz H, Salehi P, Churchill T, Madsen KL. 5-aminoimidazole-4-carboxamide riboside (AICAR) enhances GLUT2-dependent jejunal glucose transport: a possible role for AMPK. *Biochem J* **385**, 485-491 (2005).

1759. Walker KF, Chappell LC, Hague WM, Middleton P, Thornton JG. Pharmacological interventions for treating intrahepatic cholestasis of pregnancy. *Cochrane Database Syst Rev* **7**, Cd000493 (2020).

1760. Walker R, Wilson KA. Prostaglandins and the contractile action of bradykinin on the longitudinal muscle of rat isolated ileum. *Br J Pharmacol* **67**, 527-533 (1979).

1761. Walker S, Rühl U. [4199 biopsies from the endoscopic normal lower duodenum]. *Z Gastroenterol* **41**, 69-74 (2003).

1762. Wallace JL, MacNaughton WK. Gastrointestinal damage induced by platelet-activating factor: role of leukotrienes. *Eur J Pharmacol* **151**, 43-50 (1988).

1763. Walton MC, McGhie TK, Reynolds GW, Hendriks WH. The flavonol quercetin-3-glucoside inhibits cyanidin-3-glucoside absorption in vitro. *J Agric Food Chem* **54**, 4913-4920 (2006).

1764. Wan D, Liu X, Li G. The effects of montelukast on eosinophilic gastroenteritis in a mouse model. *Immunopharmacol Immunotoxicol* **35**, 292-295 (2013).

1765. Wang AL*, et al.* Glutamine ameliorates intestinal ischemia-reperfusion Injury in rats by activating the Nrf2/Are signaling pathway. *Int J Clin Exp Pathol* **8**, 7896-7904 (2015).

1766. Wang CC*, et al.* Sodium butyrate enhances intestinal integrity, inhibits mast cell activation, inflammatory mediator production and JNK signaling pathway in weaned pigs. *Innate Immun* **24**, 40-46 (2018).

1767. Wang F*, et al.* Apolipoprotein A-IV improves glucose homeostasis by enhancing insulin secretion. *Proc Natl Acad Sci U S A* **109**, 9641-9646 (2012).

1768. Wang F, Pearson KJ, Davidson WS, Tso P. Specific sequences in N termini of apolipoprotein A-IV modulate its anorectic effect. *Physiol Behav* **120**, 136-142 (2013).

1769. Wang GD*, et al.* Inhibitory neuromuscular transmission mediated by the P2Y1 purinergic receptor in guinea pig small intestine. *Am J Physiol Gastrointest Liver Physiol* **292**, G1483-1489 (2007).

1770. Wang H*, et al.* Human Na(+)-dependent vitamin C transporter 1 (hSVCT1): primary structure, functional characteristics and evidence for a non-functional splice variant. *Biochim Biophys Acta* **1461**, 1-9 (1999).

1771. Wang H*, et al.* RXRα inhibits the NRF2-ARE signaling pathway through a direct interaction with the Neh7 domain of NRF2. *Cancer Res* **73**, 3097-3108 (2013).

1772. Wang H, Pan JK, Sun M, Zhou Z, Gao H. [Effects of glutamine on alpha-sarcomeric actin and its mRNA expression of myocardium in rats with endotoxemia]. *Zhonghua Er Ke Za Zhi* **43**, 925-929 (2005).

1773. Wang J*, et al.* Protective Effect of N-Acetylcysteine against Oxidative Stress Induced by Zearalenone via Mitochondrial Apoptosis Pathway in SIEC02 Cells. *Toxins (Basel)* **10**, (2018).

1774. Wang JY, Johnson LR. Gastric and duodenal mucosal ornithine decarboxylase and damage after corticosterone. *Am J Physiol* **258**, G942-950 (1990).

1775. Wang S*, et al.* MKK4 from Litopenaeus vannamei is a regulator of p38 MAPK kinase and involved in anti-bacterial response. *Dev Comp Immunol* **78**, 61-70 (2018).

1776. Wang W*, et al.* Glycine is a nutritionally essential amino acid for maximal growth of milk-fed young pigs. *Amino Acids* **46**, 2037-2045 (2014).

1777. Wang W, Li Z, Han Q, Guo Y, Zhang B, D'Inca R. Dietary live yeast and mannan-oligosaccharide supplementation attenuate intestinal inflammation and barrier dysfunction induced by Escherichia coli in broilers. *Br J Nutr* **116**, 1878-1888 (2016).

1778. Wang W*, et al.* Glycine stimulates protein synthesis and inhibits oxidative stress in pig small intestinal epithelial cells. *J Nutr* **144**, 1540-1548 (2014).

1779. Wang X, Sun Z, Börjesson A, Andersson R. Inhibition of platelet-activating factor, intercellular adhesion molecule 1 and platelet endothelial cell adhesion molecule 1 reduces experimental pancreatitis-associated gut endothelial barrier dysfunction. *Br J Surg* **86**, 411-416 (1999).

1780. Wang X*, et al.* Lentinan modulates intestinal microbiota and enhances barrier integrity in a piglet model challenged with lipopolysaccharide. *Food Funct* **10**, 479-489 (2019).

1781. Wang XB, Osugi T, Uchida S. Muscarinic receptors stimulate Ca2+ influx via phospholipase A2 pathway in ileal smooth muscles. *Biochem Biophys Res Commun* **193**, 483-489 (1993).

1782. Wang XD, Krinsky NI, Benotti PN, Russell RM. Biosynthesis of 9-cis-retinoic acid from 9-cis-beta-carotene in human intestinal mucosa in vitro. *Arch Biochem Biophys* **313**, 150-155 (1994).

1783. Wang XD*, et al.* Intestinal uptake and lymphatic absorption of beta-carotene in ferrets: a model for human beta-carotene metabolism. *Am J Physiol* **263**, G480-486 (1992).

1784. Wang XD, Marini RP, Hebuterne X, Fox JG, Krinsky NI, Russell RM. Vitamin E enhances the lymphatic transport of beta-carotene and its conversion to vitamin A in the ferret. *Gastroenterology* **108**, 719-726 (1995).

1785. Wang XJ, Hayes JD, Henderson CJ, Wolf CR. Identification of retinoic acid as an inhibitor of transcription factor Nrf2 through activation of retinoic acid receptor alpha. *Proc Natl Acad Sci U S A* **104**, 19589-19594 (2007).

1786. Wang XL, Wang YY, Fang Q, Xue YQ, Shen JL. [In vitro killing of adult Trichinella spiralis by exogenous nitric oxide]. *Zhongguo Ji Sheng Chong Xue Yu Ji Sheng Chong Bing Za Zhi* **30**, 374-377 (2012).

1787. Wang Y, Aun R, Tse FL. Absorption of D-glucose in the rat studied using in situ intestinal perfusion: a permeability-index approach. *Pharm Res* **14**, 1563-1567 (1997).

1788. Wang Y, Liang B, Watson RR. The effect of alcohol consumption on nutritional status during murine AIDS. *Alcohol* **11**, 273-278 (1994).

1789. Wang Y, Mackenzie B, Tsukaguchi H, Weremowicz S, Morton CC, Hediger MA. Human vitamin C (L-ascorbic acid) transporter SVCT1. *Biochem Biophys Res Commun* **267**, 488-494 (2000).

1790. Wang Y, Zhang ZZ, Chen SQ, Zou ZD, Tu XH, Wang L. [Protective effect of N-acetylcysteine on the intestinal barrier dysfunction after radiation injury in rats]. *Zhonghua Wei Chang Wai Ke Za Zhi* **13**, 219-222 (2010).

1791. Wang YX, Gavras I, Lammek B, Bresnahan M, Gavras H. Effects of bradykinin and prostaglandin inhibition on systemic and regional hemodynamics in conscious normotensive rats. *J Hypertens* **9**, 805-812 (1991).

1792. Wang ZT, Du Q, Xu GJ, Wang RJ, Fu DZ, Ng TB. Investigations on the protective action of Condonopsis pilosula (Dangshen) extract on experimentally-induced gastric ulcer in rats. *Gen Pharmacol* **28**, 469-473 (1997).

1793. Ward JBJ*, et al.* Ursodeoxycholic acid and lithocholic acid exert anti-inflammatory actions in the colon. *Am J Physiol Gastrointest Liver Physiol* **312**, G550-g558 (2017).

1794. Ward MG*, et al.* Prevalence and Risk Factors for Functional Vitamin B12 Deficiency in Patients with Crohn's Disease. *Inflamm Bowel Dis* **21**, 2839-2847 (2015).

1795. Ward NC, Croft KD, Puddey IB, Hodgson JM. Supplementation with grape seed polyphenols results in increased urinary excretion of 3-hydroxyphenylpropionic Acid, an important metabolite of proanthocyanidins in humans. *J Agric Food Chem* **52**, 5545-5549 (2004).

1796. Ward SJ, Mastriani D, Casiano F, Arnold R. Pravadoline: profile in isolated tissue preparations. *J Pharmacol Exp Ther* **255**, 1230-1239 (1990).

1797. Ward SM, McKeen ES, Sanders KM. Role of nitric oxide in non-adrenergic, non-cholinergic inhibitory junction potentials in canine ileocolonic sphincter. *Br J Pharmacol* **105**, 776-782 (1992).

1798. Watabe M, Isogai Y, Numazawa S, Yoshida T. Role of c-Myc in nitric oxide-mediated suppression of cytochrome P450 3A4. *Life Sci* **74**, 99-108 (2003).

1799. Watanabe J*, et al.* Novel anti-inflammatory functions for endothelial and myeloid cyclooxygenase-2 in a new mouse model of Crohn's disease. *Am J Physiol Gastrointest Liver Physiol* **298**, G842-850 (2010).

1800. Watanabe T, Watanabe-Kominato K, Takahashi Y, Kojima M, Watanabe R. Adipose Tissue-Derived Omentin-1 Function and Regulation. *Compr Physiol* **7**, 765-781 (2017).

1801. Waterman SA, Costa M. The role of enteric inhibitory motoneurons in peristalsis in the isolated guinea-pig small intestine. *J Physiol* **477 ( Pt 3)**, 459-468 (1994).

1802. Waters WR, Reinhardt TA, Harp JA. Oral administration of putrescine inhibits Cryptosporidium parvum infection of neonatal C57BL-6 mice and is independent of nitric oxide synthesis. *J Parasitol* **83**, 746-750 (1997).

1803. Watne AL, Lai HY, Carrier J, Coppula W. The diagnosis and surgical treatment of patients with Gardner's syndrome. *Surgery* **82**, 327-333 (1977).

1804. Wei G, Yi S, Yong D, Shaozhuang L, Guangyong Z, Sanyuan H. miR-320 mediates diabetes amelioration after duodenal-jejunal bypass via targeting adipoR1. *Surg Obes Relat Dis* **14**, 960-971 (2018).

1805. Wei JW, Wei MM. Characterization of some biochemical properties of bradykinin-induced cAMP and cGMP formation in guinea pig ileum. *Chin J Physiol* **35**, 303-316 (1992).

1806. Weiss GA, Hennet T. The role of milk sialyllactose in intestinal bacterial colonization. *Adv Nutr* **3**, 483s-488s (2012).

1807. Weissenborn U, Maedge S, Buettner D, Sewing KF. Indometacin-induced gastrointestinal lesions in relation to tissue concentration, food intake and bacterial invasion in the rat. *Pharmacology* **30**, 32-39 (1985).

1808. Wellman ML, Hoffmann WE, Dorner JL, Mock RE. Comparison of the steroid-induced, intestinal, and hepatic isoenzymes of alkaline phosphatase in the dog. *Am J Vet Res* **43**, 1204-1207 (1982).

1809. Welton AF*, et al.* Ro 22-3747: a new antiallergic agent for the treatment of immediate hypersensitivity diseases. *J Pharmacol Exp Ther* **228**, 57-64 (1984).

1810. Werner B, Denzer U, Mitschke H, Brassow F. [Ascorbic acid and cancer of the duodenum. An experimental study (author's transl)]. *Langenbecks Arch Chir* **354**, 101-109 (1981).

1811. Werner B, Hrynyschyn K, Schäfer H. [Vitamin C in a long-term trial is without effect on experimental carcinogenesis]. *Langenbecks Arch Chir* **363**, 185-193 (1985).

1812. Wettergren A, Kirkegaard P. [Pouchitis: acute inflammation in the pelvic ileal reservoir. Diagnostic criteria, frequency, possible etiological factors and treatment]. *Ugeskr Laeger* **155**, 2451-2454 (1993).

1813. Weyant MJ, Carothers AM, Dannenberg AJ, Bertagnolli MM. (+)-Catechin inhibits intestinal tumor formation and suppresses focal adhesion kinase activation in the min/+ mouse. *Cancer Res* **61**, 118-125 (2001).

1814. Wheal AJ, Alexander SP, Randall MD. Vasorelaxation to N-oleoylethanolamine in rat isolated arteries: mechanisms of action and modulation via cyclooxygenase activity. *Br J Pharmacol* **160**, 701-711 (2010).

1815. Whelan G, Wood B. The metabolic consequences of jejunoileal bypass for obesity. *Aust N Z J Surg* **50**, 520-524 (1980).

1816. White J, Blower P, Canfield P. Effect of luminal perfusion rate on duodenal alkalinization in the rat in vivo and in vitro. *Dig Dis Sci* **34**, 1349-1354 (1989).

1817. Whitehouse LW, Wong LT, Solomonraj G, Paul CJ, Thomas BH. N-acetylcysteine-induced inhibition of gastric emptying: a mechanism affording protection to mice from the hepatotoxicity of concomitantly administered acetaminophen. *Toxicology* **19**, 113-125 (1981).

1818. Wijnands KA*, et al.* Arginase-1 deficiency regulates arginine concentrations and NOS2-mediated NO production during endotoxemia. *PLoS One* **9**, e86135 (2014).

1819. Wijnands KA*, et al.* Citrulline Supplementation Improves Organ Perfusion and Arginine Availability under Conditions with Enhanced Arginase Activity. *Nutrients* **7**, 5217-5238 (2015).

1820. Wiklund CU, Wiklund NP, Gustafsson LE. Modulation of neuroeffector transmission by endogenous nitric oxide: a role for acetylcholine receptor-activated nitric oxide formation, as indicated by measurements of nitric oxide/nitrite release. *Eur J Pharmacol* **240**, 235-242 (1993).

1821. Wilhelm F, Norman AW. Influence of triamcinolone, estradiol-17 beta and testosterone on 1,25-dihydroxyvitamin D3 binding performances to its chick intestinal receptor. *J Steroid Biochem* **23**, 913-918 (1985).

1822. Williams CN, Sidorov JJ. Steatorrhea in patients with liver disease. *Can Med Assoc J* **105**, 1143-1146 passim (1971).

1823. Williams EA, Coxhead JM, Mathers JC. Anti-cancer effects of butyrate: use of micro-array technology to investigate mechanisms. *Proc Nutr Soc* **62**, 107-115 (2003).

1824. Williams SJ, Parsons ME. Nitric oxide, an enteric nonadrenergic-noncholinergic relaxant transmitter: evidence using phosphodiesterase V and nitric oxide synthase inhibition. *Br J Pharmacol* **116**, 1789-1796 (1995).

1825. Wilson DE, El-Hindi E, Tao P, Poppe L. Effects of indomethacin on intestinal secretion, prostaglandin E and cyclic AMP: evidence against a role for prostaglandins in cholera toxin-induced secretion. *Prostaglandins* **10**, 581-587 (1975).

1826. Wilson FA, Treanor LL. Studies of relationship among bile-acid uptake, Na+, K+-ATPase, and Na+ gradient in isolated cells from rat ileum. *Gastroenterology* **81**, 54-60 (1981).

1827. Wilson JX. Regulation of vitamin C transport. *Annu Rev Nutr* **25**, 105-125 (2005).

1828. Wimberly AL, Forsyth CB, Khan MW, Pemberton A, Khazaie K, Keshavarzian A. Ethanol-induced mast cell-mediated inflammation leads to increased susceptibility of intestinal tumorigenesis in the APC Δ468 min mouse model of colon cancer. *Alcohol Clin Exp Res* **37 Suppl 1**, E199-208 (2013).

1829. Winne D, Markgraf I. The longitudinal intraluminal concentration gradient in the perfused rat jejunum and the appropriate mean concentration for calculation of the absorption rate. *Naunyn Schmiedebergs Arch Pharmacol* **309**, 271-279 (1979).

1830. Winne D, Verheyen W. Diffusion coefficient in native mucus gel of rat small intestine. *J Pharm Pharmacol* **42**, 517-519 (1990).

1831. Wischmeyer PE, Kahana M, Wolfson R, Ren H, Musch MM, Chang EB. Glutamine reduces cytokine release, organ damage, and mortality in a rat model of endotoxemia. *Shock* **16**, 398-402 (2001).

1832. Witkowski S*, et al.* The cleavage of vitamin E galactoside in the rat tissue homogenates. *Farmaco* **59**, 669-671 (2004).

1833. Wlodarska M*, et al.* Indoleacrylic Acid Produced by Commensal Peptostreptococcus Species Suppresses Inflammation. *Cell Host Microbe* **22**, 25-37.e26 (2017).

1834. Wolf C, Ritter U, Zschocke R, Weinert W. [Investigation of the choleretic action of febuprol on healthy test subjects (author's transl)]. *MMW Munch Med Wochenschr* **118**, 1285-1288 (1976).

1835. Wolf NM, Mueller K, Hirche F, Most E, Pallauf J, Mueller AS. Study of molecular targets influencing homocysteine and cholesterol metabolism in growing rats by manipulation of dietary selenium and methionine concentrations. *Br J Nutr* **104**, 520-532 (2010).

1836. Wolffram S, Grenacher B, Scharrer E. Transport of selenate and sulphate across the intestinal brush-border membrane of pig jejunum by two common mechanism. *Q J Exp Physiol* **73**, 103-111 (1988).

1837. Woolverton CJ, White JJ, Jr., Sartor RB. Eicosanoid regulation of acute intestinal vascular permeability induced by intravenous peptidoglycan-polysaccharide polymers. *Agents Actions* **26**, 301-309 (1989).

1838. Wormmeester L*, et al.* Quantitative contribution of NHE2 and NHE3 to rabbit ileal brush-border Na+/H+ exchange. *Am J Physiol* **274**, C1261-1272 (1998).

1839. Wren SN, Donovan MG, Selmin OI, Doetschman TC, Romagnolo DF. A Villin-Driven Fxr Transgene Modulates Enterohepatic Bile Acid Homeostasis and Response to an n-6-Enriched High-Fat Diet. *Int J Mol Sci* **21**, (2020).

1840. Wu G. Functional amino acids in growth, reproduction, and health. *Adv Nutr* **1**, 31-37 (2010).

1841. Wu G. Dietary requirements of synthesizable amino acids by animals: a paradigm shift in protein nutrition. *J Anim Sci Biotechnol* **5**, 34 (2014).

1842. Wu G, Flynn NE, Knabe DA. Enhanced intestinal synthesis of polyamines from proline in cortisol-treated piglets. *Am J Physiol Endocrinol Metab* **279**, E395-402 (2000).

1843. Wu G, Morris SM, Jr. Arginine metabolism: nitric oxide and beyond. *Biochem J* **336 ( Pt 1)**, 1-17 (1998).

1844. Wu G*, et al.* Dietary requirements of "nutritionally non-essential amino acids" by animals and humans. *Amino Acids* **44**, 1107-1113 (2013).

1845. Wu M*, et al.* Therapeutic effects of glutamic acid in piglets challenged with deoxynivalenol. *PLoS One* **9**, e100591 (2014).

1846. Wu P*, et al.* Liver Injury Impaired 25-Hydroxylation of Vitamin D Suppresses Intestinal Paneth Cell defensins, leading to Gut Dysbiosis and Liver Fibrogenesis. *Am J Physiol Gastrointest Liver Physiol*, (2020).

1847. Wu X, Pittman Iii HE, Hager T, Hager A, Howard L, Prior RL. Phenolic acids in black raspberry and in the gastrointestinal tract of pigs following ingestion of black raspberry. *Mol Nutr Food Res* **53 Suppl 1**, S76-84 (2009).

1848. Wu Y*, et al.* Influence of Butyrate Loaded Clinoptilolite Dietary Supplementation on Growth Performance, Development of Intestine and Antioxidant Capacity in Broiler Chickens. *PLoS One* **11**, e0154410 (2016).

1849. Wulff-Pérez M, Barrajón-Catalán E, Micol V, Martín-Rodríguez A, de Vicente J, Gálvez-Ruíz MJ. In vitro duodenal lipolysis of lipid-based drug delivery systems studied by HPLC-UV and HPLC-MS. *Int J Pharm* **465**, 396-404 (2014).

1850. Wusteman M, Hayes A, Stirling D, Elia M. Changes in protein distribution in the rat during prolonged "systemic injury". *J Surg Res* **56**, 331-337 (1994).

1851. Wyss A. Carotene oxygenases: a new family of double bond cleavage enzymes. *J Nutr* **134**, 246s-250s (2004).

1852. Xiao D*, et al.* Effects of dietary administering chitosan on growth performance, jejunal morphology, jejunal mucosal sIgA, occludin, claudin-1 and TLR4 expression in weaned piglets challenged by enterotoxigenic Escherichia coli. *Int Immunopharmacol* **17**, 670-676 (2013).

1853. Xiao H*, et al.* N-Acetyl-L-cysteine Protects the Enterocyte against Oxidative Damage by Modulation of Mitochondrial Function. *Mediators Inflamm* **2016**, 8364279 (2016).

1854. Xiao J, Capanoglu E, Jassbi AR, Miron A. Advance on the Flavonoid C-glycosides and Health Benefits. *Crit Rev Food Sci Nutr* **56 Suppl 1**, S29-45 (2016).

1855. Xiao L*, et al.* Angtensin II elicits a cAMP-dependent intestinal anion secretion by stimulating PGE2 release through AT1 subtype receptors in rat ileum. *Biochem Biophys Res Commun* **494**, 207-212 (2017).

1856. Xiao Y, Cui J, Shi YH, Sun J, Wang ZP, Le GW. Effects of duodenal redox status on calcium absorption and related genes expression in high-fat diet-fed mice. *Nutrition* **26**, 1188-1194 (2010).

1857. Xie J*, et al.* Ganoderma lucidum polysaccharide improves rat DSS-induced colitis by altering cecal microbiota and gene expression of colonic epithelial cells. *Food Nutr Res* **63**, (2019).

1858. Xiong Y*, et al.* Effect of chiropractic manipulation on disrupted epithelium barrier and its mechanism of specialized pro-resolving mediators in a spleen-deficiency murine model. *J Tradit Chin Med* **39**, 678-684 (2019).

1859. Xu F, Pandya JK, Chung C, McClements DJ, Kinchla AJ. Emulsions as delivery systems for gamma and delta tocotrienols: Formation, properties and simulated gastrointestinal fate. *Food Res Int* **105**, 570-579 (2018).

1860. Xu J, Soliman GM, Barralet J, Cerruti M. Mollusk glue inspired mucoadhesives for biomedical applications. *Langmuir* **28**, 14010-14017 (2012).

1861. Xu R*, et al.* Characterization of the intestinal absorption of morroniside from Cornus officinalis Sieb. et Zucc via a Caco-2 cell monolayer model. *PLoS One* **15**, e0227844 (2020).

1862. Xu X*, et al.* Glycine Relieves Intestinal Injury by Maintaining mTOR Signaling and Suppressing AMPK, TLR4, and NOD Signaling in Weaned Piglets after Lipopolysaccharide Challenge. *Int J Mol Sci* **19**, (2018).

1863. Yagasaki O, Funaki H, Yanagiya I. Contribution of endogenous prostaglandins to excitation of the myenteric plexus of guinea-pig ileum: are adrenergic factors involved? *Eur J Pharmacol* **103**, 1-8 (1984).

1864. Yağmurdur MC, Ozdemir A, Ozenç A, Kilinç K. The effects of alpha - tocopherol and verapamil on mucosal functions after gut ischemia / reperfusion. *Turk J Gastroenterol* **14**, 26-32 (2003).

1865. Yalcin D*, et al.* Intraluminal fluid infusion in a rat jejunum ischemia/reperfusion model is associated with improved tissue perfusion and less mucosal damage. *J Plast Reconstr Aesthet Surg* **73**, 590-597 (2020).

1866. Yamada C, Clark AJ, Swendseid ME. Actinomycin D effect on amino acid absorption from rat jejunal loops. *Science* **158**, 129-130 (1967).

1867. Yamaguchi N, Yamamoto T, Suruga K, Takase S. Developmental changes in gene expressions of beta-carotene cleavage enzyme and retinoic acid synthesizing enzymes in the chick duodenum. *Comp Biochem Physiol A Mol Integr Physiol* **148**, 690-697 (2007).

1868. Yamaji M*, et al.* A possible role of neurotensin in NANC relaxation of longitudinal muscle of the jejunum and ileum of Wistar rats. *Br J Pharmacol* **137**, 629-636 (2002).

1869. Yamamoto F, Sasaki S, Maeda M. Positron labeled antioxidants: synthesis and tissue biodistribution of 6-deoxy-6-[18F]fluoro-L-ascorbic acid. *Int J Rad Appl Instrum A* **43**, 633-639 (1992).

1870. Yamamoto I, Suga S, Mitoh Y, Tanaka M, Muto N. Antiscorbutic activity of L-ascorbic acid 2-glucoside and its availability as a vitamin C supplement in normal rats and guinea pigs. *J Pharmacobiodyn* **13**, 688-695 (1990).

1871. Yamamoto T*, et al.* Pretreatment with ascorbic acid prevents lethal gastrointestinal syndrome in mice receiving a massive amount of radiation. *J Radiat Res* **51**, 145-156 (2010).

1872. Yamamoto T*, et al.* Effect of glucagon on the xylitol-induced increase in the plasma concentration and urinary excretion of purine bases. *Metabolism* **45**, 1354-1359 (1996).

1873. Yamamoto Y*, et al.* Rapid alternative absorption of dietary long-chain fatty acids with upregulation of intestinal glycosylated CD36 in liver cirrhosis. *Am J Clin Nutr* **96**, 90-101 (2012).

1874. Yamane S, Kanno T, Nakamura H, Fujino H, Murayama T. Hydrogen sulfide-mediated regulation of contractility in the mouse ileum with electrical stimulation: roles of L-cysteine, cystathionine β-synthase, and K+ channels. *Eur J Pharmacol* **740**, 112-120 (2014).

1875. Yamashita S, Saitoh H, Nakanishi K, Masada M, Nadai T, Kimura T. Effects of diclofenac sodium and disodium ethylenediaminetetraacetate on electrical parameters of the mucosal membrane and their relation to the permeability enhancing effects in the rat jejunum. *J Pharm Pharmacol* **39**, 621-626 (1987).

1876. Yan F, Dibner JJ, Knight CD, Vazquez-Anon M. Effect of carbohydrase and protease on growth performance and gut health of young broilers fed diets containing rye, wheat, and feather meal. *Poult Sci* **96**, 817-828 (2017).

1877. Yang CY*, et al.* Effects of habitual chitosan intake on bone mass, bone-related metabolic markers and duodenum CaBP D9K mRNA in ovariectomized SHRSP rats. *J Nutr Sci Vitaminol (Tokyo)* **48**, 371-378 (2002).

1878. Yang G, Bibi S, Du M, Suzuki T, Zhu MJ. Regulation of the intestinal tight junction by natural polyphenols: A mechanistic perspective. *Crit Rev Food Sci Nutr* **57**, 3830-3839 (2017).

1879. Yang H, Grahn M, Schalch DS, Ney DM. Anabolic effect of IGF-I coinfused with total parenteral nutrition in dexamethasone-treated rats. *Am J Physiol* **266**, E690-698 (1994).

1880. Yang WZ, Benchaar C, Ametaj BN, Chaves AV, He ML, McAllister TA. Effects of garlic and juniper berry essential oils on ruminal fermentation and on the site and extent of digestion in lactating cows. *J Dairy Sci* **90**, 5671-5681 (2007).

1881. Yang Y, Jobin C. Novel insights into microbiome in colitis and colorectal cancer. *Curr Opin Gastroenterol* **33**, 422-427 (2017).

1882. Yang Y, Wu Z, Meininger CJ, Wu G. L-Leucine and NO-mediated cardiovascular function. *Amino Acids* **47**, 435-447 (2015).

1883. Yang Z, Liao SF. Physiological Effects of Dietary Amino Acids on Gut Health and Functions of Swine. *Front Vet Sci* **6**, 169 (2019).

1884. Yano H, Kinoshita S, Kira S. Effects of acute moderate exercise on the phagocytosis of Kupffer cells in rats. *Acta Physiol Scand* **182**, 151-160 (2004).

1885. Yao J, Gao P, Xu Y, Li Z. α-TEA inhibits the growth and motility of human colon cancer cells via targeting RhoA/ROCK signaling. *Mol Med Rep* **14**, 2534-2540 (2016).

1886. Yao K*, et al.* Improvement of carotenoid bioaccessibility from spinach by co-ingesting with excipient nanoemulsions: impact of the oil phase composition. *Food Funct* **10**, 5302-5311 (2019).

1887. Yarrington JT, Whiehair CK. Ultrastructure of gastointestinal smooth muscle in ducks with a vitamin E-selenium deficiency. *J Nutr* **105**, 782-790 (1975).

1888. Yasar U, Erdem SR, Tuncer M. Cyclosporine A preparations and their vehicles induce contraction of the guinea pig gallbladder in vitro: the role of cyclooxygenase metabolites. *Pharmacology* **58**, 309-318 (1999).

1889. Yeh JK, Aloia JF. Effect of cortisone on jejunal phosphate absorption in hypophysectomized rats. *Miner Electrolyte Metab* **10**, 398-403 (1984).

1890. Yeh JK, Aloia JF, Semla HM. Interrelation of cortisone and 1,25 dihydroxycholecalciferol on intestinal calcium and phosphate absorption. *Calcif Tissue Int* **36**, 608-614 (1984).

1891. Yeh K, Moog F. Intestinal lactase activity in the suckling rat: influence of hypophysectomy and thyroidectomy. *Science* **183**, 77-79 (1974).

1892. Yeh K, Yeh M, Holt PR, Alpers DH. Development and hormonal modulation of postnatal expression of intestinal alkaline phosphatase mRNA species and their encoded isoenzymes. *Biochem J* **301 ( Pt 3)**, 893-899 (1994).

1893. Yeh KY, Yeh M, Holt PR. Hormonal regulation of adaptive intestinal growth in artificially reared rat pups. *Am J Physiol* **253**, G802-808 (1987).

1894. Yeh KY, Yeh M, Holt PR. Differential effects of thyroxine and cortisone on jejunal sucrase expression in suckling rats. *Am J Physiol* **256**, G604-612 (1989).

1895. Yeh KY, Yeh M, Holt PR. Intestinal lactase expression and epithelial cell transit in hormone-treated suckling rats. *Am J Physiol* **260**, G379-384 (1991).

1896. Yeh KY, Yeh M, Holt PR. Thyroxine and cortisone cooperate to modulate postnatal intestinal enzyme differentiation in the rat. *Am J Physiol* **260**, G371-378 (1991).

1897. Yeh KY, Yeh M, Montgomery RK, Grand RJ, Holt PR. Cortisone and thyroxine modulate intestinal lactase and sucrase mRNA levels and activities in the suckling rat. *Biochem Biophys Res Commun* **180**, 174-180 (1991).

1898. Yehia SA, Elshafeey AH, Elsayed I. Pulsatile systems for colon targeting of budesonide: in vitro and in vivo evaluation. *Drug Deliv* **18**, 620-630 (2011).

1899. Yesmine S, Connolly K, Hill N, Coulson FR, Fenning AS. Electrophysiological, vasoactive, and gastromodulatory effects of stevia in healthy Wistar rats. *Planta Med* **79**, 909-915 (2013).

1900. Yin J*, et al.* Dietary arginine supplementation enhances intestinal expression of SLC7A7 and SLC7A1 and ameliorates growth depression in mycotoxin-challenged pigs. *Amino Acids* **46**, 883-892 (2014).

1901. Yina S, Chenghua L, Weiwei Z, Zhenhui W, Zhimeng L. The first description of complete invertebrate arginine metabolism pathways implies dose-dependent pathogen regulation in Apostichopus japonicus. *Sci Rep* **6**, 23783 (2016).

1902. Yoda A*, et al.* Eosinophilic enteritis: efficiency of the 13C-acetate breath test for assessing the disease activity. *Intern Med* **51**, 2551-2554 (2012).

1903. Yoon IS*, et al.* Cloning and heterologous expression of a Ca2+-activated chloride channel isoform from rat brain. *Biol Pharm Bull* **29**, 2168-2173 (2006).

1904. Yoshida S, Hayashi K, Kawasaki T. Pyridoxine transport in brush border membrane vesicles of guinea pig jejunum. *J Nutr Sci Vitaminol (Tokyo)* **27**, 311-317 (1981).

1905. Yoshida S*, et al.* Administration of opiate receptor antagonist inhibits mucosal atrophy of the gut in fasting rats. *J Surg Res* **93**, 177-181 (2000).

1906. Yoshida WB*, et al.* Effect of alpha-tocopherol, taurine and selenium on the attenuation of ischemia/reperfusion injury of splanchnic organs. *Cardiovasc Surg* **6**, 178-187 (1998).

1907. Yoshikawa T, Kondo M. [Free radicals in digestive diseases]. *Nihon Ronen Igakkai Zasshi* **27**, 155-160 (1990).

1908. Yoshizawa S, Sugisaki N, Moriuchi S, Hosoya N. The effect of 1alpha-hydroxyvitamin D3 and cortisone on the development of chick duodenal alkaline phosphatase in organ culture. *J Nutr Sci Vitaminol (Tokyo)* **22**, 21-28 (1976).

1909. Yoshizumi T*, et al.* Decreased immunoglobulin G levels after living-donor liver transplantation is a risk factor for bacterial infection and sepsis. *Transpl Infect Dis* **16**, 225-231 (2014).

1910. Young HM, Ciampoli D, Johnson PJ, Stebbing MJ. Inhibitory transmission to the longitudinal muscle of the mouse caecum is mediated largely by nitric oxide acting via soluble guanylyl cyclase. *J Auton Nerv Syst* **61**, 103-108 (1996).

1911. Youngs GR, Agnew JE, Levin GE, Bouchier IA. Radioselenium in duodenal aspirate as an assessment of pancreatic exocrine function. *Br Med J* **2**, 252-255 (1971).

1912. Yu S, Bruce D, Froicu M, Weaver V, Cantorna MT. Failure of T cell homing, reduced CD4/CD8alphaalpha intraepithelial lymphocytes, and inflammation in the gut of vitamin D receptor KO mice. *Proc Natl Acad Sci U S A* **105**, 20834-20839 (2008).

1913. Yu XH, Zhang DW, Zheng XL, Tang CK. Itaconate: an emerging determinant of inflammation in activated macrophages. *Immunol Cell Biol* **97**, 134-141 (2019).

1914. Yuan CH*, et al.* Protective effects of L-arginine on reperfusion injury after pancreaticoduodenal transplantation in rats. *Hepatobiliary Pancreat Dis Int* **3**, 349-354 (2004).

1915. Yuan CH, Liu YF, Liang J, Zhao N, He SG. Effects of nitric oxide on reperfusion injury following pancreaticoduodenal transplantation in rats. *Chin Med Sci J* **20**, 142-146 (2005).

1916. Yuan SY, Costa M, Brookes SJ. Neuronal control of the pyloric sphincter of the guinea-pig. *Neurogastroenterol Motil* **13**, 187-198 (2001).

1917. Yuan X, Liu X, McClements DJ, Cao Y, Xiao H. Enhancement of phytochemical bioaccessibility from plant-based foods using excipient emulsions: impact of lipid type on carotenoid solubilization from spinach. *Food Funct* **9**, 4352-4365 (2018).

1918. Yun CH*, et al.* Glucocorticoid stimulation of ileal Na+ absorptive cell brush border Na+/H+ exchange and association with an increase in message for NHE-3, an epithelial Na+/H+ exchanger isoform. *J Biol Chem* **268**, 206-211 (1993).

1919. Yüncü M, Eralp A, Koruk M, Sari I, Bağci C, Inalöz S. Effect of vitamin A against methotrexate-induced damage to the small intestine in rats. *Med Princ Pract* **13**, 346-352 (2004).

1920. Yunker AM, Galligan JJ. Endogenous NO inhibits NANC but not cholinergic neurotransmission to circular muscle of guinea pig ileum. *Am J Physiol* **271**, G904-912 (1996).

1921. Yuri T, Kono Y, Fujita T. Transport characteristics of 5-aminosalicylic acid into colonic epithelium: Involvement of sodium-coupled monocarboxylate transporter SMCT1-mediated transport system. *Biochem Biophys Res Commun* **524**, 561-566 (2020).

1922. Yuri T, Kono Y, Okada T, Terada T, Miyauchi S, Fujita T. Transport Characteristics of 5-Aminosalicylic Acid Derivatives Conjugated with Amino Acids via Human H(+)-Coupled Oligopeptide Transporter PEPT1. *Biol Pharm Bull* **43**, 697-706 (2020).

1923. Yusof M, Kamada K, Gaskin FS, Korthuis RJ. Angiotensin II mediates postischemic leukocyte-endothelial interactions: role of calcitonin gene-related peptide. *Am J Physiol Heart Circ Physiol* **292**, H3032-3037 (2007).

1924. Zaccagnino P, Saltarella M, D'Oria S, Corcelli A, Saponetti MS, Lorusso M. N-arachidonylglycine causes ROS production and cytochrome c release in liver mitochondria. *Free Radic Biol Med* **47**, 585-592 (2009).

1925. Zacharakis E*, et al.* Contribution of insulin-like growth factor I to the healing of colonic anastomoses in rats. *J Invest Surg* **20**, 9-14 (2007).

1926. Zadrozny LM, Stauffer SH, Armstrong MU, Jones SL, Gookin JL. Neutrophils do not mediate the pathophysiological sequelae of Cryptosporidium parvum infection in neonatal piglets. *Infect Immun* **74**, 5497-5505 (2006).

1927. Zagorodnyuk V, Santicioli P, Maggi CA, Giachetti A. Evidence that tachykinin NK1 and NK2 receptors mediate non-adrenergic non-cholinergic excitation and contraction in the circular muscle of guinea-pig duodenum. *Br J Pharmacol* **115**, 237-246 (1995).

1928. Zakaria el R, Althani A, Fawzi AA, Fituri OM. Molecular mechanisms of peritoneal dialysis-induced microvascular vasodilation. *Adv Perit Dial* **30**, 98-109 (2014).

1929. Zakaria el R, Garrison RN, Kawabe T, Harris PD. Direct peritoneal resuscitation from hemorrhagic shock: effect of time delay in therapy initiation. *J Trauma* **58**, 499-506; discussion 506-498 (2005).

1930. Zan J*, et al.* Role of ghrelin in small intestinal motility following pediatric intracerebral hemorrhage in mice. *Mol Med Rep* **16**, 6958-6966 (2017).

1931. Zani BG, Bohlen HG. Transport of extracellular l-arginine via cationic amino acid transporter is required during in vivo endothelial nitric oxide production. *Am J Physiol Heart Circ Physiol* **289**, H1381-1390 (2005).

1932. Zanoni JN, Buttow NC, Bazotte RB, Miranda Neto MH. Evaluation of the population of NADPH-diaphorase-stained and myosin-V myenteric neurons in the ileum of chronically streptozotocin-diabetic rats treated with ascorbic acid. *Auton Neurosci* **104**, 32-38 (2003).

1933. Zanoni JN, De Freitas P, Pereira RV, Dos Santos Pereira MA, De Miranda Neto MH. Effects of supplementation with ascorbic acid for a period of 120 days on the myosin-V and NADPHd positive myenteric neurons of the ileum of rats. *Anat Histol Embryol* **34**, 149-153 (2005).

1934. Zanoni JN, Freitas P. Effects of ascorbic acid on the vasoactive intestinal peptide synthesis in the ileum submucous plexus of normal rats. *Arq Gastroenterol* **42**, 186-190 (2005).

1935. Zanoni JN, Hernandes L, Bazotte RB, Miranda Neto MH. Terminal ileum submucous plexus: Study of the VIP-ergic neurons of diabetic rats treated with ascorbic acid. *Arq Neuropsiquiatr* **60**, 32-37 (2002).

1936. Zanoni JN, Tronchini EA, Moure SA, Souza ID. Effects of L-glutamine supplementation on the myenteric neurons from the duodenum and cecum of diabetic rats. *Arq Gastroenterol* **48**, 66-71 (2011).

1937. Zaror-Behrens G, Behrens WA, Madère R. Repletion and depletion of vitamin E in gastroduodenal mucosa. *Scand J Gastroenterol* **26**, 839-842 (1991).

1938. Zaror-Behrens G, Mueller R, Greselin E, Behrens WA. Lack of vitamin E cytoprotective effects on indomethacin-induced gastric lesions. *Res Commun Chem Pathol Pharmacol* **72**, 327-335 (1991).

1939. Zeng Y*, et al.* Vitamin D signaling maintains intestinal innate immunity and gut microbiota: potential intervention for metabolic syndrome and NAFLD. *Am J Physiol Gastrointest Liver Physiol* **318**, G542-g553 (2020).

1940. Zha A*, et al.* The Evaluation of the Antioxidant and Intestinal Protective Effects of Baicalin-Copper in Deoxynivalenol-Challenged Piglets. *Oxid Med Cell Longev* **2020**, 5363546 (2020).

1941. Zhan K, Jiang M, Gong X, Zhao G. Effect of short-chain fatty acids on the expression of genes involved in short-chain fatty acid transporters and inflammatory response in goat jejunum epithelial cells. *In Vitro Cell Dev Biol Anim* **54**, 311-320 (2018).

1942. Zhang B, Ji SQ, Hua YW, Liu YQ. [Application of jejunal interposition after radical proximal gastrectomy]. *Zhonghua Zhong Liu Za Zhi* **35**, 530-533 (2013).

1943. Zhang DM, Jiao RQ, Kong LD. High Dietary Fructose: Direct or Indirect Dangerous Factors Disturbing Tissue and Organ Functions. *Nutrients* **9**, (2017).

1944. Zhang K*, et al.* An herbal-compound-based combination therapy that relieves cirrhotic ascites by affecting the L-arginine/nitric oxide pathway: A metabolomics-based systematic study. *J Ethnopharmacol* **241**, 112034 (2019).

1945. Zhang L, Liu S, Piao X. Dietary 25-hydroxycholecalciferol supplementation improves performance, immunity, antioxidant status, intestinal morphology, and bone quality in weaned piglets. *J Sci Food Agric*, (2020).

1946. Zhang M, Ding CH, Zhang LW, Yang SY. [Effect of nitric oxide on the myoelectric activity of sphincter of Oddi and arterial blood pressure of rabbit]. *Sheng Li Xue Bao* **50**, 618-622 (1998).

1947. Zhang S, Myers S, Castro GA. Inhibition of anaphylaxis-evoked intestinal fluid secretion by the dual application of an H1 antagonist and cyclooxygenase inhibitor. *Gastroenterology* **100**, 922-928 (1991).

1948. Zhang T*, et al.* Disruption of De Novo Serine Synthesis in Muller Cells Induced Mitochondrial Dysfunction and Aggravated Oxidative Damage. *Mol Neurobiol* **55**, 7025-7037 (2018).

1949. Zhang X*, et al.* Study of the protective effects of dexamethasone on ileum mucosa injury in rats with severe acute pancreatitis. *Pancreas* **37**, e74-82 (2008).

1950. Zhang X, Okutsu M, Kanemi O, Nagatomi R. Effect of foot shock stress on the interferon-gamma production of murine intestinal intraepithelial lymphocytes. *Immunol Lett* **100**, 170-176 (2005).

1951. Zhang XP*, et al.* Preparation method of an ideal model of multiple organ injury of rat with severe acute pancreatitis. *World J Gastroenterol* **13**, 4566-4573 (2007).

1952. Zhang YP, Hao XQ, Zhang LM, Tian YT. Enhanced cyclooxygenase-2 activity leads to intestinal dysmotility following hemorrhagic shock. *Acta Cir Bras* **30**, 838-843 (2015).

1953. Zhang Z*, et al.* Influence of vitamin E tocopherol polyethylene glycol succinate 1000 on intestinal absorption of icariside II. *Pharmazie* **67**, 59-62 (2012).

1954. Zhang Z, Wang Y, Dong M, Cui J, Rong D, Dong Q. Oxymatrine ameliorates L-arginine-induced acute pancreatitis in rats. *Inflammation* **35**, 605-613 (2012).

1955. Zhao S*, et al.* Protective effect of seabuckthorn berry juice against acrylamide-induced oxidative damage in rats. *J Food Sci* **85**, 2245-2254 (2020).

1956. Zhao W, Ndisang JF, Wang R. Modulation of endogenous production of H2S in rat tissues. *Can J Physiol Pharmacol* **81**, 848-853 (2003).

1957. Zholos AV, Tsytsyura YD, Gordienko DV, Tsvilovskyy VV, Bolton TB. Phospholipase C, but not InsP3 or DAG, -dependent activation of the muscarinic receptor-operated cation current in guinea-pig ileal smooth muscle cells. *Br J Pharmacol* **141**, 23-36 (2004).

1958. Zhong Y*, et al.* Barley malt increases hindgut and portal butyric acid, modulates gene expression of gut tight junction proteins and Toll-like receptors in rats fed high-fat diets, but high advanced glycation end-products partially attenuate the effects. *Food Funct* **6**, 3165-3176 (2015).

1959. Zhou D*, et al.* Sodium butyrate attenuates high-fat diet-induced steatohepatitis in mice by improving gut microbiota and gastrointestinal barrier. *World J Gastroenterol* **23**, 60-75 (2017).

1960. Zhou J, Hao Z, Irwin N, Berthoud HR, Ye J. Gastric inhibitory polypeptide (GIP) is selectively decreased in the roux-limb of dietary obese mice after RYGB surgery. *PLoS One* **10**, e0134728 (2015).

1961. Zhou J*, et al.* Fructus Gardeniae-induced gastrointestinal injury was associated with the inflammatory response mediated by the disturbance of vitamin B6, phenylalanine, arachidonic acid, taurine and hypotaurine metabolism. *J Ethnopharmacol* **235**, 47-55 (2019).

1962. Zhou X, He L, Wu C, Zhang Y, Wu X, Yin Y. Serine alleviates oxidative stress via supporting glutathione synthesis and methionine cycle in mice. *Mol Nutr Food Res* **61**, (2017).

1963. Zhou X*, et al.* Serine prevented high-fat diet-induced oxidative stress by activating AMPK and epigenetically modulating the expression of glutathione synthesis-related genes. *Biochim Biophys Acta Mol Basis Dis* **1864**, 488-498 (2018).

1964. Zimmerman BJ, Grisham MB, Granger DN. Role of oxidants in ischemia/reperfusion-induced granulocyte infiltration. *Am J Physiol* **258**, G185-190 (1990).

1965. Zółtowska K, Olender H, Dziekońska-Rynko J, Jabłonowski Z. [The influence of various levels of carotene and protein in the diet on the index of invasion and digestive enzyme activity in chickens infected with Ascaridia galli]. *Wiad Parazytol* **42**, 65-69 (1996).

1966. Zséli J, Török TL, Vizi SE, Knoll J. Effect of prostaglandin E1 and indomethacin on responses of longitudinal muscle of guinea-pig ileum to cholecystokinin. *Eur J Pharmacol* **56**, 139-144 (1979).

1967. Zuo DC*, et al.* Action of lipopolysaccharide on interstitial cells of cajal from mouse small intestine. *Pharmacology* **90**, 151-159 (2012).

1968. Zuo DM, Zhang LY, Lu X, Liu Y, Chen ZL. Protective role of mouse MBL-C on intestinal mucosa during Shigella flexneri invasion. *Int Immunol* **21**, 1125-1134 (2009).

^1, 2, 3, 4, 5, 6, 7, 8, 9, 10, 11, 12, 13, 14, 15, 16, 17, 18, 19, 20, 21, 22, 23, 24, 25, 26, 27, 28, 29, 30, 31, 32, 33, 34, 35, 36, 37, 38, 39, 40, 41, 42, 43, 44, 45, 46, 47, 48, 49, 50, 51, 52, 53, 54, 55, 56, 57, 58, 59, 60, 61, 62, 63, 64, 65, 66, 67, 68, 69^

^70, 71, 72, 73, 74, 75, 76, 77, 78, 79, 80, 81, 82, 83, 84, 85, 86, 87, 88, 89, 90, 91, 92, 93, 94, 95, 96, 97, 98, 99, 100, 101, 102, 103, 104, 105, 106, 107, 108, 109, 110, 111, 112, 113, 114, 115, 116, 117, 118, 119, 120, 121, 122, 123, 124, 125, 126, 127, 128, 129, 130, 131, 132, 133, 134^

^135, 136, 137, 138, 139, 140, 141, 142, 143, 144, 145, 146, 147, 148, 149, 150, 151, 152, 153, 154, 155, 156, 157, 158, 159, 160, 161, 162, 163, 164, 165, 166, 167, 168, 169, 170, 171, 172, 173, 174, 175, 176, 177, 178, 179, 180, 181, 182, 183, 184, 185, 186, 187, 188, 189, 190, 191, 192, 193, 194, 195, 196, 197, 198, 199, 200, 201, 202, 203^

^204, 205, 206, 207, 208, 209, 210, 211, 212, 213, 214, 215, 216, 217, 218, 219, 220, 221, 222, 223, 224, 225, 226, 227, 228, 229, 230, 231, 232, 233, 234, 235, 236, 237, 238, 239, 240, 241, 242, 243, 244, 245, 246, 247, 248, 249, 250, 251, 252, 253, 254, 255, 256, 257, 258, 259, 260, 261, 262, 263, 264, 265, 266, 267, 268, 269, 270, 271, 272, 273, 274, 275, 276, 277, 278, 279, 280, 281, 282, 283, 284^

^285, 286, 287, 288, 289, 290, 291, 292, 293, 294, 295, 296, 297, 298, 299, 300, 301, 302, 303, 304, 305, 306, 307, 308, 309, 310, 311, 312, 313, 314, 315, 316, 317, 318, 319, 320, 321, 322, 323, 324, 325, 326, 327, 328, 329, 330, 331, 332, 333, 334, 335, 336, 337, 338, 339, 340, 341, 342, 343, 344, 345, 346, 347, 348, 349, 350, 351, 352, 353, 354, 355, 356, 357, 358, 359, 360, 361, 362, 363, 364, 365^

^366, 367, 368, 369, 370, 371, 372, 373, 374, 375, 376, 377, 378, 379, 380, 381, 382, 383, 384, 385, 386, 387, 388, 389, 390, 391, 392, 393, 394, 395, 396, 397, 398, 399, 400, 401, 402, 403, 404, 405, 406, 407, 408, 409, 410, 411, 412, 413, 414, 415, 416, 417, 418, 419, 420, 421, 422, 423, 424, 425, 426, 427, 428, 429, 430, 431, 432, 433, 434, 435, 436, 437, 438, 439, 440, 441, 442, 443, 444, 445, 446, 447, 448, 449, 450^

^451, 452, 453, 454, 455, 456, 457, 458, 459, 460, 461, 462, 463, 464, 465, 466, 467, 468, 469, 470, 471, 472, 473, 474, 475, 476, 477, 478, 479, 480, 481, 482, 483, 484, 485, 486, 487, 488, 489, 490, 491, 492, 493, 494, 495, 496, 497, 498, 499, 500, 501, 502, 503, 504, 505, 506, 507, 508, 509, 510, 511, 512, 513, 514, 515, 516, 517, 518, 519, 520, 521, 522, 523, 524, 525, 526, 527, 528, 529, 530^

^531, 532, 533, 534, 535, 536, 537, 538, 539, 540, 541, 542, 543, 544, 545, 546, 547, 548, 549, 550, 551, 552, 553, 554, 555, 556, 557, 558, 559, 560, 561, 562, 563, 564, 565, 566, 567, 568, 569, 570, 571, 572, 573, 574, 575, 576, 577, 578, 579, 580, 581, 582, 583, 584, 585, 586, 587, 588, 589, 590, 591, 592, 593, 594, 595, 596, 597, 598, 599, 600, 601, 602, 603, 604, 605, 606, 607, 608, 609, 610, 611, 612, 613^

^614, 615, 616, 617, 618, 619, 620, 621, 622, 623, 624, 625, 626, 627, 628, 629, 630, 631, 632, 633, 634, 635, 636, 637, 638, 639, 640, 641, 642, 643, 644, 645, 646, 647, 648, 649, 650, 651, 652, 653, 654, 655, 656, 657, 658, 659, 660, 661, 662, 663, 664, 665, 666, 667, 668, 669, 670, 671, 672, 673, 674, 675, 676, 677, 678, 679, 680, 681, 682, 683, 684, 685, 686, 687, 688, 689, 690, 691, 692, 693, 694, 695, 696, 697, 698^

^699, 700, 701, 702, 703, 704, 705, 706, 707, 708, 709, 710, 711, 712, 713, 714, 715, 716, 717, 718, 719, 720, 721, 722, 723, 724, 725, 726, 727, 728, 729, 730, 731, 732, 733, 734, 735, 736, 737, 738, 739, 740, 741, 742, 743, 744, 745, 746, 747, 748, 749, 750, 751, 752, 753, 754, 755, 756, 757, 758, 759, 760, 761, 762, 763, 764, 765, 766, 767, 768, 769, 770, 771, 772, 773, 774, 775, 776, 777, 778, 779, 780, 781^

^782, 783, 784, 785, 786, 787, 788, 789, 790, 791, 792, 793, 794, 795, 796, 797, 798, 799, 800, 801, 802, 803, 804, 805, 806, 807, 808, 809, 810, 811, 812, 813, 814, 815, 816, 817, 818, 819, 820, 821, 822, 823, 824, 825, 826, 827, 828, 829, 830, 831, 832, 833, 834, 835, 836, 837, 838, 839, 840, 841, 842, 843, 844, 845, 846, 847, 848, 849, 850, 851, 852, 853, 854, 855, 856, 857, 858, 859, 860, 861, 862, 863^

^864, 865, 866, 867, 868, 869, 870, 871, 872, 873, 874, 875, 876, 877, 878, 879, 880, 881, 882, 883, 884, 885, 886, 887, 888, 889, 890, 891, 892, 893, 894, 895, 896, 897, 898, 899, 900, 901, 902, 903, 904, 905, 906, 907, 908, 909, 910, 911, 912, 913, 914, 915, 916, 917, 918, 919, 920, 921, 922, 923, 924, 925, 926, 927, 928, 929, 930, 931, 932, 933, 934, 935, 936, 937, 938, 939, 940, 941, 942, 943^

^944, 945, 946, 947, 948, 949, 950, 951, 952, 953, 954, 955, 956, 957, 958, 959, 960, 961, 962, 963, 964, 965, 966, 967, 968, 969, 970, 971, 972, 973, 974, 975, 976, 977, 978, 979, 980, 981, 982, 983, 984, 985, 986, 987, 988, 989, 990, 991, 992, 993, 994, 995, 996, 997, 998, 999, 1000, 1001, 1002, 1003, 1004, 1005, 1006, 1007, 1008, 1009, 1010, 1011, 1012, 1013, 1014, 1015, 1016, 1017, 1018, 1019, 1020^

^1021, 1022, 1023, 1024, 1025, 1026, 1027, 1028, 1029, 1030, 1031, 1032, 1033, 1034, 1035, 1036, 1037, 1038, 1039, 1040, 1041, 1042, 1043, 1044, 1045, 1046, 1047, 1048, 1049, 1050, 1051, 1052, 1053, 1054, 1055, 1056, 1057, 1058, 1059, 1060, 1061, 1062, 1063, 1064, 1065, 1066, 1067, 1068, 1069, 1070, 1071, 1072, 1073, 1074, 1075, 1076, 1077, 1078, 1079, 1080, 1081, 1082, 1083, 1084, 1085, 1086, 1087, 1088, 1089, 1090, 1091, 1092, 1093, 1094, 1095, 1096, 1097, 1098, 1099^

^1100, 1101, 1102, 1103, 1104, 1105, 1106, 1107, 1108, 1109, 1110, 1111, 1112, 1113, 1114, 1115, 1116, 1117, 1118, 1119, 1120, 1121, 1122, 1123, 1124, 1125, 1126, 1127, 1128, 1129, 1130, 1131, 1132, 1133, 1134, 1135, 1136, 1137, 1138, 1139, 1140, 1141, 1142, 1143, 1144, 1145, 1146, 1147, 1148, 1149, 1150, 1151, 1152, 1153, 1154, 1155, 1156, 1157, 1158, 1159, 1160, 1161, 1162, 1163, 1164, 1165, 1166, 1167, 1168, 1169, 1170, 1171, 1172, 1173, 1174, 1175, 1176, 1177^

^1178, 1179, 1180, 1181, 1182, 1183, 1184, 1185, 1186, 1187, 1188, 1189, 1190, 1191, 1192, 1193, 1194, 1195, 1196, 1197, 1198, 1199, 1200, 1201, 1202, 1203, 1204, 1205, 1206, 1207, 1208, 1209, 1210, 1211, 1212, 1213, 1214, 1215, 1216, 1217, 1218, 1219, 1220, 1221, 1222, 1223, 1224, 1225, 1226, 1227, 1228, 1229, 1230, 1231, 1232, 1233, 1234, 1235, 1236, 1237, 1238, 1239, 1240, 1241, 1242, 1243, 1244, 1245, 1246, 1247, 1248, 1249, 1250, 1251, 1252, 1253, 1254, 1255, 1256, 1257, 1258^

^1259, 1260, 1261, 1262, 1263, 1264, 1265, 1266, 1267, 1268, 1269, 1270, 1271, 1272, 1273, 1274, 1275, 1276, 1277, 1278, 1279, 1280, 1281, 1282, 1283, 1284, 1285, 1286, 1287, 1288, 1289, 1290, 1291, 1292, 1293, 1294, 1295, 1296, 1297, 1298, 1299, 1300, 1301, 1302, 1303, 1304, 1305, 1306, 1307, 1308, 1309, 1310, 1311, 1312, 1313, 1314, 1315, 1316, 1317, 1318, 1319, 1320, 1321, 1322, 1323, 1324, 1325, 1326, 1327, 1328, 1329, 1330, 1331, 1332, 1333, 1334, 1335, 1336, 1337, 1338, 1339, 1340, 1341, 1342, 1343^

^1344, 1345, 1346, 1347, 1348, 1349, 1350, 1351, 1352, 1353, 1354, 1355, 1356, 1357, 1358, 1359, 1360, 1361, 1362, 1363, 1364, 1365, 1366, 1367, 1368, 1369, 1370, 1371, 1372, 1373, 1374, 1375, 1376, 1377, 1378, 1379, 1380, 1381, 1382, 1383, 1384, 1385, 1386, 1387, 1388, 1389, 1390, 1391, 1392, 1393, 1394, 1395, 1396, 1397, 1398, 1399, 1400, 1401, 1402, 1403, 1404, 1405, 1406, 1407, 1408, 1409, 1410, 1411, 1412, 1413, 1414, 1415, 1416, 1417, 1418, 1419, 1420, 1421, 1422, 1423, 1424, 1425^

^1426, 1427, 1428, 1429, 1430, 1431, 1432, 1433, 1434, 1435, 1436, 1437, 1438, 1439, 1440, 1441, 1442, 1443, 1444, 1445, 1446, 1447, 1448, 1449, 1450, 1451, 1452, 1453, 1454, 1455, 1456, 1457, 1458, 1459, 1460, 1461, 1462, 1463, 1464, 1465, 1466, 1467, 1468, 1469, 1470, 1471, 1472, 1473, 1474, 1475, 1476, 1477, 1478, 1479, 1480, 1481, 1482, 1483, 1484, 1485, 1486, 1487, 1488, 1489, 1490, 1491, 1492, 1493, 1494, 1495, 1496, 1497, 1498, 1499, 1500, 1501, 1502, 1503, 1504, 1505^

^1506, 1507, 1508, 1509, 1510, 1511, 1512, 1513, 1514, 1515, 1516, 1517, 1518, 1519, 1520, 1521, 1522, 1523, 1524, 1525, 1526, 1527, 1528, 1529, 1530, 1531, 1532, 1533, 1534, 1535, 1536, 1537, 1538, 1539, 1540, 1541, 1542, 1543, 1544, 1545, 1546, 1547, 1548, 1549, 1550, 1551, 1552, 1553, 1554, 1555, 1556, 1557, 1558, 1559, 1560, 1561, 1562, 1563, 1564, 1565, 1566, 1567, 1568, 1569, 1570, 1571, 1572, 1573, 1574, 1575, 1576, 1577, 1578, 1579, 1580, 1581, 1582, 1583, 1584, 1585, 1586, 1587^

^1588, 1589, 1590, 1591, 1592, 1593, 1594, 1595, 1596, 1597, 1598, 1599, 1600, 1601, 1602, 1603, 1604, 1605, 1606, 1607, 1608, 1609, 1610, 1611, 1612, 1613, 1614, 1615, 1616, 1617, 1618, 1619, 1620, 1621, 1622, 1623, 1624, 1625, 1626, 1627, 1628, 1629, 1630, 1631, 1632, 1633, 1634, 1635, 1636, 1637, 1638, 1639, 1640, 1641, 1642, 1643, 1644, 1645, 1646, 1647, 1648, 1649, 1650, 1651, 1652, 1653, 1654, 1655, 1656, 1657, 1658, 1659, 1660, 1661, 1662, 1663, 1664, 1665, 1666, 1667^

^1668, 1669, 1670, 1671, 1672, 1673, 1674, 1675, 1676, 1677, 1678, 1679, 1680, 1681, 1682, 1683, 1684, 1685, 1686, 1687, 1688, 1689, 1690, 1691, 1692, 1693, 1694, 1695, 1696, 1697, 1698, 1699, 1700, 1701, 1702, 1703, 1704, 1705, 1706, 1707, 1708, 1709, 1710, 1711, 1712, 1713, 1714, 1715, 1716, 1717, 1718, 1719, 1720, 1721, 1722, 1723, 1724, 1725, 1726, 1727, 1728, 1729, 1730, 1731, 1732, 1733, 1734, 1735, 1736, 1737, 1738, 1739, 1740, 1741, 1742, 1743, 1744, 1745, 1746^

^1747, 1748, 1749, 1750, 1751, 1752, 1753, 1754, 1755, 1756, 1757, 1758, 1759, 1760, 1761, 1762, 1763, 1764, 1765, 1766, 1767, 1768, 1769, 1770, 1771, 1772, 1773, 1774, 1775, 1776, 1777, 1778, 1779, 1780, 1781, 1782, 1783, 1784, 1785, 1786, 1787, 1788, 1789, 1790, 1791, 1792, 1793, 1794, 1795, 1796, 1797, 1798, 1799, 1800, 1801, 1802, 1803, 1804, 1805, 1806, 1807, 1808, 1809, 1810, 1811, 1812, 1813, 1814, 1815, 1816, 1817, 1818, 1819, 1820, 1821, 1822, 1823, 1824, 1825, 1826^

^1827, 1828, 1829, 1830, 1831, 1832, 1833, 1834, 1835, 1836, 1837, 1838, 1839, 1840, 1841, 1842, 1843, 1844, 1845, 1846, 1847, 1848, 1849, 1850, 1851, 1852, 1853, 1854, 1855, 1856, 1857, 1858, 1859, 1860, 1861, 1862, 1863, 1864, 1865, 1866, 1867, 1868, 1869, 1870, 1871, 1872, 1873, 1874, 1875, 1876, 1877, 1878, 1879, 1880, 1881, 1882, 1883, 1884, 1885, 1886, 1887, 1888, 1889, 1890, 1891, 1892, 1893, 1894, 1895, 1896, 1897, 1898, 1899, 1900, 1901, 1902, 1903, 1904, 1905^

^1906, 1907, 1908, 1909, 1910, 1911, 1912, 1913, 1914, 1915, 1916, 1917, 1918, 1919, 1920, 1921, 1922, 1923, 1924, 1925, 1926, 1927, 1928, 1929, 1930, 1931, 1932, 1933, 1934, 1935, 1936, 1937, 1938, 1939, 1940, 1941, 1942, 1943, 1944, 1945, 1946, 1947, 1948, 1949, 1950, 1951, 1952, 1953, 1954, 1955, 1956, 1957, 1958, 1959, 1960, 1961, 1962, 1963, 1964, 1965, 1966, 1967, 1968^
